# Cys–Cys and Cys–Lys Stapling of Unprotected Peptides Enabled by Hypervalent Iodine Reagents

**DOI:** 10.1002/anie.202014511

**Published:** 2021-03-08

**Authors:** Javier Ceballos, Elija Grinhagena, Gontran Sangouard, Christian Heinis, Jerome Waser

**Affiliations:** ^1^ Laboratory of Catalysis and Organic Synthesis Ecole Polytechnique Fédérale de Lausanne EPFL SB ISIC LCSO, BCH 1402 1015 Lausanne Switzerland; ^2^ Laboratory of Therapeutic Proteins and Peptides Ecole Polytechnique Fédérale de Lausanne, EPFL SB ISIC LPPT, BCH 5305 1015 Lausanne Switzerland

**Keywords:** bioconjugation, helicity, hypervalent iodine reagents, late-stage functionalization, peptide stapling

## Abstract

Easy access to a wide range of structurally diverse stapled peptides is crucial for the development of inhibitors of protein‐protein interactions. Herein, we report bis‐functional hypervalent iodine reagents for two‐component cysteine‐cysteine and cysteine‐lysine stapling yielding structurally diverse thioalkyne linkers. This stapling method works with unprotected natural amino acid residues and does not require pre‐functionalization or metal catalysis. The products are stable to purification and isolation. Post‐stapling modification can be accessed via amidation of an activated ester, or via cycloaddition onto the formed thioalkyne group. Increased helicity and binding affinity to MDM2 was obtained for a *i,i+*7 stapled peptide.

## Graphical abstract

Bifunctional hypervalent iodine reagents for two‐component cysteine‐cysteine and cysteine‐lysine stapling are reported. Efficient and selective stapling on non‐protected peptides was observed, and post‐stapling modification could be easily achieved using reactive esters or cycloaddition on the thioalkynes formed on the cysteine residue. One stapled peptide displayed both enhanced helicity and binding to the MDM2 protein.

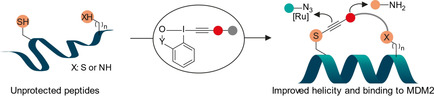

## Introduction

**Scheme 1:**
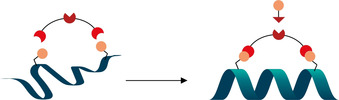
Two‐component stapling strategy and post‐stapling modification to introduce structurally diverse linkers.

Protein‐protein interactions (PPI) mediate a wide array of signaling pathways in the cell. Many of such interactions involve binding through α‐helical sequences. Identification and synthesis of these fragments therefore represents a powerful starting point for developing PPI inhibitors for drug discovery.[^1^, ^2^] Nevertheless, the helical conformation of short peptides is less stable than when the sequences are part of proteins, and they are rapidly degraded by proteases. Stapling—covalently linking two amino acids residues of peptides on the same face of an α‐helix—has been shown to enforce the helical conformation and improve the stability of peptides. It also enhances their cell membrane permeability and therefore their potential to be used as drugs.^[3]^ When developing a bioactive stapled peptide, the structure, length, lipophilicity and reactivity of the introduced linker is important.[^4^, ^5^] The linkers can be used to improve the solubility and the overall binding affinity of the peptide, not only by inducing helical conformation, but also through direct interaction with the binding protein. Therefore, during the search of prospective drug candidates, an easy access to a library of stapled peptides with different covalent linkers is important to increase the chance of finding active inhibitors and tune the properties of the lead compounds. The introduction of linker variability has been mainly addressed by two‐component stapling strategies between modified peptides and reactive small organic molecules (Scheme 1).^[3]^ Natural or non‐natural amino acids bearing reactive functional groups are introduced on the peptide during solid‐state synthesis, then reaction with a bi‐functional linker yields the desired stapled peptide. Further modification is then possible using an orthogonal functional group on the linker. In addition, the stapled peptides can not only be used as pharmaceuticals, but also as chemical biology tools.^[6]^ In the latter case, linkers with biorthogonal handles enabling in vivo visualization or target identification are especially useful.

**Scheme 2:**
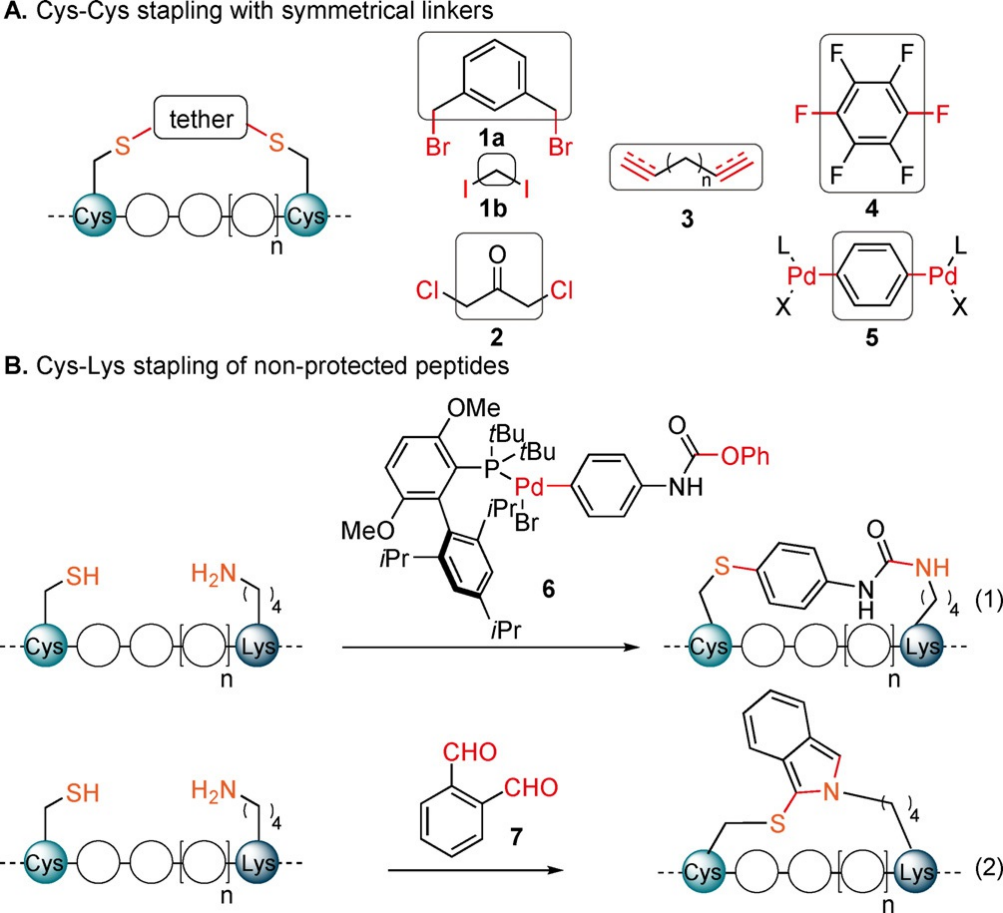
Previous Cys–Cys (A) and Cys–Lys (B) stapling strategies.

While the use of non‐natural amino acids provides good selectivity and reactivity, the required building blocks are less readily available and stapling often requires metal catalysis, which can be inconvenient and challenging on peptides.^[7]^ For these reasons, stapling methods using natural amino acids have been developed. Cysteine has been the most broadly used amino acid, due to its rare presence and high reactivity.^[8]^ The main reagents used have been either Michael acceptors or benzyl, allyl or alkyl halides **1** and dichloroacetone **2** (Scheme 2, **A**).^[9]^ More recently, thioyne/ene (**B**, reagents **3**),^[10]^ and cysteine arylation (**C**, reagents **4** and **5**)^[11]^ stapling techniques have been reported by the Chou and the Pentelute laboratories, respectively. Despite this important progress, there is still a strong need for the introduction of structurally diverse linkers reacting efficiently and with high selectivity towards cysteines.

When using two cysteines for stapling, the method is limited to symmetric linkers. To further expand linker variability, asymmetric reagents have been recently developed to staple two different natural amino acids. This strategy has been less explored, with only few reports on stapling between Lys and Glu or Asp[^3^, ^8b^] or Cys.^[12]^ Similarly to cysteine, lysine displays a strong nucleophilicity and is therefore ideally suited for labelling. However, when both residues are present, the selective introduction of a non‐symmetrical tether becomes challenging. Previous approaches for Cys–Lys stapling therefore required the use of non‐standard protecting groups^[12a]^ or the pre‐functionalization of either Cys^[12b]^ or both amino acids,^[12c]^ which led to multi‐step syntheses. More efficient approaches have been reported recently: Pentelute, Buchwald and co‐workers developed a highly selective palladium complex **6** containing an active carbamate for Cys–Lys stapling^[12d]^ [Scheme 2, **B**, Eq. (1)]. The need to synthesize palladium complexes is a drawback of this approach and limits structural diversity. The Li and Perrin groups independently reported a method using *ortho*‐phthalaldehyde (**7**) as a cheap and broadly available Cys–Lys linker [Scheme 2, **B**, Eq. (2)].[^12e^, ^12f^] Interestingly, the obtained isoindole could be readily functionalized by reaction with maleimide derivatives^[12e]^ or provided fluorescent peptides directly.^[12f]^ However, instability of the linker was observed, which could limit applications in vivo.^[12e]^ Other amino acids have been targeted for stapling with Lys by means of multicomponent reaction‐based techniques,[^8b^, ^13^] including the use of propargylated azaglycine‐Lysine A^3^,^[13a]^ *N* and *C* termini Ugi‐based cyclization,^[13b]^ or the stapling of two lysines by a Petasis reaction.^[13c]^ There is still a clear demand for simple bifunctional linkers reacting with high selectivity on non‐protected peptides that are well‐suited for structural modifications.

**Scheme 3:**
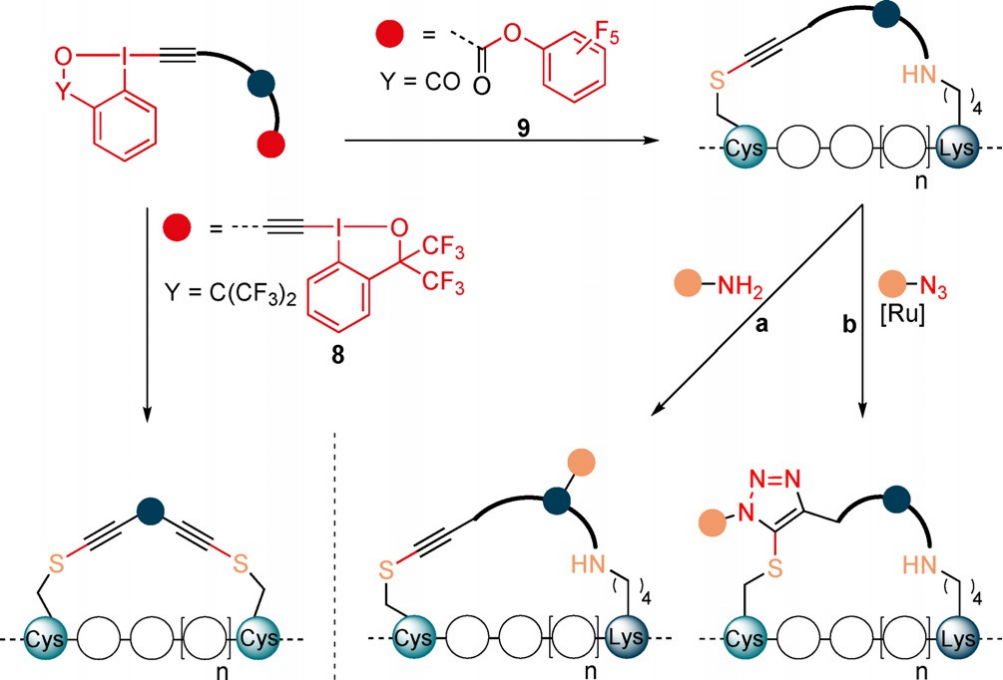
Our strategy for Cys–Cys and Cys–Lys stapling and post‐stapling functionalization using hypervalent iodine reagents.

Our group reported in 2013 a mild alkynylation of thiols using ethynyl‐1,2‐benziodoxol‐3(1*H*)‐ones (EBXs) hypervalent iodine reagents.^[14]^ We later demonstrated that these reagents were able to selectively label cysteine on peptides and proteins.^[15]^ Building on this work, we envisioned that the observed reactivity and selectivity towards Cys could be applied to the stapling of α‐helices without the need of protecting or pre‐functionalizing amino acid residues (Scheme 3). For Cys–Cys stapling EBX‐based dimer reagents **8** with two reactive hypervalent iodine warheads were designed. For Cys–Lys stapling an activated ester was introduced on the EBX reagent providing asymmetric linkers **9**. After reaction of the hypervalent iodine reagent with cysteine, the ester group of the reagent **9** would then react with a nearby amine. The tether that links the two electrophilic functional groups could be used to introduce structural variability in the stapled peptides and as a reactive group for further functionalization. Furthermore, the thioalkyne group present on the tether may open the possibility of further functionalization via [3+2] cycloaddition.

Herein, we report the use of EBX‐derived reagents **8** and **9** for Cys–Cys and Cys–Lys stapling of unprotected peptides. The obtained stapled peptides were stable, allowing purification and characterization. Post‐stapling modifications were possible using either an additional activated ester or the formed thioalkyne (**a** and **b** in Scheme 3). The impact of stapling on helicity was studied showing improved helicity with several peptides. Finally, this technology was applied to staple an α‐helical p53 derived peptide that binds to MDM2, an important cancer target.^[16]^ One stapled peptide was demonstrated to be an efficient inhibitor of MDM2 with a *K*_d_ of 29±4 nM showing a 12 times increase of potency compared to the linear peptide, emphasizing the substantial effect of the enhanced helicity.

## Results and Discussion

**Scheme 4:**
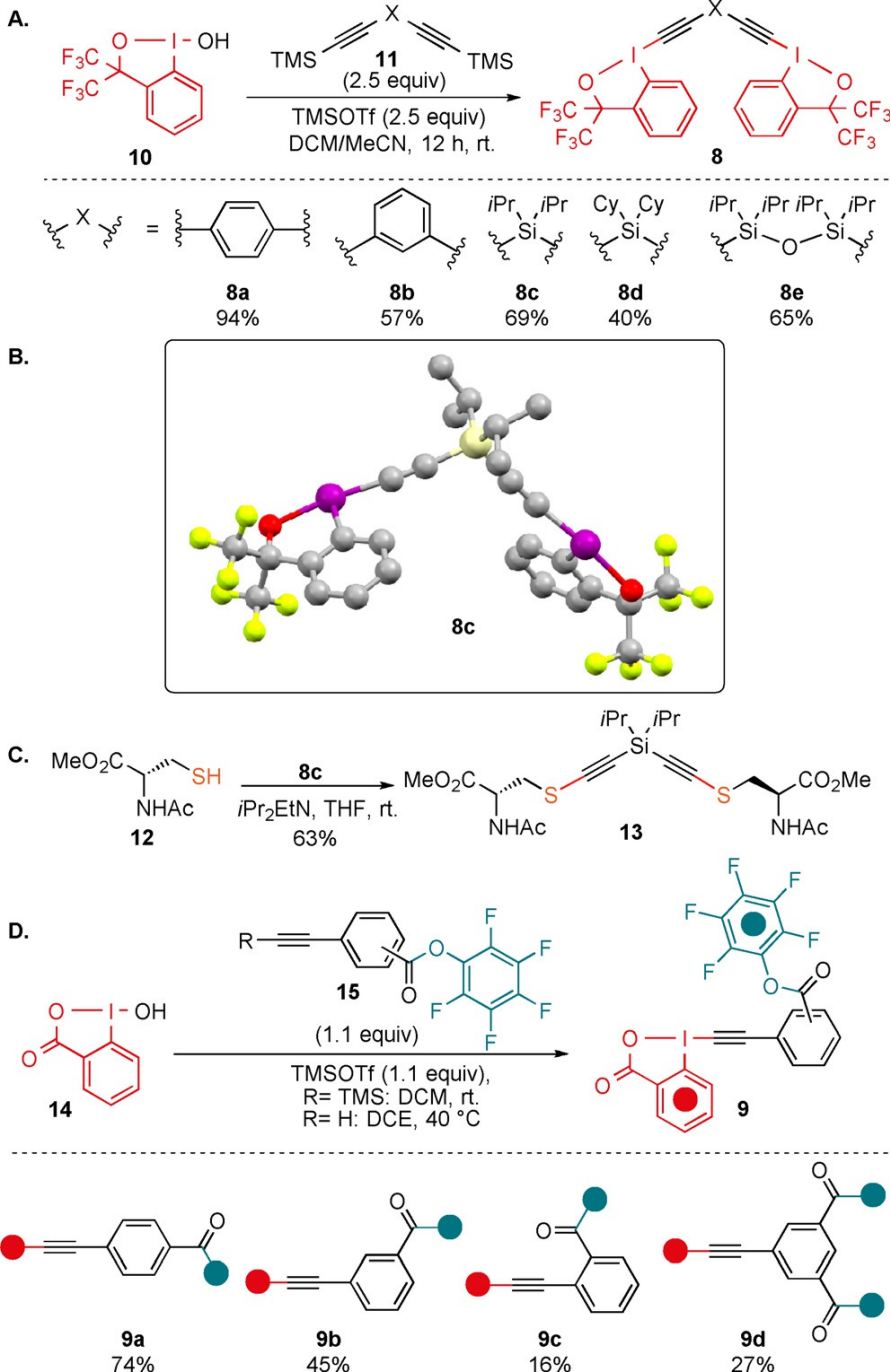
A) Synthesis of bis‐hypervalent iodine reagents **8**. B) Crystal structure of compound **8 c**. C) Reaction of **8 c** with *N*‐Acetyl‐L‐cysteine methyl ester (**12**). D) Synthesis of hypervalent iodine‐activated ester reagents **9**.

We started our study with the investigation of potential Cys–Cys stapling reagents. After several attempts, it was concluded that the frequently used more reactive 2‐iodobenzoic acid based‐EBX reagents[^14^, ^15^] were not suitable for the synthesis of dimeric reagents. First, their synthesis was long and low yielding. Second, they exhibited poor solubility in most solvents. Third, even when better solubility could be achieved, they were excessively strong oxidants, leading to disulfide formation as major pathway. Consequently, we turned our focus to the bis‐CF_3_ substituted hypervalent iodine reagents (1‐ethynyl‐3,3‐bis(trifluoromethyl)‐1,3‐dihydro‐1l3‐benzo[d][1,2]iodoxole), which exhibit a lower oxidation potential and reactivity, as well as higher solubility.^[17]^ First, the only reported aryl‐derived bis‐CF_3_ benziodoxole dimer **8 a** was synthesized (Scheme 4 A).^[17d]^ Reagent **8 a** has been previously used for the synthesis of multi‐substituted furans, but has never been applied for the alkynylation of thiols. The *meta* substituted aryl reagent **8 b** was also synthesized, as the geometry of the linker was expected to have a strong influence on stapling. In addition, new reagents **8 c**–**e** bearing silicon linkers of different lengths could also be accessed. Silicon‐substituted EBX reagents were more reactive towards thiols than alkyl‐ or aryl‐substituted analogues and led more efficiently to the alkyne product in our previous work.[^14^, ^15^] All reagents were obtained in one step from the known hypervalent iodine reagent **10** ^[18]^ in 40–94 % yield. The structure of **8 c** was further confirmed by X‐ray analysis (Scheme 4 B).^[19]^ Reagent **8 c** reacted cleanly with *N*‐Acetyl‐L‐cysteine methyl ester (**12**) to give bis‐thioalkyne **13** in 63 % yield, demonstrating that this class of benziodoxole reagents can also be used for thioalkynylation (Scheme 4 C).

Next, potential reagents for Cys–Lys stapling were prepared. In order to enable reaction with Lys (K), a commonly used pentafluoro phenyl (PFP) ester was selected.^[20]^ PFP esters are easily accessible and stable, but still reactive enough to efficiently yield amides. Having a single hypervalent iodine center allowed us to move back to the more reactive and more efficiently synthesized EBX core based on 2‐iodobenzoic acid. We also anticipated that reactivity with these EBX reagents should be sufficient with aryl substituents and decided to focus on substituted phenyl rings as tethers. Furthermore, the geometry and length of the linker can be readily modified with different substitution patterns at the phenyl ring. The *para*‐reagent **9 a** and the *meta*‐reagent **9 b** were accessed in 74 % and 45 % yield, respectively. The *ortho*‐substitution pattern was more difficult to synthesize, but the EBX **9 c** could nevertheless be obtained in 16 % yield. Finally, the highly sensitive reagent **9 d** bearing two activated esters could be accessed in 27 % yield. While the best result was obtained when a TMS protected alkyne was used, the reagents could also be accessed in slightly lower yields using directly the terminal alkynes (see Supporting Information, Section 4b).

With the reagents in hand, we moved to apply them on a peptide model (Table 1). We selected an axin‐derived peptide **16**,^[21]^ with the sequence of Ac‐ENPE**C**ILD**C**HVQRVM, which: (1) has been reported for cysteine‐cysteine (CC) stapling; (2) has been described to display low degree of helicity in solution as a linear peptide, but a high degree when stapled; (3) contains histidine (H), arginine (R), methionine (M) and glutamic acid (E) as potential competing nucleophilic residues.

**Table 1:** Optimization of the reaction conditions on peptide model Ac‐ENPE**C**ILD**C**HVQRVM (**16**).^[a]^ 
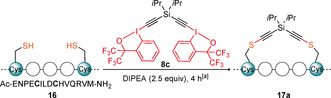

Entry	Reagent [equiv]	Solvent	Conc. [mM]^[b]^	*T* [°C]	Yield [%]^[c]^
1	1.0	DMF	1	23	55
2	1.5	DMF	1	23	67
3	3.0	DMF	1	23	72
4	5.0	DMF	1	23	73
5	3.0	DMF	1	37	78 (98^[d]^)
6	3.0	DMF	5	37	66^[e]^
7	3.0	DMSO	1	37	62
8	3.0	THF^[f]^	1	37	5
9	3.0	Dioxane^[f]^	1	37	0
10	3.0	DMF/Water (1.1:1)	1	37	0

[a] See Supporting Information (Section 8, Table S1–4) for an optimization of the base, the equivalents of base and the reaction time. [b] The peptide was dissolved in the indicated solvent (1 mM) and the base and reagent solutions were added making the final concentration not lower than 0.94 mM. [c] Calibrated yields base on absorbance at 210 nm (see Supporting Information, Figure S1). All yields are an average of duplicated reactions. [d] Relative absorbance of **17 a** vs. **16** at 210 nm. [e] Reaction time: 30 min. [f] 10 % of DMF was added to increase the solubility.

To optimize the conditions reagent **8 c** was chosen. Reaction of **16** with one equivalent of **8 c** afforded stapled peptide **17 a** in 55 % yield after 4 hours (Table 1, entry 1). Formation of the disulfide bridge was identified as the major side‐product of the reaction. Excess of the reagent resulted in yields up to 73 %, but little or no difference was found beyond 3.0 equivalents (entries 1 to 4). Raising the temperature to 37 °C, increased the yield to 78 % and allowed to better solubilize the stapling reagents (entry 5). At 5 mM, the reaction was completed in 30 minutes albeit with a slight loss of yield (entry 6). The reaction worked also well in DMSO, but the yield decreased to 62 % (entry 7). In other solvents, the solubility of the linear peptide **16** and reagent **8 c** was not sufficient and very low to no conversion was observed (entries 8 to 10).

We then set out to expand the peptide scope using the reagent **8 c** under the optimized conditions (Table 2 A). Based on our previous work with EBX,[^14^, ^15^] we identified arginine (R) as the most likely residue to potentially raise chemoselectivity issues. Hence, we selected the Ac‐YGGEAAREA**C**ARE**C**AARE Cys–Cys (*i,i+*4) stapling model **18** reported by Greenbaum and co‐workers, which contains three arginine (R) residues.^[9a]^ Compared to the model system (Table 2 A, entry 1), a similar result was obtained (entry 2). Considering that the distance between the *i* ^th^ and *i*+4^th^ amino acids in α‐helix is 5.4 Å and the distance between iodine atoms in reagent **8 c** was 8.7 Å (according to X‐ray analysis, Scheme 3 B), we wondered if **8 c** could be used for two‐loop stapling (*i,i+*7, 10.8 Å), taking into account the additional flexibility provided by the cysteine sidechains. Consequently, we synthesized another peptide model **20**, with the sequence: Ac‐QSQQTF**C**NLWRLL**C**QN. This sequence has been introduced by Verdine and co‐workers as a modification of the wild type helical binding domain of p53 that exhibits better cell permeability and helicity.^[22]^ The inhibition of the interaction between p53 and MDM2 proteins has been linked to tumor suppression. They further improved the property of the peptide by stapling in a two‐loop fashion (*i,i+*7) via metathesis, where non‐natural olefinic amino acids were used in place of cysteines. Unfortunately, stapling with **8 c** was less efficient in this case (entry 3). In order to study the chemoselectivity of our reagents, we applied **8 c** on peptide **22** containing the most nucleophilic amino acids; Ser (S), Glu (E), Arg (R), Trp (W), His (H), Gln (Q), Tyr (Y), Lys (K), Asn (N) and Met (M) in no particular order, as well as a free *N* terminus. To our delight, reactivity and selectivity comparable with the ones of other peptide models were observed (entry 4).

**Table 2:** Reagent and peptide scope. The structure of the products was established based on MS/MS experiments (see Supporting Information). Reactions were done on 0.2 to 1.5 μmol scale.

**A**. Cys–Cys stapling.^[a]^														
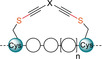	X=	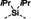			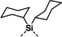			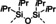			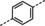			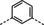
Peptide	Entry	Rel. abs. [%]^[b]^		Entry	Rel. abs. [%]		Entry	Rel. abs. [%]		Entry	Rel. abs. [%]		Entry	Rel. abs. [%]
Ac‐ENPE**C**ILD**C**HVQRVM‐NH_2_ (**16**)	1	98 [46] (**17 a**)		5	89 (**17 b**)		8	51 [29] (**17 c**)		12	27 (**17 d**)		14	13 (**17 e**)
Ac‐YGGEAAREA**C**ARE**C**AARE‐NH_2_ (**18**)	2	72 [48] (**19 a**)		6	69 (**19 b**)		9	9 [19] (**19 c**)		–	–		–	–
Ac‐QSQQTF**C**NLWRLL**C**QN‐NH_2_ (**20**)	3	30 [13] (**21 a**)		7	[22] (**21 b**)		10	44^[c]^ [17] (**21 c**)		13	46 (**21 d**)		15	13 (**21 e**)
H_2_N‐SER**C**WHE**C**YKNM‐NH_2_ (**22**)	4	79 (**23 a**)		–	–		11	42^[c]^ (**23 c**)		–	–		–	–

**B**. Cys–Lys stapling.^[d]^														
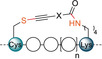	X=	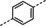				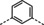				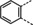				
Peptide	Entry	Rel. abs. [%]			Entry	Rel. abs. [%]			Entry	Rel. abs. [%]				
Ac‐ENPE**C**ILD**K**HVQRVM‐NH_2_ (**24**)	16	94^[c]^ [52] (**25 a**)			21	34 [7] (**25 b**)			26	101^[e]^ (**25 c**)				
Ac‐YGGEAAREA**C**ARE**K**AARE‐NH_2_ (**26**)	17	114 [65] (**27 a**)			22	77 [44] (**27 b**)			27	118^[e]^ (**27 c**)				
Ac‐QSQQTF**C**NLWRLL**K**QN‐NH_2_ (**28**)	18	117 [87] (**29 a**)			23	110 [55] (**29 b**)			28	123^[e]^ (**29 c**)				
H_2_N‐SER**C**WHE**K**YKNM‐NH_2_ (**30**)	19	63 (**31 a**)			24	20 (**31 b**)			–	–				
**H_2_N**‐RSQFY**K**HDAG**C**G‐NH_2_ (**32**)	20	69,^[c]^ 23^[c]^ (**33 a**, **33 a′**)			25	22 (**33 b**)			–	–				

[a] Reaction conditions: **8** (3.0 equiv), DIPEA (2.5 equiv), 1 mM, 37 °C, 4 h. [b] Relative absorbance of stapled product compared to a standard solution of starting material at 210 nm (see Supporting Information, Section 3). The relative absorbance correlates well with the yield, but is higher, the error was estimated to be 5 to 28 % taking in account absorbance of linker (calculated for **17 a** and **29 a**) and errors arising from weighing small amounts of starting material. The absorbance was taken as the average of two reactions (see Supporting Information, Table S5 and S9). The isolated yields obtained on 4.3 mg to 16 mg (1.9 to 7.3 μmol) scale are given in square brackets. [c] 24 hour reaction time. [d] Reaction conditions: **9** (1.1 equiv), DIPEA (2.5 equiv), 1 mM, 37 °C, 30 min. [e] The stapled peptide was unstable and could not be isolated in pure form (see Supporting Information, Section 10).

Next, we turned to the scope of reagents. The cyclohexyl‐based reagent **8 d** afforded the stapled product with peptides **16**, **18** and **20** as efficiently as the *i*Pr‐based reagent **8 c** (entries 5–7). Introduction of the longer silanol‐based linker using reagent **8 e** was generally less efficient (entries 8–11), with the exception of a slightly better result obtained for the *i*,*i+*7 model (entry 10). This is in accordance with the longer distance between the two iodine atoms. Due to the observed low reactivity of **8 e**, the monothioalkynylation intermediate was commonly detected as the major product. Longer reaction times did not increase conversion (Supporting Information, Table S5). The use of the *para‐* and *meta*‐ substituted phenyl reagents, **8 a** and **8 b** was then examined for both the one‐loop model **16** and the two‐loop model **20** (entries 12–15). In general, the phenyl‐ based linkers were less efficient than the corresponding silicon analogues.

Only the *para*‐linker together with two‐loop peptide model **20** provided a comparable result (entry 13).^[23]^ The isolation of complex pure peptides is often difficult and associated with significant loss in yield. We were therefore pleased to see that selected peptides could be isolated in pure form after preparative HPLC in 13–48 % yield (Entries 1–2, 7, 8–10).

In order to test the cysteine‐lysine (CK) stapling reagents **9**, we synthesized the same peptide models as used for Cys–Cys stapling (**16**, **18**, **20** and **22**), but exchanging the second cysteine for a lysine—to give peptides **24**, **26**, **28** and **30** (Table 2 B). In addition, the model **32**, previously used by Buchwald and co‐ workers specifically for Cys–Lys stapling,^[12d]^ albeit with a free N‐terminus was also synthesized. To our delight, the *para*‐reagent **9 a** stapled the peptide models **24**, **26**, and **28** very efficiently in only 30 minutes (entries 16–18). By HPLC, only the stapled products were observed. In this case again, higher absorbance is observed for the stapled products in comparison to the starting materials. To confirm that the relative absorbance was still reasonably correlated with the yield, the reaction was performed on larger scale. The stapled products **25 a**, **27 a** and **29 a** were isolated in 52, 65 and 87 % yields, respectively. Therefore, the stapling with the Cys–Lys system appeared to be more general and efficient than with Cys–Cys. This could be due to the higher reactivity of the hypervalent iodine reagent, or the higher flexibility of the lysine side chain. The nucleophilic peptide **30** with a free N‐terminus was stapled slightly less efficiently (entry 19). Interestingly, only one product was detected by HPLC analysis. MS/MS studies confirmed the formation of the Cys–Lys stapled product. This selectivity however, appears to be sequence dependent. In fact, when the unprotected Buchwald model **32** was examined, both stapled products—Cys–Lys (**33 a**) and Cys‐N‐terminus staple (**33 a′**)—were obtained.

The *meta*‐reagent **9 b** showed lower reactivity with peptide **24**. When previously using reagent **9 a**, full conversion was observed, but *meta*‐reagent **9 b** provided only 63 % conversion of **24** in 30 minutes with a 34 % relative absorbance for the product (entry 21). Longer reaction times did not result in significant increase in conversion. The isolation of pure product **25 b** was also difficult and low yielding. However, good results were obtained with peptides **26** and **28** resulting in 77 % and 110 % relative absorbance, respectively (entries 22 and 23). In this case, the products **27 b** and **29 b** could also be isolated in good yields −44 % and 55 % respectively. Stapling was also less efficient with peptide models **30** and **32** containing free N‐terminus (entries 24 and 25). Only the stapling between Cys–Lys was observed by MS/MS analysis of both models. In contrast, excellent reactivity and full conversion of the starting peptides **24**, **26** and **28** was again observed with the *ortho* reagent **9 c** after 30 minutes (Table 2 B, entries 26–28). However, all attempts to isolate the pure products **25 c**, **27 c** or **29 c** were unsuccessful due to the observed instability of the products in solution.^[24]^ For this reason, the peptide scope was not further investigated, as the instability of the *ortho*‐linker made it not suitable for biological applications. Overall, the trends in reactivity suggest that the position of the electron withdrawing carboxy group on the reagent has a strong influence on the efficiency of stapling.

**Figure 1:**
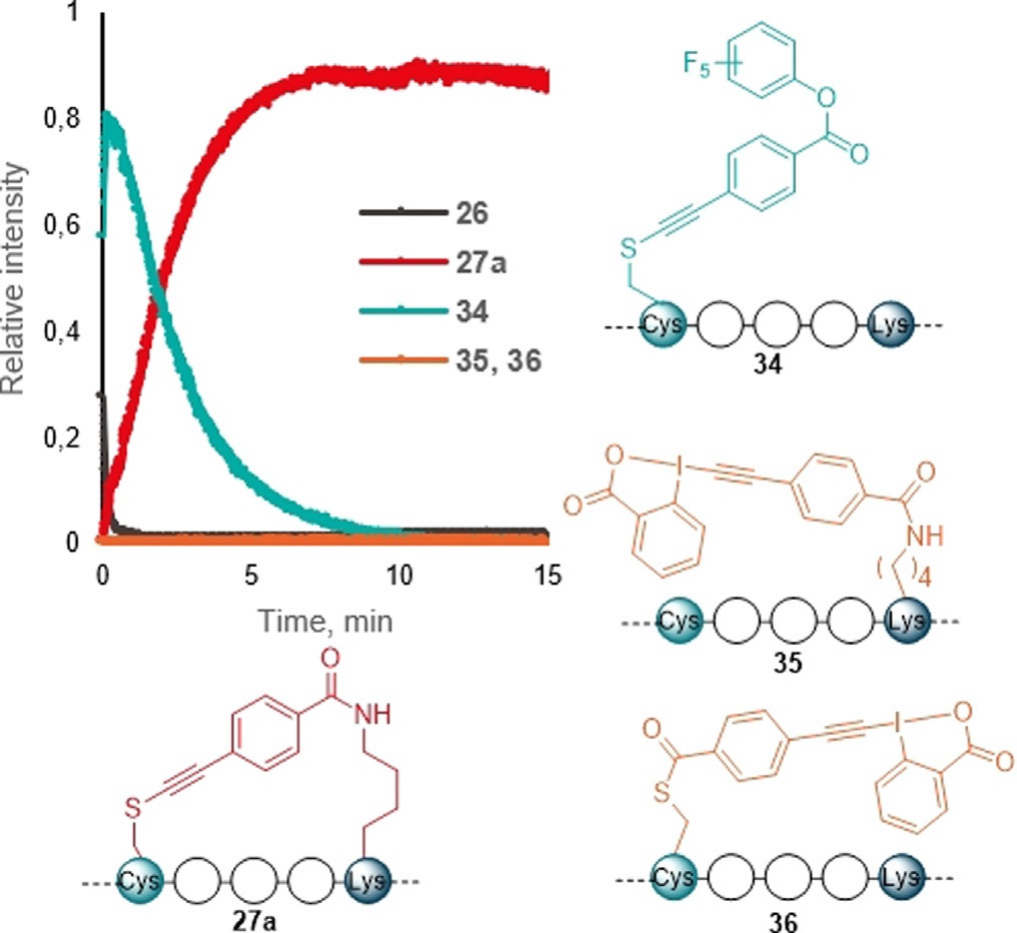
MS kinetic experiment following reaction between peptide **26** and *para* reagent **9 a**. The possible reaction intermediates **34**–**36** are shown. See Supporting Information, Figure S3 for details.

To further investigate the reaction mechanism and the origin of the observed selectivity, stapling of peptide **26** with the *para* reagent **9 a** was chosen as it displayed quantitative conversion, and no other side‐products were detected by HPLC. Kinetic MS experiments showed that the thiol attack onto the EBX core is very fast, complete in under 1 minute, yielding intermediate **34** (Figure 1). The formation of an ynamine intermediate instead is highly improbable, as EBX reagents are known to react rapidly with thiols and not with amines.[^14^, ^15^] Overall, the reaction was finished in 10 minutes even at a 0.1 mM concentration providing the product **27 a**. We were pleased to see that intermediates **35** and **36** arising from initial attack on the activated ester were not detected, suggesting that the Lys attack is proximity driven. If these intermediates were formed, a lack of selectivity would be expected. The observed high selectivity and reaction rate of EBX reagents towards sulfur nucleophile is therefore believed to be the reason behind the high efficiency observed for peptide stapling.

To show that our Cys–Lys stapling can be performed in presence of additional Lys (K) residue, we synthesized a modified version of peptide **26** (Ac‐YGGEAAREACAREKAARE) containing (4,4‐dimethyl‐2,6‐dioxo‐cyclohex‐1‐ylidene)‐3‐methyl‐butyl (ivDde) protected Lys—**ivDde‐26 a** (Table 3, **A**).^[25]^ The stapling using the reagent **9 a** proceeded efficiently and the ivDde protecting group could be removed by addition of hydrazine in one‐pot manner, yielding the final product **26 a′** in excellent rel. abs (Table 3, entry 1). While the reagent **9 b** also reacted well with **ivDde‐26 a**, the deprotection proved to be more difficult as degradation of the peptide was observed (entry 2). Since the protecting group approach lacked generality, we wanted to investigate if unprotected Lys (K) could be tolerated in our stapling method. Based on the results of the kinetic experiment showing that thiol functionalization was orders of magnitude faster (Figure 1), we thought that formation of the staple would be favored over reaction with other Lys (K) not on the same side of the helix. In addition to **26 a**, we introduced a Lys (K) in various positions relative to Cys (Table 3, **B**). After submitting the peptides **26 a**, **26 b** and **26 c** to our Cys–Lys stapling reaction conditions and reagent **9 a**, the desired *i,i+*4 stapled peptides, as confirmed by MS/MS analysis, were obtained in excellent rel. abs (entries 3–5). Only minor formation of another stapled product or overreaction at the free lysine were observed (0–13 % and 0–23 % rel. abs, respectively, see Supporting Information, Section 12b). Moreover, the product **26 a′′**, which could not be obtained using the ivDde protecting group strategy, was now formed in 74 % rel. abs (entry 6).

**Table 3:** Cys–Lys stapling in presence of additional ivDde protected and unprotected Lys. 
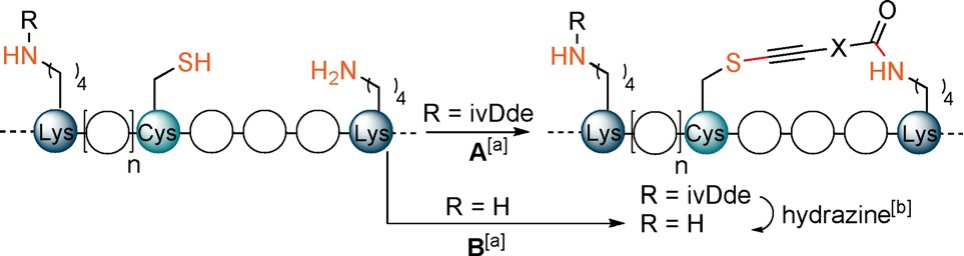

	X=	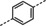		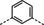
Peptide	Entry	Rel. abs. [%]^[c]^	Entry	Rel. abs. [%]
**A**				
Ac‐YGGEAAR**K(ivDde)**A**C**ARE**K**AARE‐NH_2_	1	117 % (**ivDde‐26 a′**) 97 %^[d]^ (**26 a′**)	2	72 % (**ivDde‐26 a′′**) 0 % (**26 a′′**)
(**ivDde‐26 a**)				
**B**				
Ac‐YGGEAARKA**C**ARE**K**AARE‐NH_2_ (**26 a**)	3	92 % (**26 a′**)	6	74 % (**26 a′′**)
Ac‐YGGEKAREA**C**ARE**K**AARE‐NH_2_ (**26 b**)	4	91 % (**26 b′**)	–	–
Ac‐YGGEAAREA**C**ARE**K**AAREK‐NH_2_ (**26 c**)	5	95 % (**26 c′**)	–	–

[a] Reaction conditions: **9** (1.1 equiv), DIPEA (2.5 equiv), 1 mM, 37 °C, 30 min. [b] Reaction conditions: 35 wt % hydrazine in water, 2 %, 37 °C, 30 min. [c] Relative absorbance of stapled product compared to a standard solution of starting material at 210 nm. [d] Relative absorbance of stapled product compared to a standard solution of **26 a** at 210 nm.

**Scheme 5:**
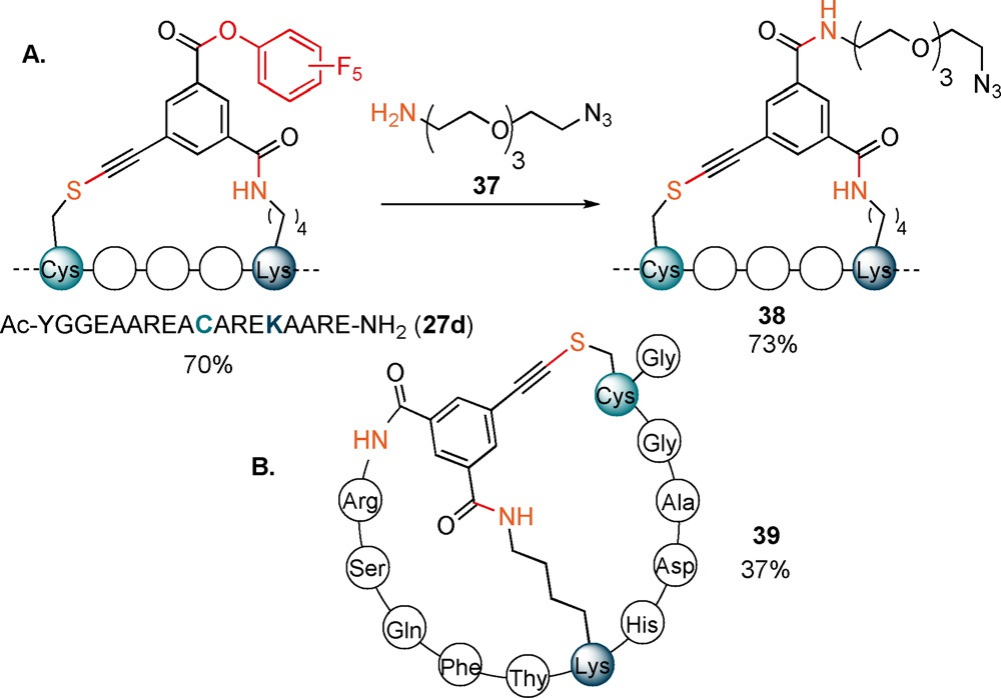
A) Post‐stapling modifications of **27 d**. B) Cys–Lys‐N‐terminus bis‐stapled product **39** obtained from peptide **32**. Relative absorbances of products compared to a standard solution of starting materials are indicated.

We then explored the reactivity of the tris‐functionalized reagent **9 d** in a one‐pot stapling/labelling sequence (Scheme 5 A). The stapled intermediate **27 d** containing a PFP ester was obtained in 70 % relative absorbance using peptide **26**.

The additional activated ester was then further reacted with an amine‐containing azido‐PEG reagent **37**, providing the final functionalized product **38** in 73 % relative absorbance. Next reagent **9 d** was applied to peptide **32** containing a free N‐terminus. After prolonged reaction time, tricyclic peptide **39** was formed in 37 % yield.

With the successful synthesis and isolation of the stapled peptides, we then wanted to explore the use of the unique thioalkyne functionality for post‐stapling modifications. The metal‐catalyzed cycloaddition between azides and terminal alkynes[^26^, ^27^] has been broadly used as a bioorthogonal reaction.^[28]^ In contrast, internal alkynes are usually less reactive. In 2017, Mascareñas and co‐workers reported a rare biocompatible Ru^II^‐catalyzed azide‐thioalkyne cycloaddition (RuAtAC) under aqueous conditions.^[29]^ Thus, we decided to take advantage of this reactivity and apply it to the thioalkyne present in our linkers. In order to apply the method to our stapled peptides, the reported conditions needed to be adjusted. The reported 2:1 ratio of thioalkyne and azide was changed to a 1:1 ratio to ensure the full conversion of the more precious stapled peptide.^[30]^ The concentration of 75 mM was unrealistic for our small reaction scale typically used for peptides and was decreased to 27 mM to ensure solubility. Moreover, the solvent was changed from water or DCM to DMF, to ensure that a one‐pot—stapling and cycloaddition—procedure could be done in the same solvent. After optimization using model stapled peptide **29 a** in combination with 1 equivalent of benzyl azide (**40 a**) (Supporting Information, Table S10), full conversion to yield two regioisomers of the triazole product in a ratio of 10:1 was achieved in presence of 20 mol % of Ru catalyst at 27 mM concentration (Table 4, entry 1). Azides bearing fluorescent dyes (5/6‐TAMRA‐PEG_3_‐N_3_) (**40 b**) and 6‐FAM‐N_3_ (**40 c**)) were then used. In both cases full conversion was observed (entries 2 and 3). The effect of varying the linkers was explored next. Lower conversion, but higher regioselectivity was obtained when using staple **29 b** bearing a *meta* linker. (entry 4). The optimized conditions were then applied to the *para* linked peptides **27 a** and **25 a** using benzyl azide (**40 a**). Excellent reactivity and selectivity were observed with staple **27 a** (entry 5). The desired product was also successfully obtained using staple **25 a**, but a lower conversion was observed (entry 6), possibly due to a more hindered reaction site, created by leucine (L) and isoleucine (I) amino acid residues. The cycloaddition was not successful on the Cys–Cys staple **17 a**, probably due to the steric hindrance resulting from the *i*Pr groups (Supporting Information, Section 14b).

**Table 4:** Azide and stapled peptide scope for RuAtAC. 
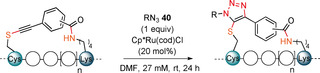

Entry	Sequence	Staple	R=	absorb. ratio [%]^[a]^	
1	Ac‐QSQQTFCNLWRLLKQN‐NH_2_	**29 a**	Bn (**40 a**)	quant.	(10:1)
					(**41 a**, **41 a′**)
2			5/6‐TAMRA‐PEG_3_ (**40 b**)	quant.	(**41 b**, **41 b′**)
3			6‐FAM (**40 c**)	quant.	(**41 c**)
4	Ac‐QSQQTFCNLWRLLKQN‐NH_2_	**29 b**	Bn (**40 a**)	71	(36:1)
					(**42**, **42′**)
5	Ac‐YGGEAAREACAREKAARE‐NH_2_	**27 a**	Bn (**40 a**)	quant.	(32:1)
					(**43**, **43′**)
6	Ac‐ENPECILDKHVQRVN‐NH_2_	**25 a**	Bn (**40 a**)	59	(**44**)
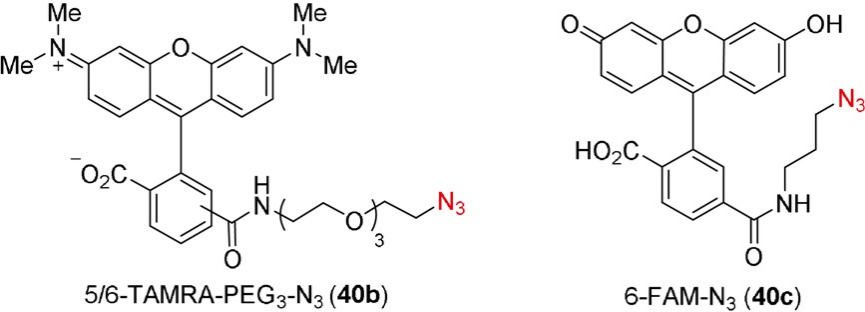					

[a] Absorbance ratio(%)=[(UV absorbance of product)/((combined UV absorbance of stapled peptide and product)] * 100, and is given as combined yield when both regioisomers where observed. Only starting material, reagent and products were observed by HPLC. In brackets ratio of regioisomers is indicated where applicable. Products **41 b** and **41 b′** could not be fully separated, while only one peak corresponding to the *m*/*z* of the desired product was detected for **41 c** and **44** by HPLC analysis. Only the structure of the likely major product is drawn.

**Scheme 6:**
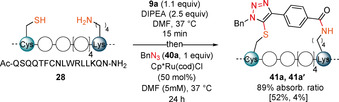
One‐pot stapling/ RuAtAC procedure. Only the structure of the likely major product is presented. Absorbance ratio(%)=[(UV absorbance of product)/((combined UV absorbance of stapled peptide and product)] * 100. Full conversion of peptide **28** was observed. Isolated yield is given in bracket.

The optimized reaction conditions were adjusted to enable one‐pot stapling and subsequent RuAtAC (Scheme 6). The concentration of the reaction was further reduced to 5 mM to ensure intramolecular attack during the stapling step. To reach full conversion, the ruthenium catalyst loading in the cycloaddition step needed to be increased to 50 mol %. The linear peptide **28** could then be converted into triazole products **41 a** and **41 a′** in one‐pot and 89 % conversion of the stapling intermediate. The one‐pot procedure was then used to isolate both regioisomers in 52 % and 4 % yield.

**Figure 2:**
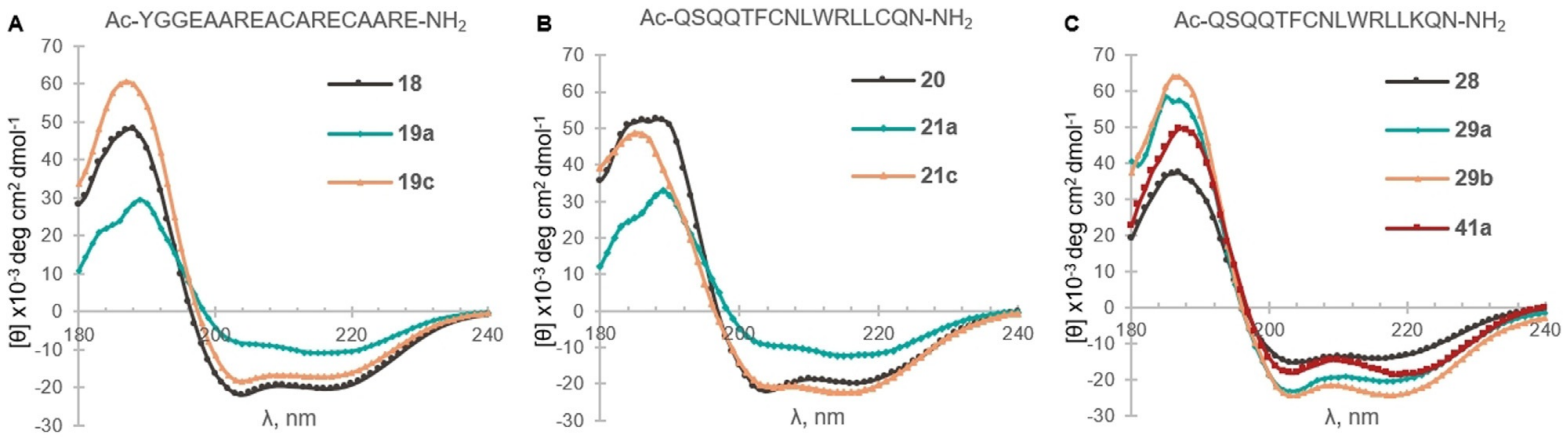
Circular Dichroism (180–240 nm) spectra of linear (represented by a black line) and stapled peptides. Measured using 0.1 mM 40 % TFE/Water solutions. See Figure S3–6 in Supporting Information for the data of other stapled peptides.

To study the changes induced in the peptide conformations, we obtained circular dichroism measurements of the helical linear peptides and the corresponding isolated stapled peptides (Table 2, entries 1–3, 7–10, 16–18, 21–23). Both one‐ and two‐loop staples **19 a** and **21 a** with the bis(isopropyl)silyl tether seemed to have lost partially the alpha helix conformation (Figure 2 A,B). On the other hand, the staples **19 c** and **21 c** with the longer silanol linker showed similar helicity compared to linear peptide **18**, as seen by the values at 222 nm (Table S11).^[31]^ A similar behavior was observed for peptide **17 c**, while for **17 a** obtaining a precise measurement under the same conditions was difficult (See Supporting Information, Section 16, Figure S4 and S5). For Cys–Lys staples in general, little change or decrease in helicity was observed for one‐loop staples **25** and **27** (see Supporting Information, Figure S6 and S7). In contrast, *meta*‐ and *para*‐ two‐loop staples **29 a** and **29 b** as well as the corresponding triazole staple **41 a** displayed higher helicity than linear peptide **28** (Figure 2 C). Overall, the greatest helicity increase was obtained for **29 b** with a *meta* linker for Cys–Lys stapling of the two‐loop model **28**.

**Scheme 7:**
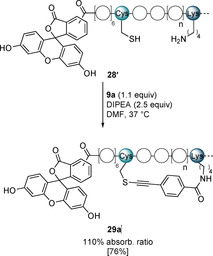
Stapling reaction using fluorescein labelled linear peptide **28′** and reagent **9 a**. Isolated yield is given in bracket.

Finally, we tested if modification of peptides with the novel stapling reagents can yield efficient inhibitors of PPIs. For this we studied the interaction of the p53‐derived peptide sequence Ac‐QSQQTF**C**NLWRLL**C**/**K**QN (**20** and **28**) with MDM2. We first tested the binding of the peptides to MDM2 using a fluorescence polarization (FP) competition assay in which the displacement of a linear, fluorescein‐labeled reporter peptide was measured (fluorescein‐GSGSSQETFSDLWKLLPEN‐NH_2_). The stapled peptides **21 a**, **29 a** and **29 b** efficiently displaced the probe at concentrations close to that of MDM2, suggesting high binding affinities and *K*_d_s in the nanomolar range (Supporting Information, Figure S8). We decide to further investigate the binding of the staple **29 a** as it exhibited good helicity (Figure 2), binding affinity in competition assay and could be isolated in high yield (Table 2). In order to accurately determine the binding affinities we synthesized **28′**, and the corresponding stapled peptide **29 a′**, both as conjugates with a 5(6)‐carboxyfluorescein at the N‐terminus (Scheme 7).

**Figure 3:**
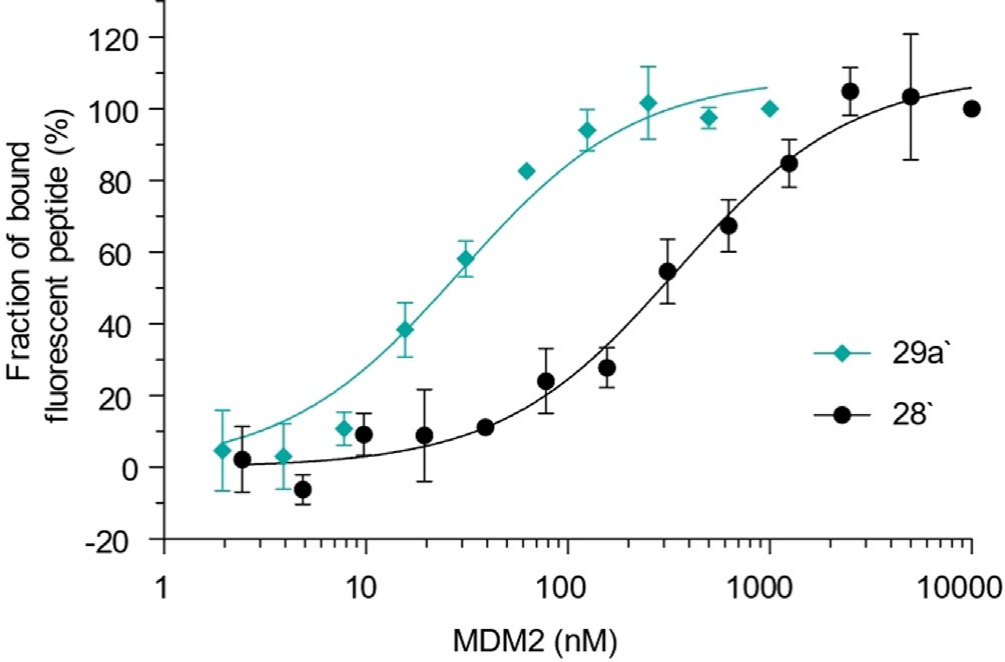
Binding of fluorescein‐labelled stapled peptide **29 a′** and a linear peptide **28′** with the same sequence to MDM2, measured in a fluorescence polarization‐based assay. Average values and SDs of three independent measurements are shown.

The presence of fluorescein did not affect the stapling reaction, and product **29 a′** was isolated in 76 % yield and could be used to measure the binding to MDM2 in a direct FP assay. The stapled peptide bound to MDM2 with a 12‐fold higher affinity (*K*_d_=29±4 nM) than the linear one (*K*_d_=346±45 nM) (Figure 3). Interestingly, both the absolute value and the increase of affinity are higher than for the reported metathesis staple (*K*_d_=55±10 nM and 2‐fold increase).^[22]^ It is comparable to affinities observed for other reported highly optimized metathesis‐based peptide staples.^[32]^ In addition, a single pure staple is obtained in contrast to the *E*/*Z* isomers formed in metathesis reactions, which display different affinities. This result showed that the developed stapling reagents can substantially improve the binding affinity of α‐helical peptides and yield high‐affinity inhibitors of PPIs.

## Conclusion

In summary, we have reported the synthesis of new bifunctional hypervalent iodine reagents and their use for peptide stapling. Reagents bearing two reactive hypervalent iodine sites were developed for Cys–Cys stapling, whereas combining one hypervalent iodine group with an activated ester enabled Cys–Lys stapling. Both one‐loop (*i,i+*4) and two‐loop (*i,i+*7) stapling was possible. The stapling did not require the use of protecting or activating groups. The geometry and length of the linker in the reagents greatly influenced the stapling efficiency and helicity. Post‐modification of the stapled peptides was achieved either by introducing an additional reactive ester group, or by ruthenium‐catalyzed cycloaddition of the formed thioalkynes with azides. Depending on the linker length and geometry, either an increase or decrease of helicity was observed. Stapled peptide **29 a** displayed both enhanced helicity and binding affinity to the MDM2 protein.

## Conflict of interest

The authors declare no conflict of interest.

## Supporting information

As a service to our authors and readers, this journal provides supporting information supplied by the authors. Such materials are peer reviewed and may be re‐organized for online delivery, but are not copy‐edited or typeset. Technical support issues arising from supporting information (other than missing files) should be addressed to the authors.

Supplementary
