# Supplementary material for: Cys–Cys and Cys–Lys Stapling of Unprotected Peptides Enabled by Hypervalent Iodine Reagents
Source: Angew Chem Int Ed Engl. 2021 Mar 8;60(16):9022–31. doi: 10.1002/anie.202014511 (PMC8048981; doi:10.1002/anie.202014511)

Angewandte Chemie
Eine Zeitschrift der Gesellschaft Deutscher Chemiker

Supporting Information

Cys–Cys and Cys–Lys Stapling of Unprotected Peptides Enabled by Hypervalent Iodine Reagents

*Javier Ceballos[+], Elija Grinhagena[+], Gontran Sangouard, Christian Heinis, and Jerome Waser**

anie_202014511_sm_miscellaneous_information.pdf

Supporting Information

Table of Contents

1. General procedures 3
2. HPLC-MS and preparative HPLC information 3
   a. HPLC-MS analysis 3
   b. Preparative HPLC 4
3. Synthesis and Characterization of peptides 4
   a. Synthesis of Cysteine-Cysteine containing peptides 6
   b. Synthesis of Cysteine-Lysine containing peptides 8
4. Synthesis of the stapling reagents 14
   a. Cysteine-Cysteine stapling reagents 14
   b. Cysteine-Lysine stapling reagents 22
5. Small molecule model 30
6. Crystal structure 31
7. Peptide model Calibration 31
8. Cysteine-cysteine reaction optimization 35
   a. Base optimization 35
   b. Base loading optimization 37
   c. Reagent loading 39
   d. Temperature 41
   e. Concentration 41
   f. Solvent 42
9. Cysteine-Cysteine Stapling Scope 45
10. Cysteine-Lysine Stapling Scope 60
11. Kinetic Data for the reaction of the Cysteine-Lysine system 72
12. Cysteine-Lysine Stapling in presence of additional Lys 73
13. Stapling using the reagent 9d containing additional activated ester 77
14. RuAtAC procedure 81
   a. Optimization 81
   b. Azide and stapled peptide scope 84
15. One-pot RuAtAC procedure 90
   a. Optimization 90
   b. Isolation of the products using optimized one-pot procedure 91
16. Circular Dichroism Measurements 93
17. Synthesis of Fluoresceine Labelled Stapled Peptides 96
18. Binding assays 97

19. NMR spectra of the synthesized compounds...99

1. General procedures

All reactions using anhydrous conditions were performed with oven-dried glassware, under an atmosphere of nitrogen, unless stated otherwise. Tetrahydrofuran, acetonitrile, diethyl ether and dichloromethane (DCM) were dried by passage over activated alumina, under nitrogen atmosphere, on an Innovative Technology Solvent Delivery System (water content < 10 ppm, Karl-Fischer titration). Dichloroethane and ethanol were purchased from Acros and trifluoroethanol was purchased from Fluorochem. DMSO was purchased from Sigma-Aldrich. All the Fmoc-protected amino acids and Rink Amide MBHA resin were purchased from GL Biochem or Bachem. 1-[Bis(dimethylamino)methylene]-1H-1,2,3-triazolo[4,5-b]pyridinium 3-oxide hexafluorophosphate (HATU, Bachem) and N,N-diisopropylethylamine (DIPEA, Iris Biotech GmbH) were used as received. All the other reagents were purchased from ABCR, Acros, AlfaAesar, Apollo Scientific, Fluorochem, Fluka, Roth, Sigma-Aldrich and TCI and were used as such. For flash chromatography, distilled technical grade solvents were used. Chromatographic purification was performed as flash chromatography using Macherey-Nagel silica 40-63, 60 Å, using the solvents indicated as eluent with 0.1 – 0.5 bar pressure. TLC was performed on Merck silica gel 60 F254 TLC aluminum or glass plates and visualized with UV light or permanganate stain. Melting points were measured on a Büchi B-540 melting point apparatus using open glass capillaries. 1H-NMR spectra were recorded on a Brucker DPX-400 400 MHz spectrometer in $CDCl_3$, DMSO-d6 or D_2O. All signals are reported in ppm with the internal $CHCl_3$ signal at 7.26 ppm, the internal DMSO signal at 2.50 ppm and MeOD as 3.31 ppm as standard. The data is being reported as: s = singlet, d = doublet, t = triplet, q = quadruplet, qi = quintet, m = multiplet or unresolved, br = broad signal, app = apparent, coupling constant(s) in Hz, integration, interpretation.13C-NMR spectra were recorded with 1H-decoupling on a Brucker DPX-400 100 MHz spectrometer in $CDCl_3$, DMSO-d6 or MeOD. All signals are reported in ppm with the internal $CHCl_3$ signal at 77.16 ppm or the internal DMSO signal at 39.52 ppm as standard. Spectra were fully assigned using COSY, HSQC, HMBC and ROESY. Infrared spectra were recorded on a JASCO FT-IR B4100 spectrophotometer with an ATR PRO410-S and a ZnSe prisma and are reported as cm^{-1} (w = weak, m = medium, s = strong, br = broad). High-resolution mass spectrometric measurements were performed by the mass spectrometry service of ISIC at the EPFL on LTQ Orbitrap ELITE ETD (Thermo fisher), Xevo G2-S QTOF (Waters), or LTQ Orbitrap ELITE ETD (Thermo fisher). Circular dichroism measurements were performed using a 100 µM solution of peptide in a mixture of water and 2,2,2-trifluoroethanol (TFE), Spectra were measured from 260 nM to 190 nM on a J-810 Spectropolarimeter (Jasco, Oklahoma City, USA).

2. HPLC-MS and preparative HPLC information

a. HPLC-MS analysis

HPLC-MS measurements were performed on an Agilent 1290 Infinity HPLC system with a G4226a 1290 Autosampler, a G4220A 1290 Bin Pump and a G4212A 1290 DAD detector, connected to a 6130 Quadrupole LC/MS, coupled with a Waters XBridge C18 column (250 x 4.6 mm, 5 µm). Water:acetonitrile 95:5 (solvent A) and water:acetonitrile 5:95 (solvent B), each containing 0.1% formic acid, were used as the mobile phase, at a flow rate of 0.6 $mL.min^{-1}$. The gradient was programmed as follows:

Method 1: 100% A for 5 minutes and then a gradient to 100% B in 20 minutes, plus 5 minutes of 100% B.

Method 2: Gradient from 100% A to a 100% B in 20 minutes, plus 5 minutes of 100% B.

Method 1 was used for HPLC-MS analysis unless noted otherwise.

The column temperature was set up to 25 °C. Low-resolution mass spectrometric measurements were acquired using the following parameters: positive electrospray ionization (ESI), temperature of drying gas = 350 °C, flow rate of drying gas = 12 L. min^{-1}, pressure of nebulizer gas = 60 psi, capillary voltage = 2500 V and fragmentor voltage = 70 V.

b. Preparative HPLC

Preparative RP-HPLC were performed on an Agilent 1260 HPLC system with a G2260A 1260 Prep ALS Autosampler, a G1361a 1260 Prep Pump, a G1365C 1260 MWD detector and a G1364B 1260 FC-PS collector, coupled with a Waters XBridge semi-preparative C18 column (19 x 150 mm, 5 µm). Water (solvent A) and water:acetonitrile 5:95 (solvent B), each containing 0.1% TFA, were used as the mobile phase at a flow rate of 20 $mL.min^{-1}$. The gradient was programmed as follows: 100% A isocratic for 5 minutes followed by 100% A to 100% B in 20 minutes then isocratic for 5 minutes.

3. Synthesis and Characterization of peptides

Solid-Phase Peptide Synthesis (SPPS):

Peptides were synthesized on an MultiPep RSi parallel peptide synthesizer (Intavis) using standard Fmoc SPPS-chemistry and Rink Amide MBHA resin (0.337 mmol/g resin, 0.05 mmol scale). Each coupling cycle was initiated by Fmoc deprotection on the Rink Amide MBHA resin, achieved by shaking the resin with 800 µL of 20% v/v piperidine in dimethylformamide (DMF) at 400 rpm, over 5 minutes twice. Then the resin was washed with DMF (6000 µL x7). The coupling was carried out by shaking Rink Amide MBHA resin with a Fmoc-protected monomer (4.0 equiv.), 2-(1H-benzotriazol-1-yl)-1,1,3,3-tetramethyluronium hexafluorophosphate (HBTU, 4.0 equiv.), hydroxybenzotriazole (HOBt, 4.0 equiv.) and *N*-Methylmorpholine (6.0 equiv.), in DMF (1.3 mL), at 400 rpm, over 30 minutes twice. Alternatively, HATU (4.0 equiv.) instead of the HBTU and HOBt combination was used for the coupling. Capping using Cap Mixture (5% v/v Ac_2O and 6% v/v 2,6-lutidine in DMF) was carried out at the end of each cycle, followed by a DMF wash (6000 µL x7). The synthesis was finished by deprotection of Fmoc using 20% v/v piperidine in dimethylformamide at 400 rpm, over 5 minutes two times. The N-terminus was either left unprotected or was acylated or fluoresceinated. Acetylation of the N-terminal was achieved by incubating the resin with Cap Mixture three times. Fluoresceination was achieved by shaking Rink Amide MBHA resin with a 5(6)-carboxyfluorescein (2.0 equiv.), 2-(1H-benzotriazol-1-yl)-1,1,3,3-tetramethyluronium hexafluorophosphate (HATU, 2.0 equiv.) and *N*-Methylmorpholine (3.0 equiv.), in DMF (1.3 mL), at 400 rpm, over 30 minutes. Next, washing steps were performed with dimethylformamide (5 x 3 mL). Finally, resin was dried with dichloromethane (5 x 3 mL).

Peptide cleavage and deprotection:

Peptides were deprotected and cleaved from the resin by treatment with 2.5% v/v water and 2.5% v/v Triisopropyl silane in neat trifluoroacetic acid (5 mL). The resulting mixture was shaken for 2 hours, at room temperature. The resin was removed by filtration and peptides were precipitated

in cold diethyl ether (50 mL), followed by a 2 hours incubation at -20 °C. Peptides were pelleted by centrifugation at 4000 rpm, for 5 minutes. Finally, the mother liquors were carefully removed.

Peptide purification and analysis:

Peptides were dissolved in water with a minimum amount of organic co-solvent (acetonitrile, dimethylformamide or dimethyl sulfoxide). Peptides were then purified on preparative RP-HPLC with a gradient of 5 minutes of solvent A (100% Water with 0.1% TFA) and then 20 minutes gradient to 100% solvent B (5% Water/AcCN with 0.1% TFA). Fractions containing the desired peptide were lyophilized. Peptides were obtained as TFA salts, one molecule of TFA was assumed for every basic amino acid residue - Lys (K), Arg (R) and His (H). The purity was assessed by HPLC-MS analysis. At the same time, low-resolution mass spectrometric measurements were also acquired. In order to obtain high-resolution mass spectrometric measurements, the purified peptide was submitted to the mass spectrometry service of ISIC at the EPFL that uses a MICROMASS (ESI) Q-TOF Ultima API.

MS/MS fragmentation

The regioselectivity of the stapling was confirmed using MS/MS analysis. The spectra were obtained by the mass spectrometry service of ISIC at the EPFL using Thermo Orbitrap Elite instrument. The desired ion was selected using mass filters and submitted to fragmentations. The obtained data was analyzed using fragment generation program on eln.epfl.ch.[1] For the calculations peak threshold for intensity was set to 0.5% and 0.03% for quantity, precision was set to 5 ppm and minimal similarity: 70%. The peaks were compared to theoretical peaks. The theoretical peak width was calculated from the mass of the ion by the formula provided in the script. The zone was set to -0.5 to 4.5 ppm. y and b fragments with and without linker were selected and reported. In the cases were fragmentation was low, c and z fragments and/or fragments arising from neutral losses were included. The fragments are reported as: **fragment type** (fragment intensity), the fragments containing the linker mass are indicated by *italics*. The unexpected fragments are noted by *. The unexpected fragments exhibit small intensity and could arise due to background noise, small amount of impurities or if small amount of the staple was broken during the fragmentation experiment. The MS/MS experiments on Cys-Cys systems are of less importance as the Cys-EBX reactivity has been well established by our group among others (see manuscript). Hence, only selected MS/MS analysis were performed on Cys-Cys stapled peptides.

[1] a) J. S. Desport, G. Frache, L. Patiny *Ref Rapid Commun Mass Spectrom.* **2020**, *e8652*; b) D. Ortiz, N Gasilova, F. Sepulveda, L. Patiny, P. J. Dyson, L. Menin, *Ref Rapid Commun Mass Spectrom.* **2020.**

a. Synthesis of Cysteine-Cysteine containing peptides

AcetGluAsnProGluCysIleLeuAspCysHisValGlnArgValMetNH$_2$ (16)

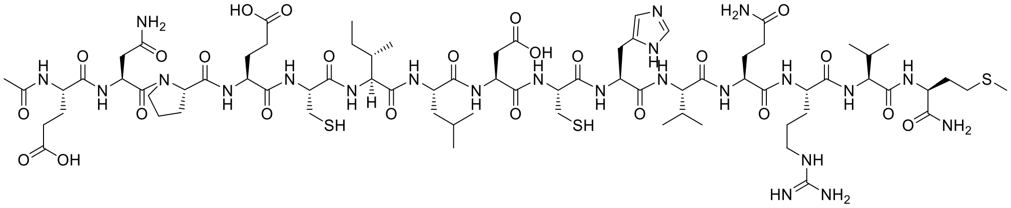

Following the general procedure, Ac-ENPECILDCHVQRVM-NH$_2$ (**16**) was obtained as white amorphous solid.

HRMS (nanochip-ESI/LTQ-Orbitrap) m/z: $[M + H]^+$ Calcd for $C_{75}H_{124}N_{23}O_{24}S_3^+$ 1826.8346; Found 1826.8376.HPLC-UV Chromatogram (210 nm):

Retention time: 13.2 min

Absorbance area (mAU): 3128.8

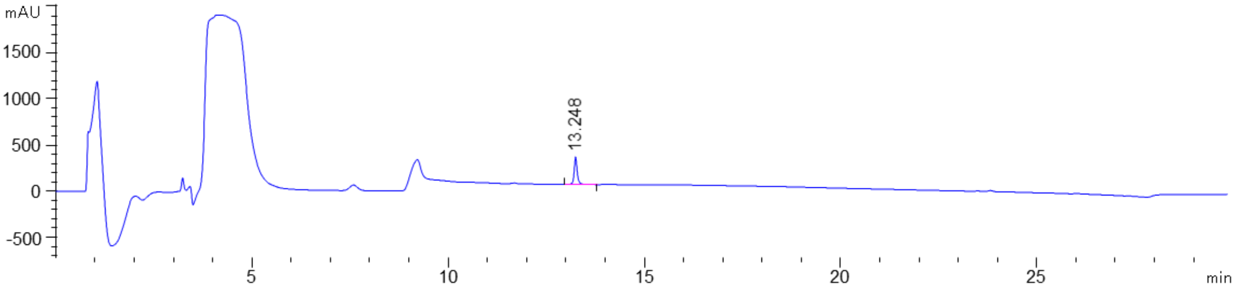

AcetTyrGlyGlyGluAlaAlaArgGluAlaCysAlaArgGluCysAlaAlaArgGluNH$_2$ (18)

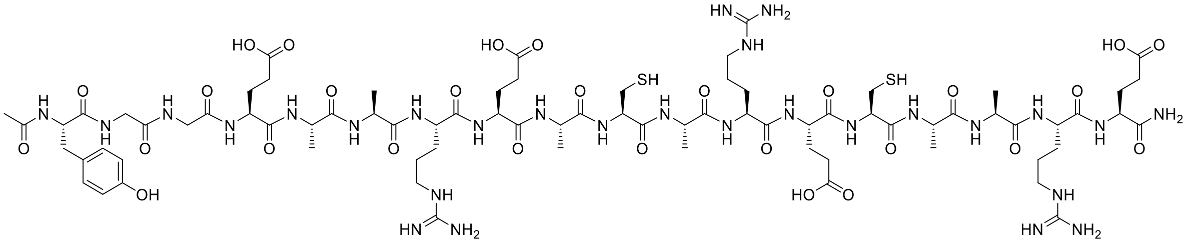

Following the general procedure, Ac-YGGEAAREACARECAARE-NH$_2$ (**18**) was obtained as white amorphous solid.

HRMS (ESI/QTOF) m/z: $[M + H_2]^{+2}$ Calcd for $C_{77}H_{126}N_{28}O_{28}S_2^{+2}$ 977.4363; Found 977.4379

HPLC-UV Chromatogram (210 nm) of a 1 mM solution in DMF:

Retention time: 11.1 min

Absorbance area (mAU): 3676

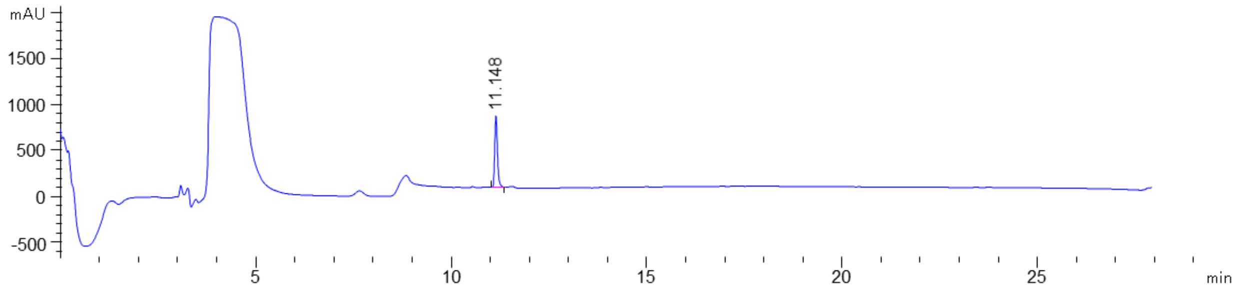

AcetGlnSerGlnGlnThrPheCysAsnLeuTrpArgLeuLeuLysGlnAsnNH$_2$ (20)

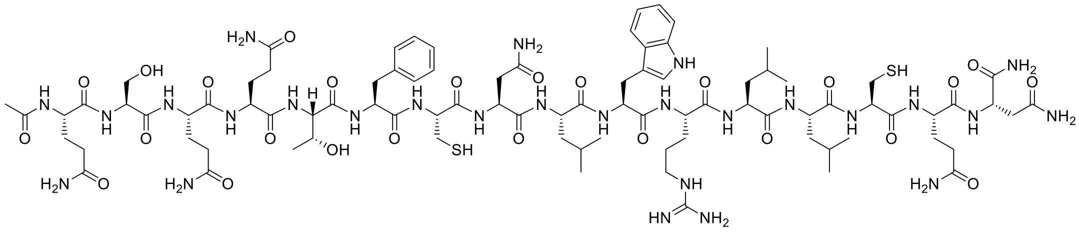

Following the general procedure, Ac-QSQQTFCNLWRLLCQN-NH$_2$ (**20**) was obtained as white amorphous solid.

HRMS (ESI/QTOF) m/z: $[M+H]^+$ Calcd for $C_{87}H_{136}N_{27}O_{25}S_2^+$ 2022.9637; Found 2022.9679

HPLC-UV Chromatogram (210 nm) of a 1 mM solution in DMF:

Retention time: 15.8 min

Absorbance area (mAU): 3916

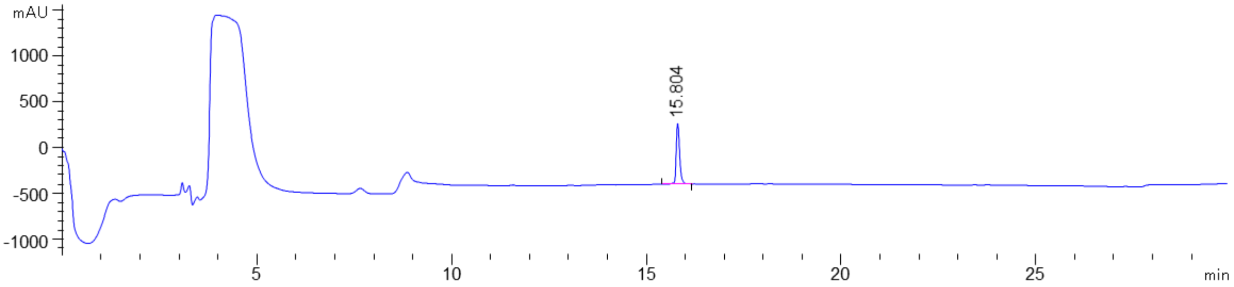

HSerGluArgCysTrpHisGluCysTyrLysAsnMetNH$_2$ (22)

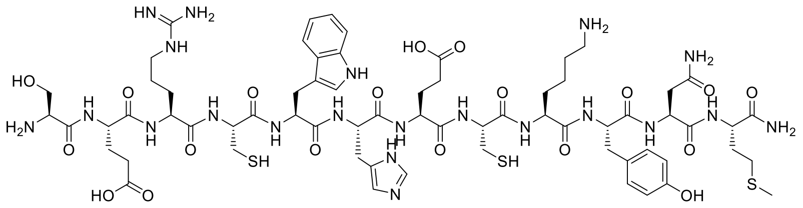

Following the general procedure, H-SERCWHECYKNM-NH_2 (**22**) was obtained as white amorphous solid.

HRMS (APPI/LTQ-Orbitrap) m/z: $[M + H_2]^{+2}$ Calcd for $C_{66}H_{99}N_{21}O_{19}S_3^{+2}$ 792.8289; Found 792.8251.

HPLC-UV Chromatogram (210 nm) of a 1 mM solution in DMF:

Retention time: 10.9 min

Absorbance area (mAU): 3515

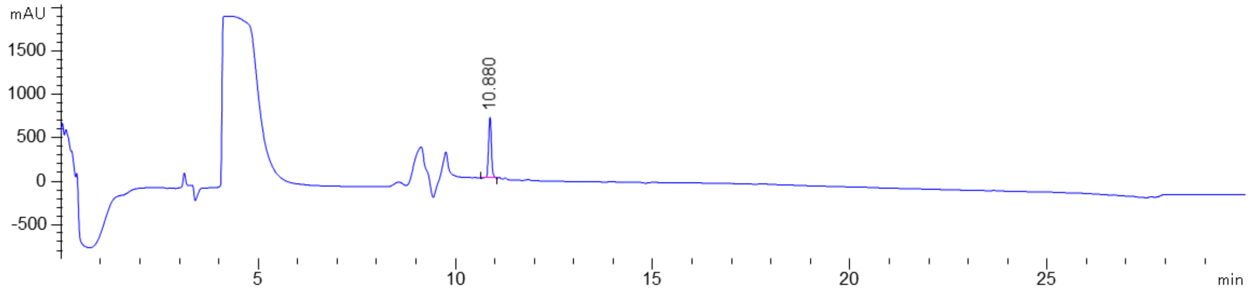

b. Synthesis of Cysteine-Lysine containing peptides

AcetGluAsnProGluCysIleLeuAspLysHisValGlnArgValMetNH₂ (24)

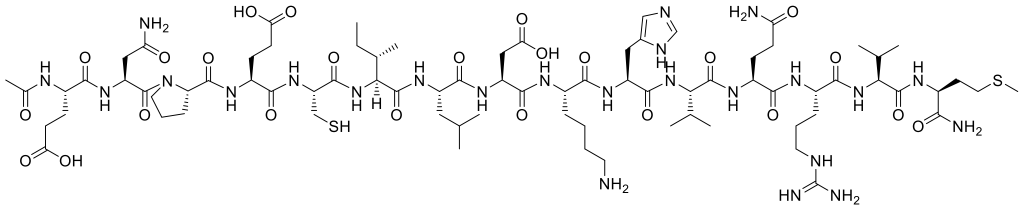

Following the general procedure, Ac-ENPECILDKHVQRVM-NH_2 (**24**) was obtained as white amorphous solid.

HRMS (nanochip-ESI/LTQ-Orbitrap) m/z: $[M + H_2]^{+2}$ Calcd for $C_{78}H_{132}N_{24}O_{24}S_2^{+2}$ 926.4638; Found 926.4629.

HPLC-UV Chromatogram (210 nm) of a 1 mM solution in DMF:

Retention time: 12.4 min

Absorbance area (mAU): 3127

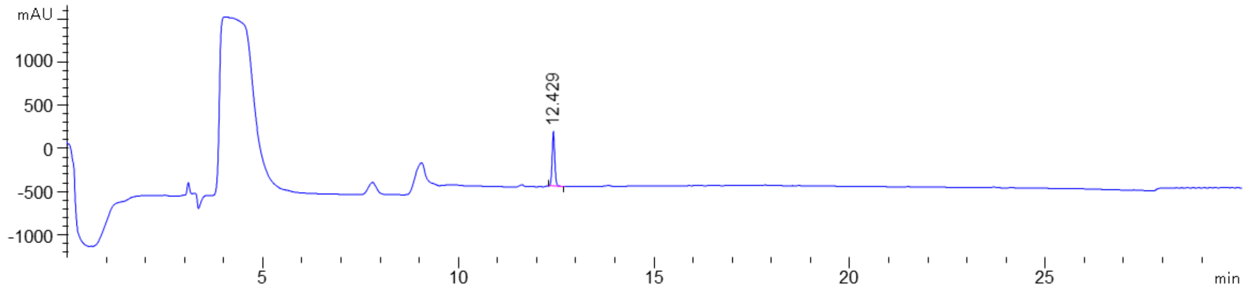

AcetTyrGlyGlyGluAlaAlaArgGluAlaCysAlaArgGluLysAlaAlaArgGluNH$_2$ (26)

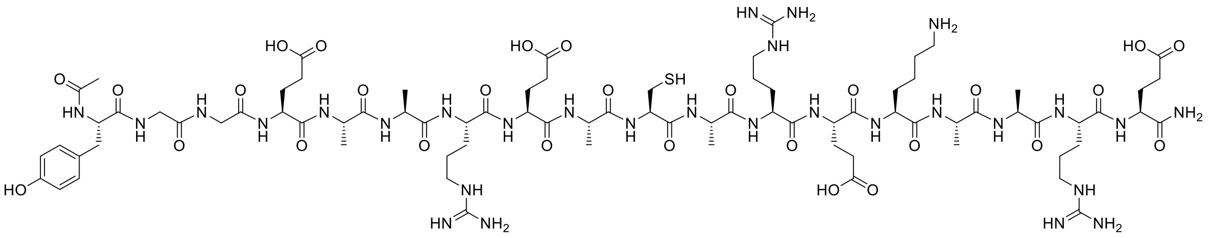

Following the general procedure, Ac-YGGEAAREACAREKAARE-NH$_2$ (**26**) was obtained as white amorphous solid.

HRMS (nanochip-ESI/LTQ-Orbitrap) m/z: $[M + H_2]^{+2}$ Calcd for $C_{80}H_{133}N_{29}O_{28}S^{+2}$ 989.9792; Found 989.9789.

HPLC-UV Chromatogram (210 nm) of a 1 mM solution in DMF:

Retention time: 10.5 min

Absorbance area (mAU): 2414

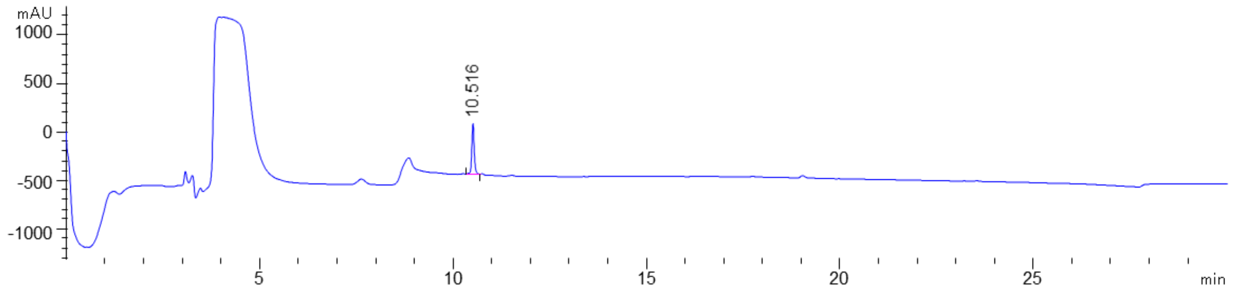

AcetGlnSerGlnGlnThrPheCysAsnLeuTrpArgLeuLeuLysGlnAsnNH$_2$ (28)

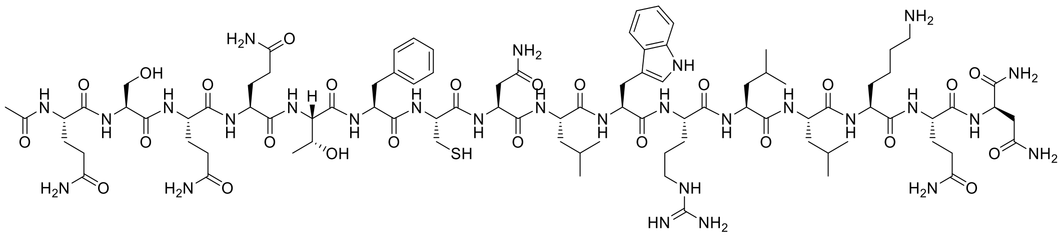

Following the general procedure, Ac-QSQQTFCNLWRLLKQN-NH_2 (**28**) was obtained as white amorphous solid.

HRMS (nanochip-ESI/LTQ-Orbitrap) m/z: [M+ H_2]$^{+2}$ Calcd for $C_{90}H_{144}N_{28}O_{25}S^{+2}$ 1024.5284; Found 1024.5329.

HPLC-UV Chromatogram (210 nm) of a 1 mM solution in DMF:

Retention time: 14.4 min

Absorbance area (mAU): 3134

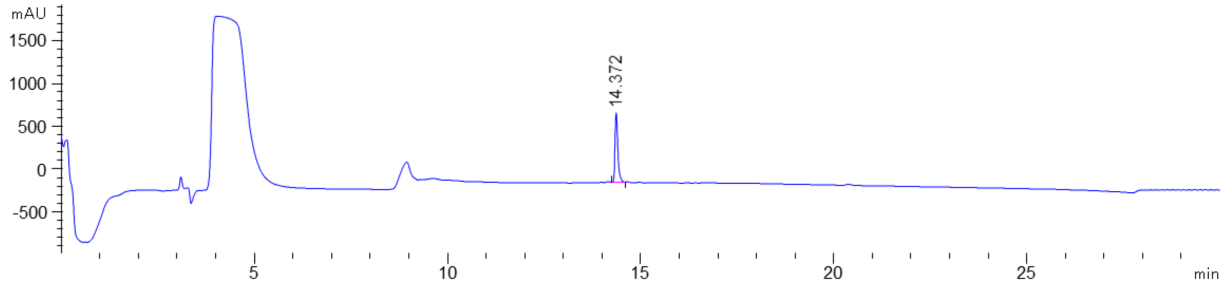

5(6)-FAM-GlnSerGlnGlnThrPheCysAsnLeuTrpArgLeuLeuLysGlnAsnNH$_2$ (28')

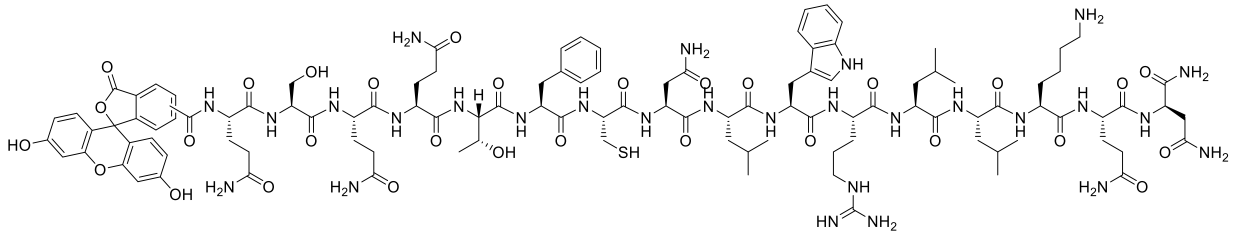

Following the general procedure, 5(6)-FAM-QSQQTFCNLWRLLKQN-NH_2 (**28'**) was obtained as yellow amorphous solid.

HRMS (ESI/QTOF) m/z: [M]+ Calcd for $C_{109}H_{152}N_{28}O_{30}S^{+2}$ 1182.5469; Found 1182.5482.

HPLC-UV Chromatogram (210 nm) of a 1 mM solution in DMF:

Retention time: 14.9 min

Absorbance area (mAU): 7129

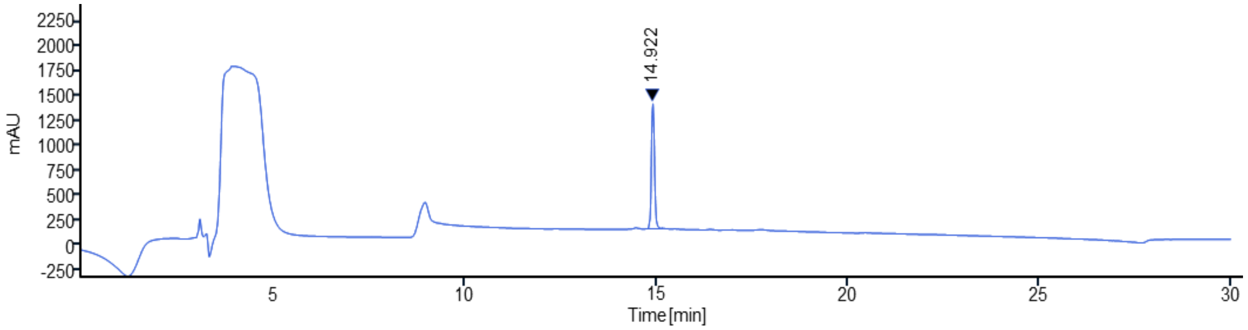

HSerGluArgCysTrpHisGluLysTyrAsnMetNH₂ (30)

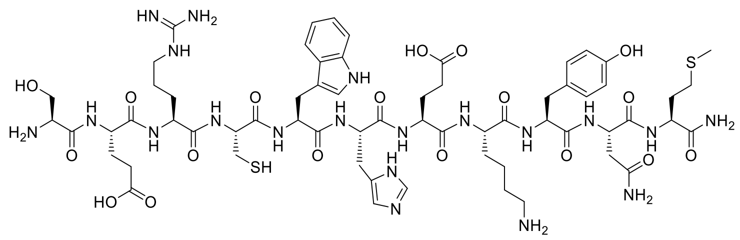

Following the general procedure, H-SERCWHEKYNM-NH_2 (**30**) was obtained as white amorphous solid.

HRMS (nanochip-ESI/LTQ-Orbitrap) m/z: $[M + H_3]^{+3}$ Calcd for $C_{63}H_{95}N_{20}O_{18}S_2^{+3}$ 494.5519; Found 494.5497

HPLC-UV Chromatogram (210 nm) of a 1 mM solution in DMF:

Retention time: 10.6 min

Absorbance area (mAU): 3417

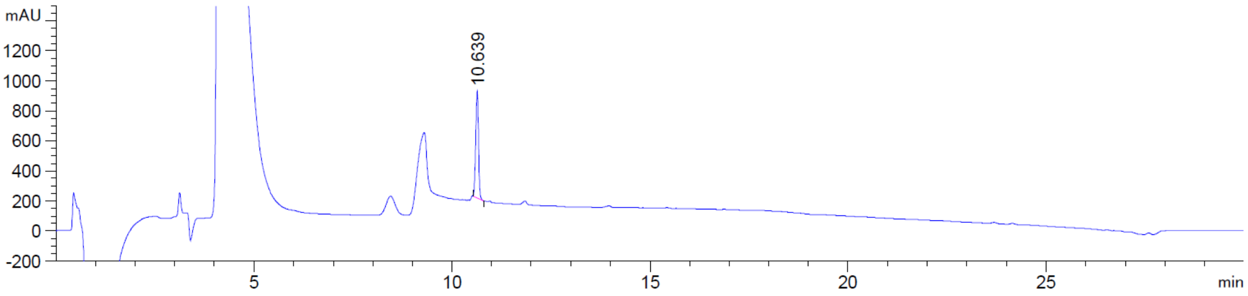

HArgSerGlnPheTyrLysHisAspAlaGlyCysGlyNH₂ (32)

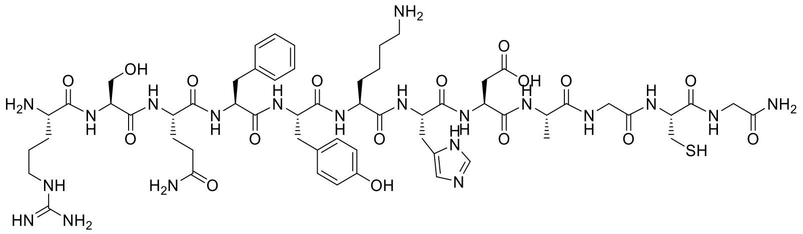

Following the general procedure, H-RSQFYKHDAGCG-NH_2 (**32**) was obtained as white amorphous solid.

HRMS (APPI/LTQ-Orbitrap) m/z: $[M + H_2]^{+2}$ Calcd for $C_{58}H_{88}N_{20}O_{17}S^{+2}$ 684.3173; Found 684.3144.

HPLC-UV Chromatogram (210 nm) of a 1 mM solution in DMF:

Retention time: 9.8 min

Absorbance area (mAU): 2795

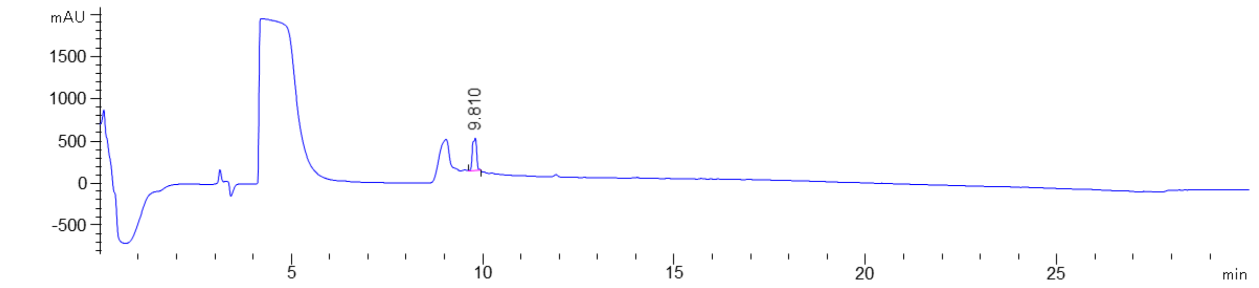

AcetTyrGlyGlyGluAlaAlaArgLysAlaCysAlaArgGluLysAlaAlaArgGluNH₂ (26a)

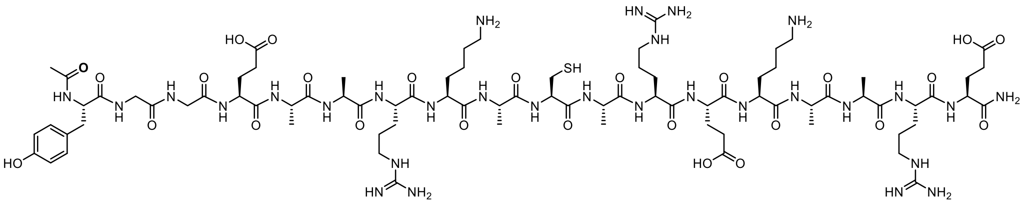

Following the general procedure, Ac-YGGEAARKACAREKAARE-NH_2 (**26a**) was obtained as white amorphous solid.

HRMS (nanochip-ESI/LTQ-Orbitrap) m/z: $[M + H_3]^{+3}$ Calcd for $C_{81}H_{139}N_{30}O_{26}S^{+3}$ 660.0060; Found 660.0075.

HPLC-UV Chromatogram (210 nm) of a 1 mM solution in DMF:

Retention time: 10.0 min

Absorbance area (mAU): 1933

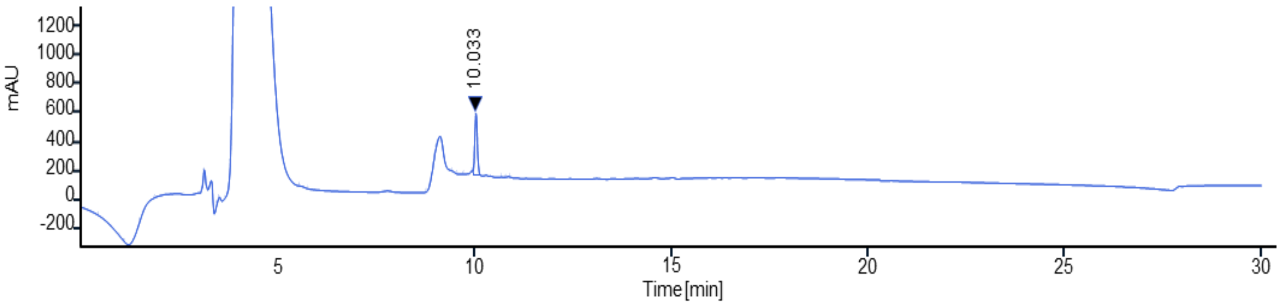

AcetTyrGlyGlyGluLysAlaArgGluAlaCysAlaArgGluLysAlaAlaArgGluNH₂ (26b)

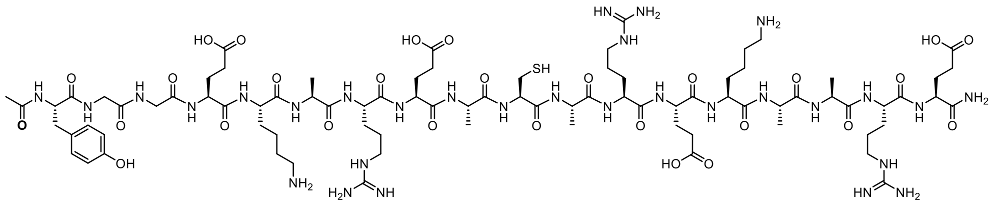

Following the general procedure, Ac-YGGEKAREACAREKAARE-NH_2 (**26b**) was obtained as white amorphous solid.

HRMS (nanochip-ESI/LTQ-Orbitrap) m/z: $[M + H_4]^{+4}$ Calcd for $C_{83}H_{142}N_{30}O_{28}S^{+4}$ 509.7577; Found 509.7594.

HPLC-UV Chromatogram (210 nm) of a 1 mM solution in DMF:

Retention time: 9.9 min

Absorbance area (mAU): 1861

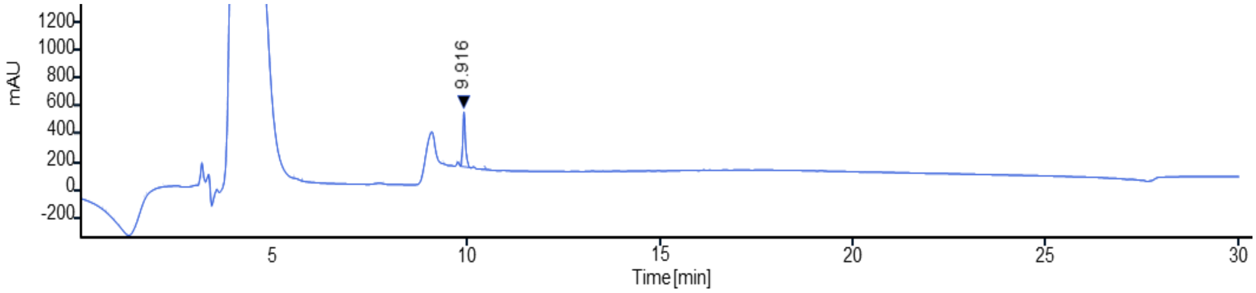

AcetTyrGlyGlyGluAlaAlaArgGluAlaCysAlaArgGluLysAlaAlaArgGluLysNH$_2$ (26c)

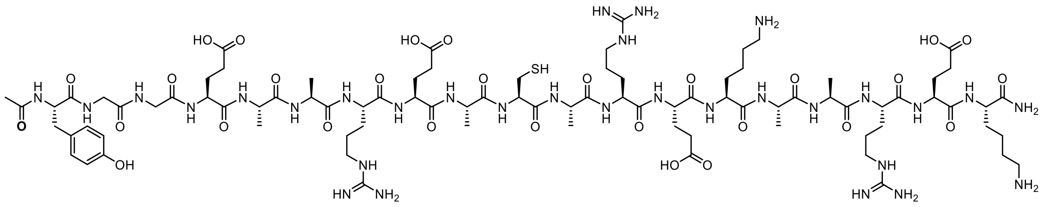

Following the general procedure, Ac-YGGEAAREACAREKAAREK-NH$_2$ (**26c**) was obtained as white amorphous solid.

HRMS (nanochip-ESI/LTQ-Orbitrap) m/z: $[M + H_4]^{+4}$ Calcd for $C_{86}H_{147}N_{31}O_{29}S^{+4}$ 527.5170; Found 527.5195.

HPLC-UV Chromatogram (210 nm) of a 1 mM solution in DMF:

Retention time: 10.4 min

Absorbance area (mAU): 2058

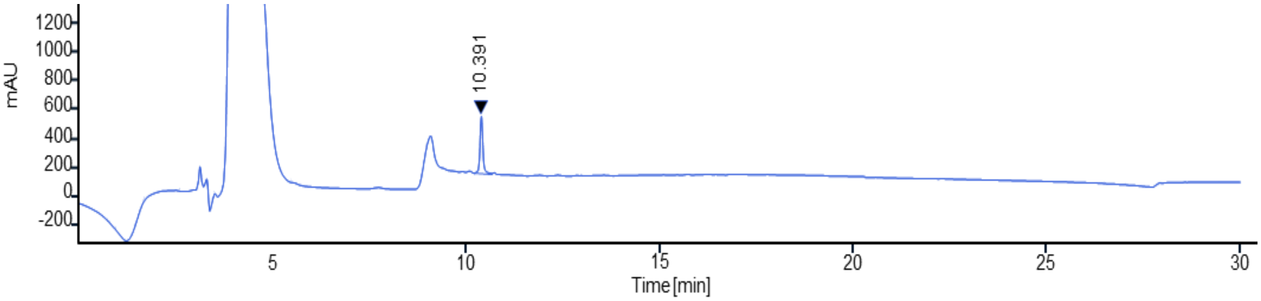

AcetTyrGlyGlyGluAlaAlaArgLys(ivDde)AlaCysAlaArgGluLysAlaAlaArgGluNH$_2$ (ivDde-26a)

Following the general procedure, Ac-YGGEAARK(ivDde)ACAREKAARE-NH$_2$ (**ivDde-26a**) was obtained as white amorphous solid.

HRMS (nanochip-ESI/LTQ-Orbitrap) m/z: $[M + H_2]^{+2}$ Calcd for $C_{94}H_{156}N_{30}O_{28}S^{+2}$ 1092.5708; Found 1092.5751.

HPLC-UV Chromatogram (210 nm) of a 1 mM solution in DMF:

Retention time: 12.1 min

Absorbance area (mAU): 2243

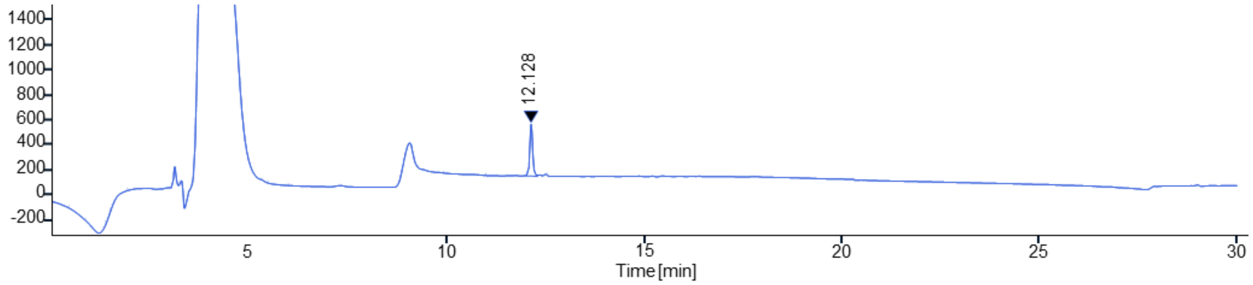

4. Synthesis of the stapling reagents

a. Cysteine-Cysteine stapling reagents

1-Chloro-3,3-bis(trifluoromethyl)-3-(1H)-1,2- benziodoxole (47)

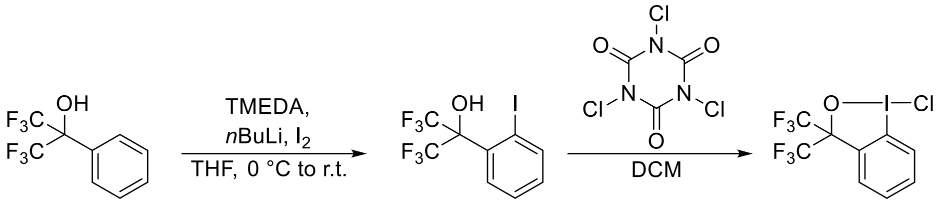

45 **46** **47**

Following a reported procedure,[2] tetramethylethylenediamine, distilled over KOH, (TMEDA, 1.26 mL, 8.32 mmol, 0.20 equiv.) was added to a solution of *n*-butyllithium in hexanes (*n*BuLi, 36.6 mL, 91.0 mmol, 2.20 equiv.). After 15 minutes, the solution was cooled to 0 °C and 1,1,1,3,3,3-hexafluoro-2- phenylpropan-2-ol (**46**) (7.00 mL, 41.6 mmol, 1.00 equiv.), in tetrahydrofuran (37 mL), was added dropwise. The reaction was stirred 30 minutes at 0 °C, followed by 18 hours at room temperature. Iodine (11.2 g, 44.1 mmol, 1.06 equiv.) was added in small portions at 0 °C. The mixture was stirred at 0 °C for 30 minutes and then at room temperature for 4 hours. The reaction was quenched with a solution of saturated aqueous ammonium chloride (100 mL) and extracted with diethyl ether (100 mL). The aqueous layer was then extracted twice with diethyl ether (3 x 50 mL). The organic layers were combined, washed twice with a solution of saturated aqueous sodium thiosulfate (2 x 50 mL), dried over magnesium sulfate, filtered and reduced to afford 1,1,1,3,3,3-hexafluoro-2-(2-iodophenyl)propan-2-ol (**46**) (12.7 g, 34.4 mmol, 82%) as an orange oil which was used without further purification. The crude oil was dissolved in DCM (34 mL) under air and trichloro*iso*cyanuric acid (2.80 g, 12.0 mmol, 0.35 equiv.) was then added portionwise at 0 °C. After 30 minutes, the resulting suspension was filtered and the filtrate was concentrated *in vacuo*. The resulting solid was dissolved into diethyl ether (50 mL), filtered, dried

---

[2] Perozzi, E. F.; Michalak, R. S.; Figuly, G. D.; Stevenson, W. H.; Dess, D. B.; Ross, M. R.; Martin, J. C. *J. Org. Chem.* **1981**, *46*, 1049.

and washed with small amounts of dichloromethane to afford 1-chloro-3,3-bis(trifluoromethyl)-3-(1H)-1,2- benziodoxole (**47**) (1.59 g, 3.93 mmol, 11%) as a yellow solid.

^{1}H NMR ($CDCl_3$, 400 MHz) δ 8.09 (d, *J* = 8.5 Hz, 1H, C_{Ar}-*H*), 7.85 (dt, *J* = 8.6, 4.3 Hz, 1H, C_{Ar}-*H*), 7.73 (d, *J* = 4.6 Hz, 2H, C_{Ar}-*H*).
Spectroscopic data was consistent with the values reported in literature.[3]

1-Hydroxy-3,3-bis(trifluoromethyl)-3-(1H)-1,2-benziodoxole (10)

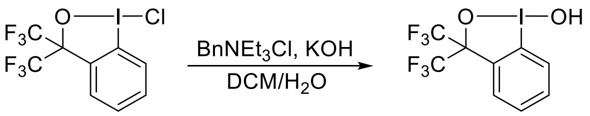

47 **10**

Following a reported procedure,[4] benzyltriethylammonium chloride (63 mg, 0.20 mmol, 0.05 equiv.) was added to a stirring solution of 1-chloro-3,3-bis(trifluoromethyl)-3-(1H)-1,2-benziodoxole (**47**) (1.59 g, 3.93 mmol, 1.00 equiv.) in dichloromethane (27 mL) and potassium hydroxide (0.22 g, 3.9 mmol, 1.0 equiv.) in water (4 mL). The reaction was stirred for 5 hours under air. The organic layer was separated, dried over magnesium sulfate and concentrated *in vacuo*. The resulting solid was purified over a silica plug with ethyl acetate, then recrystallized in ethyl acetate and washed with pentane to afford 1-hydroxy-3,3-bis(trifluoromethyl)-3-(1H)-1,2-benziodoxole **10** (0.653 g, 1.69 mmol, 43%) as a colorless solid.

^{1}H NMR (DMSO-d_6, 400 MHz) δ 7.81-7.88 (m, 2H, C_{Ar}-*H*), 7.76 (d, *J* = 7.7 Hz, 1H, C_{Ar}-*H*), 7.67 (ddd, *J* = 8.0, 6.7, 1.5 Hz, 1H, C_{Ar}-*H*).

Spectroscopic data was consistent with the values reported in literature.[4]

Diisopropylbis((trimethylsilyl)ethynyl)silane (11c)

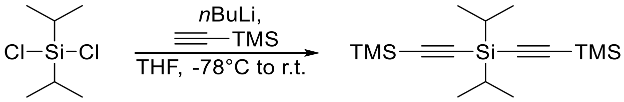

48 **11c**

To a solution of ethynyltrimethylsilane (1.4 mL, 10 mmol, 2.0 equiv.) in THF (25 mL) at -78°C, *n*-butyl lithium (2.5 M in hexane, 4.4 mL, 11 mmol, 2.2 equiv.) was added and the mixture stirred for 10 minutes at -78 °C and 1 hour at room temperature. Dichlorodiisopropylsilane (**48**) (0.90 mL, 5.0 mmol, 1.0 equiv.) was then added and the reaction was allowed to stir overnight at room temperature. The reaction was quenched with water for 5 minutes and extracted with diethylether. The solvents were removed under reduced pressure and the crude product was purified by flash chromatography (Pentane) to afford diisopropylbis((trimethylsilyl)ethynyl)silane **11c** (0.82 g, 2.6 mmol, 52%) as a clear oil.

Rf= 0.8 (pentane).

---

[3] Cvengros, J.; Stolz, D.; Togni, A. *Synthesis* **2009**, 2818.
[4] Blake, A. J.; Novak, A.; Davies, M.; Robinson, R. I.; Woodward, S. *Synth. Commun.* **2009**, *39*, 1065–1075

^{1}**H NMR** ($CDCl_3$, 400 MHz): δ 1.06 (d, *J* = 6.4 Hz, 12H, CH(C*H*$_3$)$_2$), 0.94-1.03 (m, 2H, C*H*(CH$_3$)$_2$), 0.18 (s, 18H, Si-C*H*$_3$).

^{13}**C NMR** ($CDCl_3$, 101 MHz): δ 116.8 (-*C*C-Si), 106.8 (-C*C*-Si), 17.7 (*C*H(CH$_3$)$_2$), 12.3 (CH(*C*H$_3$)$_2$), 0.0 (Si-*C*H$_3$).

IR (ν_{max}, cm^{-1}): 2955 (m), 2867 (w), 1462 (w), 1251 (m), 995 (w), 842 (s), 778 (s), 671 (w).

HRMS (ESI/QTOF) m/z: $[M + Ag]^+$ Calcd for $C_{16}H_{32}AgSi_3^+$ 415.0857; Found 415.0857.

Dicyclohexylbis((trimethylsilyl)ethynyl)silane (11d)

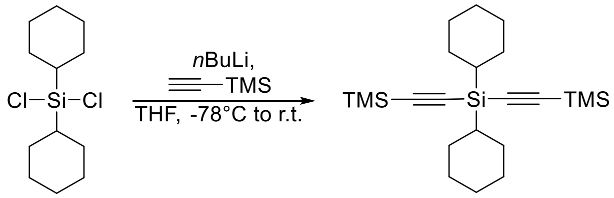

49 **11d**

To a solution of ethynyltrimethylsilane (1.4 mL, 10 mmol, 2.0 equiv.) in THF (25 mL) at -78°C, *n*-butyl lithium (2.5 M in hexane, 4.4 mL, 11 mmol, 2.2 equiv.) was added and the mixture stirred for 10 minutes at -78 °C and 1 hour at room temperature. Dichlorodicyclohexyl silane (**49**) (1.2 mL, 5.0 mmol, 1.0 equiv.) was then added and the reaction was allowed to stir overnight at room temperature. The reaction was quenched with water for 5 minutes and extracted with diethylether. The solvents were removed under reduced pressure and the crude product was purified by flash chromatography (Pentane) to afford dicyclohexylbis((trimethylsilyl)ethynyl)silane **11d** (0.72 g, 1.8 mmol, 36%) as a white solid.

Rf = 0.6 (pentane).

^{1}**H NMR** ($CDCl_3$, 400 MHz): δ 1.79-1.70 (m, 10H, C*H*$_2$), 1.32-1.23 (m, 10H, C*H*$_2$), 0.81 (m, 2H, SiC*H*), 0.18 (s, 18H, Si-C*H*$_3$).

^{13}**C NMR** ($CDCl_3$, 101 MHz): δ 116.8 (-*C*C-Si), 107.6 (-C*C*-Si), 28.0 (*C*H$_2$), 27.5 (*C*H$_2$), 26.9 (*C*H$_2$), 23.6 (*C*H(CH$_2$)$_2$), 0.0 (Si-*C*H$_3$).

m.p. (°C): 116-118.

IR (ν_{max}, cm^{-1}): 2966 (w), 2919 (m), 2847 (w), 1447 (w), 1248 (m).

HRMS (ESI/QTOF) m/z: $[M + Ag]^+$ Calcd for $C_{22}H_{40}AgSi_3^+$ 495.1483; Found 495.1483.

1,1,3,3-Tetraisopropyl-1,3-bis((trimethylsilyl)ethynyl)disiloxane (11e)

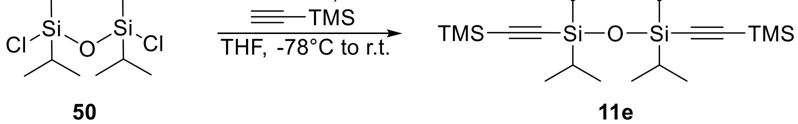

50 **11e**

To a solution of ethynyltrimethylsilane (0.50 mL, 3.6 mmol, 2.0 equiv.) in diethylether (9.2 mL, 0.4 M) at -78°C, *n*-butyllithium (2.5 M in hexane, 1.6 mL, 4.0 mmol, 2.2.0 equiv.) was added and the

mixture stirred for 10 minutes at -78 °C and 1 hour at room temperature. 1,3-Dichloro-1,1,3,3-tetraisopropyldisiloxane (**50**) (0.57 mL, 1.8 mmol, 1.0 equiv.) was then added and the reaction was allowed to stir overnight at room temperature. The reaction was quenched with water for 5 minutes and extracted with diethylether. The solvents were removed under reduced pressure and the crude product was purified by flash chromatography (1% EtOAc/Pentane) to afford 1,1,3,3-tetraisopropyl-1,3-bis((trimethylsilyl)ethynyl)disiloxane **11e** (0.16 g, 0.37 mmol, 21%) as a clear oil. Minor impurities could not be separated completely, the product was used as such in the next step.

Rf = 0.5 (1% EtOAc in pentane).

^{1}H NMR ($CDCl_3$, 400 MHz): δ 1.05 (d, *J* = 7.0 Hz, 24H, CCH_3), 0.98-0.87 (m, 4H, $CHCH_3$), 0.16 (s, 18H, $SiCH_3$).

^{13}C NMR ($CDCl_3$, 101 MHz) (isolated with an impurity): δ 115.7 (-*C*C-Si), 109.1 (-C*C*-Si), 29.9, 17.1, 16.9, 13.1, 0.0 (Si*C*H_3).

IR (ν_{max}, cm^{-1}) 2957 (m), 2926 (w), 2867 (w), 1464 (w), 1251 (m), 1082 (w), 997 (w), 841 (s), 761 (s), 683 (m), 608 (w).

HRMS (APPI/LTQ-Orbitrap) m/z: $[M + H]^+$ Calcd for $C_{22}H_{47}OSi_4^+$ 439.2698; Found 439.2686.

1,4-Bis((3,3-bis(trifluoromethyl)-1l3-benzo[d][1,2]iodaoxol-1(3H)-yl)ethynyl)benzene (8a)

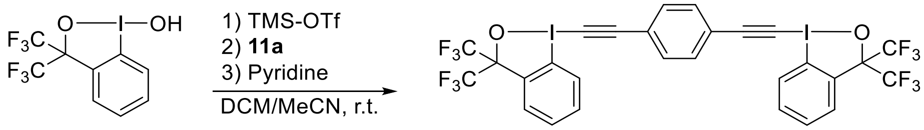

To a solution of 3,3-bis(trifluoromethyl)-1l3-benzo[d][1,2]iodaoxol-1(3H)-ol (**10**) (30 mg, 0.078 mmol, 2.5 equiv.) in DCM (0.4 mL), trimethylsilyl trifluoromethanesulfonate (0.015 mL, 0.078 mmol, 2.5 equiv.) was added and the reaction was allowed to stir at room temperature for 20 minutes, before adding 1,4-bis((trimethylsilyl)ethynyl)benzene (**11a**) (8.4 mg, 0.031 mmol. 1.0 equiv.). The reaction was left stirring overnight and then quenched with $NaHCO_3$ (sat. aqueous solution) for 10 minutes. The organic layer was washed with $NaHCO_3$ (sat. aqueous solution). After partial evaporation of the solvents under reduced pressure, 1,4-bis((3,3-bis(trifluoromethyl)-1l3-benzo[d][1,2]iodaoxol-1(3H)-yl)ethynyl)benzene **8a** precipitated as a white solid (25 mg, 0.029 mmol, 94%). The characterization data matches the data reported in the literature by Wu *et al.*[5]

Rf = 0.3 (DCM).

^{1}H NMR ($CDCl_3$, 400 MHz): δ 8.35 (dd, *J* = 8.0, 1.3 Hz, 2H, C_{Ar}-*H*), 7.87 (dtd, *J* = 14.0, 7.1, 1.6 Hz, 4H, C_{Ar}-*H*), 7.80 (d, *J* = 7.5 Hz, 2H, C_{Ar}-*H*), 7.76 (s, 4H, C_{Ar}-*H*).

^{13}C NMR (CDCl3, 101 MHz): δ 133.8 (C_{Ar}), 132.7 (C_{Ar}), 131.6 (C_{Ar}), 130.1 (C_{Ar}), 129.6 (C_{Ar}), 129.2 (C_{Ar}), 123.7 (q, *J* = 291.0 Hz, CF_3), 122.2 (C_{Ar}), 112.0 (C_{Ar}), 102.7, 82.4-81.3 (m, *C*O), 58.7.

[5] J. Wu, N. Yoshikai, *Angew. Chem. Int. Ed.* **2015**, *54*, 11107–11111.

Decomposition point (°C): 225.1-226.9.

IR (v_{max}, cm^{-1}): 2360 (s), 2338 (s), 2143 (w), 1264 (m), 1184 (m), 1151 (m), 950 (m), 836 (w), 761 (m), 733 (m), 692 (m), 660 (m), 634 (m), 620 (s).

HRMS (ESI/QTOF) m/z: $[M + H]^+$ Calcd for $C_{28}H_{13}F_{12}I_2O_2^+$ 862.8808; Found 862.8807.

1,3-Bis((3,3-bis(trifluoromethyl)-1l3-benzo[d][1,2]iodaoxol-1(3H)-yl)ethynyl)benzene (8b)

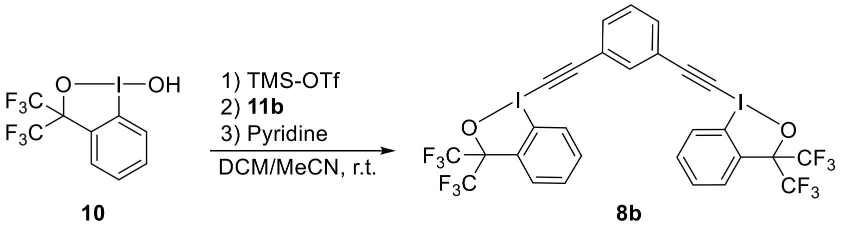

To a solution of 3,3-bis(trifluoromethyl)-1l3-benzo[d][1,2]iodaoxol-1(3H)-ol (**10**) (30 mg, 0.078 mmol, 2.5 equiv.) in DCM (0.4 mL), trimethylsilyl trifluoromethanesulfonate (0.015 mL, 0.078 mmol, 2.5 equiv.) was added and the reaction was allowed to stir at room temperature for 20 minutes, before adding 1,3-bis((trimethylsilyl)ethynyl)benzene (**11b**) (8.4 mg, 0.031 mmol. 1.0 equiv.). The reaction was left stirring overnight and then quenched with $NaHCO_3$ (sat. aqueous solution) for 15 minutes. The organic layer was washed with $NaHCO_3$ (sat. aqueous solution) and the solvents were then evaporated under reduced pressure. The crude product was purified by preparative thin layer chromatography (DCM) to afford 1,3-bis((3,3-bis(trifluoromethyl)-1l3-benzo[d][1,2]iodaoxol-1(3H)-yl)ethynyl)benzene **8b** (15 mg, 0.018 mmol, 57%) as a white solid.

Rf = 0.3 (DCM).

^{1}H NMR ($CDCl_3$, 400 MHz): δ 8.38 (dd, J = 7.8, 1.5 Hz, 2H, C_{Ar}-H), 7.97 (t, J = 1.7 Hz, 1H C_{Ar}-H), 7.88 (dtd, J = 14.4, 7.1, 1.6 Hz, 4H C_{Ar}-H), 7.81-7.77 (m, 4H C_{Ar}-H), 7.59 (t, J = 7.8 Hz, 1H C_{Ar}-H).

^{13}C NMR ($CDCl_3$, 101 MHz): δ 135.7 (C_{Ar}), 133.9 (C_{Ar}), 133.8 (C_{Ar}), 131.6 (C_{Ar}), 130.1 (C_{Ar}), 129.7 (C_{Ar}), 129.2 (C_{Ar}), 123.7 (q, J = 290.9 Hz, CF_3), 121.8 (C_{Ar}), 111.9 (C_{Ar}), 102.2, 81.8 (m, CO), 57.1.[6]

Decomposition point (°C): 245.2-247.1.

IR (v_{max}, cm^{-1}): 2360 (w), 2331 (w), 2140 (w), 1809 (w), 1712 (w), 1570 (w), 1469 (w), 1435 (w), 1261 (S), 1184 (S), 1145 (S), 955 (S), 796 (m), 757 (m), 728 (m), 685 (m), 655 (m).

HRMS (ESI/QTOF) m/z: $[M + H]^+$ Calcd for $C_{28}H_{13}F_{12}I_2O_2^+$ 862.8808; Found 862.8804.

[6] One peak was unresolved.

Bis((3,3-bis(trifluoromethyl)-1l3-benzo[d][1,2]iodaoxol-1(3H)-yl)ethynyl)diisopropylsilane (8c)

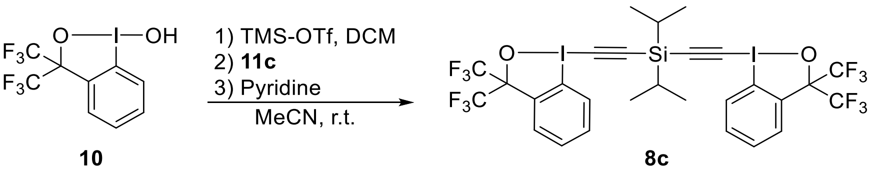

To a solution of 1-Hydroxy-3,3-bis(trifluoromethyl)-3-(1H)-1,2-benziodoxole (**10**) (0.20 g, 0.52 mmol, 2.5 equiv.) in DCM (6.6 mL), trimethylsilyl trifluoromethanesulfonate (0.10 mL, 0.52 mmol, 2.5 equiv.) was added and the mixture was allowed to stir for 20 minutes at room temperature. The solvents were then removed under reduced pressure and the solid was redissolved in MeCN (4.0 mL) and diisopropylbis((trimethylsilyl)ethynyl)silane (**11c**) (0.064 g, 0.21 mmol, 1.0 equiv.) was added. After 20 minutes pyridine (0.025 mL, 0.31 mmol, 2.5 equiv.) was added and the reaction was stirred for 20 minutes, the solvents were then evaporated under reduced pressure and the solid was partitioned between DCM and water. The organic layer was then washed with sat. aqueous solution of $NaHCO_3$ and the solvents removed under reduced pressure. The crude product was purified by flash chromatography (DCM) to yield **8c** (0.128 g, 0.142 mmol, 69%) as a white solid.

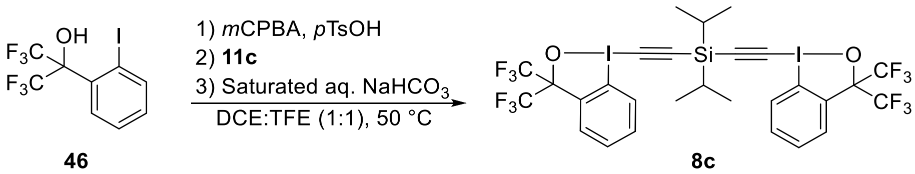

One pot procedure: 1,1,1,3,3,3-hexafluoro-2-(2-iodophenyl)propan-2-ol (0.575 g, 1.55 mmol, 2.50 equiv.), *p*-Toluene sulfonic acid (0.294 g, 1.55 mmol, 2.50 equiv.) and *m*CPBA (0.295 g, 1.70 mmol, 2.75 equiv.) were dissolved in a 1 to 1 mixture of DCE and TFE (4.6 mL). The mixture was then heated for 1 hour at 50 °C before adding diisopropylbis((trimethylsilyl)ethynyl)silane (**11c**) (0.240 g, 0.777 mmol, 1.00 equiv.). The reaction was left to stir at 50 °C for 24 hours. The solvents were then evaporated under reduced pressure and the residue was partitioned between DCM and $NaHCO_3$ (sat. aqueous solution). The organic layer was washed with $NaHCO_3$ (sat. aqueous solution), then evaporated under reduce pressure to afford the crude product. The crude product was purified by column chromatography (DCM) to afford **8c** (0.161 g, 0.179 mmol, 23%) as a white solid.

Rf = 0.6 (DCM).

^{1}H NMR ($CDCl_3$, 400 MHz): δ 8.28 (d, *J* = 8.2 Hz, 2H, C_{Ar}-*H*), 7.84 (d, *J* = 7.6 Hz, 2H, C_{Ar}-*H*), 7.70 (t, *J* = 7.4 Hz, 2H, C_{Ar}-*H*), 7.64 (td, *J* = 7.8, 7.3, 1.6 Hz, 2H, C_{Ar}-*H*), 1.21 (m, 14H, Si-C*H*, Si-CHCH_3).

^{13}C NMR ($CDCl_3$, 101 MHz): δ 133.1 (C_{Ar}-I), 131.5 (C_{Ar}-CO), 130.2 (C_{Ar}-H), 130.1 (C_{Ar}-H), 128.3 (C_{Ar}-H), 123.6 (q, *J* = 290.1 Hz, CF_3), 110.9 (C_{Ar}-H), 106.8 (*C*C-I), 81.7 (dt, *J* = 59.3, 29.3 Hz, *C*O), 74.0 (C*C*-I), 17.7 (CH_3), 12.4 (Si-*C*).

m.p. (°C): 190-191.

IR (ν_{max}, cm^{-1}): 2937 (w), 2869 (w), 1461 (w), 1266 (m), 1182 (s), 1148 (s), 1007 (w), 965 (m), 948 (s), 878 (w), 759 (m), 707 (s).

HRMS (APPI/LTQ-Orbitrap) m/z: [M + H]+ Calcd for $C_{28}H_{23}F_{12}I_2O_2Si^+$ 900.9360; Found 900.9359.

Bis((3,3-bis(trifluoromethyl)-1l3-benzo[d][1,2]iodaoxol-1(3H)-yl)ethynyl)dicyclohexylsilane (8d)

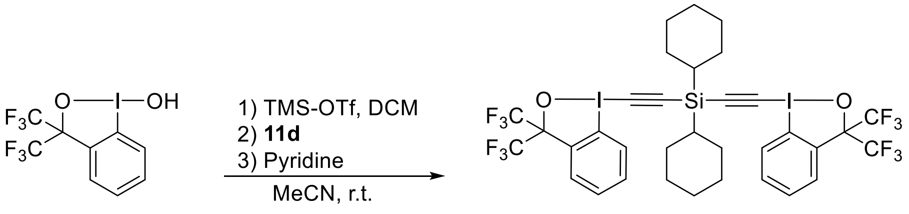

To a solution of 1-Hydroxy-3,3-bis(trifluoromethyl)-3-(1H)-1,2-benziodoxole (**10**) (0.025 g, 0.065 mmol, 2.5 equiv.) in DCM (0.8 mL), trimethylsilyl trifluoromethanesulfonate (0.013 mL, 0.065 mmol, 2.5 equiv.) was added and the mixture was allowed to stir for 20 minutes at room temperature. The solvents were then removed under reduced pressure and the solid was redissolved in MeCN (0.5 mL) and dicyclohexylbis((trimethylsilyl)ethynyl)silane (**11d**) (0.010 g, 0.026 mmol, 1.0 equiv.) was added. After 20 minutes pyridine (0.0031 mL, 0.039 mmol, 2.5 equiv.) was added and the reaction was stirred for 20 minutes, the solvents were then evaporated under reduced pressure and the solid was partitioned between DCM and water. The organic layer was then washed with a sat. aqueous solution of $NaHCO_3$ and the solvents removed under reduced pressure. The crude product was purified by flash chromatography (DCM) to yield **8d** (0.010 g, 0.010 mmol, 40%) as a white solid.

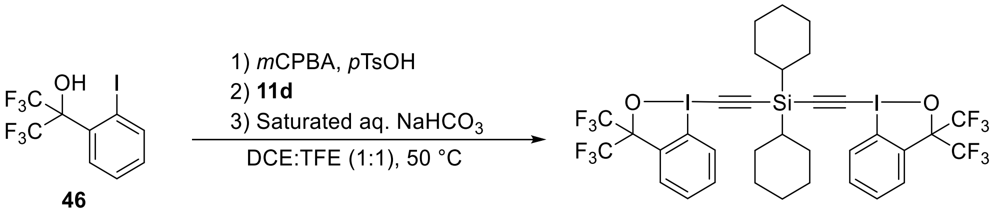

One pot procedure: 1,1,1,3,3,3-hexafluoro-2-(2-iodophenyl)propan-2-ol (**46**) (0.500 g, 1.35 mmol, 2.50 equiv.), *p*-Toluene sulfonic acid (0.256 g, 1.35 mmol, 2.5 equiv.) and *m*CPBA (0.256 g, 1.49 mmol, 2.75 equiv.) were dissolved in a 1 to 1 mixture of DCE and TFE (4.6 mL, 0.17 M). The mixture was then heated for 1 hour at 50 °C before adding dicyclohexylbis((trimethylsilyl)ethynyl)silane (**11d**) (0.240 g, 0.540 mmol, 1.00 equiv.). The reaction was left to stir at 50 °C for 24 hours. The solvents were then evaporated under reduced pressure and the residue was partitioned between DCM and $NaHCO_3$ (sat. aqueous solution). The organic layer was washed with $NaHCO_3$ (sat. aqueous solution), then evaporated under reduced pressure to afford the crude product. The crude product was purified by column chromatography (DCM) to afford **8d** (0.225 g, 0.229 mmol, 42%) as a white solid.

Rf = 0.6 (DCM).

^{1}**H NMR** ($CDCl_3$, 400 MHz): δ 8.29 (dd, *J* = 8.3, 1.1 Hz, 2H, C_{Ar}-*H*), 7.85 (dq, *J* = 7.7. 1.4 Hz, 2H, C_{Ar}-*H*), 7.70 (td, *J* = 7.5, 1.1 Hz, 2H, C_{Ar}-*H*), 7.63 (ddd, *J* = 8.6, 7.2, 1.6 Hz, 2H C_{Ar}-*H*), 1.79-1.92 (m, 10H, CH_2), 1.25-1.44 (m, 10H, CH_2), 1.04 (tt, *J* = 12.3, 2.8 Hz, 2H, Si-C*H*).

^{13}**C NMR** ($CDCl_3$, 101 MHz): δ 133.1 (*C*$_{Ar}$-I), 131.5 (*C*$_{Ar}$-CO), 130.2 (*C*$_{Ar}$-H), 130.1 (*C*$_{Ar}$-H), 128.4 (*C*$_{Ar}$-H), 123.6 (q, *J* = 291 Hz, *C*F_3), 111.0 (*C*$_{Ar}$-H), 107.2 (*C*C-I), 82.0-81.4 (m, *C*O), 74.0 (C*C*-I), 27.7 (*C*H_2), 27.7 (*C*H_2), 26.7 (*C*H_2), 23.6 (Si*C*)

m.p. (°C): 198-200.

IR (ν_{max}, cm^{-1}): 2918 (w), 2849 (w), 1440 (w), 1267 (s), 1258 (s), 1180 (s), 1162 (s), 1148 (s), 1002 (w), 965 (s), 951 (s), 882 (w), 845 (w), 820 (w), 761 (m), 740 (s), 730 (s), 707 (s), 660 (s), 641 (m).

HRMS (APPI/LTQ-Orbitrap) m/z: $[M + H]^+$ Calcd for $C_{34}H_{31}F_{12}I_2O_2Si^+$ 980.9986; Found 981.0011.

1,3-Bis((3,3-bis(trifluoromethyl)-1l3-benzo[d][1,2]iodaoxol-1(3H)-yl)ethynyl)-1,1,3,3-tetraisopropyldisiloxane (8e)

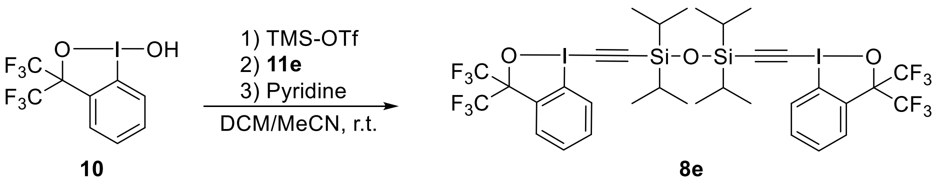

To a solution of 1-hydroxy-3,3-bis(trifluoromethyl)-3-(1H)-1,2-benziodoxole (**10**) (0.274 g, 0.709 mmol, 2.50 equiv.) in DCM (9.1 mL), trimethylsilyl trifluoromethanesulfonate (0.137 mL, 0.709 mmol, 2.50 equiv.) was added and the mixture was allowed to stir for 20 minutes at room temperature. The solvents were then removed under reduced pressure and the solid was redissolved in MeCN (5.5 mL) and 1,1,3,3-tetraisopropyl-1,3-bis((trimethylsilyl)ethynyl)disiloxane (**11e**) (0.125 g, 0.284 mmol, 1.00 equiv.) was added. After 20 minutes pyridine (0.057 mL, 0.71 mmol, 2.50 equiv.) was added and the reaction was stirred for 20 minutes, the solvents were then evaporated under reduced pressure and the solid was partitioned between DCM and water. The organic layer was then washed with $NaHCO_3$ (sat. aqueous solution) and the solvents removed under reduced pressure. The crude product was purified by flash chromatography (5 to 10% EtOAc/Pentane) to yield **8e** (0.189 g, 0.184 mmol, 65%) as a white solid.

Rf = 0.4 (5% EtOAc/Pentane).

^{1}**H NMR** ($CDCl_3$, 400 MHz): δ 8.29 (dd, *J* = 8.2, 1.1 Hz, 2H, C_{Ar}-*H*), 7.81 (dq, *J* = 7.7, 1.4 Hz, 2H, C_{Ar}-*H*), 7.66 (td, *J* = 7.4, 1.2 Hz, 2H, C_{Ar}-*H*), 7.59 (ddd, *J* = 8.6, 7.1, 1.6 Hz, 2H, C_{Ar}-*H*), 1.16-1.06 (m, 28H, C*H*$(CH_3)_2$ and CH(CH_3)$_2$).

^{13}**C NMR** ($CDCl_3$, 101 MHz): δ 132.9 (*C*$_{Ar}$-I), 131.5 (*C*$_{Ar}$-CO), 130.2 (*C*$_{Ar}$-H), 130.0 (*C*$_{Ar}$-H), 128.3 (*C*$_{Ar}$-H), 123.6 (q, *J* = 290 Hz, *C*F_3), 111.0 (*C*$_{Ar}$-H), 110.7 (*C*C-I), 81.6 (dt, *J* = 59.4, 29.5 Hz, *C*O), 17.2 (*C*H_3), 14.0 (*C*Si).

m.p. (°C): 193-195.

IR (ν_{max}, cm^{-1}): 3667 (w), 2978 (s), 2902 (s), 1449 (w), 1401 (m), 1251 (m), 1222 (m), 1181 (m), 1152 (m), 1057 (s), 964 (m), 946 (m), 877 (m), 763 (m), 728 (m), 688 (s).

HRMS m/z: $[M + H]^+$ Calcd for $C_{34}H_{37}F_{12}I_2O_3Si_2^+$ 1031.0174; Found 1031.0197.

b. Cysteine-Lysine stapling reagents

2-Iodosylbenzoic acid (14)

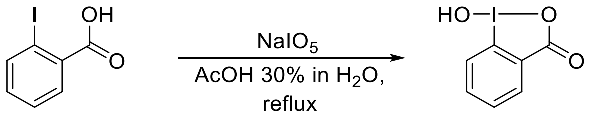

51 **14**

Following a reported procedure,[7] sodium periodate (18.1 g, 85.0 mmol, 1.00 equiv.) and 2-iodobenzoic acid (**51**) (20.0 g, 81.0 mmol, 1.05 equiv.) were suspended in 30% (v:v) aq. AcOH (60 mL). The mixture was vigorously stirred under reflux for 4 hours protected from light and then cooled to room temperature. The mixture was diluted with cold water (0.20 L). The resulting solid was collected by vacuum filtration and washed with cold water (3 x 20 mL) and cold acetone (3 x 20 mL) and then air-dried in darkness overnight to afford 2-iodosylbenzoic acid (**14**) (10.1 g, 38.4 mmol, 95%) as a white solid.

^{1}H NMR (400 MHz, DMSO-d_6) δ 8.01 (dd, J = 7.5, 1.5 Hz, 1H, $C_{Ar}H$), 7.96 (ddd, J = 8.5, 7.2, 1.6 Hz, 1H, $C_{Ar}H$), 7.87 – 7.82 (m, 1H, $C_{Ar}H$), 7.71 (td, J = 7.3, 1.0 Hz, 1H, $C_{Ar}H$);

^{13}C NMR (100 MHz, DMSO-d_6) δ 167.7(CO_2), 134.5(C_{Ar}), 131.5(C_{Ar}), 131.1(C_{Ar}), 130.4(C_{Ar}), 126.3(C_{Ar}), 120.4(C_{Ar}).

Spectroscopic data was consistent with the values reported in literature.[7]

Perfluorophenyl 4-((trimethylsilyl)ethynyl)benzoate (15a)

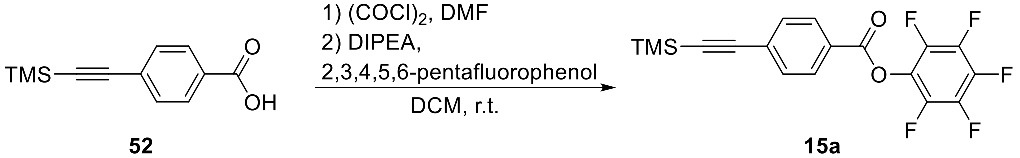

52 **15a**

In an oven-dried Schlenk flask, to a solution of 4-((trimethylsilyl)ethynyl)benzoic acid (**52**) (150 mg, 0.687 mmol, 1.00 equiv.) in anhydrous DCM (2.3 mL) oxalyl dichloride (74 μL, 0.86 mmol, 1.3 equiv.) and DMF (53 μL, 0.69 mmol, 1.0 equiv.) were added at room temperature. The mixture was stirred for 1 hour and concentrated to dryness to yield 4-((trimethylsilyl)ethynyl)benzoyl chloride, which was used as crude for further synthesis.

To a solution of 4-((trimethylsilyl)ethynyl)benzoyl chloride (54 mg, 0.23 mmol, 1.0 equiv.) in DCM (1 mL) at room temperature, DIPEA (0.044 mL, 0.25 mmol, 1.1 equiv.) and pentafluorophenol (44 mg, 0.24 mmol, 1.05 equiv.) were added. The solution was stirred for 2.5 hours and directly filtered through a Celite® pad with pentane as eluent. The solvents were then removed under reduced pressure to afford the crude product **15a** as a white solid (84 mg, 0.22 mmol, 95%) with enough purity to continue the synthesis.

Rf = 0.5 (pentane).

---

[7] Brand, J. P.; Waser, J. *Synthesis* **2012**, *44*, 1155.

^{1}**H NMR** ($CDCl_3$, 400 MHz): δ 8.13 (d, *J* = 8.6 Hz, 2H, C_{Ar}-*H*), 7.61 (d, *J* = 8.5 Hz, 2H, C_{Ar}-*H*), 0.28 (s, 9H, SiCH_3).

^{13}**C NMR** ($CDCl_3$, 101 MHz): δ 162.2 (*C*O_2), 141.5 (dm, *J* = 252.2 Hz, *C*$_{Ar}$-F), 139.8 (dm, *J* = 253.3 Hz, *C*$_{Ar}$-F), 138.1 (dm, *J* = 254.3 Hz, *C*$_{Ar}$-F), 132.4 (*C*$_{Ar}$), 130.6 (*C*$_{Ar}$), 129.9 (*C*$_{Ar}$), 126.4 (*C*$_{Ar}$), 125.4 (m, *C*$_{Ar}$-O), 0.0 (Si*C*H_3).

m.p. (°C): 123-125.

IR (ν_{max}, cm^{-1}) 2960 (w), 2161 (w), 1766 (s), 1603 (w), 1516 (s), 1247 (s), 1179 (m), 1045 (s), 991 (s), 835 (s), 757 (s), 687 (m), 635 (m).

HRMS (APPI/LTQ-Orbitrap) m/z: $[M + H]^+$ Calcd for $C_{18}H_{14}F_5O_2Si^+$ 385.0678; Found 385.0668.

Perfluorophenyl 4-((3-oxo-1λ^3-benzo[d][1,2]iodaoxol-1(3H)-yl)ethynyl)benzoate (9a)

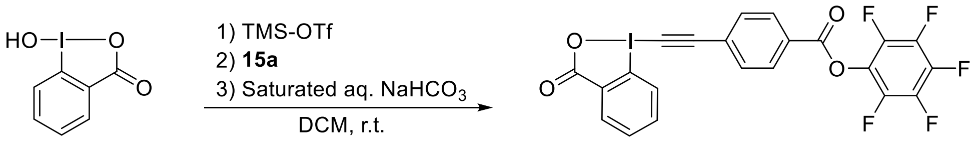

14 **9a**

To a solution of 2-iodosyl benzoic acid (**14**) (264 mg, 0.710 mmol, 1.00 equiv.) in DCM (2.2 mL), trimethylsilyl trifluoromethanesulfonate (0.15 mL, 0.78 mmol, 1.1 equiv.) was added and the reaction was allowed to stir at room temperature for 1 hour, before adding perfluorophenyl 4-((trimethylsilyl)ethynyl)benzoate (**15a**) (300 mg, 0.780 mmol, 1.10 equiv.). The reaction was left stirring for 4.5 hours and then quenched with $NaHCO_3$ (sat. aqueous solution) for 15 minutes. The organic layer was washed with $NaHCO_3$ (sat. aqueous solution) and the solvents were then evaporated under reduced pressure. The crude product was purified by flash chromatography (1.5% MeOH/DCM) to afford perfluorophenyl 4-((3-oxo-1λ^3-benzo[d][1,2]iodaoxol-1(3H)-yl)ethynyl)benzoate **9a** (293 mg, 0.525 mmol, 74%) as a white solid.

Rf = 0.2 (1.5 % MeOH/ DCM).

^{1}**H NMR** ($CDCl_3$, 400 MHz): δ 8.42 (dd, *J* = 7.2, 2.0 Hz, 1H, C_{Ar}-*H*), 8.27-8.24 (m, 3H, C_{Ar}-*H*), 7.83-7.76 (m, 4H, C_{Ar}-*H*).

^{13}**C NMR** ($CDCl_3$, 101 MHz): δ 166.9 (*C*O_2), 161.7 (*C*O_2), 141.4 (dm, *J* = 251.9 Hz, *C*$_{Ar}$-F) *C*$_{Ar}$-F), 139.9 (dm, *J* = 254.0 Hz, *C*$_{Ar}$-F), 138.1 (dm, *J* = 254.3 Hz, *C*$_{Ar}$-F), 135.2 (*C*$_{Ar}$), 133.2 (*C*$_{Ar}$), 132.7 (*C*$_{Ar}$), 131.9 (*C*$_{Ar}$), 131.4 (*C*$_{Ar}$), 131.0 (*C*$_{Ar}$), 128.4 (*C*$_{Ar}$), 127.1 (*C*$_{Ar}$), 126.6 (*C*$_{Ar}$), 125.2 (m, *C*$_{Ar}$-O), 116.3 (*C*$_{Ar}$), 104.3 (*C*-I), 56.3 (C*C*-C_{Ar}).

m.p. (°C): 240-245.

IR (ν_{max}, cm^{-1}): 3022 (w), 2941 (w), 2849 (w), 2154 (w), 1758 (s), 1623 (m), 1518 (s), 1321 (w), 1240 (m), 1181 (w), 1146 (m), 1044 (s), 993 (s), 855 (m), 827 (m), 746 (s), 687 (m), 606 (m).

HRMS (ESI/QTOF) m/z: $[M + H]^+$ Calcd for $C_{22}H_9F_5IO_4^+$ 558.9460; Found 558.9459.

Perfluorophenyl 3-((3-oxo-1$_{\lambda}^{3}$-benzo[d][1,2]iodaoxol-1(3H)-yl)ethynyl)benzoate (9b)

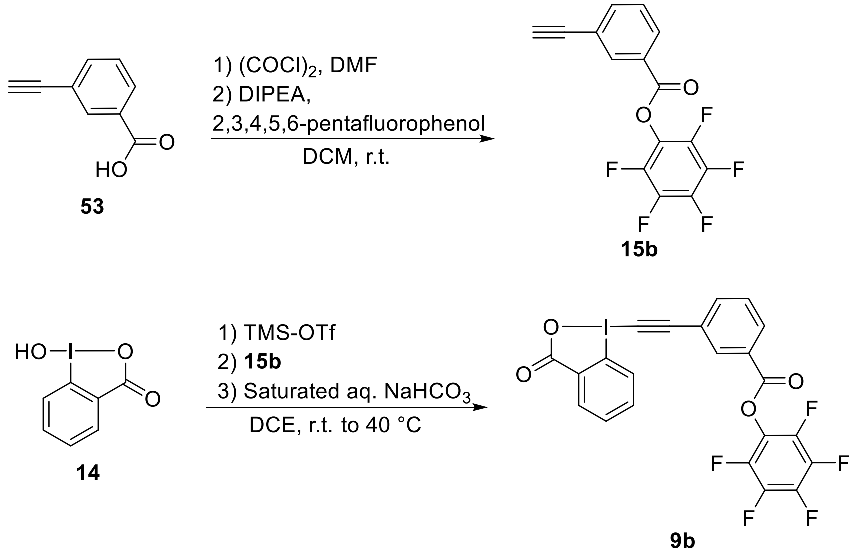

To a solution of 3-ethynylbenzoic acid (**53**) (300 mg, 2.05 mmol, 1.00 equiv.) in DCM (6.8 mL) oxalyl dichloride (0.220 mL, 2.57 mmol, 1.25 equiv.) and few drops of DMF were added. The mixture was stirred for 1 hour and concentrated to dryness. The obtained solid was re-dissolved in DCM (8.0 mL). To this solution N-ethyl-N-isopropylpropan-2-amine (0.400 mL, 2.06 mmol 1.10 equiv.) and 2,3,4,5,6-pentafluorophenol (397 mg, 2.16 mmol, 1.05 equiv.) were added. The mixture was stirred for 2.5 hours. The crude product was purified by flash chromatography (0-4% EtOAc/pentane) to afford perfluorophenyl 3-ethynylbenzoate (**15b**) (556 mg, 1.78 mmol, 87%) as white solid.

Rf= 0.5 (2% EtOAc in pentane).

^{1}H NMR ($CDCl_3$, 400 MHz): δ 8.32 (t, *J* = 1.7 Hz, 1H, Ar*H*), 8.17 (dt, *J* = 7.9, 1.5 Hz, 1H, Ar*H*), 7.80 (dt, *J* = 7.8, 1.4 Hz, 1H, Ar*H*), 7.53 (t, *J* = 7.8 Hz, 1H, Ar*H*), 3.18 (s, 1H, C*H*).

^{13}C NMR ($CDCl_3$, 101 MHz): δ 162.0 (*C*O_2), 141.6 (dm, *J* = 252.0 Hz, C_{Ar}-F), 139.8 (dm, *J* = 253.1 Hz, C_{Ar}-F) 138.0 (dm, *J* = 252.0 Hz, C_{Ar}-F) 138.1 (C_{Ar}), 134.4 (C_{Ar}), 130.9 (C_{Ar}), 129.2 (C_{Ar}), 127.5 (C_{Ar}), 125.5-125.1 (m, C_{Ar}-O), 123.5 (C_{Ar}), 82.0, 79.2.

m.p. (°C): 61-63.

IR (ν_{max}, cm^{-1}) 3302 (w), 2962 (w), 2925 (m), 2873 (w), 2854 (w), 1765 (m), 1756 (m), 1732 (w).

To a suspension of 2-iodosylbenzoic acid (**14**) (20 mg, 0.076 mmol, 1.0 equiv.) in DCE (0.23 mL), trimethylsilyl trifluoromethanesulfonate (0.015 mL, 0.076 mmol, 1.0 equiv.) was added. The mixture was stirred for 1 hour at room temperature, then perfluorophenyl 3-ethynylbenzoate (**15b**) (26 mg, 0.083 mmol, 1.1 equiv.) was added. The mixture was stirred for 24 hours (overnight) at 40 °C. The reaction was quenched with a saturated aq. $NaHCO_3$ solution. The two layers were separated and the aqueous layer was extracted with DCM. The combined organic layers were dried over $MgSO_4$, filtered and the solvent was removed under reduced pressure. The crude product was purified by flash chromatography (0-2% MeOH/DCM) to afford perfluorophenyl 3-((3-oxo-1$_{\lambda}^{3}$-benzo[d][1,2]iodaoxol-1(3H)-yl)ethynyl)benzoate (**9b**) (19 mg, 0.034 mmol, 45%) as white solid.

Rf = 0.3 (4% MeOH in DCM).

^{1}H NMR ($CDCl_3$, 400 MHz): δ 8.48 – 8.40 (m, 2H, C_{Ar}-*H*), 8.34 – 8.23 (m, 2H, C_{Ar}-*H*), 7.92 (d, *J* = 7.8 Hz, 1H, C_{Ar}-*H*), 7.86 – 7.75 (m, 2H, C_{Ar}-*H*), 7.66 (t, *J* = 7.9 Hz, 1H, C_{Ar}-*H*).

^{13}C NMR ($CDCl_3$, 101 MHz): δ 166.5 (*C*O_2), 161.5 (*C*O_2), 141.3 (dm, *J* = 249.0 Hz, C_{Ar}-F), , 139.7 (dm, *J* = 239.5 Hz, C_{Ar}-F), 138.4 (C_{Ar}), 138.0 (dm, *J* = 256.0 Hz, C_{Ar}-F), , 135.2 (C_{Ar}), 134.9 (C_{Ar}), 132.7 (C_{Ar}), 132.4 (C_{Ar}), 131.8 (C_{Ar}), 131.2 (C_{Ar}), 129.6 (C_{Ar}), 127.8 (C_{Ar}), 126.3 (C_{Ar}), 125.2 – 124.7 (m, C_{Ar}-O), 121.9 (C_{Ar}), 116.1 (C_{Ar}), 104.1 (*C*-I), 53.2 (*C*-CC_{Ar}).

Decomposition point. (°C): 180.5-183.2.

IR (v_{max}, cm^{-1}) 3079 (w), 2356 (w), 2335 (w), 2151 (w), 1760 (m), 1697 (w), 1681 (w), 1644 (s), 1620 (w), 1603 (w).

HRMS (ESI/QTOF) m/z: $[M + H]^+$ Calcd for $C_{22}H_9F_5IO_4^+$ 558.9460; Found 558.9474.

Methyl 2-((trimethylsilyl)ethynyl)benzoate (55)

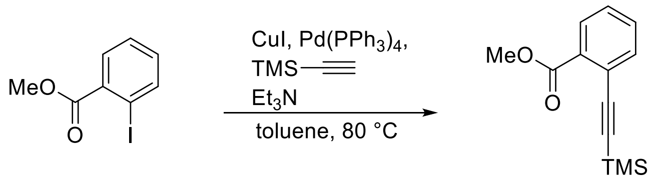

54 **55**

To a suspension of CuI (109 mg, 0.572 mmol, 10 mol%) and $Pd(PPh_3)_4$ (331 mg, 0.286 mmol, 5 mol%) in toluene (14.5 mL) was added methyl 2-iodobenzoate (**54**) (0.84 mL, 5.7 mmol, 1.0 equiv.), trimethylsilylacetylene (1.1 mL, 8.0 mmol, 1.4 equiv.) and Et_3N (2.8 mL, 20 mmol, 3.5 equiv.), at room temperature. The reaction mixture was heated to 80 °C and stirred for 12 hours. Afterwards, the reaction mixture was filtered through a pad of Celite® and washed with AcOEt. The organic layer was washed with a saturated NH_4Cl aqueous solution (50 mL x 3), dried ($MgSO_4$) and concentrated.
The crude product was purified by flash chromatography (0-5% AcOEt/pentane) to afford methyl 2-((trimethylsilyl)ethynyl)benzoate (**55**) as a colourless oil (1.30 g, 5.59 mmol, 98% yield).

^{1}H NMR ($CDCl_3$, 400 MHz): δ 7.93 – 7.87 (m, 1H, C_{Ar}-*H*), 7.61 – 7.55 (m, 1H, C_{Ar}-*H*), 7.44 (td, *J* = 7.6, 1.5 Hz, 1H, C_{Ar}-*H*), 7.36 (td, *J* = 7.6, 1.4 Hz, 1H, C_{Ar}-*H*), 3.92 (s, 3H, CH_3), 0.27 (s, 9H, SiCH_3).

Spectroscopic data was consistent with the values reported in literature.[8]

[8] K. Norseeda, N. Chaisan, C. Thongsornkleeb, J. Tummatorn, S. Ruchirawat *J. Org. Chem.* **2019**, 84, 16222-1623

2-Ethynylbenzoic acid (57)

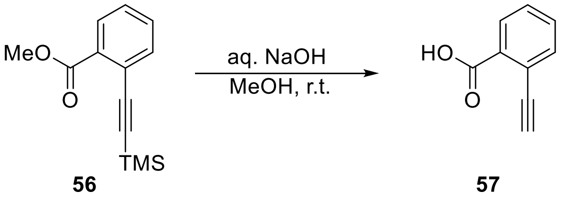

Following a reported procedure,[9] to a solution of the methyl 2-((trimethylsilyl)ethynyl)benzoate (**56**) (717 mg, 3.09 mmol, 1.00 equiv.) in MeOH (10 mL), 1 M aqueous solution of sodium hydroxide (30.9 mL, 30.9 mmol, 10.0 equiv.) was added. The mixture was stirred for 2 hours. The organic layer was removed and the aqueous layer was acidified with 1 M HCl to pH = 2.7. The aqueous solution was washed with diethyl ether (100 mL x 3). The combined organic layers were washed with brine, dried ($MgSO_4$) and concentrated. The obtained yellow solid was washed with pentane (x3), providing the desired 2-ethynylbenzoic acid **57** in quantitative yield as light-yellow solid with enough purity for further synthesis.

^{1}H NMR (MeOD, 400 MHz): δ 7.91 (dd, *J* = 7.8, 1.5 Hz, 1H, C_{Ar}-*H*), 7.61 (dd, *J* = 7.7, 1.4 Hz, 1H, C_{Ar}-*H*), 7.52 (td, *J* = 7.6, 1.5 Hz, 1H, C_{Ar}-*H*), 7.45 (td, *J* = 7.6, 1.5 Hz, 1H, C_{Ar}-*H*), 3.73 (s, 1H, CC-*H*).

Spectroscopic data was consistent with the values reported in literature.[9]

Perfluorophenyl 2-((3-oxo-1λ^3-benzo[d][1,2]iodaoxol-1(3H)-yl)ethynyl)benzoate (9c)

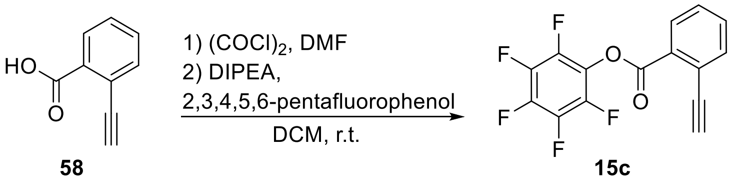

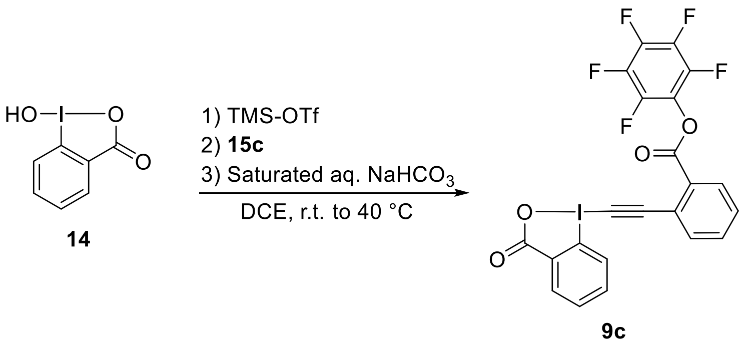

To a suspension of crude 2-ethynylbenzoic acid (**58**) (451 mg, 3.09 mmol, 1.00 equiv.) in DCM (10 mL) was added oxalyl dichloride (0.331 mL, 3.86 mmol, 1.25 equiv.) and DMF (0.24 mL, 3.1 mmol, 1.0 equiv.). The mixture was stirred vigorously for 1 hour and concentrated to dryness. The obtained solid was re-dissolved in DCM (14 mL). To this solution N-ethyl-N-isopropylpropan-2-

[9] A. S. K Hashmi, C Lothschütz., R. Döpp, M. Ackermann, J. De Buck Becker, M. Rudolph, C. Scholz, F. Romingera *Adv. Synth. Catal*. **2012**, *354*, 133 – 147.

amine (0.591 mL, 3.40 mmol, 1.10 equiv.) and 2,3,4,5,6-pentafluorophenol (597 mg, 3.24 mmol, 1.05 equiv.) were added. The mixture was stirred for 12 hours and additional N-ethyl-N-isopropylpropan-2-amine (0.40 mL, 2.3 mmol, 0.74 equiv.) was added to reach pH = 7. Purification by column chromatography (0-6% EtOAc/pentane) afforded perfluorophenyl 2-ethynylbenzoate **15c** as brown solid. The solid was washed with pentane and the filtrate was left to re-crystalize, the procedure was repeated two times yielding perfluorophenyl 2-ethynylbenzoate (**15c**) (508 mg, 1.63 mmol, 53%) as light pink solid.

Rf = 0.5 (0.5% EtOAc in pentane).

^{1}H NMR ($CDCl_3$, 400 MHz): δ 8.19 (dd, *J* = 7.8, 1.4 Hz, 1H, C_{Ar}-*H*), 7.73 (dd, *J* = 7.7, 1.4 Hz, 1H, C_{Ar}-*H*), 7.63 (td, *J* = 7.6, 1.4 Hz, 1H, C_{Ar}-*H*), 7.52 (td, *J* = 7.7, 1.4 Hz, 1H, C_{Ar}-*H*), 3.46 (s, 1H, CC-*H*).

^{13}C NMR ($CDCl_3$, 151 MHz): 161.7(CO_2), 141.6 (dm, *J* = 252.2 Hz, C_{Ar}-F), 139.8 (dm, *J* = 254.8 Hz, C_{Ar}-F), 138.0 (dm, *J* = 251.9 Hz, C_{Ar}-F), 135.5 (C_{Ar}), 133.7 (C_{Ar}), 131.6 (C_{Ar}), 129.0 (C_{Ar}), 129.0 (C_{Ar}), 125.6 – 124.9 (m, C_{Ar}-O), 124.3 (C_{Ar}), 84.1, 81.2.

m.p. (°C): 78.7-79.2.

IR (ν_{max}, cm^{-1}) 3277 (w), 3264 (w), 2962 (w), 1756 (s), 1718 (w).

HRMS not MS active.

To a suspension of 2-iodosylbenzoic acid (**14**) (418 mg, 1.58 mmol, 1.00 equiv.) in DCE (16 mL) was added trimethylsilyl trifluoromethanesulfonate (287 µL, 1.58 mmol, 1.00 equiv.) under nitrogen atmosphere at room temperature. The mixture was vortexed and stirred for 1 hour. Afterwards, perfluorophenyl 2-ethynylbenzoate (**15c**) (544 mg, 1.74 mmol, 1.10 equiv.) was added under nitrogen atmosphere and the reaction was heated to 40 °C and stirred for 48 hours. The reaction was quenched with saturated aq. $NaHCO_3$ solution (20 mL) and stirred for 3 hours. The two layers were separated and the aqueous layer was extracted with DCM (2 x 50 mL). The combined organic layers were dried over $MgSO_4$, filtered and the solvent was removed under reduced pressure. Purification by column chromatography (0-6% MeOH/DCM/) afforded the desired product perfluorophenyl 2-((3-oxo-1λ^3-benzo[d][1,2]iodaoxol-1(3H)-yl)ethynyl)benzoate (**9c**) as a brown solid (139 mg, 249 µmol, 16% yield).

Rf = 0.2 (4% MeOH in DCM).

^{1}H NMR ($CDCl_3$, 400 MHz): δ 8.46 (dd, *J* = 7.9, 1.3 Hz, 1H, C_{Ar}-*H*), 8.39 (dd, *J* = 7.2, 2.0 Hz, 1H, C_{Ar}-*H*), 8.33 (dd, *J* = 8.1, 1.3 Hz, 1H, C_{Ar}-*H*), 7.82 (dd, *J* = 7.7, 1.4 Hz, 1H, C_{Ar}-*H*), 7.80 – 7.66 (m, 3H, C_{Ar}-*H*), 7.66 (td, *J* = 7.7, 1.5 Hz, 1H, C_{Ar}-*H*).

^{13}C NMR ($CDCl_3$, 101 MHz): δ 166.7(CO_2), 161.1(CO_2), 141.4 (dm, *J* = 251.6 Hz, C_{Ar}-F), 139.8 (dm, *J* = 245.3 Hz, C_{Ar}-F), 138.1 (dm, *J* = 252.2 Hz, C_{Ar}-F), , 135.9 (C_{Ar}), 135.2 (C_{Ar}), 134.2 (C_{Ar}), 132.6 (C_{Ar}), 132.0 (C_{Ar}), 131.8 (C_{Ar}), 131.2 (C_{Ar}), 130.5 (C_{Ar}), 128.9 (C_{Ar}), 127.1 (C_{Ar}), 125.2 – 124.8 (m, C_{Ar}-O), 123.0 (C_{Ar}), 116.6 (C_{Ar}), 103.3 (*C*-I), 57.7 (C*C*-C_{Ar}).

Decomposition point (°C): 176.1-177.6.

IR (ν_{max}, cm^{-1}) 2989 (w), 2961 (w), 2902 (w), 2361 (w), 2154 (w), 1766 (w), 1754 (m), 1640 (m), 1621 (m).

HRMS (ESI/QTOF) m/z: $[M + H]^+$ Calcd for $C_{22}H_9F_5IO_4^+$ 558.9460; Found 558.9466.

Bis(perfluorophenyl) 5-((3-oxo-1λ3-benzo[d][1,2]iodaoxol-1(3H)-yl)ethynyl)isophthalate (9d)

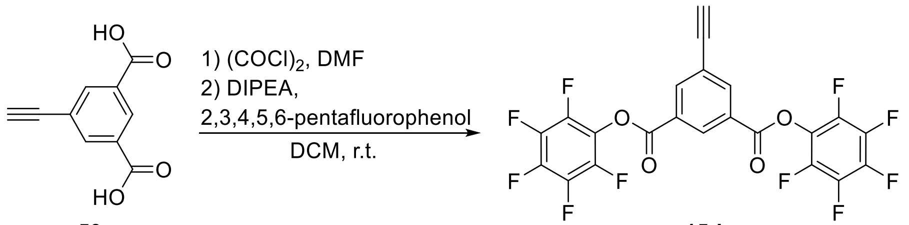

59 **15d**

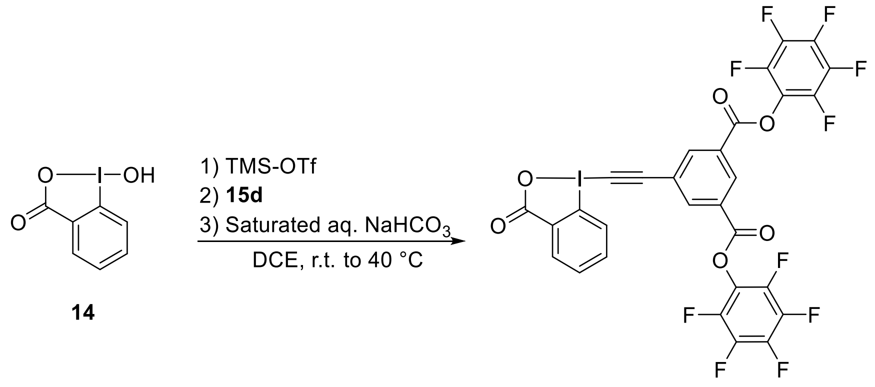

14 **9d**

To a suspension of 5-ethynylisophthalic acid (**59**) (100 mg, 0.526 mmol, 1.00 equiv.) in DCM (1.8 mL) oxalyl dichloride (0.113 mL, 1.32 mmol, 2.50 equiv.) and few drops of DMF were added. The mixture was stirred vigorously for 2 hours and concentrated to dryness. The obtained solid was re-dissolved in DCM (1.8 mL). To this solution N-ethyl-N-isopropylpropan-2-amine (0.201 mL, 1.15 mmol 1.10 equiv.) and 2,3,4,5,6-pentafluorophenol (203 mg, 1.10 mmol, 2.10 equiv.) were added. The mixture was stirred for 64 hours. The crude product was purified by flash chromatography (0-3% EtOAc/pentane) to afford bis(perfluorophenyl) 5-ethynylisophthalate (**15d**) (145 mg, 0.278 mmol, 53%) as white solid.

Rf = 0.3 (2% EtOAc in pentane).

^{1}H NMR ($CDCl_3$, 400 MHz): δ 8.96 – 8.92 (m, 1H, C_{Ar}-*H*), 8.62 – 8.56 (m, 2H, C_{Ar}-*H*), 3.31 (s, 1H, C-*H*).

^{13}C NMR ($CDCl_3$, 101 MHz): δ 160.9 (*C*O_2), 141.4 (dm, *J* = 256.2 Hz, C_{Ar}-F), 140.1 (dm, *J* = 254.7 Hz, C_{Ar}-F), 139.7 – 136.5 (m, C_{Ar}, C_{Ar}-F), 132.5 (C_{Ar}), 128.7(C_{Ar}), 125.4 – 124.6 (m, C_{Ar}, C_{Ar}-O), 81.2, 80.5.

m.p. (°C): 139.6 - 140.2.

IR (ν_{max}, cm^{-1}) 3266 (w), 1786 (w), 1781 (w), 1766 (w), 1756 (w), 1744 (w).

To a suspension of 2-iodosylbenzoic acid (**14**) (39.5 mg, 0.150 mmol, 1.00 equiv.) in DCE (0.45 mL) trimethylsilyl trifluoromethanesulfonate (27.1 µL, 0.150 mmol, 1.00 equiv.) was added at room

temperature. The mixture was vortexed and stirred for 1 hour. Afterwards, bis(perfluorophenyl) 5-ethynylisophthalate (**15d**) (86.0 mg, 0.170 mmol, 1.05 equiv.) was added and the reaction was heated to 40 °C and for 16 hours. The reaction was quenched with a saturated aq. $NaHCO_3$ solution (5 mL) and stirred for 2 hours. The two layers were separated and the aqueous layer was extracted with DCM (2 x 20 mL). The combined organic layers were dried over $MgSO_4$, filtered and the solvent was removed under reduced pressure. The crude product was purified by flash chromatography (0-4% MeOH/DCM) to afford the desired product bis(perfluorophenyl) 5-((3-oxo-1λ^3-benzo[d][1,2]iodaoxol-1(3H)-yl)ethynyl)isophthalate (**9d**) as a white solid (30.5 mg, 40.0 μmol, 27% yield).

Rf = 0.3 (4% MeOH in DCM).

^{1}H NMR ($CDCl_3$, 400 MHz): δ 9.05 (t, *J* = 1.7 Hz, 1H, C_{Ar}-*H*), 8.70 (d, *J* = 1.7 Hz, 2H, C_{Ar}-*H*), 8.45 (dd, *J* = 6.9, 2.2 Hz, 1H, C_{Ar}-*H*), 8.30 – 8.23 (m, 1H, C_{Ar}-*H*), 7.89 – 7.77 (m, 2H, C_{Ar}-*H*).

^{13}C NMR ($CDCl_3$, 151 MHz): δ 166.9 (*C*O_2), 160.6 (*C*O_2), 141.3 (dm, *J* = 253.1 Hz, C_{Ar}-F), 140.1 (dm, *J* = 254.7 Hz, C_{Ar}-F), 139.8 (C_{Ar}), 138.2 (dm, *J* = 254.0 Hz, C_{Ar}-F), 135.5 (C_{Ar}), 133.8 (C_{Ar}), 132.9 (C_{Ar}), 132.1 (C_{Ar}), 131.2 (C_{Ar}), 129.1 (C_{Ar}), 126.6 (C_{Ar}), 125.0 – 124.6 (m, C_{Ar}-O), 123.5 (C_{Ar}), 116.2 (C_{Ar}), 102.0 (C-I), 56.2 (C*C*-C_{Ar}).

Decomposition point (°C): 197.2-198.5.

IR (ν_{max}, cm^{-1}) 3094 (w), 3063 (w), 2955 (w), 2921 (w), 2357 (m), 2332 (m), 1760 (m), 1716 (w), 1699 (w), 1684 (w), 1650 (w), 1634 (w), 1621 (w).

HRMS (ESI/QTOF) m/z: $[M + H]^+$ Calcd for $C_{29}H_8F_{10}IO_6^+$ 768.9200; Found 768.9200.

5. Small molecule model

(4R,14R)-Methyl 14-acetamido-9,9-diisopropyl-4-(methoxycarbonyl)-2-oxo-6,12-dithia-3-aza-9-silapentadeca-7,10-diyn-15-oate (13)

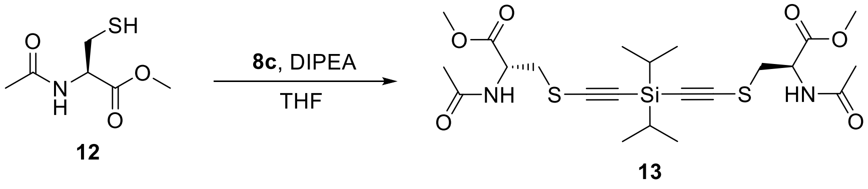

To a solution of bis((3,3-bis(trifluoromethyl)-1l3-benzo[d][1,2]iodaoxol-1(3H)-yl)ethynyl)diisopropylsilane (**8c**) (122 mg, 0.136 mmol, 1.00 equiv.) in THF (1.9 mL), N-ethyl-N-isopropylpropan-2-amine (11.8 µL, 0.073 mmol, 0.5 equiv.) followed by methyl acetyl *L*-cysteinate (**12**) (48 mg, 0.027 mmol, 2.0 equiv.) was added at room temperature and the mixture was stirred for 12 h. Afterwards, additional methyl acetyl *L*-cysteinate (**12**) (24 mg, 0.014 mmol, 1.0 equiv.) and N-ethyl-N-isopropylpropan-2-amine (12 µL, 0.073 mmol, 0.50 equiv.) were added. After 2 hours the crude reaction mixture was purified using reverse phase preparative liquid chromatography (0-100% AcCN/H_20) to afford (4R,14R)-methyl 14-acetamido-9,9-diisopropyl-4-(methoxycarbonyl)-2-oxo-6,12-dithia-3-aza-9-silapentadeca-7,10-diyn-15-oate (**13**) (45 mg, 0.086, 63%) as light-yellow viscous oil.

^{1}H NMR (400 MHz, $CDCl_3$) δ 6.61 (d, *J* = 7.6 Hz, 2H, N*H*), 4.95 (dt, *J* = 7.5, 4.7 Hz, 2H, C*H*(N)), 3.81 (s, 6H, C*H*$_3$O), 3.33 (dd, *J* = 13.9, 4.4 Hz, 2H, *H*CH(S)), 3.21 (dd, *J* = 13.8, 5.1 Hz, 2H, HC*H*(S)), 2.08 (s, 6H, CO(C*H*$_3$)), 1.13 – 0.92 (m, 14H, C*H*(CH_3)$_2$ and CH(C*H*$_3$)$_2$).

^{13}C NMR (101 MHz, $CDCl_3$) δ 170.4 (*C*O), 170.2 (*C*O), 96.4 (*C*Si), 94.6 (*C*S), 53.1, 52.5, 37.8, 23.2, 17.7 (CH(*C*H$_3$)$_2$), 12.6.

HRMS (nanochip-ESI/LTQ-Orbitrap) m/z: $[M + Na]^+$ Calcd for $C_{22}H_{34}N_2NaO_6S_2Si^+$ 537.1520; Found 537.1509.

IR (ν_{max}, cm^{-1}) 2953 (w), 2865 (w), 2093 (m), 1745 (m), 1663 (m).

6. Crystal structure

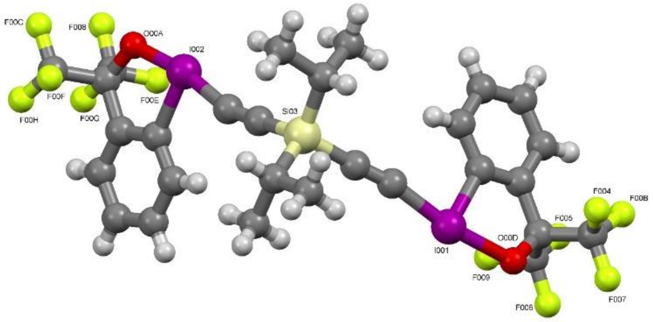

X-ray of single crystal of **8c**. Supplementary crystallographic data for this compound have been deposited at Cambridge Crystallographic Data Centre (CCDC 2040767) and can be obtained free of charge via www.ccdc.cam.ac.uk/data_request/cif.

7. Peptide model Calibration

Peptide used for the calibration: Ac-ENPECILDCHVQRVM-NH_2

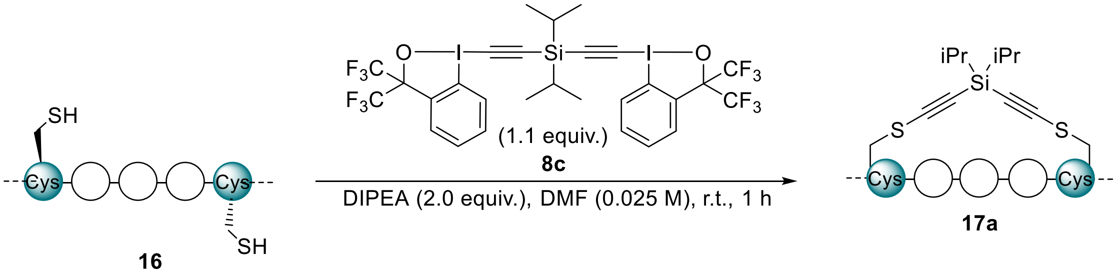

Sequence: Ac-ENPECILDCHVQRVM-NH_2

To a solution of Ac-ENPECILDCHVQRVM-NH_2 (**16**) (28.8 mg, 0.0150 mmol, 1.00 equiv.) in DMF (0.6 mL, 0.025M), DIPEA (5.2 µL, 0.030 mmol, 2.0 equiv.) was added. After 5 minutes, bis((3,3-bis(trifluoromethyl)-1l3-benzo[d][1,2]iodaoxol-1(3H)-yl)ethynyl)diisopropylsilane (**8c**) (14.9 mg, 0.0170 mmol, 1.10 equiv.) was added and the solution was allowed to stir at room temperature for 1 hour. The solution was then directly injected into a preparative RP-HPLC (following method 1) and lyophilized to afford the stapled product (**17a**) (10.2 mg, 33%). A 2 mM solution of the stapled peptide was prepared and the aliquot for the calibration were prepared by dilution (see table below).

HRMS (nanochip-ESI/LTQ-Orbitrap) m/z: $[M + H_2]^{+2}$ Calcd for $C_{85}H_{137}N_{23}O_{24}S_3Si^{+2}$ 993.9564; Found 993.9577.

MS/MS Characterization:

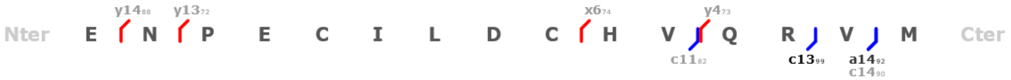

a14 (17.3), ***a14*** (35.9), ***c11*** (2.0), ***c13*** (35.8), ***c14*** (100.6), ***c14*** (1.0), ***x6*** (78.8), **y4** (1.3), ***y13*** (2.2), ***y14*** (0.9).

Absorbance (mAU) versus concentration (mM) of the stapled peptide (**17a**).

Conc. (mM)	Absorbance (mAU)
1	3677.3
0.8	3318
0.6	2615.5
0.4	1661.7
0.2	973.1
0.1	412.5

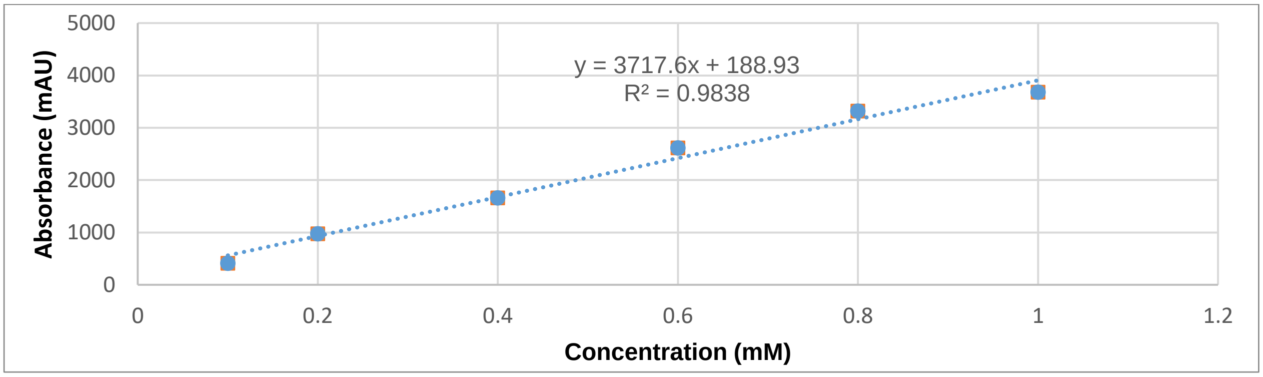

Figure S1: Linear equation of the absorbance (mAU) versus concentration (mM) of the stapled peptide (**17a**).

The equation of the trendline was used to calculate the yield based on absorbance of the reaction with Ac-ENPECILDCHVQRVM-NH_2 as substrate.

HPLC-UV Chromatograms (210 nm):

Retention time: 9.7 min

0.1 mM:

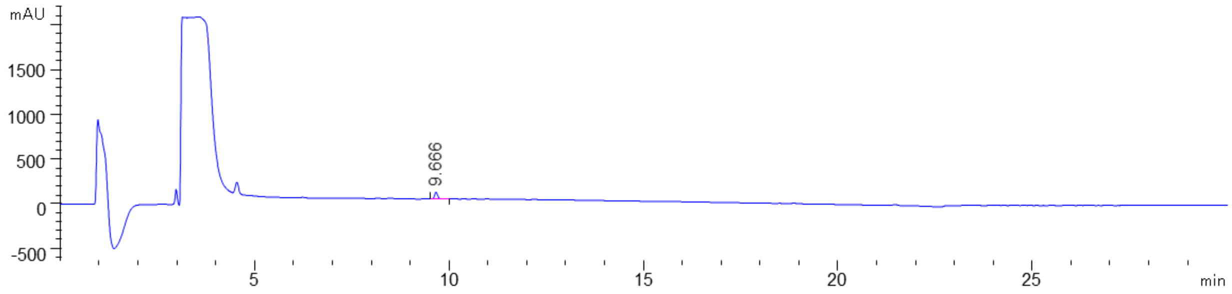

0.2 mM:

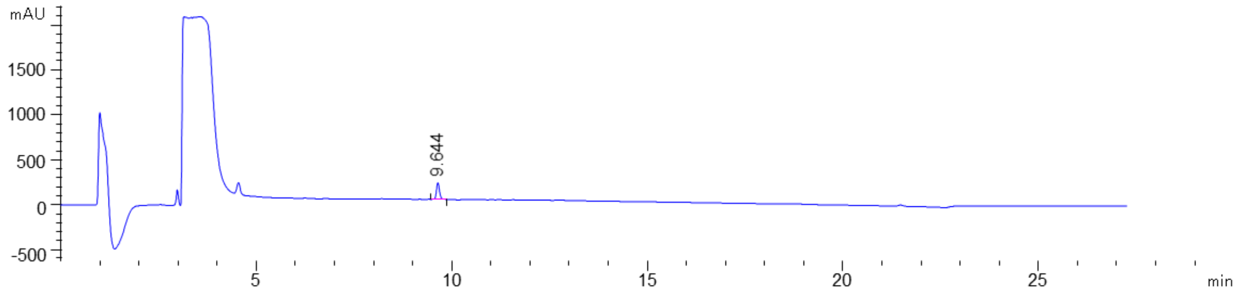

0.4 mM:

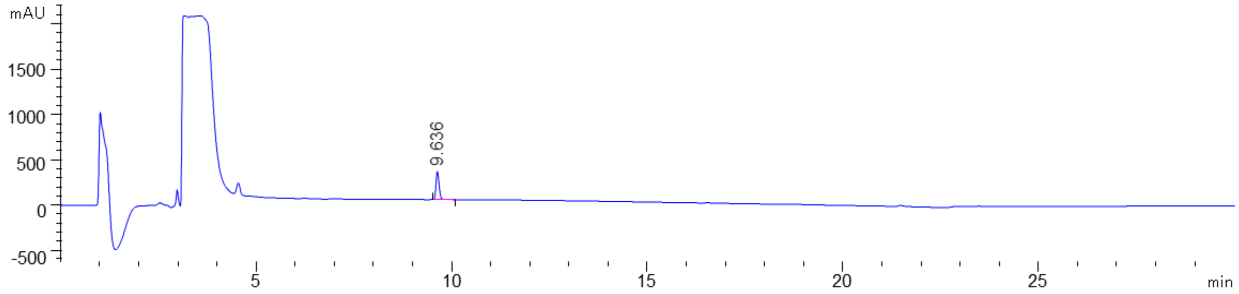

0.6 mM:

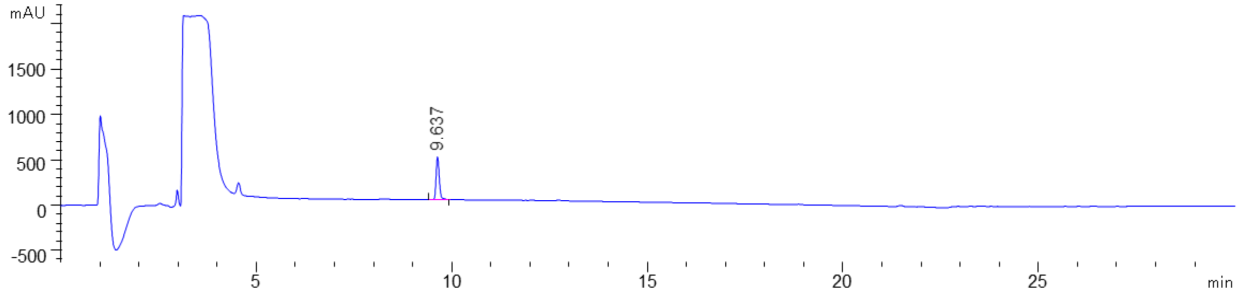

0.8 mM:

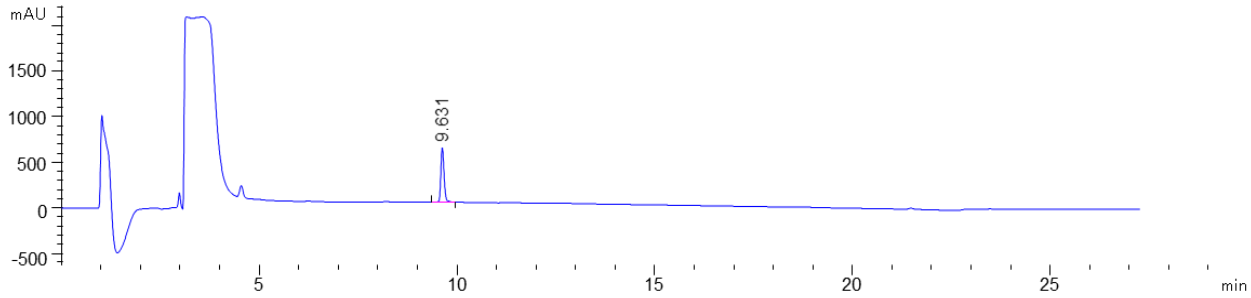

1.0 mM:

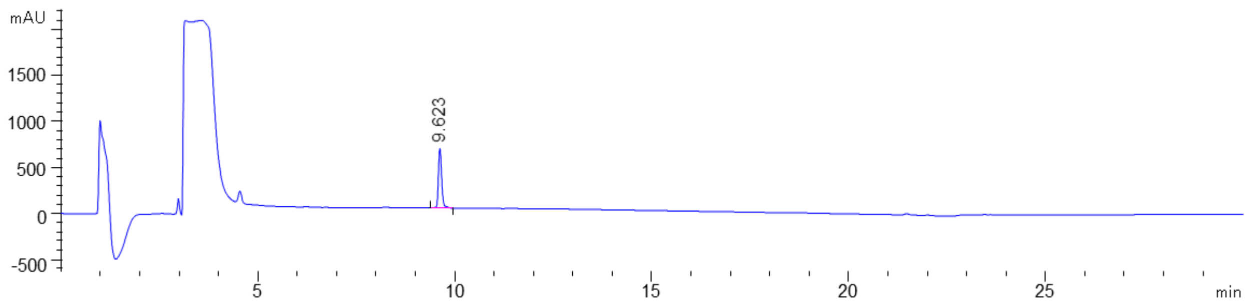

8. Cysteine-cysteine reaction optimization

a. Base optimization

General Procedure for the base optimization: Ac-ENPECILDCHVQRVM-NH_2 (**16**) (0.50 to 3.0 mg, 0.24 to 1.5 µmol, 1.0 equiv.) was weighed in a 1.5 mL Eppendorf tube. DMF that had been bubbled with nitrogen for 30 minutes was used to prepare a 1 mM solution. To 91 µL of the peptide solution, 1.0 µL of a 0.2M solution of DIPEA (2.2 equiv.) in DMF and then 1.0 µL of a 0.1M solution of bis((3,3-bis(trifluoromethyl)-1l3-benzo[d][1,2]iodaoxol-1(3H)-yl)ethynyl)diisopropylsilane (**8c**) (1.1 equiv.) in DMF were added. The mixture was vortexed for a few seconds and shaken at room temperature for 30 minutes and analyzed by HPLC (Method **1**).

Table S1: Yield (%) for a series of bases, based on the model calibration (see Section 7: Peptide model Calibration)

Base	Absorbance (mAU)	Yield (%)
DIPEA	1815	45
DBU	168	0
TMG	237	1
Morpholine	856	18
Pyridine	1617	39
2,6-Lutidine	1897	47
2,2,6,6-Tetramethylpiperidine	1362	32
Quinoline	1896	47
NMM	1923	48

Based on the similarity of the results for NMM, Quinoline, 2,6-Lutidine and DIPEA, DIPEA was selected as the base of choice for the stapling reactions.

HPLC-UV Chromatograms (210 nm):

Retention time product: 9.7 min

Retention time reduced Iodocompound byproduct (**46**): 17.5 min

DIPEA:

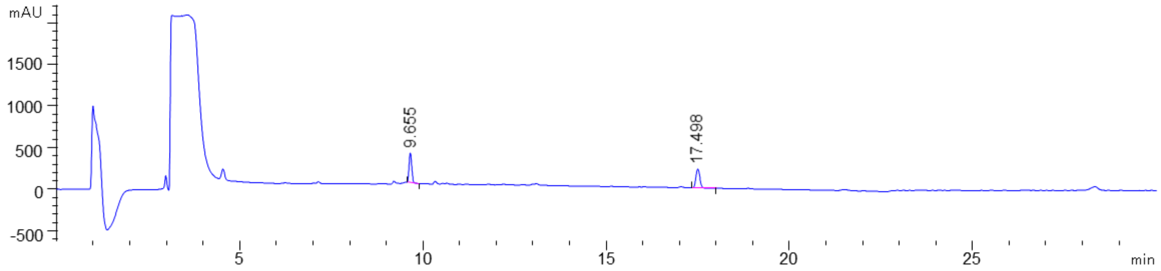

DBU:

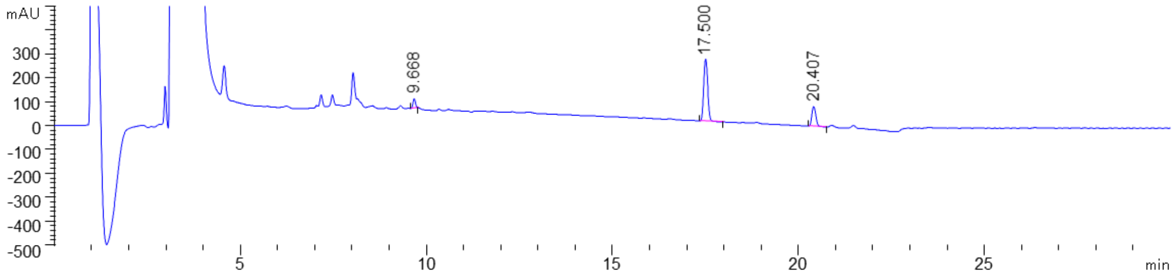

TMG:

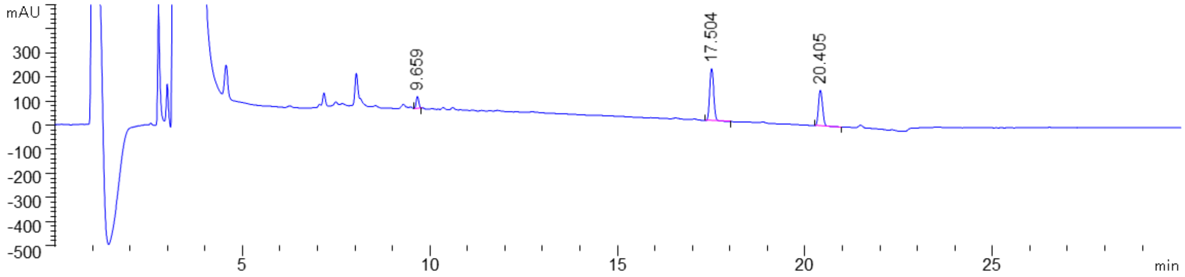

Morpholine:

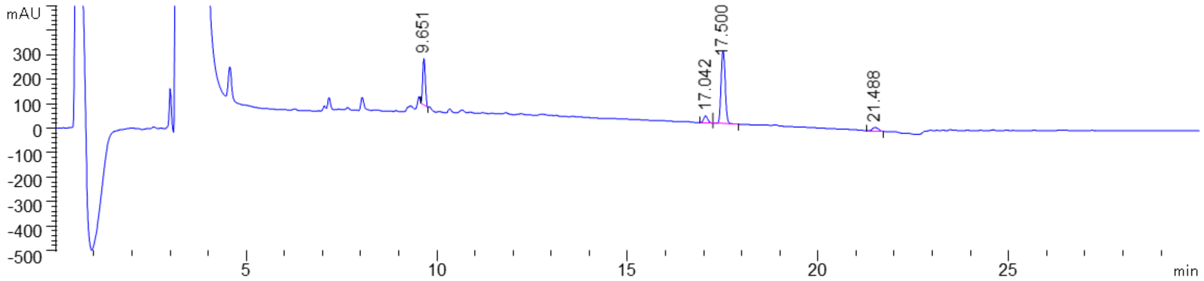

Pyridine:

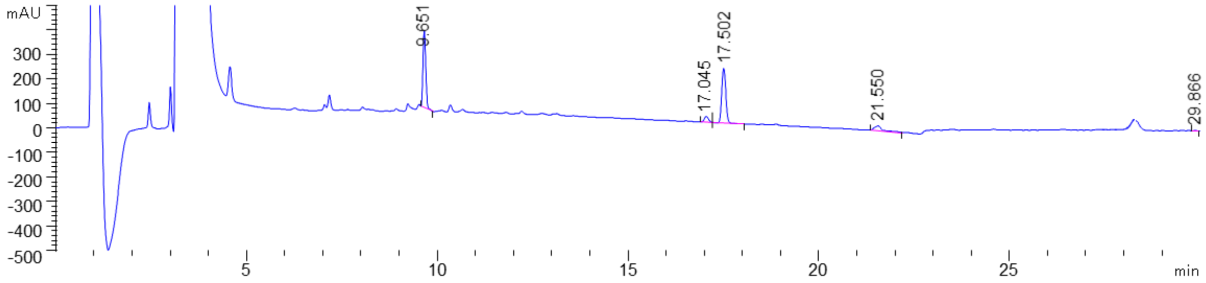

2,6-Lutidine:

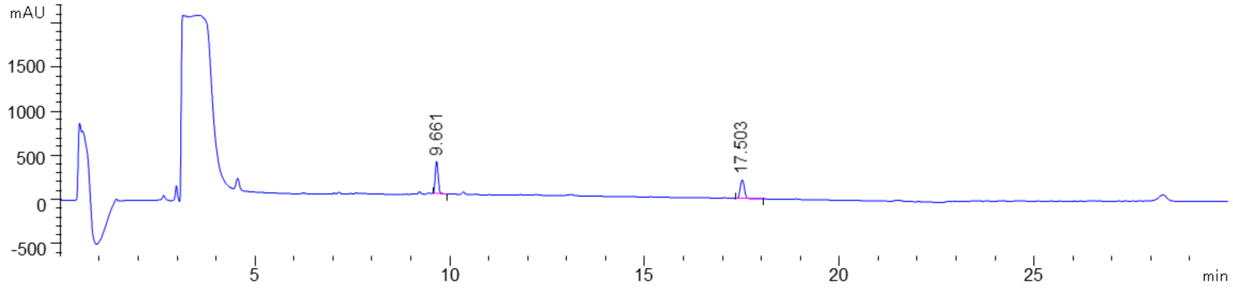

2,2,6,6-Tetramethylpiperidine:

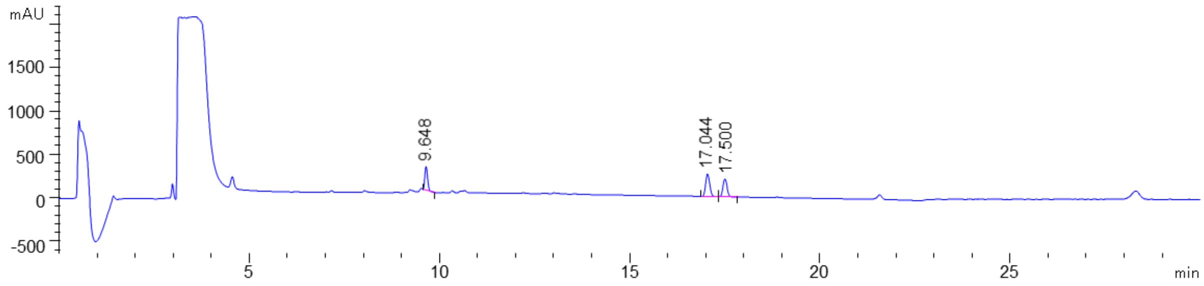

Quinoline:

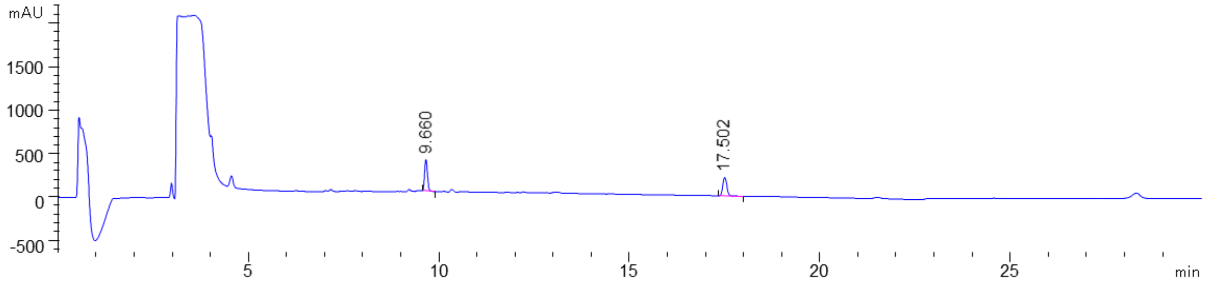

NMM:

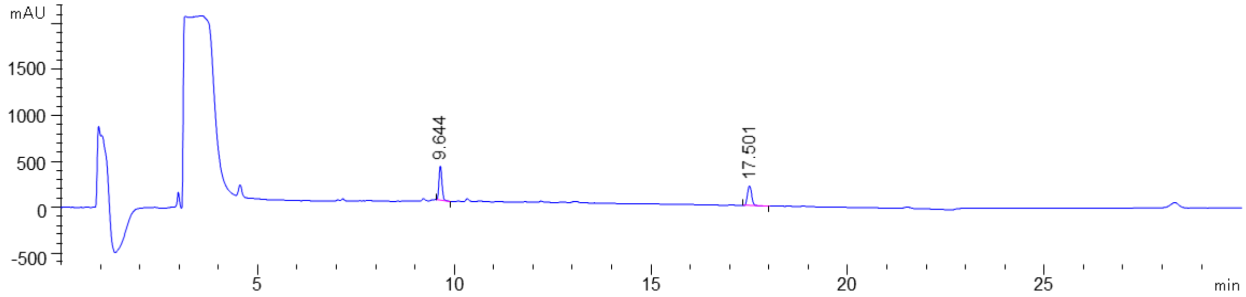

b. Base loading optimization

General Procedure for the base loading optimization: Ac-ENPECILDCHVQRVM-NH_2 (**16**) (0.50 to 3.0 mg, 0.24 to 1.5 µmol, 1.0 equiv.) was weighed in a 1.5 mL Eppendorf tube. DMF that had been bubbled with nitrogen for 30 minutes was used to prepare a 1 mM solution. To 91 µL of

the peptide solution, 1.0 µL of a 0.002, 0.02, 0.1 or 0.2 M solution of DIPEA (2.2 equiv.) in DMF and then 1.0 µL of a 0.1M solution of bis((3,3-bis(trifluoromethyl)-1l3-benzo[d][1,2]iodaoxol-1(3H)-yl)ethynyl)diisopropylsilane (**8c**) (1.1 equiv.) in DMF were added. The mixture was vortexed for a few seconds and shaken at room temperature for 30 minutes and analyzed by HPLC (Method **1**).

Table S2: Yield (%) for bases loading optimization, based on the model calibration (see Section 7: Peptide model Calibration)

Base loading (equiv.)	Absorbance (mAu)	Yield (%)
0	691	14
0.02	1020	23
0.22	1789	44
1.1	1722	42
2.2	1778	44

HPLC-UV Chromatograms (210 nm):

Retention time stapled product: 9.7 min

Retention time starting peptide: 7.6 min

Retention time reduced Iodocompound byproduct [(1,1,1,3,3,3-hexafluoro-2-(2-iodophenyl)propan-2-ol] (**46**): 17.5 min

0.0 equivalents:

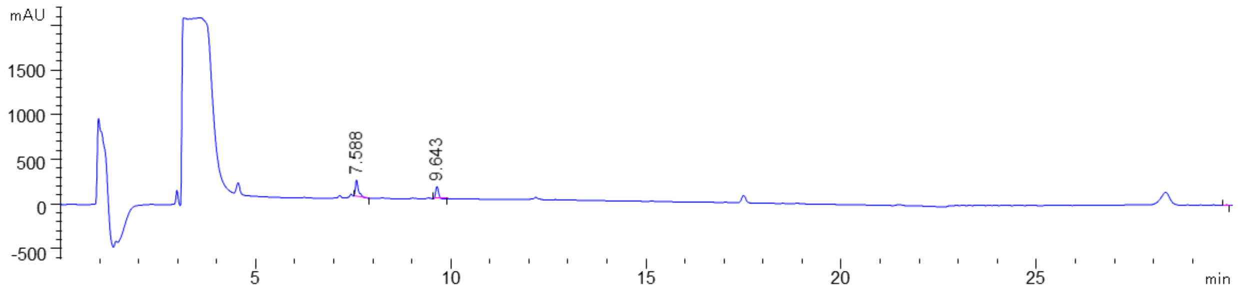

0.02 equivalents:

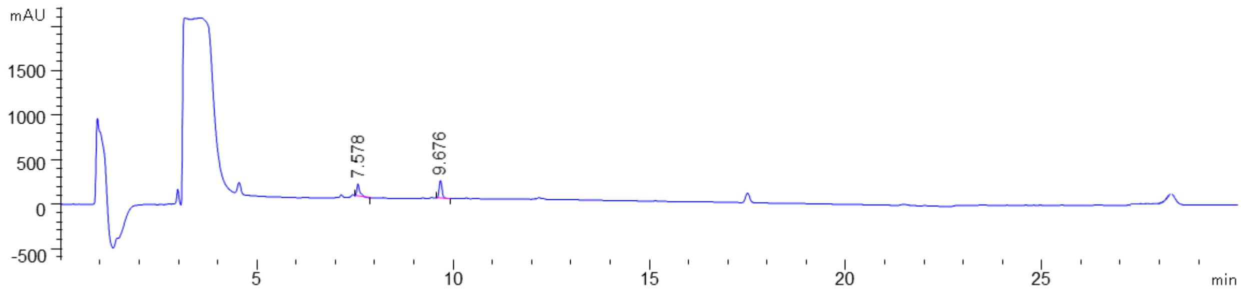

0.22 equivalents:

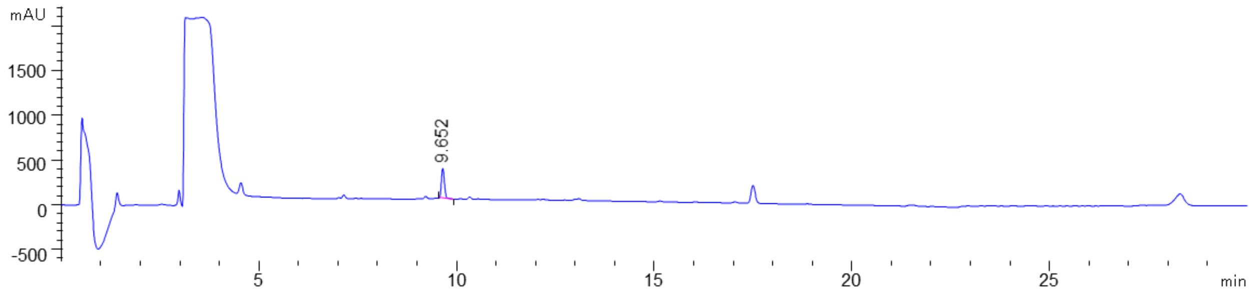

1.1 equivalents:

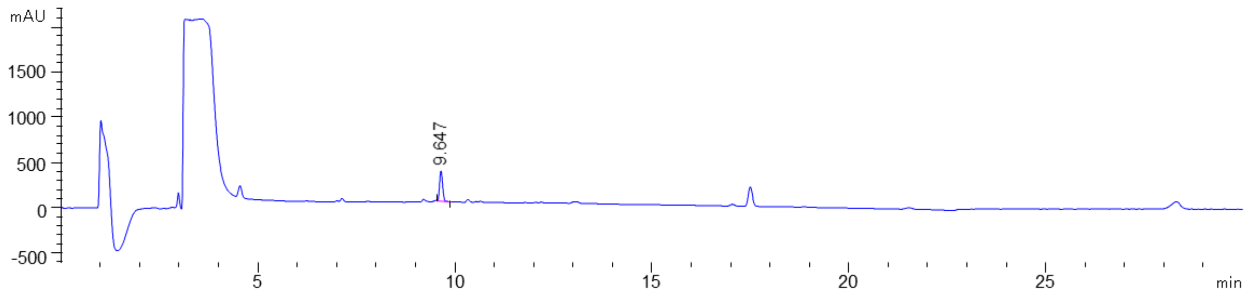

2.2 equivalents:

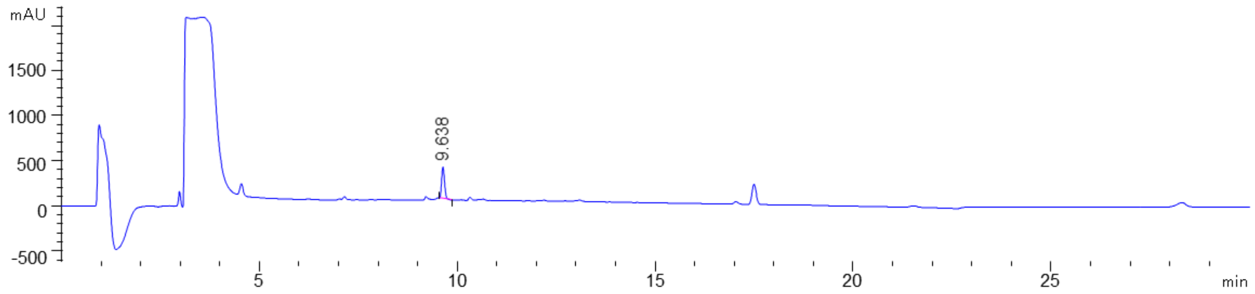

c. Reagent loading

General Procedure for the reagent loading optimization: Ac-ENPECILDCHVQRVM-NH_2 (**16**) (0.50 to 3.0 mg, 0.24 to 1.5 µmol, 1.0 equiv.) was weighed in a 1.5 mL Eppendorf tube. DMF that had been bubbled with nitrogen for 30 minutes was used to prepare a 1 mM solution. The solution was split in two fractions of the same volume. To both peptide solutions, DIPEA in DMF (0.2M, 2.5 equiv.) and then bis((3,3-bis(trifluoromethyl)-1l3-benzo[d][1,2]iodaoxol-1(3H)-yl)ethynyl)diisopropylsilane (**8c**) in DMF (0.1 M, 1.0-5.0 equiv.) were added. The mixture was vortexed for a few seconds and shaken at room temperature for 240 minutes and analyzed by HPLC (Method **2**).

Table S3: Reagent loading optimization. Yields are based on the model calibration (see Section 7: Peptide model Calibration)

Reagent loading	Abs. 1 (mAu)	Abs. 2 (mAu)	Average Yield (%)
1.0	2175	2162	55
1.5	2595	2607	67
3.0	2805	2700	72
5.0	2700	2794	73

HPLC-UV Chromatograms (210 nm) (only one of the duplicates is shown):

Retention time stapled product: 15.1 min

Retention time starting peptide: 13.2 min

Retention time reduced Iodocompound byproduct (**46**): 22.2 min

1.0 equivalents:

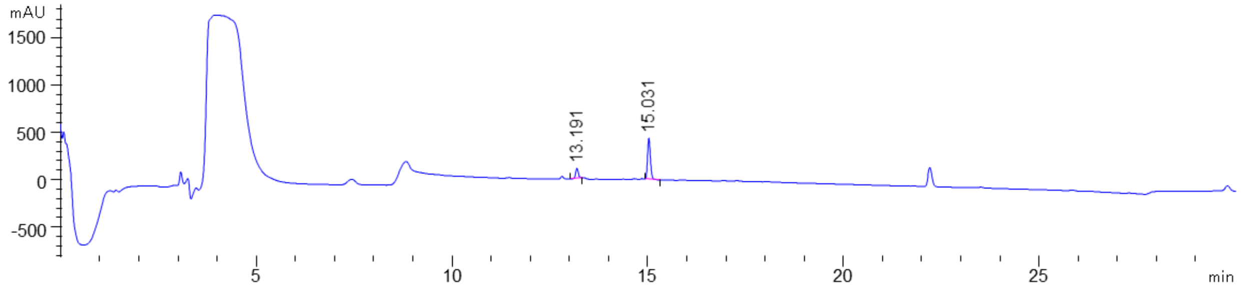

1.5 equivalents:

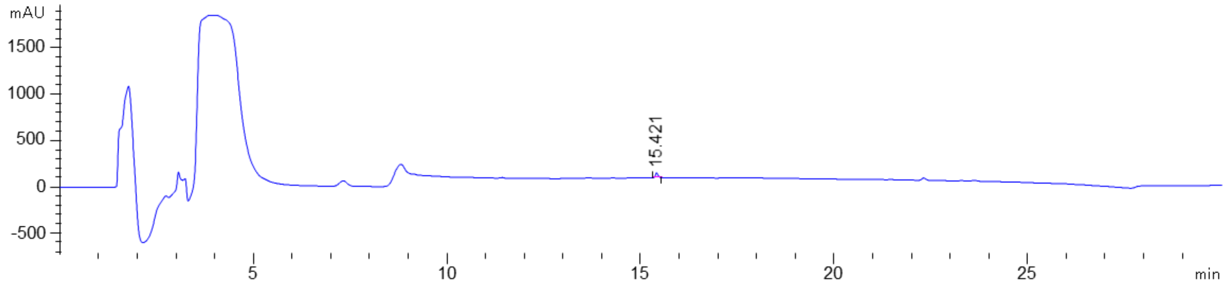

3.0 equivalents:

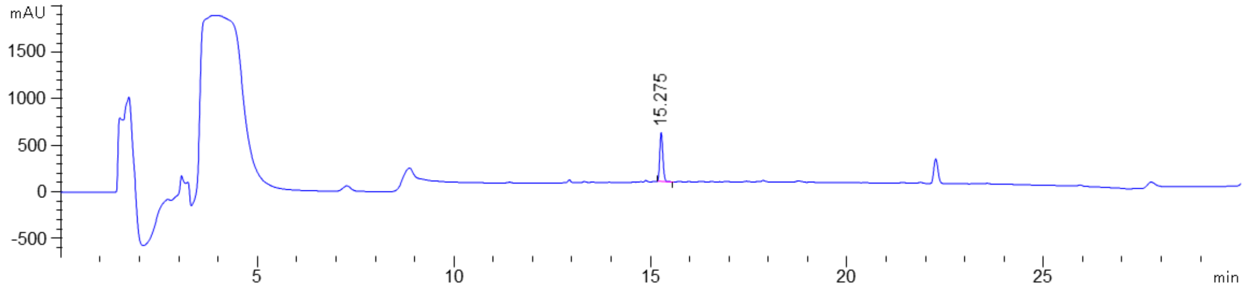

5.0 equivalents:

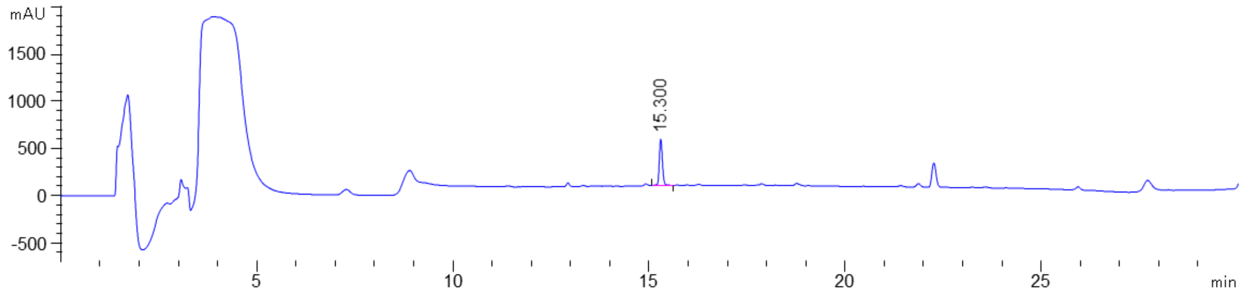

d. Temperature

Ac-ENPECILDCHVQRVM-NH_2 (**16**) (0.60 mg, 0.29 µmol, 2.0 equiv.) was weighed in a 1.5 mL Eppendorf tube. DMF (302 µL, 1.0 mM), that had been bubbled with nitrogen for 30 minutes, was used to prepare a 1 mM solution. The solution was split in two fractions of the same volume (146 µL). To both peptide solutions, DIPEA in DMF (0.2M, 1.8 µL, 2.5 equiv.) and then bis((3,3-bis(trifluoromethyl)-1l3-benzo[d][1,2]iodaoxol-1(3H)-yl)ethynyl)diisopropylsilane (**8c**) in DMF (0.1M, 4.4 µL, 3.0 equiv.) were added. The mixture was vortexed for a few seconds and shaken at 37°C for 240 minutes and analyzed by HPLC (Method **2**).

Temperature	Abs. 1 (mAu)	Abs. 2 (mAu)	Averaged Yield (%)
37 °C	2701	2794	78

HPLC-UV Chromatograms (210 nm) (only one of the duplicates is shown):

Retention time stapled product: 15.1 min

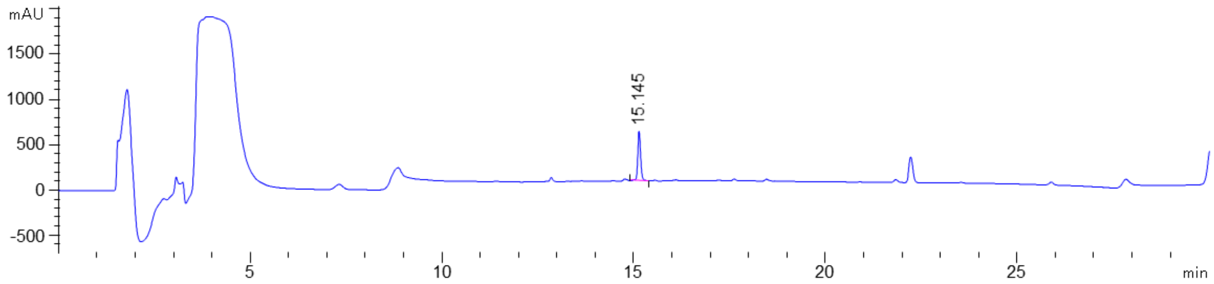

e. Concentration

Ac-ENPECILDCHVQRVM-NH_2 (**16**) (0.64 mg, 0.31 µmol, 2.0 equiv.) was weighed in a 1.5 mL Eppendorf. DMF (49.0 µL, 6.4 mM), that had been bubbled with nitrogen for 30 minutes, was used to prepare a 6.4 mM solution. The solution was split in two fractions of the same volume (24.5 µL). To both peptide solutions, DIPEA in DMF (0.2M, 1.9 µL, 2.5 equiv.) and then bis((3,3-bis(trifluoromethyl)-1l3-benzo[d][1,2]iodaoxol-1(3H)-yl)ethynyl)diisopropylsilane (**8c**) in DMF (0.1M, 4.7 µL, 3.0 equiv.) were added. The mixture was vortexed for a few seconds and shaken at 37°C for 30 minutes and analysed by HPLC (Method **2**).

Concentration (mM)	Abs. 1 (mAu)	Abs. 2 (mAu)	Averaged Yield (%)
5.0	1289	1209	66

HPLC-UV Chromatograms (210 nm) (only one of the duplicates is shown):

Retention time stapled product: 15.1 min

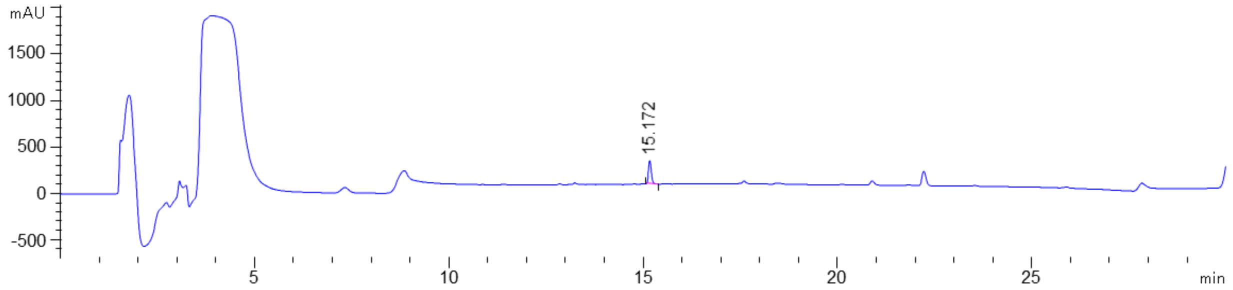

f. Solvent

Procedure A for the solvent optimization: Ac-ENPECILDCHVQRVM-NH_2 (**16**) (0.50 to 3.0 mg, 0.24 to 1.5 µmol, 2.0 equiv.) was weighed in a 1.5 mL Eppendorf tube. THF that had been bubbled with nitrogen for 30 minutes was used to prepare a 1 mM solution. The solution was split in two fractions of the same volume. To both peptide solutions, DIPEA in DMF (0.2M, 2.5 equiv.) and then bis((3,3-bis(trifluoromethyl)-1l3-benzo[d][1,2]iodaoxol-1(3H)-yl)ethynyl)diisopropylsilane (**8c**) in DMF (0.1 M, 1.0-5.0 equiv.) were added. The mixture was vortexed for a few seconds and shaken at 37 °C for 240 minutes and analyzed by HPLC (Method **2**).

Procedure B for the solvent optimization: Ac-ENPECILDCHVQRVM-NH_2 (**16**) (0.50 to 3.0 mg, 0.24 to 1.5 µmol, 2.0 equiv.) was weighed in a 1.5 mL Eppendorf tube. DMF that had been bubbled with nitrogen for 30 minutes was used to prepare a 10 mM solution. The solution was split in two fractions of the same volume and THF (bubbled with nitrogen) was added to yield a 1 mM final concentration. To both peptide solutions, DIPEA in DMF (0.2M, 2.5 equiv.) and then bis((3,3-bis(trifluoromethyl)-1l3-benzo[d][1,2]iodaoxol-1(3H)-yl)ethynyl)diisopropylsilane (**8c**) in DMF (0.1 M, 1.0-5.0 equiv.) were added. The mixture was vortexed for a few seconds and shaken at room temperature for 240 minutes and analyzed by HPLC (Method **2**).

Procedure C for the solvent optimization: Ac-ENPECILDCHVQRVM-NH_2 (**16**) (0.50 to 3.0 mg, 0.24 to 1.5 µmol, 2.0 equiv.) was weighed in a 1.5 mL Eppendorf tube. A mixture of DMF/Water (1:1) that had been bubbled with nitrogen for 30 minutes was used to prepare a 1 mM solution. The solution was split in two fractions of the same volume. To both peptide solutions, DIPEA in DMF (0.2M, 2.5 equiv.) and then bis((3,3-bis(trifluoromethyl)-1l3-benzo[d][1,2]iodaoxol-1(3H)-yl)ethynyl)diisopropylsilane (**8c**) in DMF (0.1 M, 1.0-5.0 equiv.) were added. The mixture was vortexed for a few seconds and shaken at room temperature for 240 minutes and analyzed by HPLC (Method **2**).

Table S4: Solvent optimization. Yields are based on the model calibration (see Section 7: Peptide model Calibration)

Solvent	Procedure	Abs. 1 (mAu)	Abs. 2 (mAu)	Averaged Yield (%)
THF	A	0	0	0
DMF/THF	B	159	207	5
DMF/Dioxane	B	0	0	0
DMSO	A	2394	2367	62
DMF/Water	C	110	91	0

HPLC-UV Chromatograms (210 nm) (only one of the duplicates is shown):

Retention time stapled product (**16**): 15.1 min

Retention time starting peptide (**17a**): 13.2 min

THF:

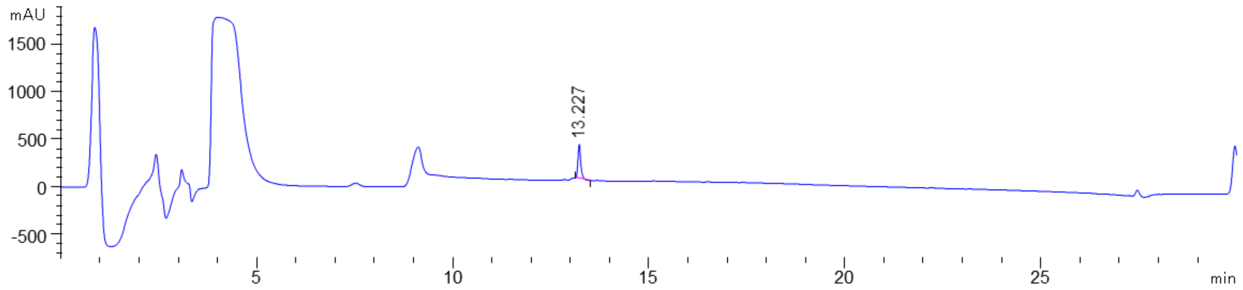

DMF/THF:

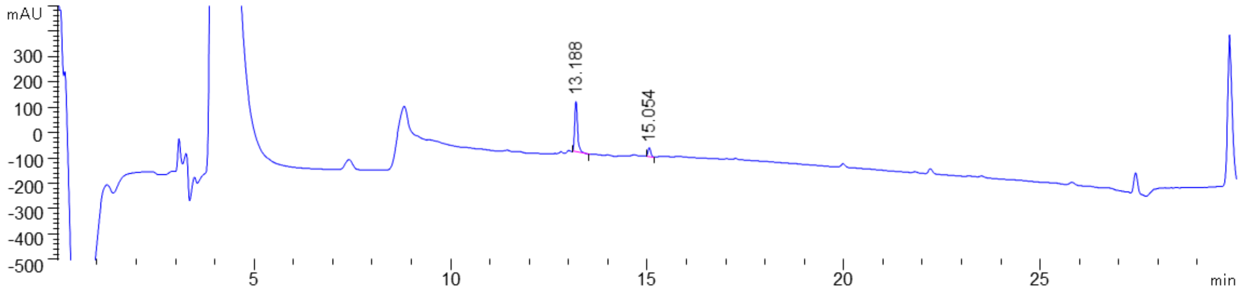

DMF/Dioxane:

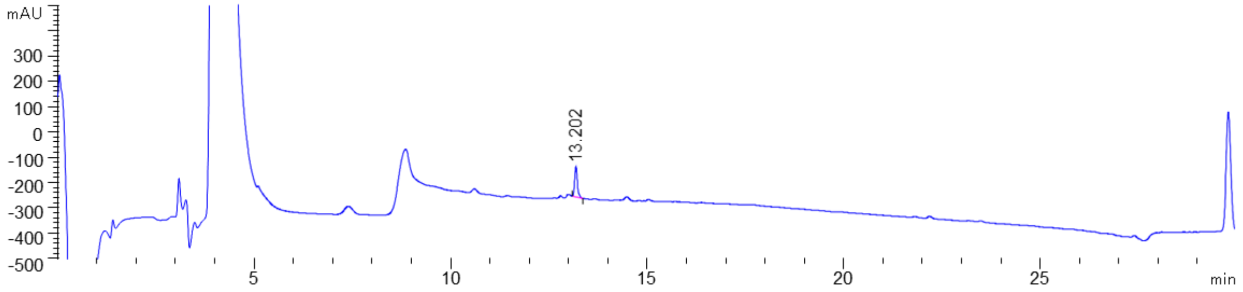

DMSO:

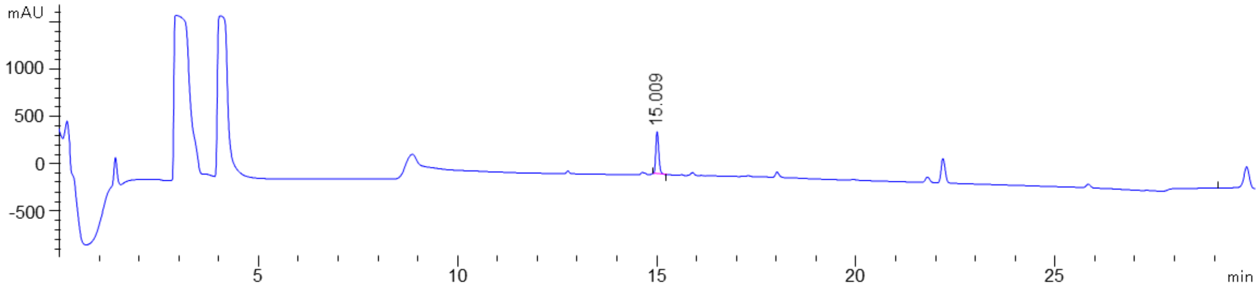

DMF/Water:

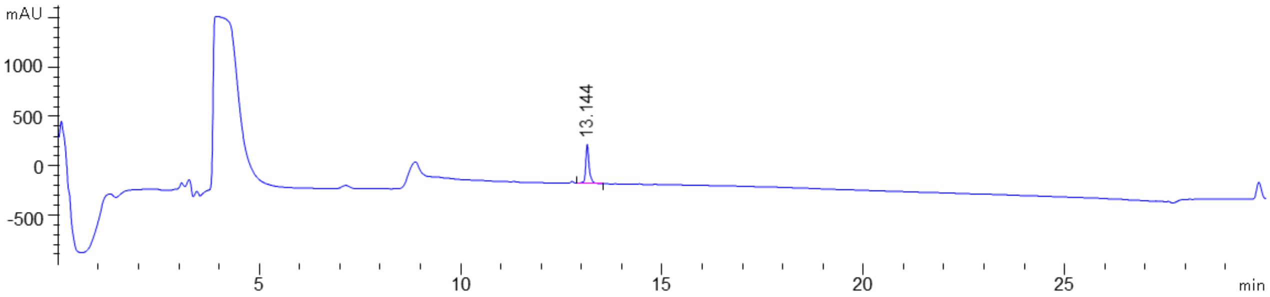

9. Cysteine-Cysteine Stapling Scope

General Procedure for Cysteine-Cysteine stapling on analytical scale: The selected linear peptide (0.50 to 3.0 mg, 1.0 equiv.) was weighed in a 1.5 mL Eppendorf tube. DMF that had been bubbled with nitrogen for 30 minutes was used to prepare a 1 mM solution. The solution was split in two fractions of the same volume. To both peptide solutions, DIPEA in DMF (0.2 M, 2.5 equiv.) and then the corresponding stapling reagent (**8**) in DMF (0.1 M, 3.0 equiv.) were added. The mixture was vortexed for a few seconds and shaken at 37 °C for 240, 1440 or 2880 minutes, and analysed by HPLC (Method **2**). The yields were approximated by comparison of the absorbances of the linear and the staple peptides, assuming no significant changes in absorbance at 210 nm.

General Procedure for isolation of Cysteine-Cysteine stapled peptides: The corresponding peptide (1.0 equiv.) was weighed in a 1.5 mL Eppendorf tube. DMF that had been bubbled with nitrogen for 30 minutes was used to prepare a 2 mM solution (when need the reaction transferred to a microwave seal cap vial. DIPEA (2.5 equiv.) and then the stapling reagent (3.0 equiv.) were added. The mixture was vortexed for a few seconds and shaken at 37 °C overnight. The reaction was then directly purified by preparative RP-HPLC.

Table S5: Peptide and reagent scope. Relative absorbance of stapled product compared to a standard solution of starting material at 210 nm. Absorbance for peptide **17** at 1 mM concentration = 3128.8 mAu. From the absorbance obtained for product **17a**, the absorbance increase for the linker can be calculated to be 18%. Considering errors arising from weighing small amounts of starting material, the relative absorbance is estimated to be 8-28% higher than the yield. Entries correspond to those in Table 2.

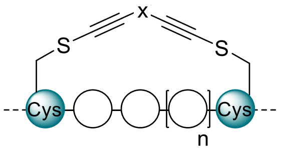

Entry	Product where x=	Linear Peptide	Linear Peptide Abs. (mAu)	Time (min)	Abs. 1 (mAu)	Abs. 2 (mAu)	Rel. abs. (%)
**1**	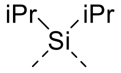 **17a**	Ac-ENPECILDCHVQRVM-NH_2	3129	240	2701	2794	98 (78)[46]
2	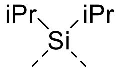 **19a**	Ac-YGGEAAREACARECAARE-NH_2	3676	240	2780	2544	72 [48]
				1440	2956	2429	73
3	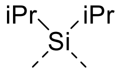 **21a**	Ac-QSQQTFCNLWRLLCQN-NH_2	3916	240	1182	1194	30 [13]
				1440	1039	1032	26
4	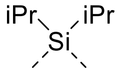 **23a**	H-SERCWHECYKNM-NH_2	3515	240	2551	2989	79
				1440	2413	2751	73
5	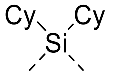 **17b**	Ac-ENPECILDCHVQRVM-NH_2	3129	240	2758	2786	89 (72)

6	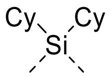 **19b**	Ac-YGGEAAREACARECAARE-NH2	3676	240	2658	2427	69
7	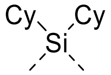 **21b**	Ac-QSQQTFCNLWRLLCQN-NH2	-	-	-	-	[22]
8	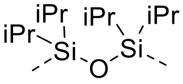 **17c**	Ac-ENPECILDCHVQRVM-NH2	3129	240	1628	1587	51 (40) [29]
9	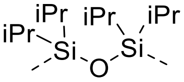 **19c**	Ac-YGGEAAREACARECAARE-NH2	3676	240	425	200	9 [19]
10	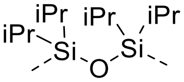 **21c**	Ac-QSQQTFCNLWRLLCQN-NH2	3916	240	1182	-	30
				1440	1717	-	44 [17]
				2880	1854	-	47
11	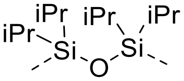 **23c**	H-SERCWHECYKNM-NH2	3515	240	559	503	15
				1440	1424	1564	42
12	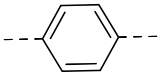 **17d**	Ac-ENPECILDCHVQRVM-NH2	3129	240	846	869	27 (19)
				1080	867	831	27 (19)
13	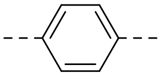 **21d**	Ac-QSQQTFCNLWRLLCQN-NH2	3916	240	1843	1776	46
				1440	1960	-	50
14	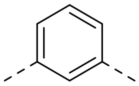 **17e**	Ac-ENPECILDCHVQRVM-NH2	3129	240	398	410	13 (6)
				1080	400	563	15 (8)
15	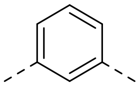 **21e**	Ac-QSQQTFCNLWRLLCQN-NH2	3916	240	521	528	13
				1440	585	-	15

Values in round brackets were calculated based on the calibration done for the product **17a**.
Values in square brackets are isolated yields.

Retention times of the common by-products:
22 min and 28 min - exhibit low ionization and originate from the excess of stapling reagent used.

Commonly observed side-product:
The majority of the side products present in the crude mixtures matched m/z that corresponded to oxidation of the linear peptide (Figure S2, A), mono-thioalkynylation or thioalkyne EBX-adduct staple; Figure S2, B), mono-adduct of EBX or bis EBX-staple (Figure S2, C). Double thioalkynylation (Figure S2, D) was only observed for some reagents when double loop stapling was done. In some cases, m/z that could not be assigned to any logical side product were also

detected. A comment is added above the chromatogram when significant amount of specific side product is present.

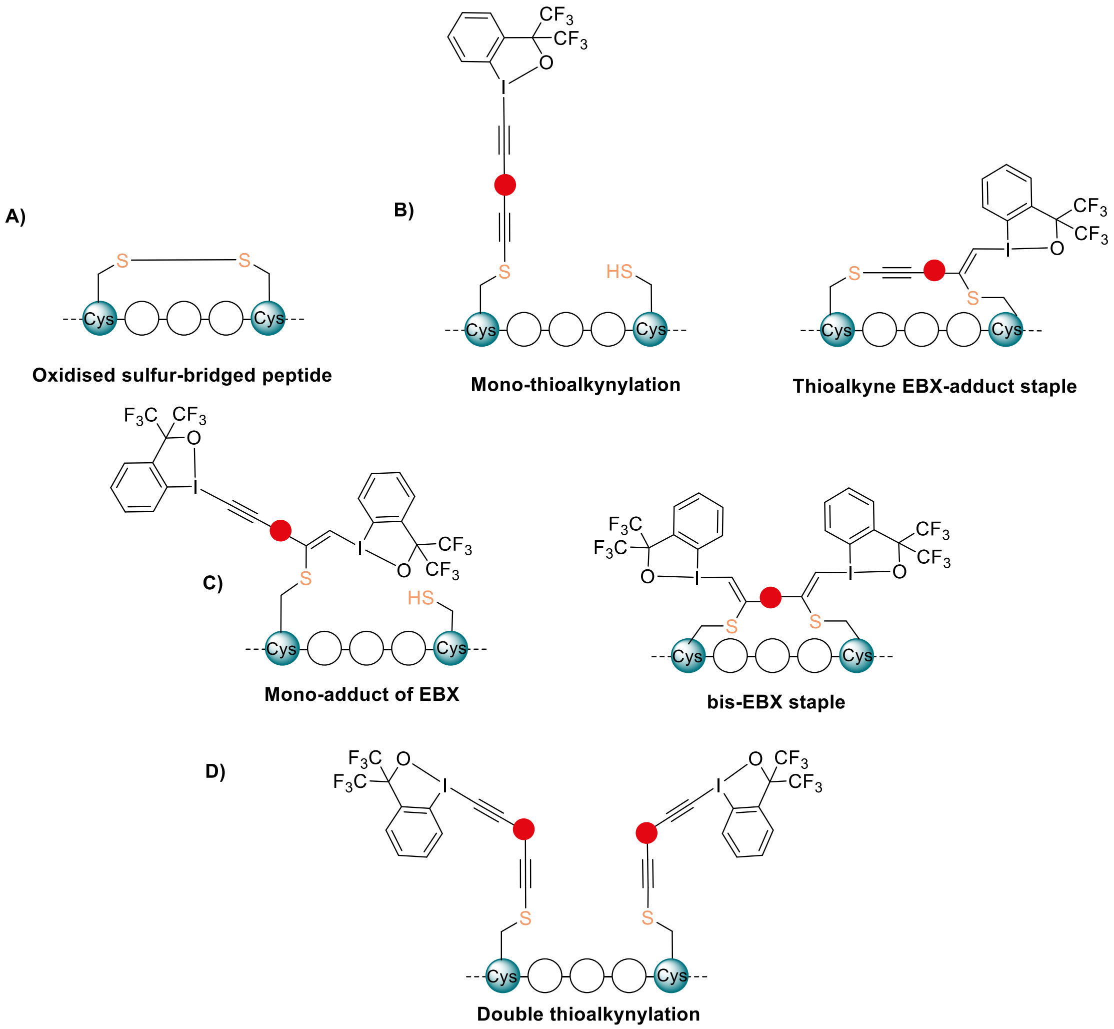

Figure S2: Structures of commonly observed side products.

17a (Entry 1, Table 2)

Following the general procedure for Cysteine-Cysteine stapling on analytical scale **17a** (retention time 15.1 min) was obtained in 98% relative absorbance after 4 hours.

HPLC-UV Chromatograms (210 nm) of the crude mixture (only one of the duplicates is shown):

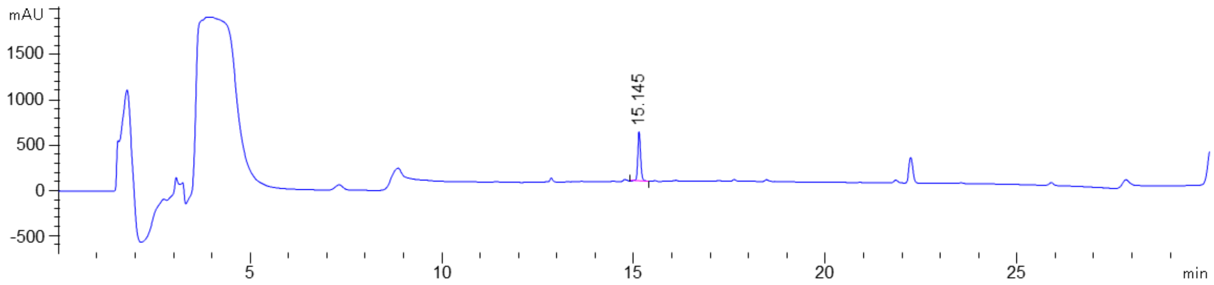

Following the general procedure for isolation of Cysteine-Cysteine stapled peptides, Ac-ENPECILDCHVQRVM-NH_2 (**16**) (6.8 mg, 3.3 µmol) together with bis((3,3-bis(trifluoromethyl)-1l3-benzo[d][1,2]iodaoxol-1(3H)-yl)ethynyl)diisopropylsilane (**8c**) afforded the product (**17a**) (3.4 mg, 1.5 µmol, 46%) as a white amorphous solid (retention time 15.0 min).

HPLC-UV chromatogram at 210 nm of the isolated product:

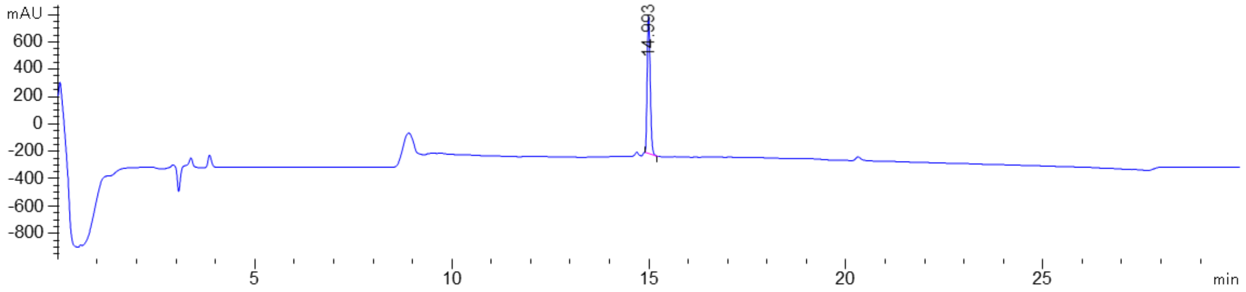

19a (Entry 2, Table 2)

Following the general procedure for Cysteine-Cysteine stapling on analytical scale **19a** (retention time 13.6 min) was obtained in 72% relative absorbance after 4 hours.

HRMS (nanochip-ESI/LTQ-Orbitrap) m/z: $[M + H]^{+2}$ Calcd for $C_{87}H_{138}N_{28}O_{28}S_2Si^{+2}$ 1057.4718; Found 1057.4718.

HPLC-UV Chromatogram (210 nm) of the crude mixture (only one of the duplicates is shown):

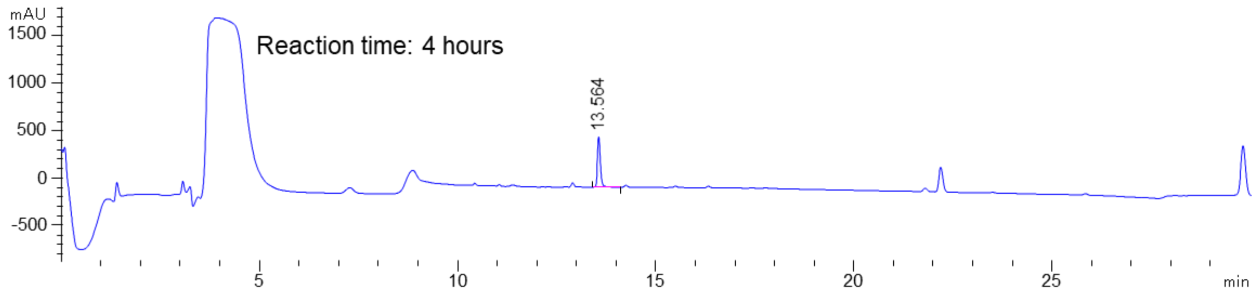

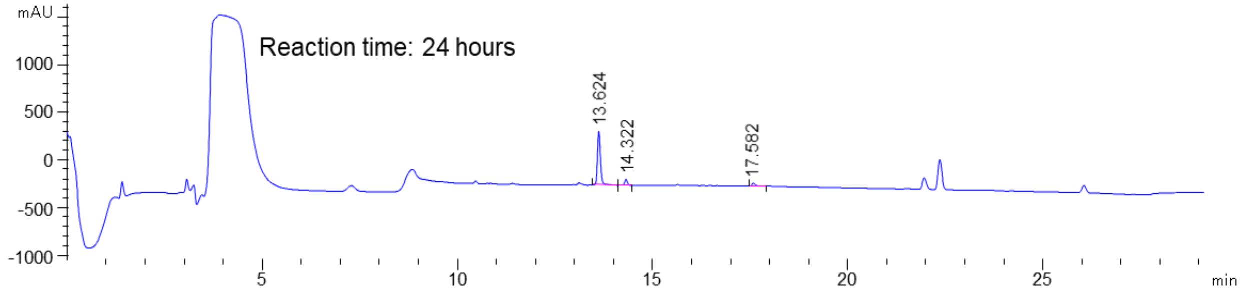

Following the general procedure for isolation of Cysteine-Cysteine stapled peptides, Ac-YGGEAAREACARECAARE-NH_2 (**18**) (4.9 mg, 2.1 µmol) together with bis((3,3-bis(trifluoromethyl)-1l3-benzo[d][1,2]iodaoxol-1(3H)-yl)ethynyl)diisopropylsilane (**8c**) afforded the product (**19a**) (2.3 mg, 0.95 µmol, 48%) as a white amorphous solid (retention time 13.7 min).

HPLC-UV chromatogram at 210 nm of the isolated product:

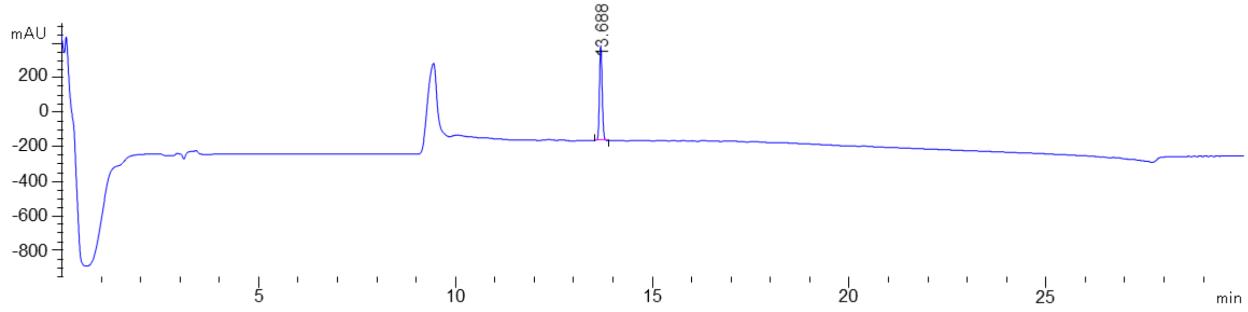

21a (Entry 3, Table 2)

Following the general procedure for Cysteine-Cysteine stapling on analytical scale **21a** (retention time 17.1 min) was obtained in 30% relative absorbance after 4 hours.

HRMS (nanochip-ESI/LTQ-Orbitrap) m/z: $[M + Na]^{+2}$ Calcd for $C_{97}H_{148}N_{27}NaO_{25}S_2Si^{+2}$ 1103.0119; Found 1103.0131.

HPLC-UV Chromatogram (210 nm) of the crude mixture (only one of the duplicates is shown): 21.5: m/z = 903.4, 1354.9. unknown product (1855 mAU).

21.8: m/z = 394.9 by-products of stapling reagent.

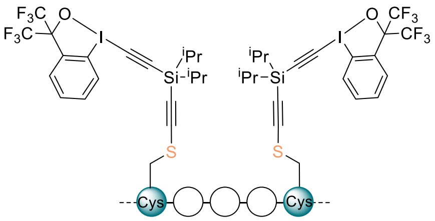

Chemical Formula: $C_{125}H_{171}F_{12}I_2N_{27}O_{27}S_2Si_2^{2+}$
m/z: 1542.4869 (100.0%))

23.7: m/z = 1542.5 (+2), 1028.7 (+3). double thioalkynylation.29.8: m/z = 525.0 by-products of stapling reagents (1482 mAU).

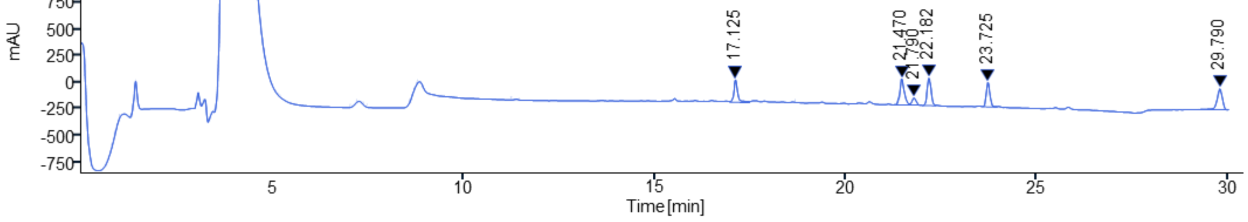

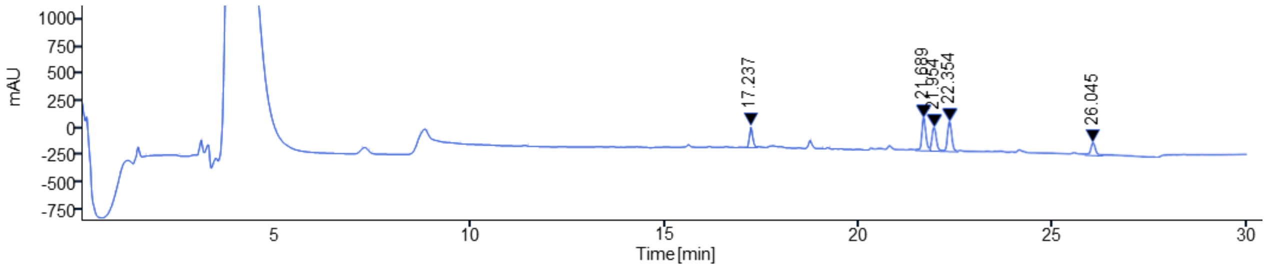

MS/MS Characterization:

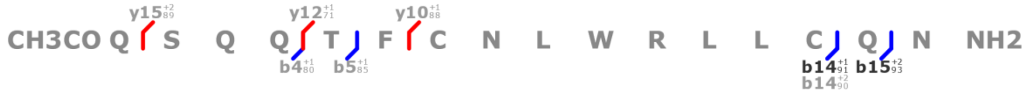

b4 (3.8), **b5** (4.1), ***b11**** *(0.6)*, ***b12**** *(0.7)*, ***b14*** *(24.0)*, ***b14*** *(12.8)*, ***b15*** *(26.7)*, ***y10*** *(26.4)*, ***y12*** *(3.2)*, ***y15*** *(22.8)*.

Following the general procedure for isolation of Cysteine-Cysteine stapled peptides, Ac-QSQQTFCNLWRLLKQN-NH_2 (**20**) (15.6 mg, 7.28 µmol) together with bis((3,3-bis(trifluoromethyl)-1l3-benzo[d][1,2]iodaoxol-1(3H)-yl)ethynyl)diisopropylsilane (**8c**) afforded the product (**21a**) (2.2 mg, 0.96 µmol, 13%) as a white amorphous solid (retention time 17.1 min).

HPLC-UV chromatogram at 210 nm of the isolated product:

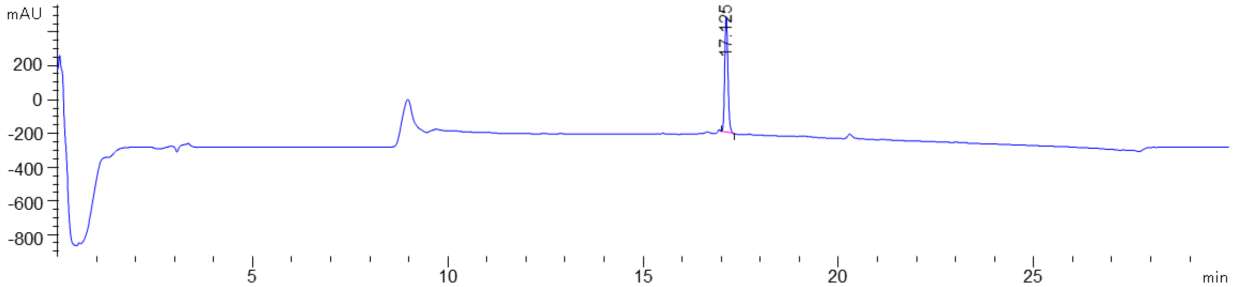

23a (Entry 4, Table 2)

Following the general procedure for Cysteine-Cysteine stapling on analytical scale **23a** (retention time 13.7 min) was obtained in 79% relative absorbance after 4 hours.

HRMS (ESI/QTOF) m/z: $[M + H]^{+2}$ Calcd for $C_{76}H_{111}N_{21}O_{19}S_3Si^{+2}$ 872.8643; Found 872.8637.

HPLC-UV Chromatogram (210 nm) of the crude mixture (only one of the duplicates is shown):

Retention time oxidized peptide: 10.3 min

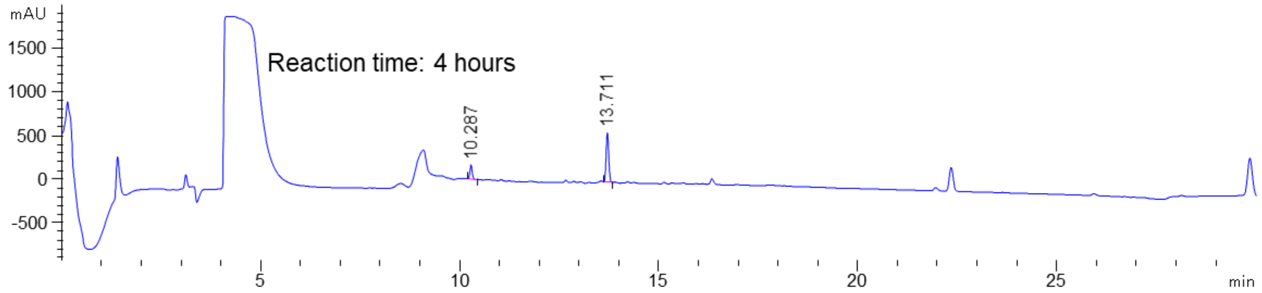

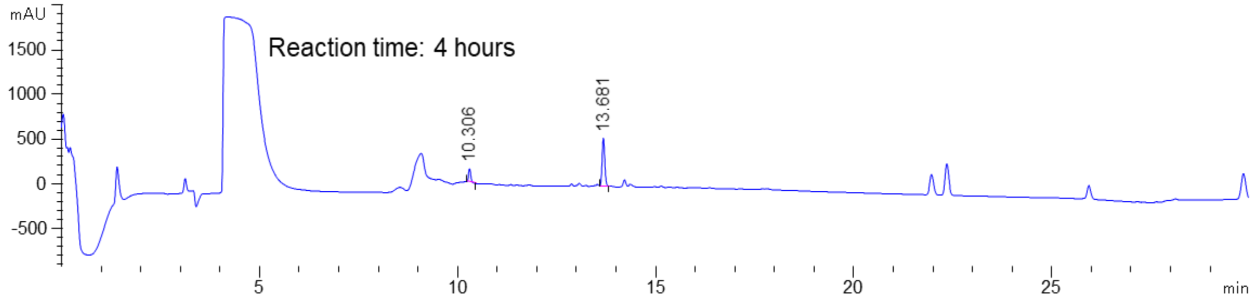

MS/MS Characterization:

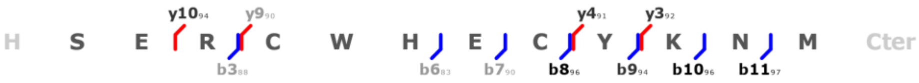

b3 (6.1), **b6*** (45.7), **b7*** (1.0), ***b8*** *(12.5)*, ***b8*** *(4.8)*, ***b9*** *(9.2)*, ***b9*** *(11.0)*, ***b10*** *(7.7)*, ***b10*** *(48.6)*, ***b11*** *(50.4)*, **y3** (2.8), **y4** (3.9), ***y9*** *(5.8)*, ***y9*** (1.4), ***y10*** *(8.0)*.

17b (Entry 5, Table 2)

Following the general procedure for Cysteine-Cysteine stapling on analytical scale **17b** (retention time 16.3 min) was obtained in 89% relative absorbance after 4 hours.

HRMS (nanochip-ESI/LTQ-Orbitrap) m/z: $[M + H_2]^{+2}$ Calcd for $C_{91}H_{145}N_{23}O_{24}S_3Si^{+2}$ 1033.9877; Found 1033.9888.

HPLC-UV Chromatogram (210 nm) (only one of the duplicates is shown):

Retention time (**46**) (in Method 2): 22.2 min

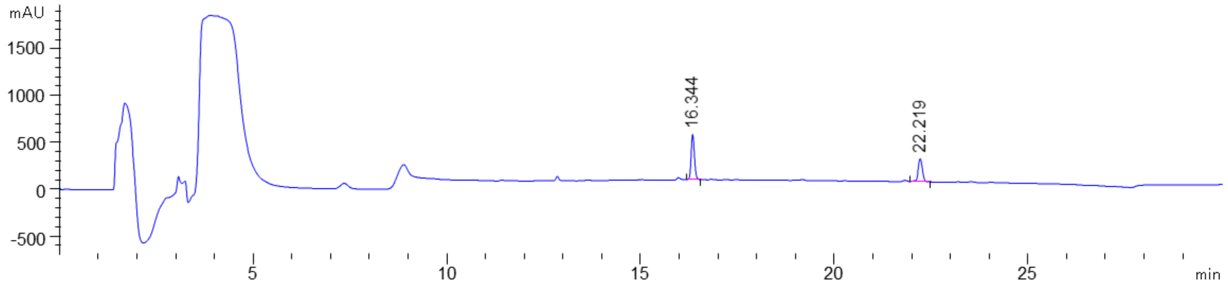

MS/MS characterization:

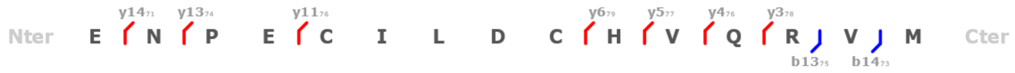

b13 (58.9), ***b14*** (100.3), **y3** (2.4), **y4** (6.0), **y5** (3.1), **y6** (5.1), ***y11*** (1.5), ***y13*** (5.4), ***y14*** (3.3).

19b (Entry 6, Table 2)

Following the general procedure for Cysteine-Cysteine stapling on analytical scale **19b** (retention time 14.9 min) was obtained in 69% relative absorbance after 4 hours.

HRMS (ESI/QTOF) m/z: $[M + H_2]^{+2}$ Calcd for $C_{93}H_{146}N_{28}O_{28}S_2Si^{+2}$ 1097.5031; Found 1097.5038.

HPLC-UV Chromatogram (210 nm) of the crude mixture (only one of the duplicates is shown):

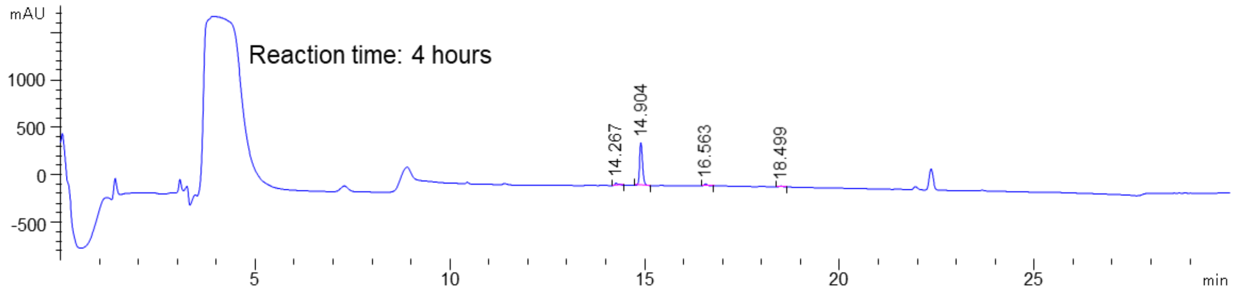

21b (Entry 7, Table 2)

Following the general procedure for isolation of Cysteine-Cysteine stapled peptides, Ac-QSQQTFCNLWRLLCQN-NH_2 (**20**) (13.7 mg, 6.41 µmol) together with bis((3,3-bis(trifluoromethyl)-1l3-benzo[d][1,2]iodaoxol-1(3H)-yl)ethynyl)dicyclohexylsilane (**8d**) afforded the product (**21b**) (3.4 mg, 1.5 µmol, 22%) as a white amorphous solid (retention time 18.5 min).

HRMS (nanochip-ESI/LTQ-Orbitrap) m/z: $[M + H_2]^{+2}$ Calcd for $C_{103}H_{157}N_{27}O_{25}S_2Si^{+2}$ 1132.0522; Found 1132.0557.

HPLC-UV chromatogram at 210 nm of the isolated product:

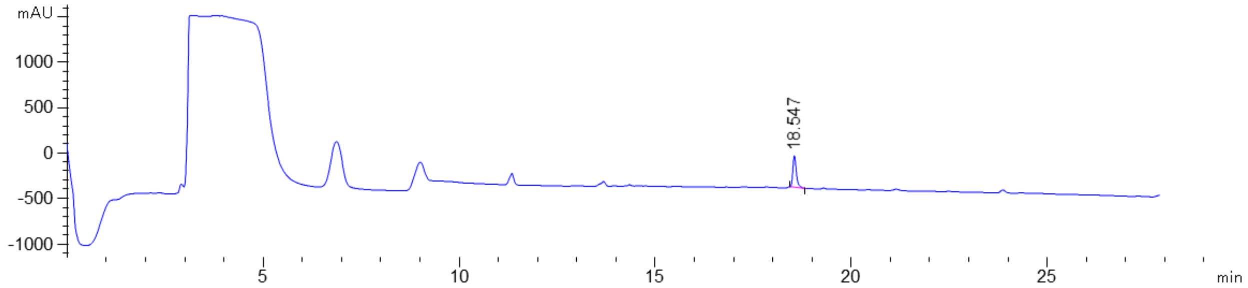

17c (Entry 8, Table 2)

Following the general procedure for Cysteine-Cysteine stapling on analytical scale **19b** (retention time 16.8 min) was obtained in 51% relative absorbance after 4 hours.

HRMS (nanochip-ESI/LTQ-Orbitrap) m/z: $[M+H_2]^{+2}$ Calcd for $C_{91}H_{149}N_{23}O_{25}S_3Si_2^{+2}$ 1057.9892; Found 1057.9891.

HPLC-UV Chromatogram (210 nm) of the crude mixture (only one of the duplicates is shown):

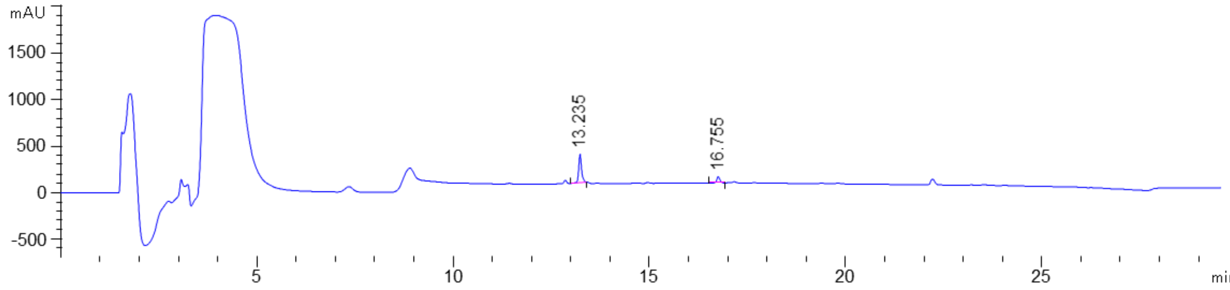

MS/MS characterization:

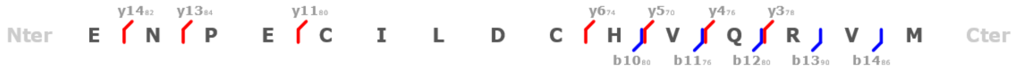

b10 *(0.9)*, ***b11*** *(1.9)*, ***b12*** *(1.3)*, ***b13*** *(57.8)*, ***b14*** *(101.3)*, **y3** (1.6), **y4** (4.6), **y5** (2.7), **y6** (3.1), ***y11*** *(0.8)*, ***y11*** *(0.9)*, ***y13*** *(4.7)*, ***y14*** *(3.0)*.

Following the general procedure for isolation of Cysteine-Cysteine stapled peptides, Ac-ENPECILDCHVQRVM-NH_2 (**16**) (9.7 mg, 4.7 µmol) together with 1,3-bis((3,3-bis(trifluoromethyl)-1l3-benzo[d][1,2]iodaoxol-1(3H)-yl)ethynyl)-1,1,3,3-tetraisopropyldisiloxane (**8e**) afforded the product (**17c**) (3.2 mg, 1.4 µmol, 29%) as a white amorphous solid (retention time 16.5 min).

HPLC-UV chromatogram at 210 nm of the isolated product:

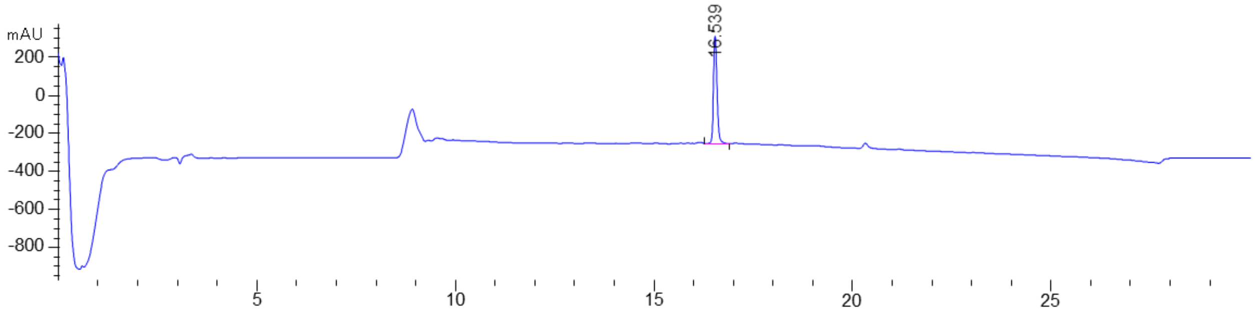

19c (Entry 9, Table 2)

Following the general procedure for Cysteine-Cysteine stapling on analytical scale **19c** (retention time 15.4 min) was obtained in 9% relative absorbance after 4 hours.

HRMS (ESI/QTOF) m/z: $[M]^+$ Calcd for $C_{93}H_{151}N_{28}O_{29}S_2Si_2^+$ 2244.0176; Found 2244.0127.

HPLC-UV Chromatogram (210 nm) of the crude mixture (only one of the duplicates is shown):

Retention time starting linear peptide: 11.1 min

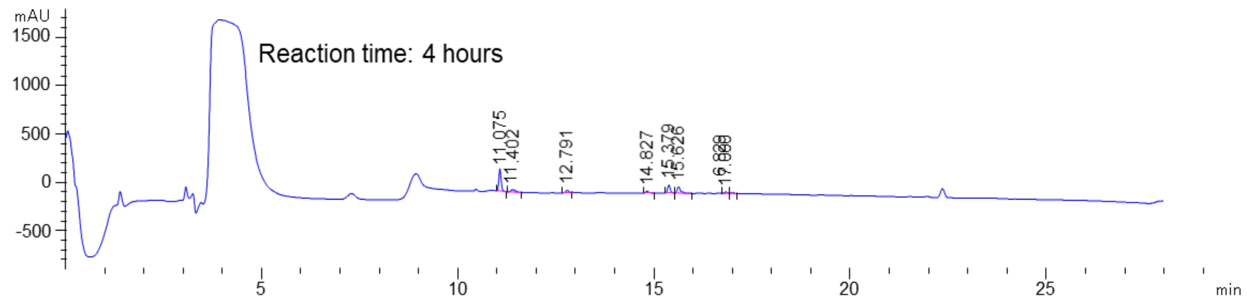

Following the general procedure for isolation of Cysteine-Cysteine stapled peptides, Ac-YGGEAAREACARECAARE-NH_2 (**18**) (9.2 mg, 4.0 µmol) together with 1,3-bis((3,3-bis(trifluoromethyl)-1l3-benzo[d][1,2]iodaoxol-1(3H)-yl)ethynyl)-1,1,3,3-tetraisopropyldisiloxane (**8e**) afforded the product (**19c**) (1.9 mg, 0.74 µmol, 19%) as a white amorphous solid (retention time 15.3 min).

HPLC-UV chromatogram at 210 nm of the isolated product:

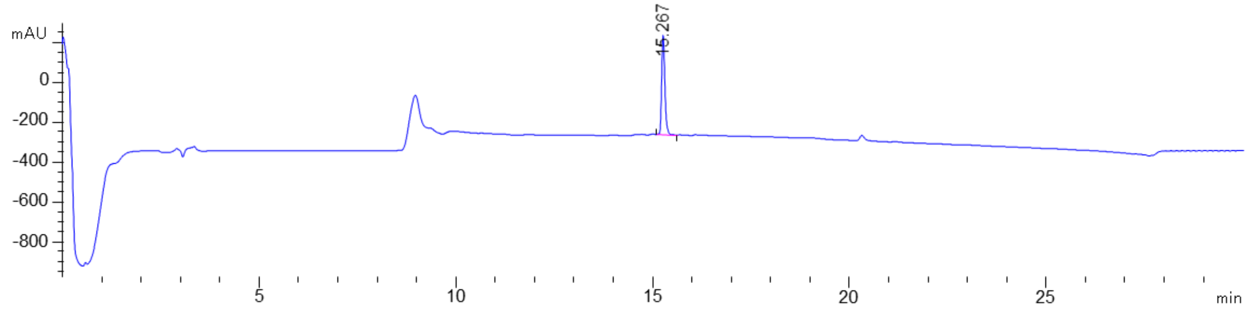

21c (Entry 10, Table 2)

Following the general procedure for Cysteine-Cysteine stapling on analytical scale **21c** (retention time 19.1 min) was obtained in 44% relative absorbance after 24 hours.

HRMS (ESI/QTOF) m/z: $[M]^+$ Calcd for $C_{103}H_{162}N_{27}O_{26}S_2Si^{1+}$ 2313.1159; Found 2313.1079

HPLC-UV Chromatogram (210 nm) (only one of the duplicates is shown):

Retention time starting linear peptide: 15.7 min

Retention time oxidized peptide: 15.8 min

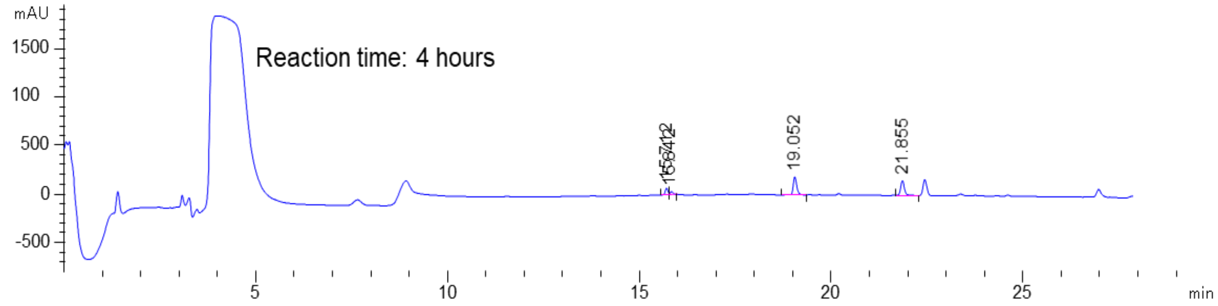

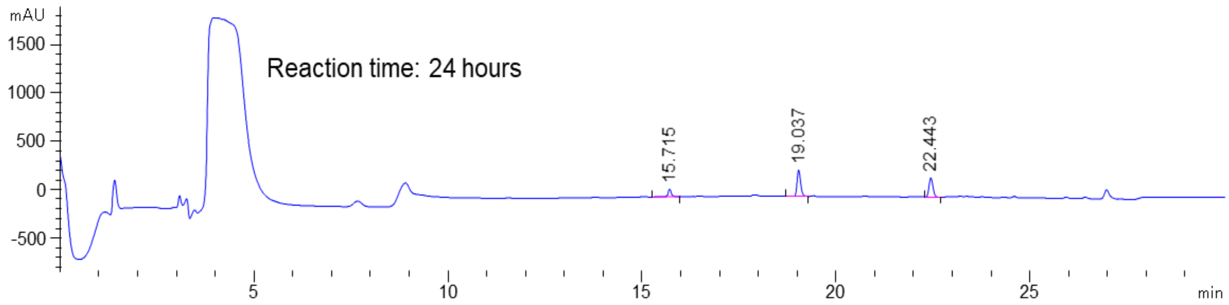

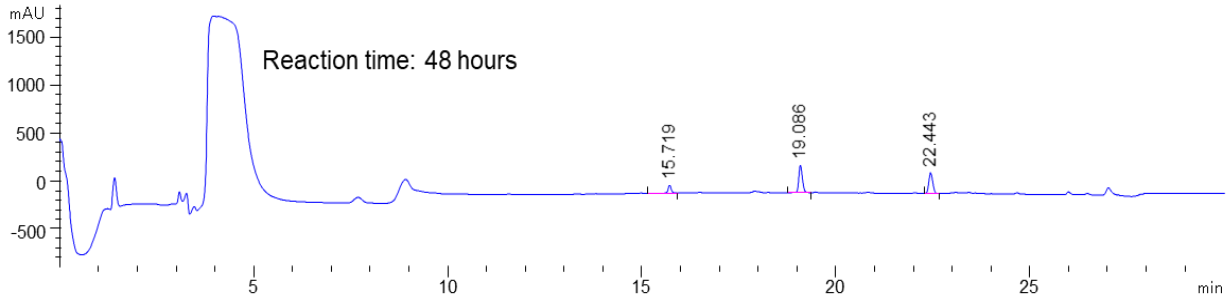

Following the general procedure for isolation of Cysteine-Cysteine stapled peptides, Ac-QSQQTFCNLWRLLKQN-NH_2 (**20**) (13.9 mg, 6.50 µmol) together with 1,3-bis((3,3-bis(trifluoromethyl)-1l3-benzo[d][1,2]iodaoxol-1(3H)-yl)ethynyl)-1,1,3,3-tetraisopropyldisiloxane (**8e**) afforded the product (**21c**) (2.6 mg, 1.1 µmol, 17%) as a white amorphous solid.

HPLC-UV chromatogram at 210 nm of the isolated product:

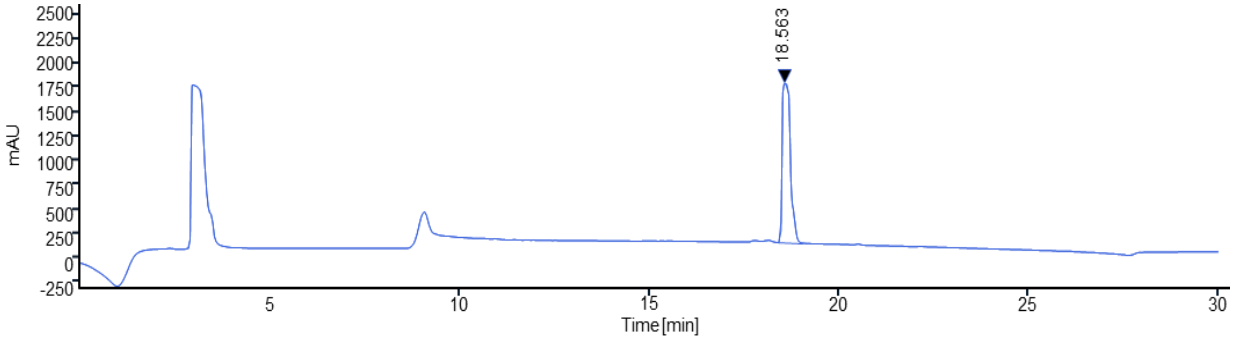

23c (Entry 11, Table 2)

Following the general procedure for Cysteine-Cysteine stapling on analytical scale **23c** (retention time 15.1 min) was obtained in 42% relative absorbance after 24 hours. The HPLC Chromatogram shows a similar area of stapled product (15.1 min) and oxidized product (10.3 min), which is in accordance to the relative absorbance reported (42%). The peak at 22.4 min corresponds to the stapling reagent, which was added in excess and therefore should not be considered for the relative absorbance.

HRMS (ESI/QTOF) m/z: $[M + H]^{+2}$ Calcd for $C_{82}H_{125}N_{21}O_{20}S_3Si_2^{+2}$ 937.9050; Found 937.9046

HPLC-UV Chromatogram (210 nm) (only one of the duplicates is shown):

Retention time starting linear peptide: 10.8 min

Retention time oxidized peptide: 10.3 min

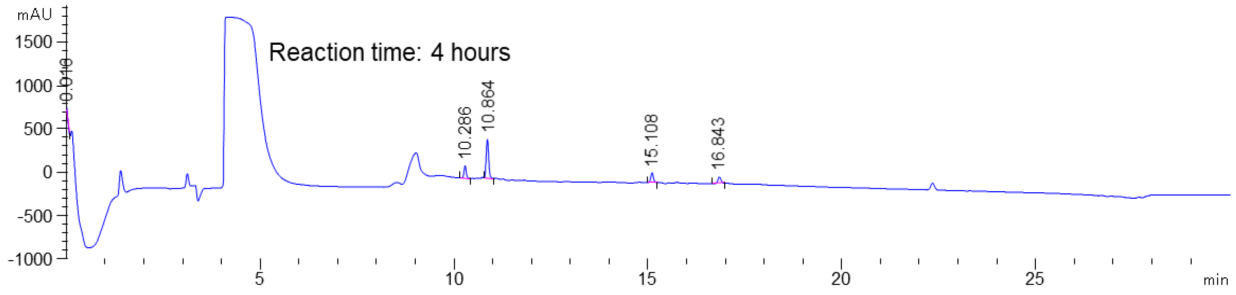

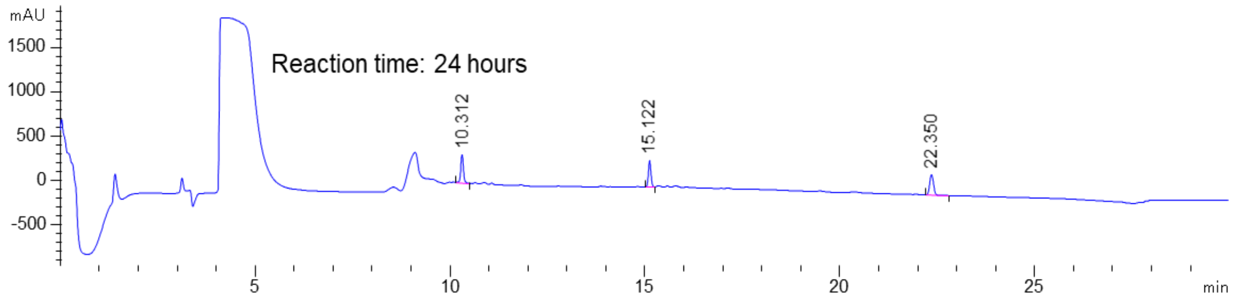

17d (Entry 12, Table 2)

Following the general procedure for Cysteine-Cysteine stapling on analytical scale **17d** (retention time 14.5 min) was obtained in 27% relative absorbance after 4 hours. For further clarification of occurring side-reactions, all of the potential peptide-related peaks were integrated (see Table S6 below) and the potential structures of the detected side-products have been drawn (see Figure S2). The 27% reported relative absorbance correlates well with the 20% of total area reported in the table below. We believe the relative area estimation to be more accurate. Only the relative absorbances were reported in the main part of the manuscript.

Table S6: Side-products detected in the formation of **17d** after 4 hours

Entry	Ret. Time (min)	Abs. 1 (mAu)	Abs. 2 (mAu)	Rel. Area from all products (%)	Assignment (see figure S2)
**1**	12.8	603	604	14	linear peptide (**16**)
**2**	14.5	846	869	20	desired product
**3**	15.4	1782	1834	42	mono-thioalkynylation or thioalkyne EBX-adduct staple
**4**	15.9	284	263	6	mono-thioalkynylation or thioalkyne EBX-adduct staple
**5**	16.6	772	824	18	mono-adduct of EBX or bis EBX-staple

Peaks at 22.2 and 27.6 min exhibit low ionization and are coming from the excess of reagent, thus the absorbances of these peaks have been excluded for the above estimation of yield.

HRMS (nanochip-ESI/LTQ-Orbitrap) m/z: $[M + HNa]^{+2}$ Calcd for $C_{85}H_{126}N_{23}NaO_{24}S_3^{+2}$ 985.9197; Found 985.9264.

HPLC-UV Chromatogram (210 nm) of the crude mixture (only one of the duplicates is shown):

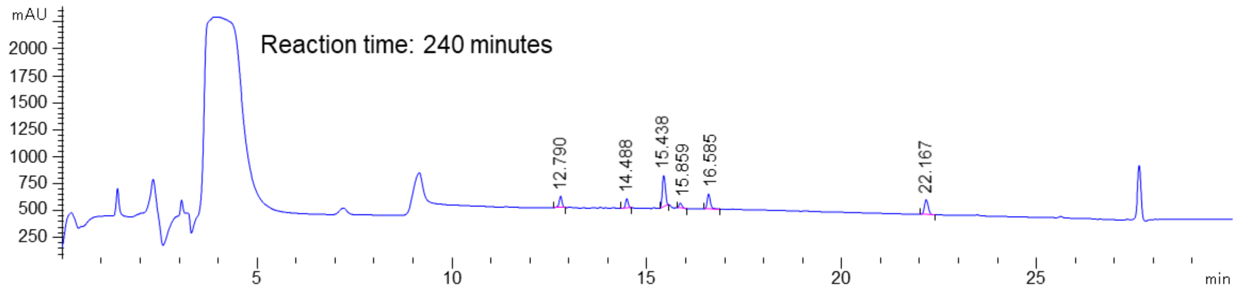

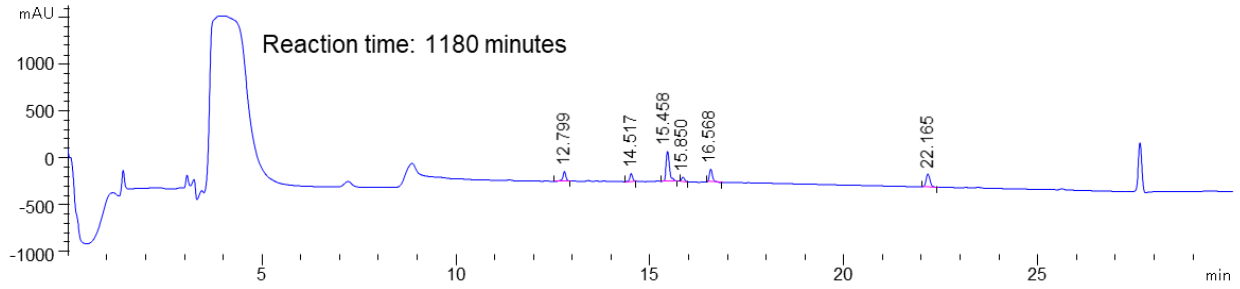

21d (Entry 13, Table 2)

Following the general procedure for Cysteine-Cysteine stapling on analytical scale **21d** (retention time 16.1 min) was obtained in 46% relative absorbance after 4 hours. All of the potential peptide-related peaks were integrated (see Table S7 below). The 46% reported relative absorbance correlates well with the 42% of total area reported in the table below.

Table S7: Side-products detected in the formation of **21d** after 4 hours

Entry	Ret. Time (min)	Abs. (mAu)	Abs. 2 (mAu)	Rel. Area from all products (%)	Assignment (see figure below for structures)
**1**	12.8	382	372	42	desired product
**2**	17.4	880	839	20	mono-thioalkynylation or thioalkyne EBX-adduct staple
**3**	17.7	1682	1682	39	mono-thioalkynylation or thioalkyne EBX-adduct staple

Peaks at 22.2 and 27.6 min exhibit low ionization and are coming from the excess of reagent, thus the absorbances of these peaks have been excluded for the above estimation of yield.

HRMS (nanochip-ESI/LTQ-Orbitrap) m/z: $[M + H]^+$ Calcd for $C_{97}H_{139}N_{27}O_{25}S^{1+}$ 2145.9871; Found 2145.9818

HPLC-UV Chromatogram (210 nm) of the crude mixture (only one of the duplicates is shown):

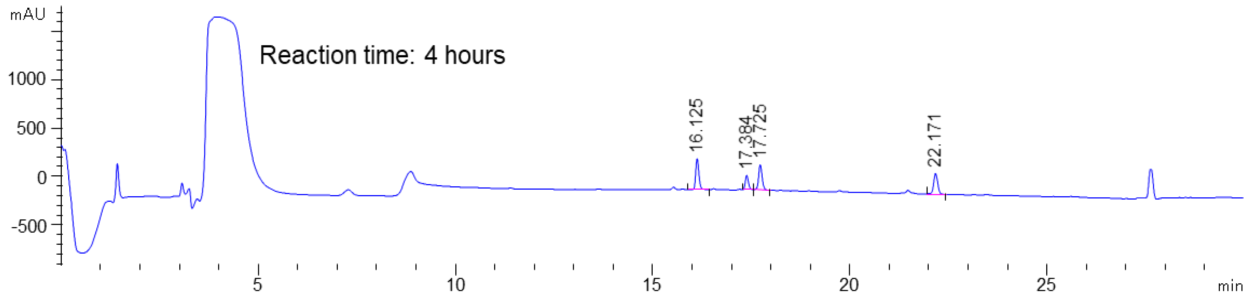

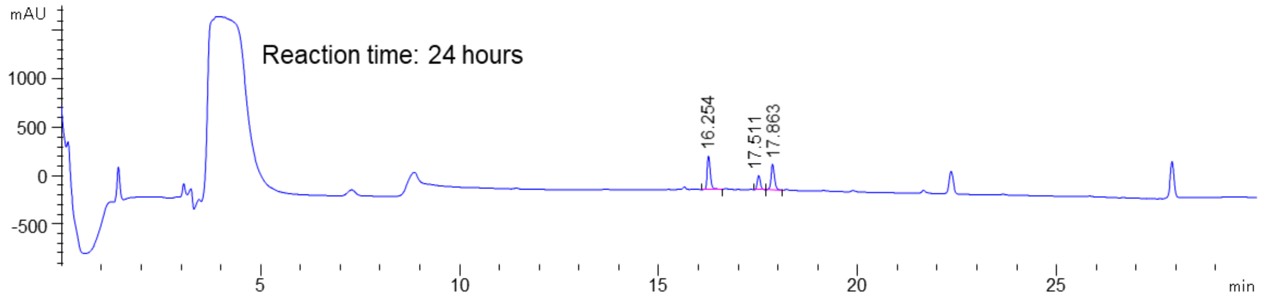

17e (Entry 14, Table 2)

Following the general procedure for Cysteine-Cysteine stapling on analytical scale **17e** (retention time 13.9 min) was obtained in 13% relative absorbance after 4 hours. All of the potential peptide-related peaks were integrated (see Table S8). The 13% reported relative absorbance correlates well with the 14% of total area reported in the table below.

Table S8: Side-products detected in the formation of **17e** after 4 hours

Entry	Ret. Time (min)	Abs. (mAu)	Abs. 2 (mAu)	Rel. Area from all products (%)	Assignment (see figure below for structures)
**1**	12.8	382	372	14	linear peptide (**16**)
**2**	13.9	398	410	14	desired product
**3**	15.6	946	943	34	mono-thioalkynylation or thioalkyne EBX-adduct staple
**4**	16.0	746	764	27	mono-thioalkynylation or thioalkyne EBX-adduct staple
**5**	18.5	295	319	11	unknown Product (m/z 706.2, 937.9, 1406.3)

Peaks at 22.2 and 27.6 min exhibit low ionization and are coming from the excess of reagent, thus the absorbances of these peaks have been excluded for the above estimation of yield

HRMS (nanochip-ESI/LTQ-Orbitrap) m/z: $[M + H_2]^{+2}$ Calcd for $C_{85}H_{127}N_{23}O_{24}S_3^{+2}$ 974.9288; Found 974.9295.

HPLC-UV Chromatogram (210 nm) of the crude mixture (only one of the duplicates is shown):

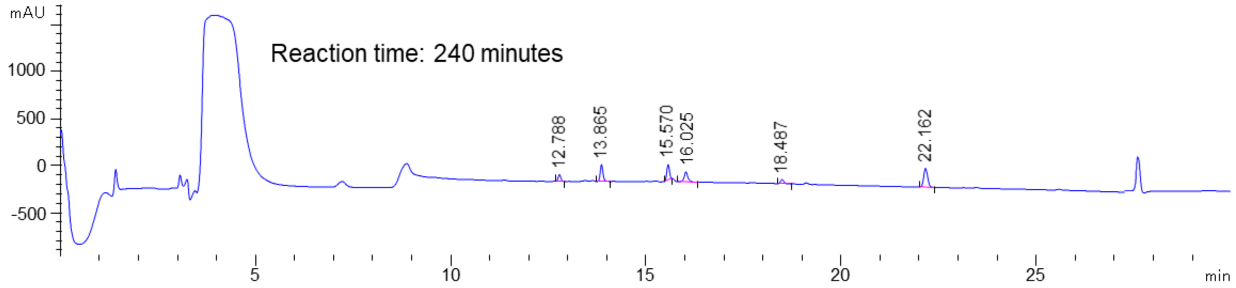

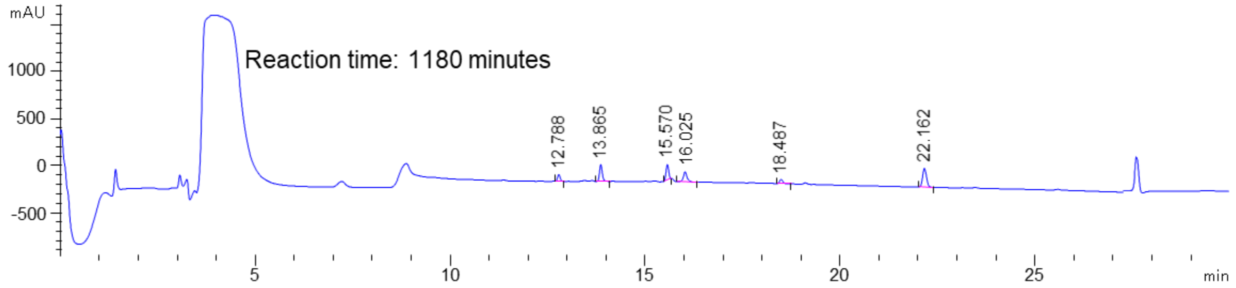

21e (Entry 15, Table 2)

Following the general procedure for Cysteine-Cysteine stapling on analytical scale **21e** (retention time 16.5 min) was obtained in 13% relative absorbance after 4 hours.

HRMS (nanochip-ESI/LTQ-Orbitrap) m/z: $[M + Na]^{+2}$ Calcd for $C_{97}H_{139}N_{27}NaO_{25}S_2^{+2}$ 1084.4882; Found 1084.4860.

HPLC-UV Chromatogram (210 nm) (only one of the duplicates is shown):

Retention time starting linear peptide: 15.6 min

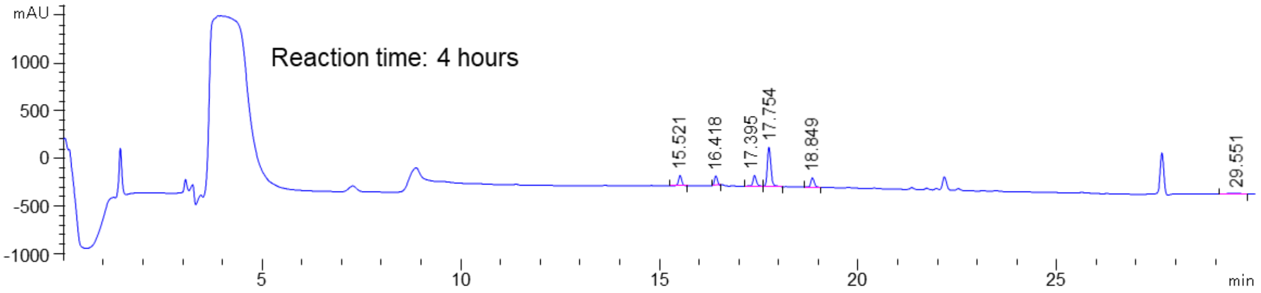

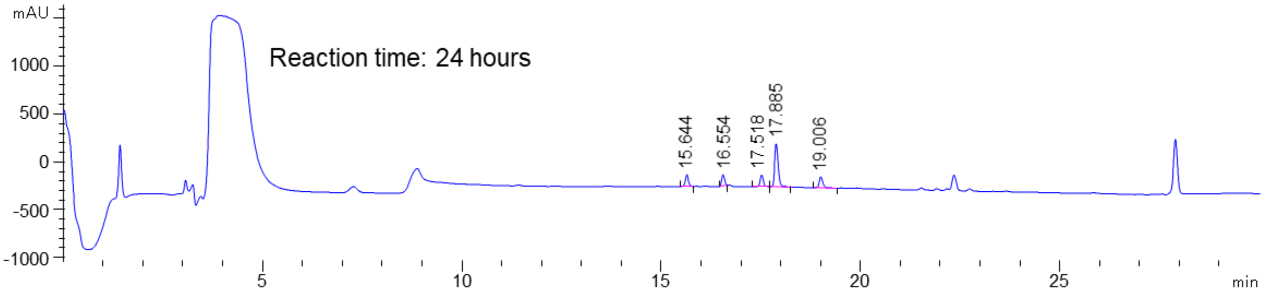

10. Cysteine-Lysine Stapling Scope

General Procedure for Cysteine-Lysine stapling on analytical scale: The selected linear peptide (0.50 to 3.0 mg, 1.0 equiv.) was weighed in a 1.5 mL Eppendorf tube. DMF that had been bubbled with nitrogen for 30 minutes was used to prepare a 1 mM solution. The solution was split in two fractions of the same volume. To both peptide solutions, DIPEA in DMF (0.2 M, 2.5 equiv.) and then the corresponding stapling reagent (**9**) in DMF (0.1 M, 1.1 equiv.) was added. The mixture was vortexed for a few seconds and shaken at 37 °C for 30 minutes (unless otherwise indicated), and analyzed by HPLC (Method **2**). The yields were approximated by comparison of the absorbances of the linear and the stapled peptides, assuming no change in absorbance.

General Procedure for isolation of Cysteine-Lysine stapled peptides: The corresponding peptide (1.0 equiv.) was weighed in a 1.5 mL Eppendorf tube. DMF that had been bubbled with nitrogen for 30 minutes was used to prepare a 1 mM solution (when the volume was bigger than 1.5 mL, the reaction was transferred to a microwave seal cap vial). DIPEA (2.5 equiv.) and then the stapling reagent (1.1 equiv.) were added. The mixture was vortexed for a few seconds and shaken from 1 to 24 hours. The reaction was then directly purified by preparative RP-HPLC.

Table S9: Cysteine-Lysine peptide and reagent scope. Relative absorbance of stapled product compared to a standard solution of starting material at 210 nm. The absorbance increase for the products was calculated to be 15% by comparing absorbance of 1 mM solution of **28** and **29a** at 210 nm. Considering errors arising from weighing small amounts of starting material, the relative absorbance is estimated to be 5-25% higher than the yield. Entries correspond to those in Table 2.

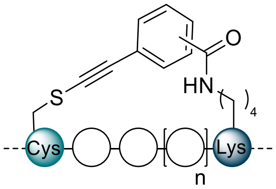

Entry	Substitution pattern	Linear Peptide	Linear Peptide Abs. (mAu)	Time (min)	Abs. 1 (mAu)	Abs. 2 (mAu)	Rel. abs. (%)
16	*para* (**25a**)	Ac-ENPECILDKHVQRVM-NH_2	3127	30	2020	1845	62
				1440	3084	2811	94 [52]
17	*para* (**27a**)	Ac-YGGEAAREACAREKAARE-NH_2	2414	30	2645	2845	114 [65]
18	*para* (**29a**)	Ac-QSQQTFCNLWRLLKQN-NH_2	4134	30	4894	4811	117 [87]
19	*para* (**31a**)	H-SERCWHEKYNM-NH_2	3417	1440[a]	2409	2314	69
20	*para* (**33a, 33a`**)	H-RSQFYKHDAGCG-NH_2	2739	30	1486[b] (501)[c]	1463[b] (474)[c]	54[b] (18)[c]
				1440	1958[b] (670)[c]	1801[b] (585)[c]	69[b] (23)[c]
21	*meta* (**25b**)	Ac-ENPECILDKHVQRVM-NH_2	3127	30	1066	1049	34 [7]
22	*meta* (**27b**)	Ac-YGGEAAREACAREKAARE-NH_2	2414	30	1899	1839	77 [44]
23	*meta* (**29b**)	Ac-QSQQTFCNLWRLLKQN-NH_2	4134	30	4556	4504	110 [55]
24	*meta* (**31b**)	H-SERCWHEKYNM-NH_2	3417	30	702	698	20
25	*meta* (**33b**)	H-RSQFYKHDAGCG-NH_2	2739	30	606	603	22
26	*ortho* (**25c**)	Ac-ENPECILDKHVQRVM-NH_2	3127	30	3205	3082	101

				1440	1947	1666	58
27	*ortho* (**27c**)	Ac-YGGEAAREACAREKAARE-NH_2	2414	30	2827	2878	118
				1440	1041	961	41
28	*ortho* (**29c**)	Ac-QSQQTFCNLWRLLKQN-NH_2	4134	30	4870	5270	123
				1440	4254	4435	105

[a] The reaction was analyzed after 30 minutes, but a significant amount of activated ester intermediate was detected.
[b] Major product, tentatively assigned as Cys-Lys stapling based on MS/MS analysis.
[c] Minor product, tentatively assigned as Cys-N-terminus stapling based on MS/MS analysis.
Yield in square brackets correspond to the isolated yield.

Retention times of the common by-products:

2-iodobenzoic acid (**51**)	17.5-17.6 min
Diisopropylethylammonium perrfluorophenyloxide	19.5-19.9 min
Reagent **9**	22.2 min

25a (Entry 16, Table 2)

Following the general procedure for Cysteine-Lysine stapling on analytical scale **25a** (retention time 13.2 min) was obtained in 94% relative absorbance after 24 hours.

HRMS (nanochip-ESI/LTQ-Orbitrap) m/z: $[M+H_2]^{+2}$ Calcd for $C_{87}H_{134}N_{24}O_{25}S_2^{+2}$ 989.4691; Found 989.4655.

HPLC-UV Chromatograms (210 nm) of the crude mixture (only one of the duplicates is shown):

Side product: 13.9 min exhibiting the same m/z as the expected product.

30 min:

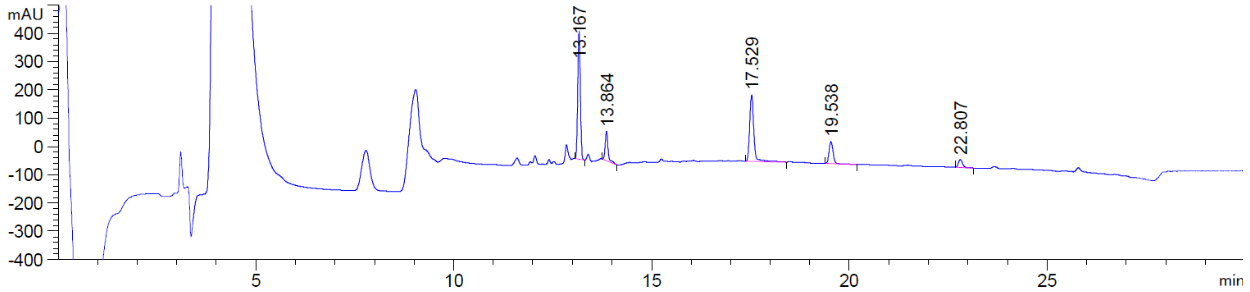

24 hours:

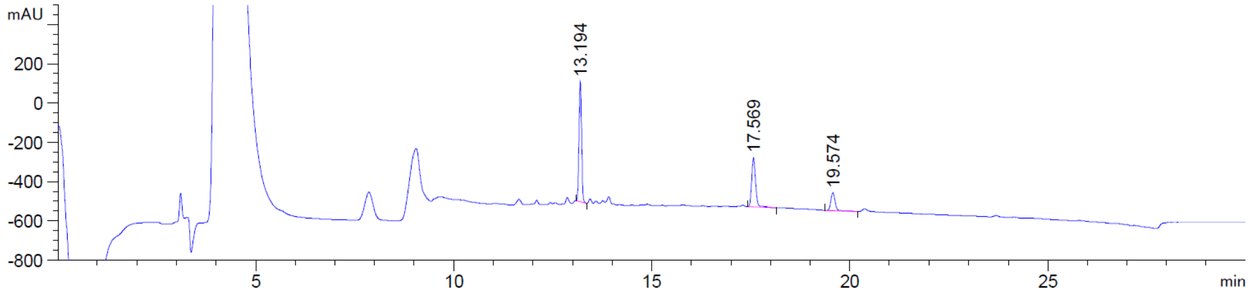

MS/MS Characterization:

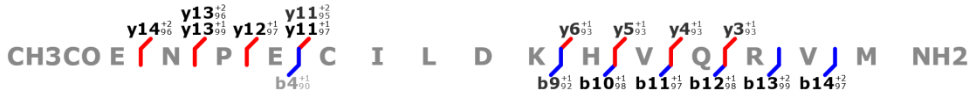

b4 (1.3), ***b9*** *(1.2)*, ***b10*** *(7.6)*, ***b11*** *(11.6)*, ***b12*** *(5.2)*, ***b13*** *(27.3)*, ***b14*** *(101.0)*, **y3** (3.1), **y4** (11.0), **y5** (7.3), **y6** (7.6), ***y11*** *(5.2)*, ***y11*** *(1.8)*, ***y12*** *(0.9)*, ***y13*** *(20.1)*, ***y13*** *(1.1)*, ***y14*** *(14.6)*.

Following the general procedure for isolation of Cysteine-Lysine stapled peptides, Ac-ENPECILDKHVQRVM-NH_2 (**24**) (5.6 mg, 2.6 µmol) together with perfluorophenyl 4-((3-oxo-1λ^3-benzo[d][1,2]iodaoxol-1(3H)-yl)ethynyl)benzoate (**9a**) afforded the product (**25b**) (2.9 mg, 1.3 µmol, 52%) as a white amorphous solid (retention time 13.1 min).

HPLC-UV chromatogram at 210 nm of the isolated product:

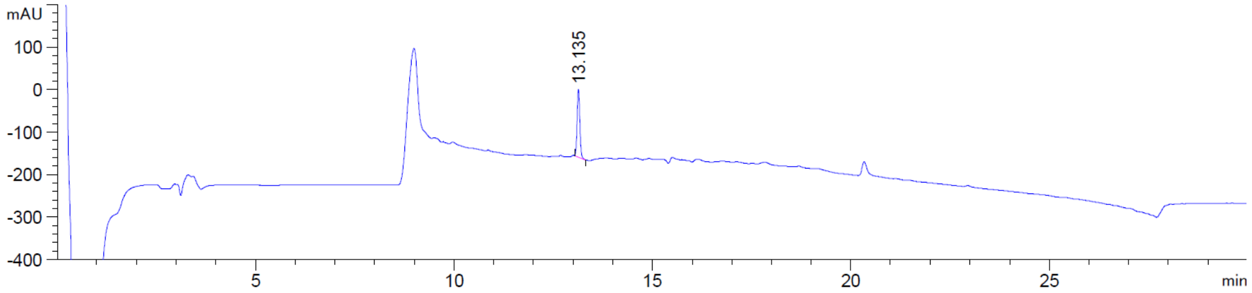

27a (Entry 17, Table 2)

Following the general procedure for Cysteine-Lysine stapling on analytical scale **27a** (retention time 11.6 min) was obtained in 114% relative absorbance after 30 minutes.

HRMS (nanochip-ESI/LTQ-Orbitrap) m/z: $[M + H_2]^{+2}$ Calcd for $C_{89}H_{135}N_{29}O_{29}S^{+2}$ 1052.9845; Found 1052.9874.

HPLC-UV Chromatogram (210 nm) of the crude mixture (only one of the duplicates is shown):

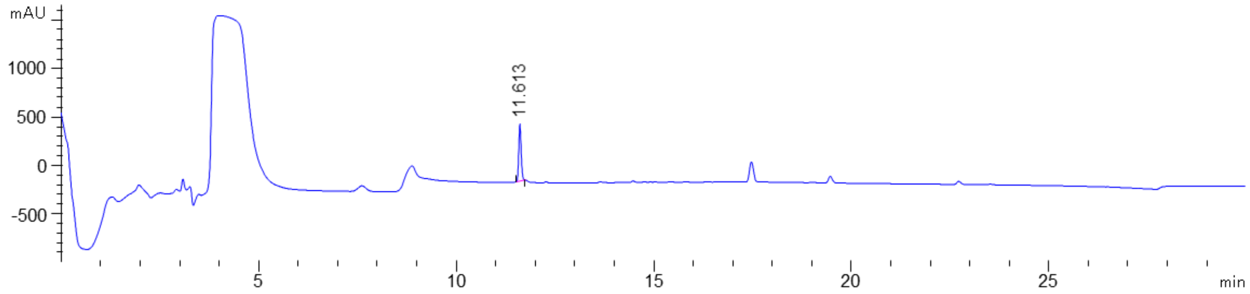

MS/MS Characterization:

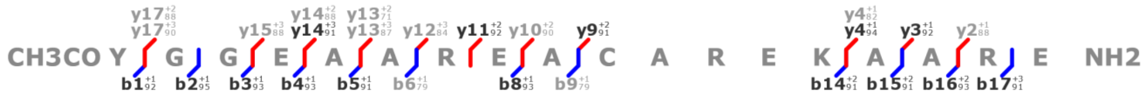

b1 (1.5), **b2** (6.2), **b3** (4.9), **b4** (5.3), **b5** (3.2), **b6** (0.9), **b8** (29.5), **b9** (6.0), ***b14*** *(5.0)*, ***b15*** *(9.1)*, **b15** (0.3), ***b16*** *(14.9)*, ***b17*** *(38.3)*, ***b17*** *(0.4)*, **y2** (1.3), **y3** (4.0), **y4** (10.9), ***y4*** *(1.5)*, ***y9*** *(11.8)*, ***y10***

(81.3), ***y11*** *(26.6)*, ***y12*** *(1.4)*, ***y12*** *(0.5)*, ***y13*** *(2.2)*, ***y13*** *(0.7)*, ***y14*** *(53.3)*, ***y14*** *(0.8)*, ***y15*** *(1.5)*, ***y17*** *(8.3)*, ***y17*** *(1.4)*.

Following the general procedure for isolation of Cysteine-Lysine stapled peptides, Ac-YGGEAAREACAREKAARE-NH_2 (**26**) (5.7 mg, 2.3 µmol) together with perfluorophenyl 4-((3-oxo-1λ^3-benzo[d][1,2]iodaoxol-1(3H)-yl)ethynyl)benzoate (**9a**) afforded the product (**27a**) (3.7 mg, 1.5 µmol, 65%) as a white amorphous solid (retention time 11.7 min).

HPLC-UV chromatogram at 210 nm of the isolated product:

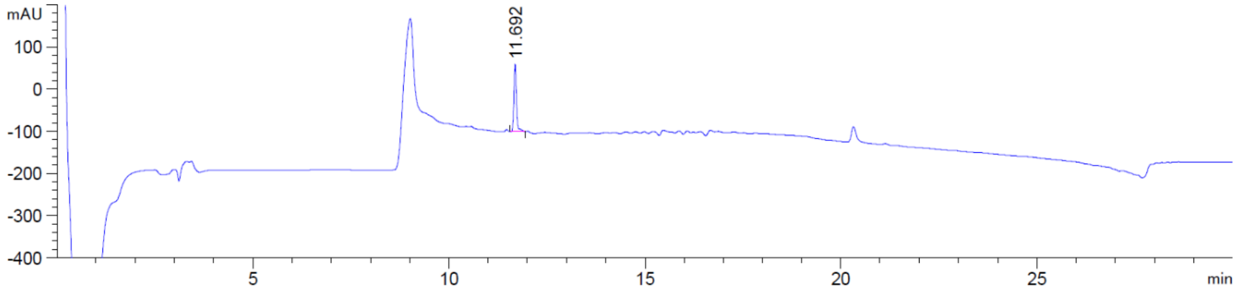

29a (Entry 18, Table 2)

Following the general procedure for Cysteine-Lysine stapling on analytical scale **29a** (retention time 15.3 min) was obtained in 117% relative absorbance after 30 minutes.

HRMS (nanochip-ESI/LTQ-Orbitrap) m/z: $[M+H_2]^{+2}$ Calcd for $C_{99}H_{146}N_{28}O_{26}S^{+2}$ 1087.5336; Found 1087.5347.

HPLC-UV Chromatogram (210 nm) of the crude mixture (only one of the duplicates is shown):

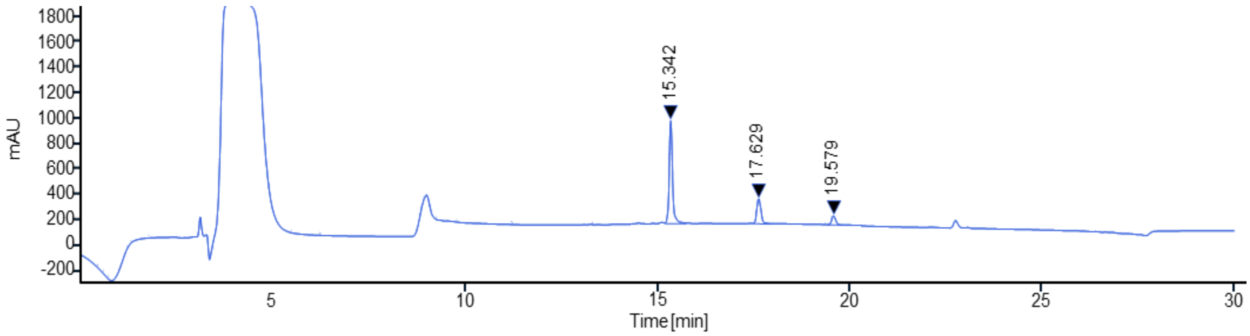

MS/MS Characterization:

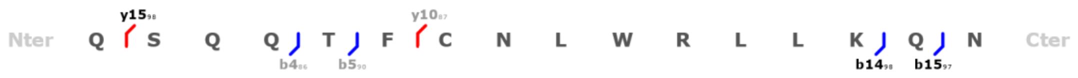

b4 (0.5), **b5** (1.3), ***b14*** *(18.7)*, ***b14*** *(86.2)*, ***b15*** *(7.6)*, ***y10*** *(0.6)*, ***y15*** *(1.0)*.

Following the general procedure for isolation of Cysteine-Lysine stapled peptides, Ac-QSQQTFCNLWRLLKQN-NH_2 (**28**) (4.3 mg, 1.9 µmol) together with perfluorophenyl 4-((3-oxo-1λ^3-benzo[d][1,2]iodaoxol-1(3H)-yl)ethynyl)benzoate (**9a**) afforded the product (**29a**) (3.8 mg, 1.7 µmol, 87%) as a white amorphous solid (retention time 15.5 min).

HPLC-UV chromatogram at 210 nm of the isolated product:

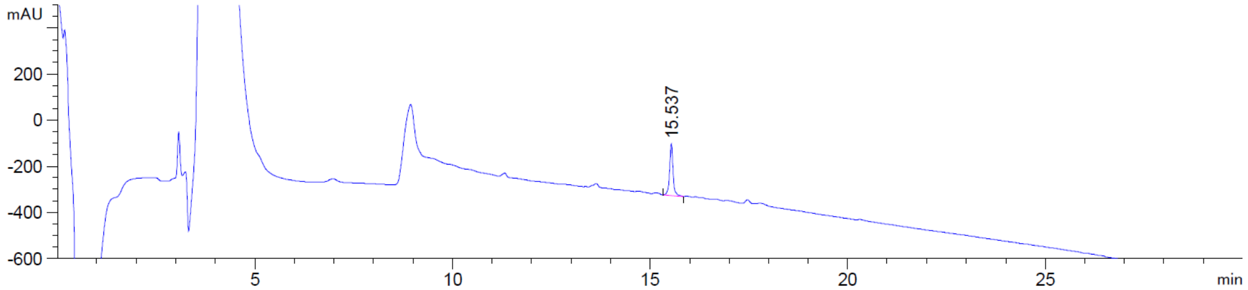

31a (Entry 19, Table 2)

Following the general procedure for Cysteine-Lysine stapling on analytical scale **31a** (retention time 11.6 min) was obtained in 63% relative absorbance after 30 minutes.

HRMS (nanochip-ESI/LTQ-Orbitrap) m/z: $[M+H_2]^{+2}$ Calcd for $C_{72}H_{96}N_{20}O_{19}S_2^{+2}$ 804.3296; Found 804.3296.

HPLC-UV Chromatograms (210 nm) of the crude mixture (only one of the duplicates is shown):

30 min:

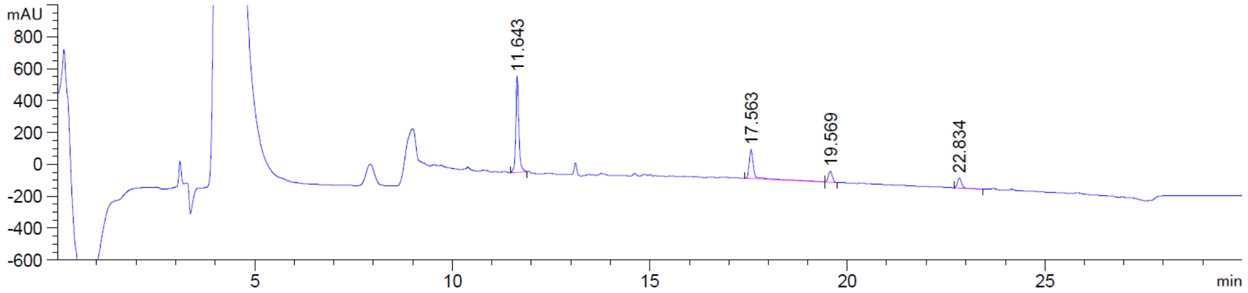

MS/MS Characterization:

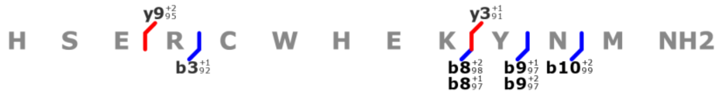

b3 (0.6), ***b8*** *(12.2)*, ***b8*** *(7.9)*, ***b9*** *(17.2)*, ***b9*** *(3.7)*, ***b10*** *(96.4)*, **y3** (0.8), ***y9*** *(1.8)*.

33a and 33a` (Entry 20, Table 2)

Following the general procedure for Cysteine-Lysine stapling on analytical scale **33a and 33a`** (retention time 11.2 and 11.8 min) were obtained in 69% and 23% relative absorbance after 24 hours.

33a: **HRMS** (nanochip-ESI/LTQ-Orbitrap) m/z: $[M+H_2]^{+2}$ Calcd for $C_{67}H_{90}N_{20}O_{18}S^{+2}$ 747.3226; Found 747.3241.

33a`: **HRMS** (nanochip-ESI/LTQ-Orbitrap) m/z: $[M+H_2]^{+2}$ Calcd for $C_{67}H_{90}N_{20}O_{18}S^{+2}$ 747.3226; Found 747.3195.

HPLC-UV Chromatograms (210 nm) of the crude mixture (only one of the duplicates is shown):

30 min:

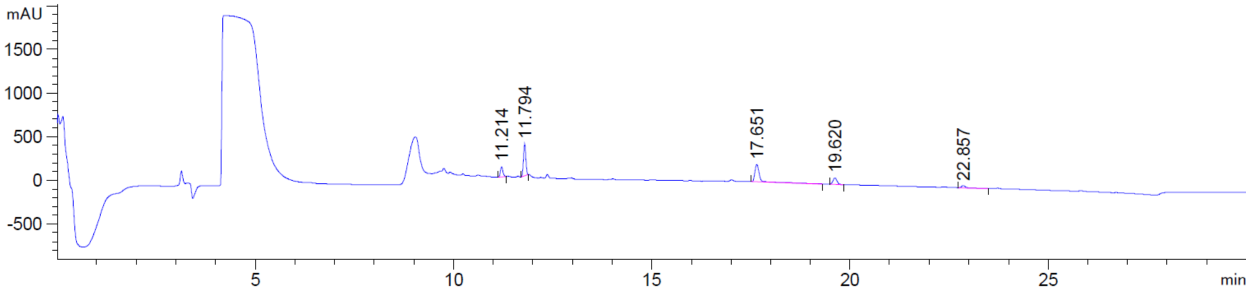

MS/MS Characterization of the major product:

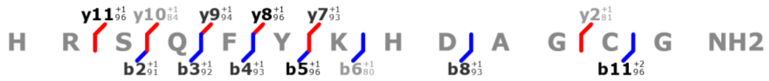

b2 (1.0), **b3** (5.5), **b4** (17.8), **b5** (59.4), **b6** (0.8), **b8*** (0.8), ***b11*** *(103.5)*, ***y2**** *(0.6)*, ***y7*** *(37.4)*, ***y8*** *(15.2)*, ***y9*** *(6.7)*, ***y10*** *(1.1)*, ***y11*** *(2.9)*.

MS/MS Characterization of the minor product:

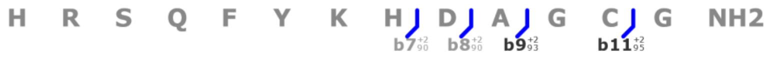

b7 *(1.8)*, ***b8*** *(0.7)*, ***b9*** *(0.6)*, ***b11*** *(67.8)*.

25b (Entry 21, Table 2)

Following the general procedure for Cysteine-Lysine stapling on analytical scale **25b** (retention time 14.0 min) was obtained in 34% relative absorbance after 30 minutes.

HRMS (nanochip-ESI/LTQ-Orbitrap) m/z: $[M+H_2]^{+2}$ Calcd for $C_{87}H_{134}N_{24}O_{25}S_2^{+2}$ 989.4691; Found 989.4698.

HPLC-UV Chromatograms (210 nm) of the crude mixture (only one of the duplicates is shown):

30 min:

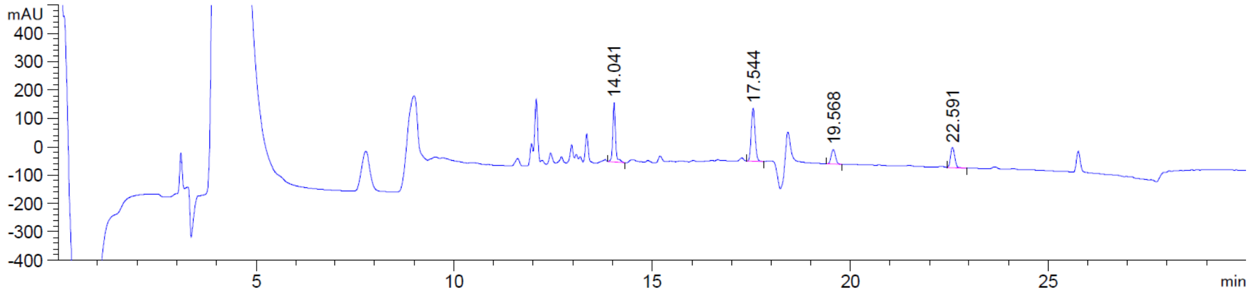

MS/MS Characterization:

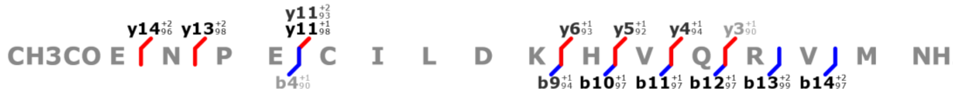

b4 (0.7), ***b9*** *(1.5)*, ***b10*** *(6.9)*, ***b11*** *(10.4)*, ***b12*** *(4.5)*, ***b13*** *(30.5)*, ***b14*** *(102.4)*, **y3** (2.7), **y4** (9.9), **y5** (8.1), **y6** (10.1), ***y11*** *(1.9)*, ***y11*** *(0.7)*, ***y13*** *(6.5)*, ***y14*** *(5.6)*.

Following the general procedure for isolation of Cysteine-Lysine stapled peptides, Ac-ENPECILDKHVQRVM-NH_2 (**24**) (5.4 mg, 2.5 µmol) together with perfluorophenyl 3-((3-oxo-1λ^3-benzo[d][1,2]iodaoxol-1(3H)-yl)ethynyl)benzoate (**9b**) afforded the product (**25b**) (0.4 mg, 0.2 µmol, 7% with 25% inseparable impurity) as a white amorphous solid (retention time of the product 14.0 min, of the impurity 13.5 min).

HPLC-UV chromatogram at 210 nm of the isolated product:

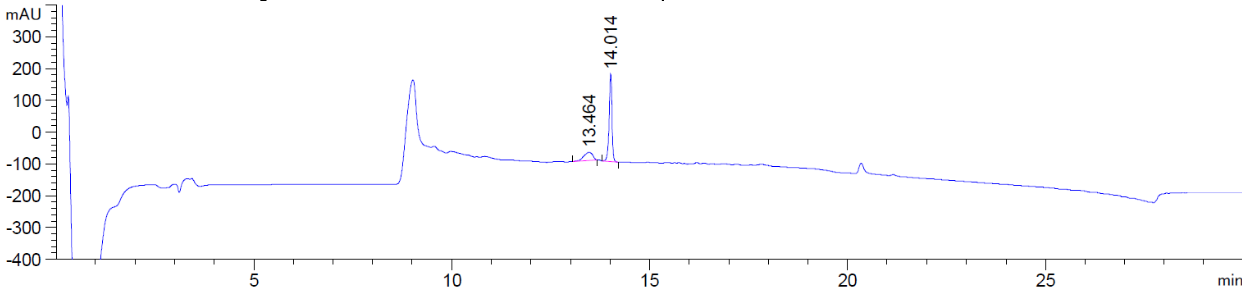

27b (Entry 22, Table 2)

Following the general procedure for Cysteine-Lysine stapling on analytical scale **27b** (retention time 12.1 min) was obtained in 77% relative absorbance after 30 minutes.

HRMS (nanochip-ESI/LTQ-Orbitrap) m/z: $[M + H_2]^{+2}$ Calcd for $C_{89}H_{135}N_{29}O_{29}S^{+2}$ 1052.9845; Found 1052.9847.

HPLC-UV Chromatogram (210 nm) of the crude mixture (only one of the duplicates is shown):

Retention time of a S/N attack on the activated ester: 11.4 min

Retention time of the oxidized peptide: 11.0 min

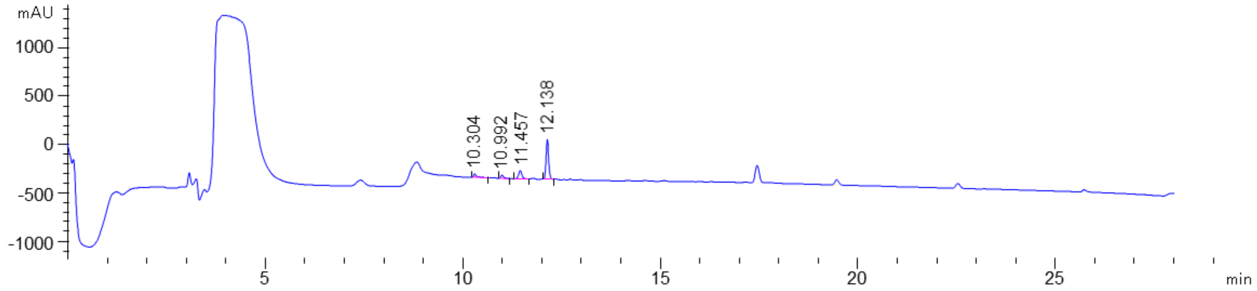

MS/MS Characterization:

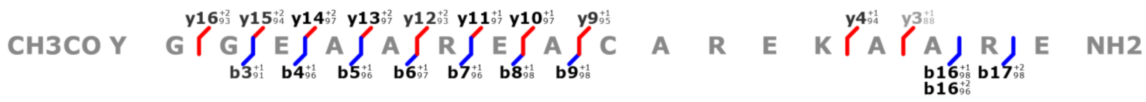

b3 (0.6), **b4** (3.9), **b5** (5.9), **b6** (3.6), **b7** (11.0), **b8** (9.4), **b9** (18.1), ***b16*** *(1.0)*, ***b16*** *(0.7)*, ***b17*** *(26.3)*, **y3** (0.8), **y4** (1.7), ***y9*** *(5.9)*, ***y10*** *(4.1)*, ***y11*** *(2.4)*, ***y12*** *(0.7)*, ***y13*** *(0.7)*, ***y14*** *(12.8)*, ***y15*** *(0.6)*, ***y16*** *(0.8)*.

Following the general procedure for isolation of Cysteine-Lysine stapled peptides, Ac-YGGEAAREACAREKAARE-NH_2 (**26**) (7.3 mg, 3.0 µmol) together with perfluorophenyl 3-((3-oxo-$1\lambda^3$-benzo[d][1,2]iodaoxol-1(3H)-yl)ethynyl)benzoate (**9b**) afforded the product (**27b**) (3.2 mg, 1.3 µmol, 44%) as a white amorphous solid (retention time 12.2 min).

HPLC-UV chromatogram at 210 nm of the isolated product:

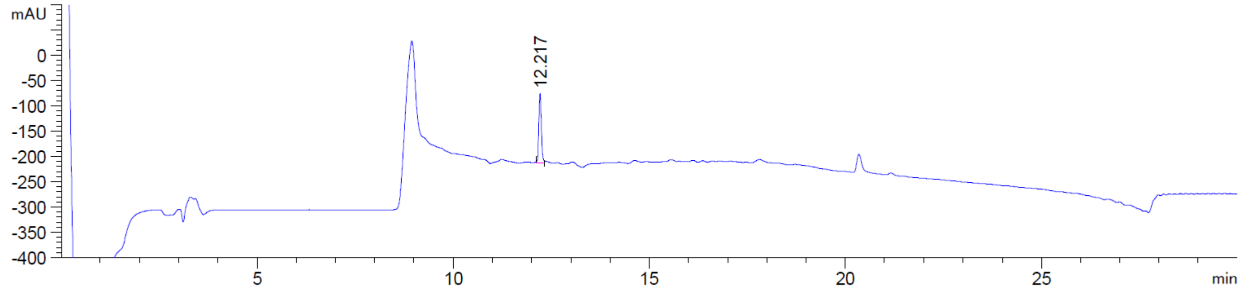

29b (Entry 23, Table 2)

Following the general procedure for Cysteine-Lysine stapling on analytical scale **29b** (retention time 15.9 min) was obtained in 110% relative absorbance after 30 minutes.

HRMS (nanochip-ESI/LTQ-Orbitrap) m/z: $[M+H_2]^{+2}$ Calcd for $C_{99}H_{147}N_{28}O_{26}S^{+2}$ 1088.0376; Found 1088.0413.

HPLC-UV Chromatograms (210 nm) of the crude mixture (only one of the duplicates is shown):

30 min:

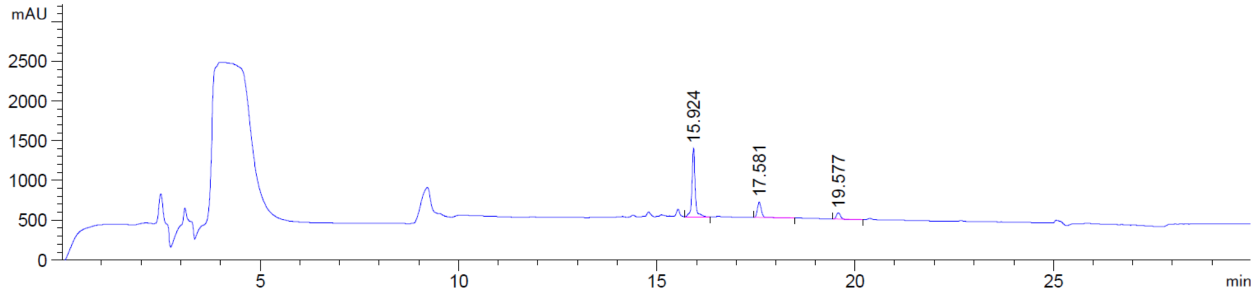

MS/MS Characterization:

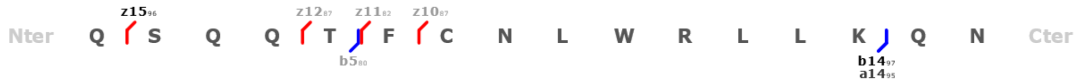

a14 *(4.9)*, **b5** (0.7), ***b14*** *(83.0)*, ***b14*** *(7.3)*, ***z10*** *(1.1)*, ***z11*** *(0.5)*, ***z12*** *(0.7)*, ***z15*** *(3.3)*.

Following the general procedure for isolation of Cysteine-Lysine stapled peptides, Ac-QSQQTFCNLWRLLKQN-NH_2 (**28**) (4.9 mg, 2.2 µmol) together with perfluorophenyl 3-((3-oxo-$1\lambda^3$-benzo[d][1,2]iodaoxol-1(3H)-yl)ethynyl)benzoate (**9b**) afforded the product (**29b**) (2.7 mg, 1.2 µmol, 55%) as a white amorphous solid (retention time 15.8 min).

HPLC-UV chromatogram at 210 nm of the isolated product:

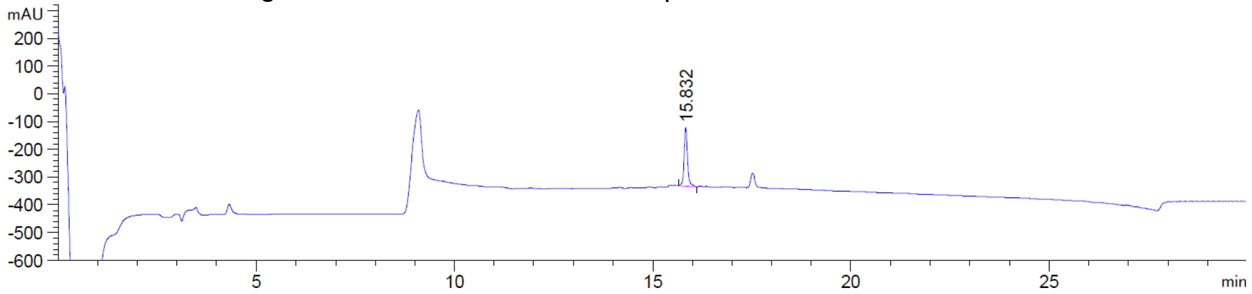

31b (Entry 24, Table 2)

Following the general procedure for Cysteine-Lysine stapling on analytical scale **31b** (retention time 12.1 min) was obtained in 20% relative absorbance after 30 minutes.

HRMS (nanochip-ESI/LTQ-Orbitrap) m/z: $[M+H_2]^{+2}$ Calcd for $C_{72}H_{96}N_{20}O_{19}S_2^{+2}$ 804.3296; Found 804.3290.

HPLC-UV Chromatogram (210 nm) (only one of the duplicates is shown):

Retention time stapled product: 12.1 min

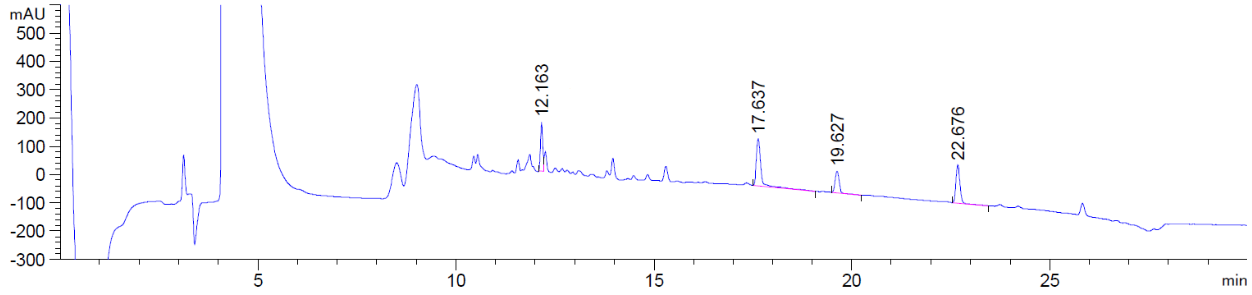

MS/MS Characterization:

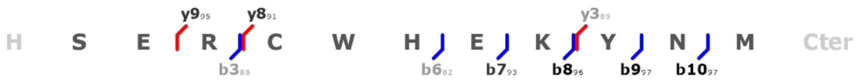

b3 (0.7), ***b6**** *(0.7)*, ***b7**** *(1.1)*, ***b8*** *(9.5)*, ***b8*** *(9.7)*, ***b9*** *(3.3)*, ***b9*** *(13.2)*, ***b10*** *(72.1)*, **y3** (1.4), ***y8*** *(0.6)*, ***y9*** *(2.0)*.

33b (Entry 25, Table 2)

Following the general procedure for Cysteine-Lysine stapling on analytical scale **33b** (retention time 12.0 min) was obtained in 22% relative absorbance after 30 minutes.

HRMS (nanochip-ESI/LTQ-Orbitrap) m/z: $[M + H_3]^{+3}$ Calcd for $C_{67}H_{91}N_{20}O_{18}S^{+3}$ 498.5508; Found 498.5514.

HPLC-UV Chromatograms (210 nm) (only one of the duplicates is shown):

30 min:

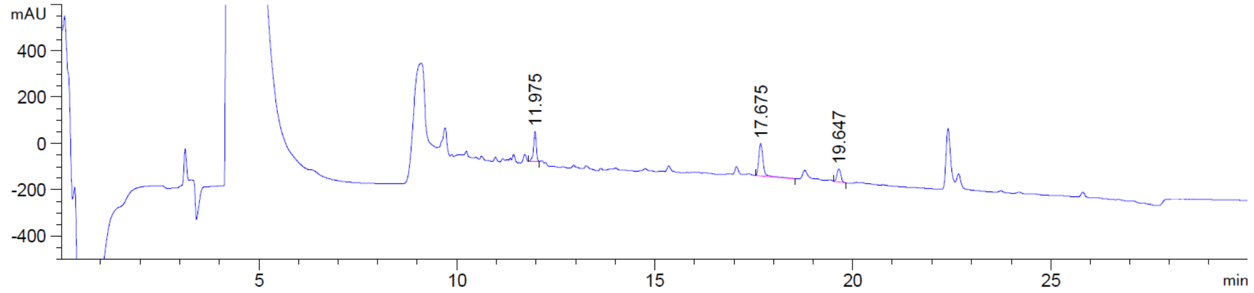

MS/MS Characterization:

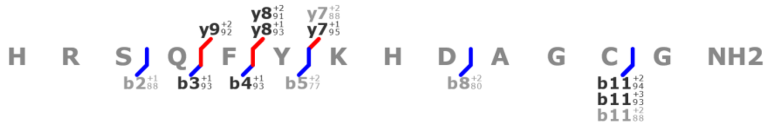

b2 (0.9), **b3** (16.2), **b4** (26.9), **b5** (0.8), **b8*** (1.0), ***b11*** *(52.4)*, ***b11*** *(26.5)*, **b11** (2.1), ***y7*** *(14.1)*, ***y7*** *(4.1)*, ***y8*** *(5.0)*, ***y8*** *(4.2)*, ***y9*** *(3.5)*.

25c (Entry 26, Table 2)

Following the general procedure for Cysteine-Lysine stapling on analytical scale **25c** (retention time 13.7 min) was obtained in 101% relative absorbance after 30 minutes.

HRMS (nanochip-ESI/LTQ-Orbitrap) m/z: $[M+H_2]^{+2}$ Calcd for $C_{87}H_{134}N_{24}O_{25}S_2^{+2}$ 989.4691; Found 989.4713.

HPLC-UV Chromatograms (210 nm) (only one of the duplicates is shown):

New product appears at 13.1 min

30 min:

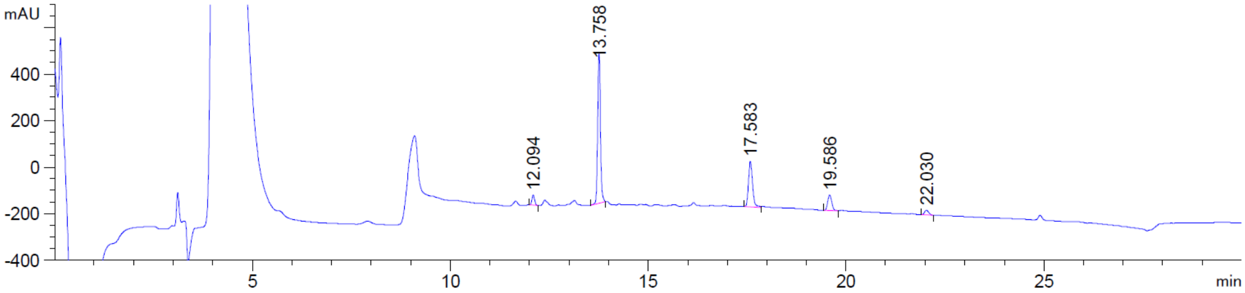

24 hours:

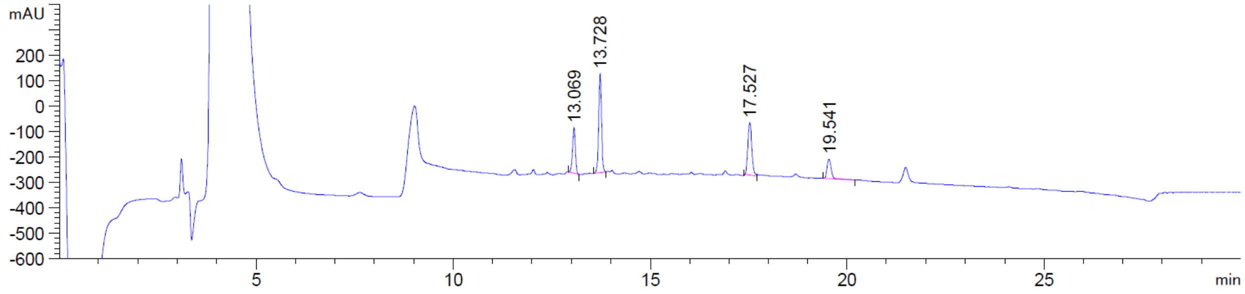

MS/MS Characterization:

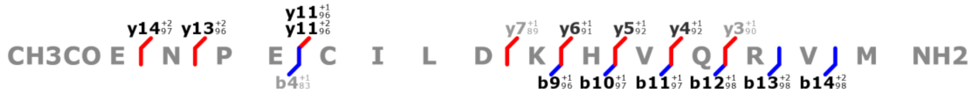

b4 (0.7), ***b9*** *(3.1)*, ***b10*** *(6.0)*, ***b11*** *(8.5)*, ***b12*** *(4.2)*, ***b13*** *(31.3)*, ***b14*** *(100.3)*, **y3** (2.3), **y4** (7.0) **y5** (7.2), **y6** (9.2), ***y7**** *(1.1)*, ***y11*** *(1.8)*, ***y11*** *(1.3)*, ***y13*** *(6.9)*, ***y14*** *(7.2)*.

27c (Entry 27, Table 2)

Following the general procedure for Cysteine-Lysine stapling on analytical scale **27c** (retention time 12.0 min) was obtained in 118% relative absorbance after 30 minutes.

HRMS (nanochip-ESI/LTQ-Orbitrap) m/z: $[M+H_3]^{+3}$ Calcd for $C_{89}H_{136}N_{29}O_{29}S^{+3}$ 702.3254; Found 702.3274

HPLC-UV Chromatograms (210 nm) of the crude mixture (only one of the duplicates is shown):

New products appear at: 11.2 and 12.1 min

HPLC-UV chromatogram at 210 nm of the crude reaction mixture after 30 min:
:

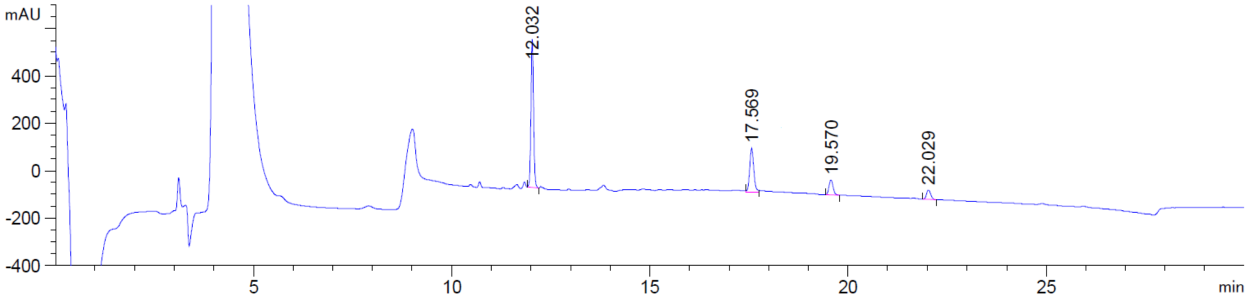

HPLC-UV chromatogram at 210 nm of the crude reaction mixture after 24 hours:

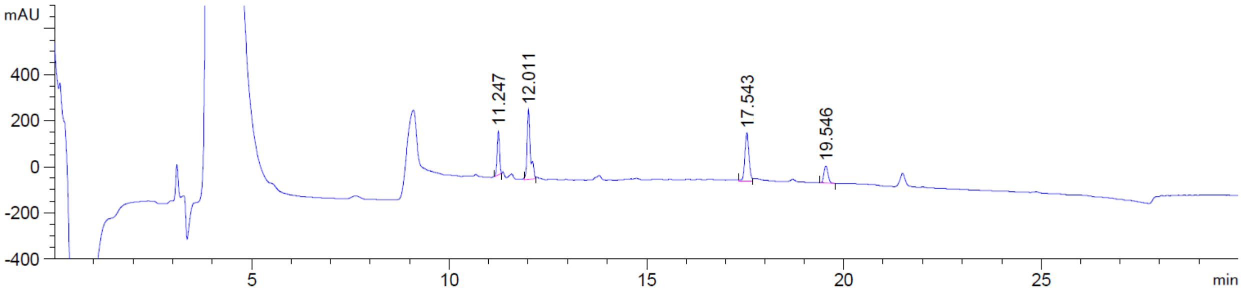

MS/MS Characterization:

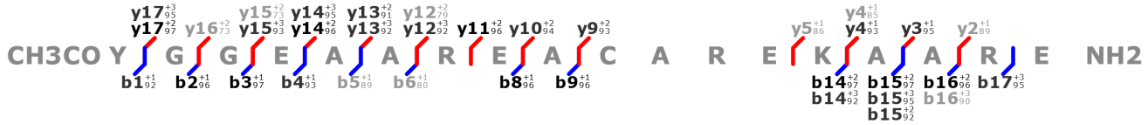

b1 (1.8), **b2** (7.5), **b3** (5.9), **b4** (6.0), **b5** (3.6), **b6** (1.0), **b8** 42.5, **b9** (8.2), ***b14*** *(6.5)*, ***b14*** *(0.8)*, ***b15*** *(10.6)*, ***b15*** *(1.0)*, ***b15*** *(0.5)*, ***b16*** *(13.1)*, ***b16*** *(0.9)*, ***b17*** *(40.3)*, **y2** (2.2), **y3** (6.8), **y4** (14.8), ***y4*** *(3.0)*, **y5*** (2.1), ***y9*** *(11.5)*, ***y10*** *(101.3)*, ***y11*** *(20.2)*, ***y12*** *(1.1)*, ***y12*** *(1.1)*, ***y13*** *(1.8)*, ***y13*** *(1.3)*, ***y14*** *(52.1)* ***y14*** *(2.5)*, ***y15*** *(1.5)*, ***y15*** *(1.2)*, ***y16*** *(5.1)*, ***y17*** *(9.4)*, ***y17*** *(6.0)*.

29c (Entry 28, Table 2)

Following the general procedure for Cysteine-Lysine stapling on analytical scale **29c** (retention time 16.1 min) was obtained in 123% relative absorbance after 30 minutes.

HRMS (nanochip-ESI/LTQ-Orbitrap) m/z: $[M+H_2]^{+2}$ Calcd for $C_{99}H_{147}N_{28}O_{26}S^{+2}$ 1088.0376; Found 1088.0413.

HPLC-UV Chromatograms (210 nm) of the crude mixture (only one of the duplicates is shown):

New product appears at 15.0 min

HPLC-UV chromatogram at 210 nm of the crude reaction mixture after 30 min:

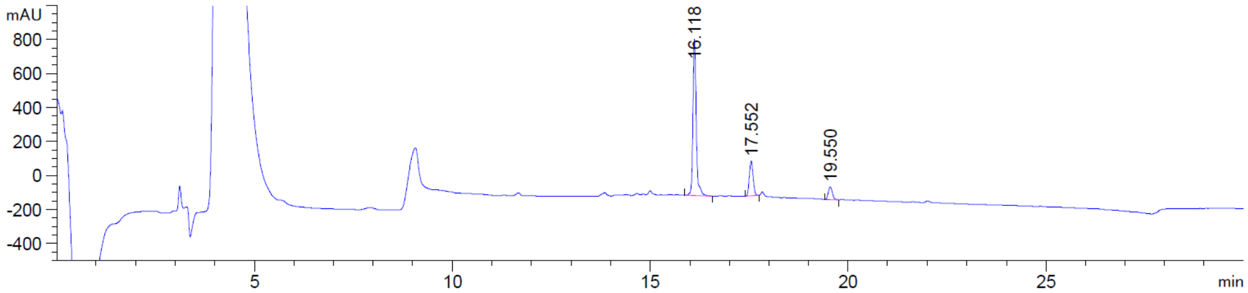

HPLC-UV chromatogram at 210 nm of the crude reaction mixture after 24 hours:

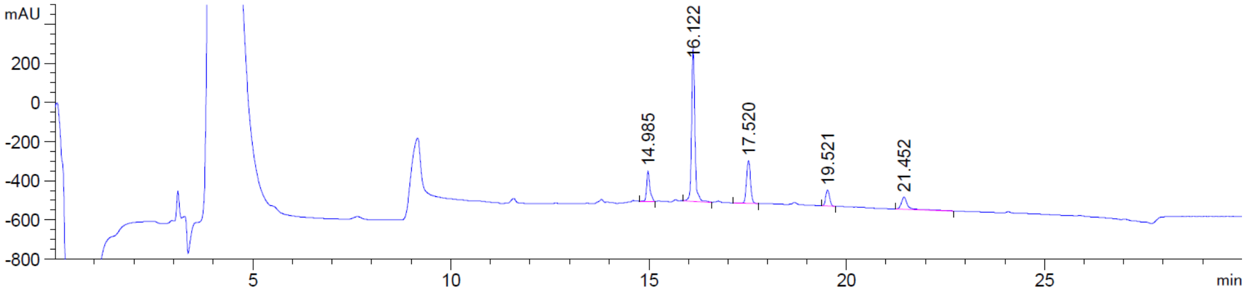

MS/MS Characterization:

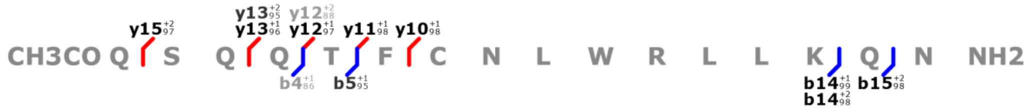

b4 (2.1), **b5** (4.6), ***b14*** *(99.0)*, ***b14*** *(10.2)*, ***b15*** *(80.3)*, ***y10*** *(6.6)*, ***y11*** *(4.2)*, ***y12*** *(4.7)*, ***y12*** *(0.7)*, ***y13*** *(1.6)*, ***y13*** *(1.4)*, ***y15*** *(11.7)*.

11. Kinetic Data for the reaction of the Cysteine-Lysine system

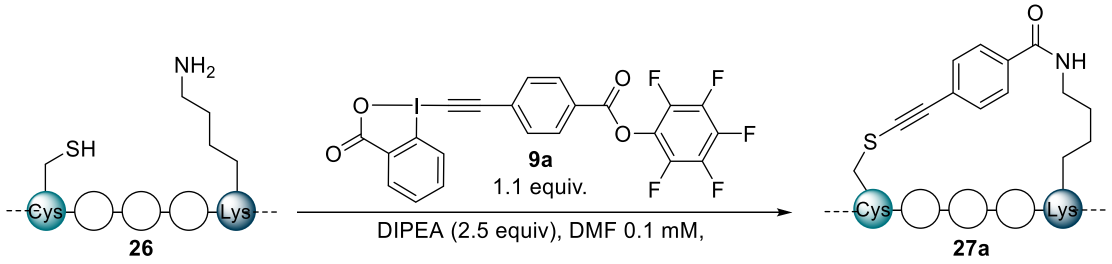

Sequence: Ac-YGGEAAREACAREKAARE-NH_2

The linear peptide Ac-YGGEAAREACAREKAARE-NH_2 (**26**) (0.61 mg) was weighed in a 1.5 mL Eppendorf tube. DMF (0. 25 mL) that had been bubbled with nitrogen for 30 minutes was used to prepare a 1 mM solution. The solution was further diluted to 0.1 mM and 100 µL were placed in a 96-well plate well. To this solution a 0.02 M solution of DIPEA in DMF (1.25 µL, 2.5 equiv.) was added and directly after a 0.01 M solution of stapling reagent (**27a**) in DMF (1.1 µL, 1.1 equiv.) was added. The mixture was mixed with the micropipette a few times before starting the injection (around 5 seconds after mixing).

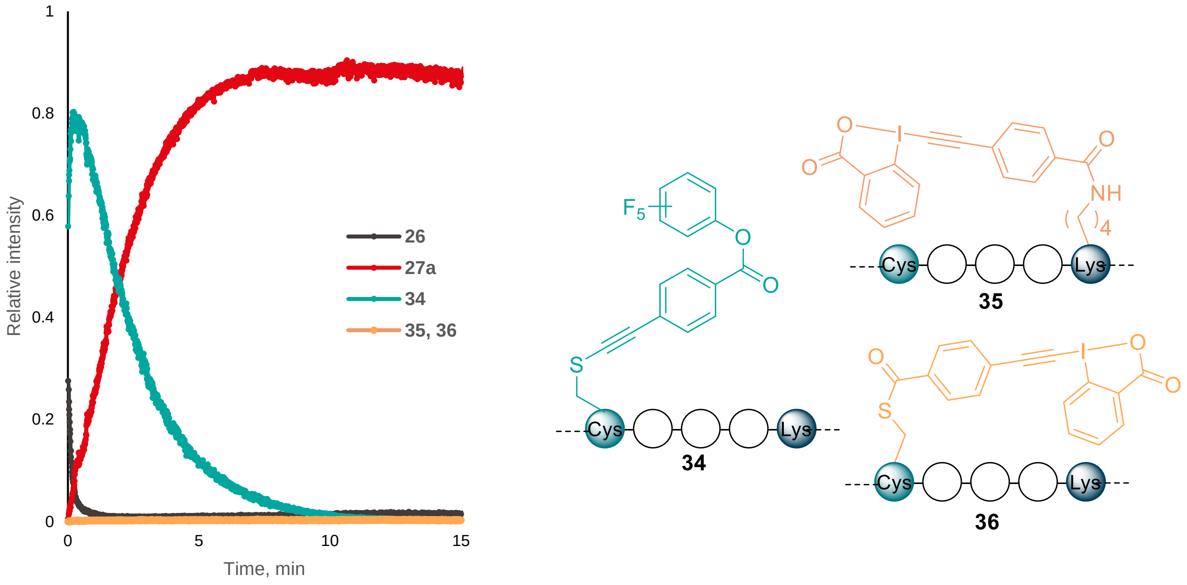

Figure S3: Kinetic experiment based on the ions detected of the linear peptide (**26**), the stapled product (**27a**) and the potential Lysine and Cysteine attack resulting intermediates (**35**,**36** and **34**, respectively). The total ion counts were normalized to 1.

12. Cysteine-Lysine Stapling in presence of additional Lys

Retention times of the common by-products:

2-iodobenzoic acid (**51**)	17.5-17.6 min
Diisopropylethylammonium perrfluorophenyloxide	19.5-19.9 min
Reagent **9**	22.2 min

a) ivDde protected Lys

ivDde-26a` (Entry 1, Table 3)

Following the General Procedure for Cysteine-Lysine stapling on analytical scale using **ivDde-26a** and **9a** the product **ivDde-26a`** was obtained in 117% relative absorbance (13.1 min, 2900 and 2365 mAu) after 30 minutes, addition of 35wt% hydrazine in water to the crude mixture (2%) provided **26a`** after 30 minutes in 84% relative absorbance (compared to **ivDde-26a**) and 97% relative absorbance (compared to **26a**) (11.0 min, 2069 and 1695 mAu).

ivDde-26a`

HRMS (nanochip-ESI/LTQ-Orbitrap) m/z: $[M + H_2]^{+2}$ Calcd for $C_{103}H_{158}N_{30}O_{29}S^{+2}$ 1155.5760; Found 1155.5798.
HPLC-UV Chromatogram (210 nm) of the crude mixture (only one of the duplicates is shown):

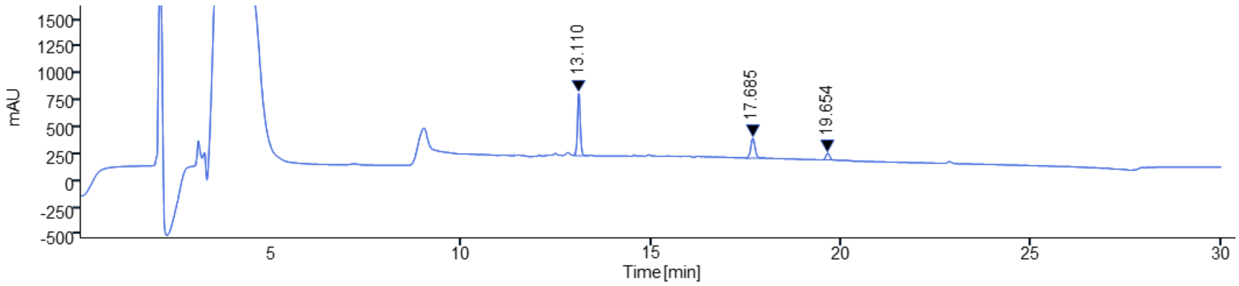

26a`

HRMS (nanochip-ESI/LTQ-Orbitrap) m/z: $[M + H_2]^{+2}$ Calcd for $C_{90}H_{140}N_{30}O_{27}S^{+2}$ 1052.5107; Found 1052.5146.

HPLC-UV Chromatogram (210 nm) of the crude mixture (only one of the duplicates is shown):

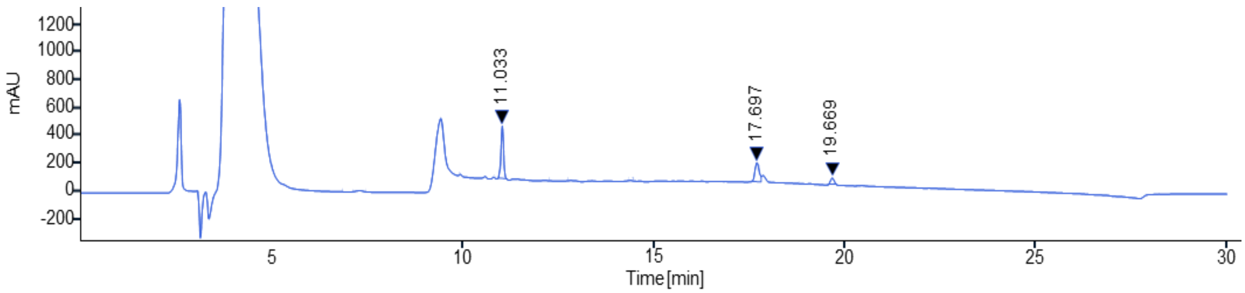

MS/MS Characterization:

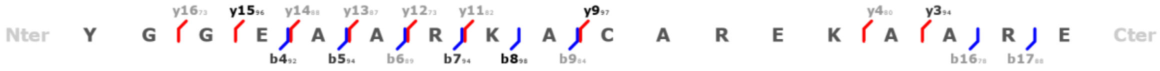

b4 (2.8), **b5** (5.2), **b6** (2.7), **b7** (9.8), **b8** (3.8), **b9** (4.9), ***b16*** (1.3), ***b17*** (43.0), **y3** (0.8), **y4** (1.4), ***y9*** (2.1), ***y11*** (3.7), ***y12*** (0.7), ***y13*** (1.0), ***y14*** (15.5), ***y15*** (1.0), ***y16*** (0.6).

ivDde-26a`` (Entry 1, Table 2)

Following the General Procedure for Cysteine-Lysine stapling on analytical scale using **ivDde-26a** and **9b** the product **26a``** was obtained in 72% relative absorbance (13.2 min, 1549 and 1673 mAu) after 30 minutes.

ivDde-26a``

HRMS (nanochip-ESI/LTQ-Orbitrap) m/z: [M + $H_2]^{+2}$ Calcd for $C_{103}H_{158}N_{30}O_{29}S^{+2}$ 1155.5760; Found 1155.5814.

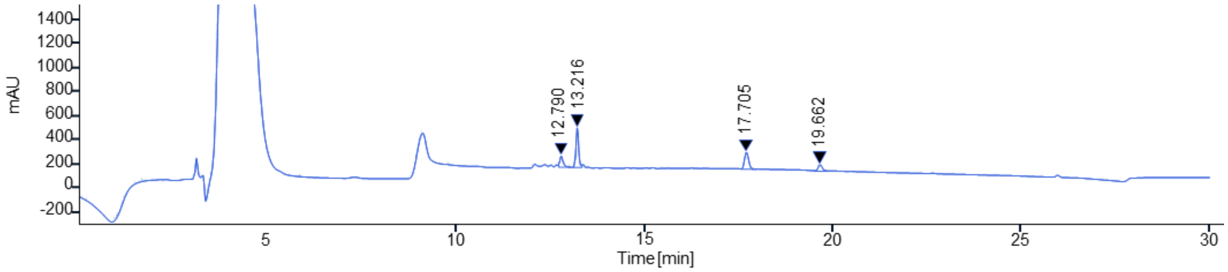

12.8 min oxidized **ivDde-26a** (550, 449 mAu, 22% rel. abs.)

After 30 minutes, addition of 35wt% hydrazine in water to the crude mixture (2%) resulted in degradation as observed by formation of white precipitate and HPLC chromatogram.

26a``

Peaks at 11.6 min (364 and 458 mAu) and 11.1 min (147 and 132 mAu) did not match the expected m/z within 5 ppm error.

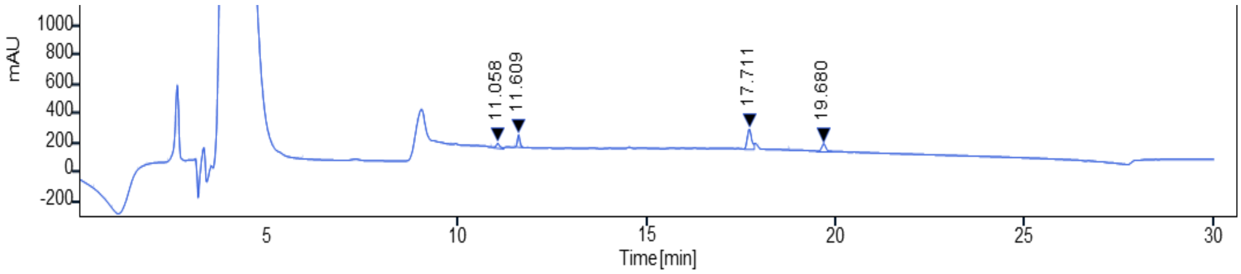

b) Unprotected Lys

26a` (Entry 3, Table 3)

Following the General Procedure for Cysteine-Lysine stapling on analytical scale using **26a** and **9a** the product **26a`** was obtained in 92% relative absorbance (11.1 min, 1785 and 1769 mAu) after 30 minutes.

HRMS (nanochip-ESI/LTQ-Orbitrap) m/z: $[M + H_2]^{+2}$ Calcd for $C_{90}H_{140}N_{30}O_{27}S^{+2}$ 1052.5107; Found 1052.5124.

HPLC-UV Chromatogram (210 nm) of the crude mixture (only one of the duplicates is shown):

11.4 min undesired stapling between Cys and introduced Lys (271, 245 mAu, 13% rel. abs.)

12.4 min over reactivity between additional Lys and excess of the reagent (295, 280 mAu, 15% rel. abs.)

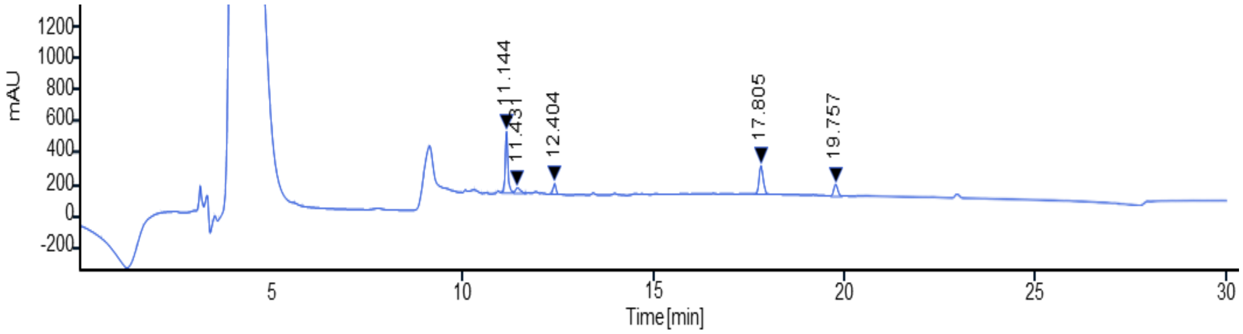

MS/MS Characterization:

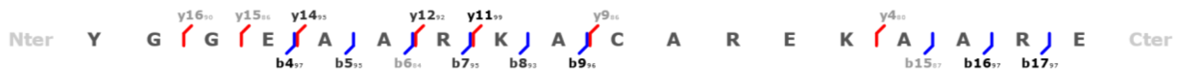

b4 (2.2), **b5** (3.0), **b6** (1.7), **b7** (4.8), **b8** (1.3), **b9** (2.1), ***b15*** (0.7), ***b16*** (1.0), ***b16*** (0.6), ***b17*** (35.5), **y4** (1.0), ***y9*** (1.7), ***y11*** (2.8), ***y12*** (0.7), ***y14*** (13.3), ***y15*** (0.5), ***y16*** (0.8).

26b` (Entry 4, Table 3)

Following the General Procedure for Cysteine-Lysine stapling on analytical scale using **26b** and **9a** the product **26b`** was obtained in 91% relative absorbance (11.0 min, 17380 and 1739 mAu) after 30 minutes.

HRMS (nanochip-ESI/LTQ-Orbitrap) m/z: $[M + H_4]^{+4}$ Calcd for $C_{92}H_{144}N_{30}O_{29}S^{+4}$ 541.2604; Found 541.2626

HPLC-UV Chromatogram (210 nm) of the crude mixture (only one of the duplicates is shown):

11.2 min undesired stapling between Cys and introduced Lys (137, 127 mAu, 7%)

12.5 min over reactivity between additional Lys and excess of the reagent (308, 316 mAu, 17%)

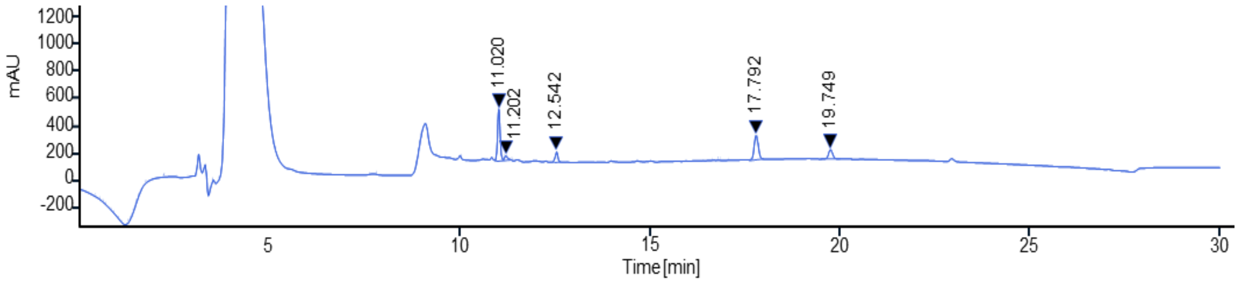

MS/MS Characterization:

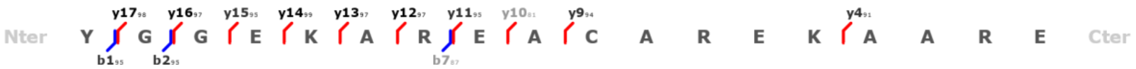

b1 (1.0), **b2** (0.7), **b7** (0.7), **y4** (0.6), ***y9*** (0.5), ***y10*** (1.4), ***y11*** (1.5), ***y12*** (0.6), ***y13*** (2.4), ***y14*** (5.5), ***y15*** (2.5), ***y16*** (1.8), ***y16*** (8.1), ***y17*** (19.9), ***y17*** (98.6).

26c` (Entry 5, Table 3)

Following the General Procedure for Cysteine-Lysine stapling on analytical scale using **26c** and **9a** the product **26c`** was obtained in 95% relative absorbance (11.3 min, 1890 and 2027 mAu) after 30 minutes.

HRMS (nanochip-ESI/LTQ-Orbitrap) m/z: $[M + H_3]^{+3}$ Calcd for $C_{95}H_{148}N_{31}O_{30}S^{+3}$ 745.0238; Found 745.0234.

HPLC-UV Chromatograms (210 nm) of the crude mixture (only one of the duplicates is shown):

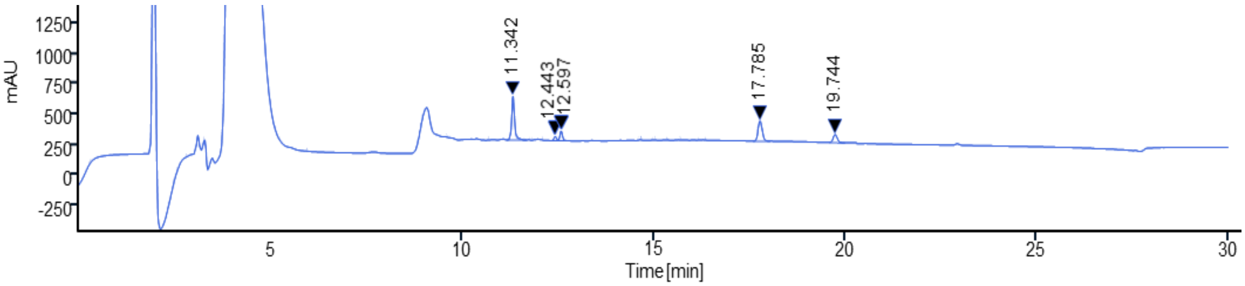

12.4 and 12.6 min over reactivity due to excess of the reagent (166, 141 and 384, 292 mAu, 7% and 16% rel. abs.)

MS/MS Characterization:

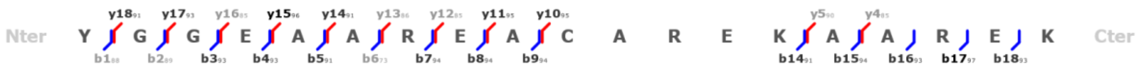

b1 (0.7), **b2** (7.7), **b3** (6.3), **b4** (7.7), **b5** (4.6), **b6** (1.1), **b7** (6.5), **b8** (45.3), **b9** (8.9), ***b14*** (1.6), ***b15*** (2.3), ***b16*** (3.1), ***b17*** (4.5), ***b17*** (13.4), ***b18*** (9.0), ***b18*** (3.0), **y4** (1.7), **y5** (3.4), ***y10*** (11.8), ***y11*** (100.4), ***y12*** (1.7), ***y13*** (1.9), ***y14*** (2.7), ***y15*** (71.1), ***y16*** (2.0), ***y17*** (4.2), ***y18*** (10.9), ***y18*** (4.7).

26a`` (Entry 6, Table 3)

Following the General Procedure for Cysteine-Lysine stapling on analytical scale using **26a** and **9b** the product **26a``** was obtained in 74% relative absorbance (11.6 min, 1378 and 1486 mAu) after 30 minutes.

HRMS (nanochip-ESI/LTQ-Orbitrap) m/z: $[M + H_4]^{+4}$ Calcd for $C_{90}H_{142}N_{30}O_{27}S^{+4}$ 526.7590; Found 526.7611.

HPLC-UV Chromatogram (210 nm) of the crude mixture (only one of the duplicates is shown):

Retention time of a S/N attack on the activated ester: 11.0 min (315 mAu)

Retention time of the oxidized peptide: 10.5 min (149 mAu)

26a: 9.9 min (147 mAu)

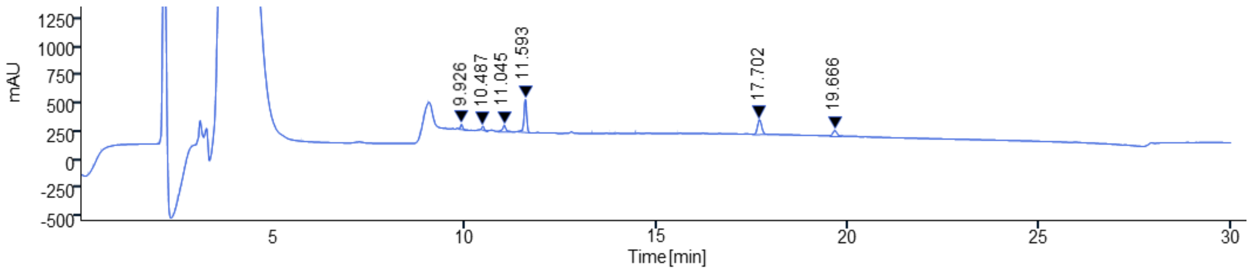

MS/MS Characterization:

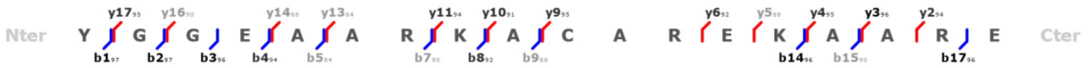

b1 (100.5), **b2** (72.5), **b3** (34.0), **b4** (12.7), **b5** (4.9), **b7** (4.0), **b8** (5.6), **b9** (1.5), ***b14*** (1.9), ***b15*** (2.3), ***b17*** (1.6), **y2** (10.6), **y3** (15.7), **y4** (36.2), **y5*** (5.1), **y6*** (5.9), ***y9*** (6.0), ***y9*** (1.6), ***y10*** (15.0), ***y10*** (1.5), ***y11*** (19.2), ***y11*** (0.6), ***y13*** (1.0), ***y14*** (6.4), ***y14*** (0.6), ***y16*** (0.8), ***y17*** (1.9), ***y17*** (1.5).

13. Stapling using the reagent 9d containing additional activated ester

27d:

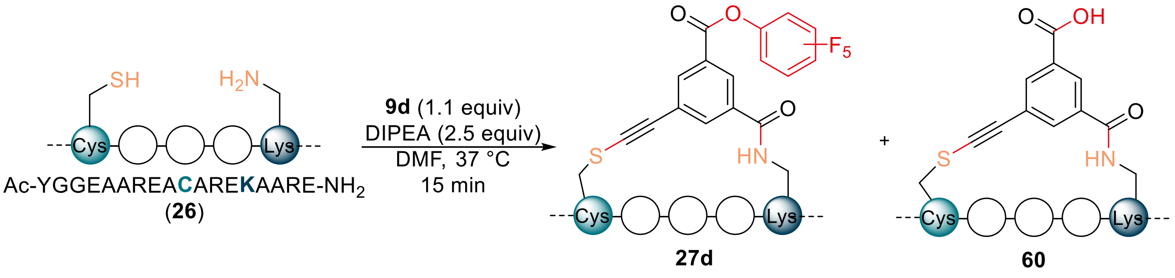

Following the general procedure for Cysteine-Lysine stapling on analytical scale using Ac-YGGEAAREACAREKAARE-NH_2 (**26**) (Absorbance area (210 nm): 2414 mAu of 1 mM solution) and reagent **9d** product **27d** (retention time 14.3 min) was obtained in 70% relative absorbance (1666 and 1713 mAu for both duplicates) after 15 minutes. Side product arising from hydrolysis of the additional activated ester (**60**) was also observed on HPLC chromatogram (retention time 11.8 min).

HRMS (nanochip-ESI/LTQ-Orbitrap) m/z: $[M]^{+3}$ Calcd for $C_{96}H_{135}F_5N_{29}O_{31}S^{+3}$ 772.3168; Found 772.3196.

HPLC-UV Chromatograms (210 nm) (only one of the duplicates is shown):

Retention times of the common by-products:

2-iodobenzoic acid (**51**)	17.5-17.6 min
Diisopropylethylammonium perrfluorophenyloxide	19.5-19.9 min
Reagent **9d**	22.2 min

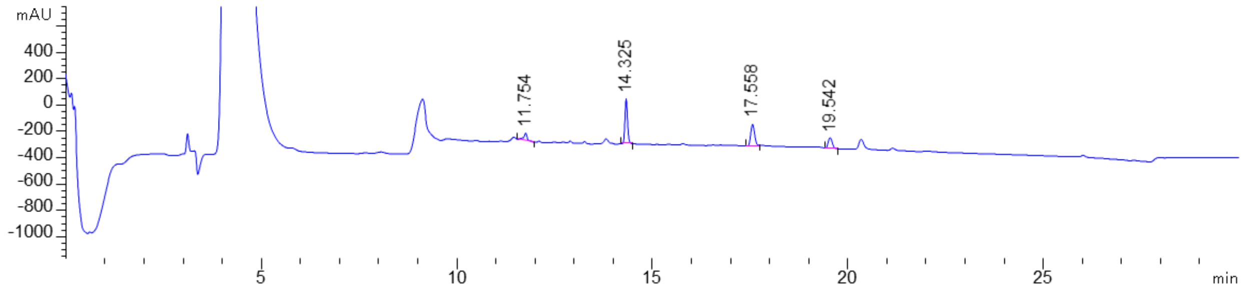

MS/MS Characterization:

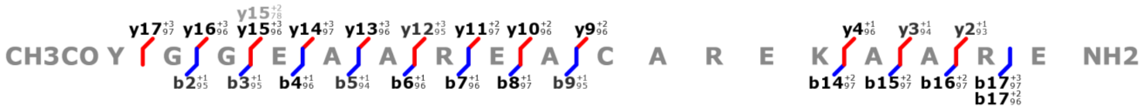

b2 (5.4), **b3** (4.8), **b4** (5.5), **b5** (3.4), **b6** (0.9), **b7** (9.8), **b8** (39.5), **b9** (7.3), ***b14*** *(7.0)*, ***b15*** *(11.4)*, ***b16*** *(18.4)*, ***b17*** *(42.4)*, ***b17*** *(0.5)*, **y2** (1.5), **y3** (4.7), **y4** (12.0), ***y9*** *(14.3)*, ***y10*** *(99.3)*, ***y11*** *(21.7)*, ***y12*** *(1.6)*, ***y13*** *(2.5)*, ***y14*** *(64.2)*, ***y15*** *(1.7)*, ***y15*** *(0.8)*, ***y16*** *(3.5)*, ***y17*** *(9.5)*.

38:

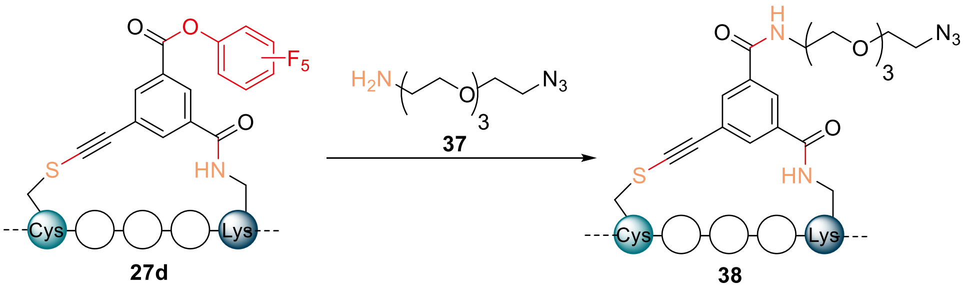

To the crude mixture of **27d**, 11-Azido-3,6,9-trioxaundecan-1-amine (**37**) in DMF (0.1 M, 1 equiv.) was added and reaction was continued to shack at 37 °C for 15 minutes. The product **38** (retention time 12.5 min) was obtained in 73% relative absorbance (relative to **27d**) (1118 and 1342 mAu for both duplicates).

HRMS (nanochip-ESI/LTQ-Orbitrap) m/z: $[M]^{+3}$ Calcd for $C_{98}H_{152}N_{33}O_{33}S^{+3}$ 783.6978; Found 783.6991.

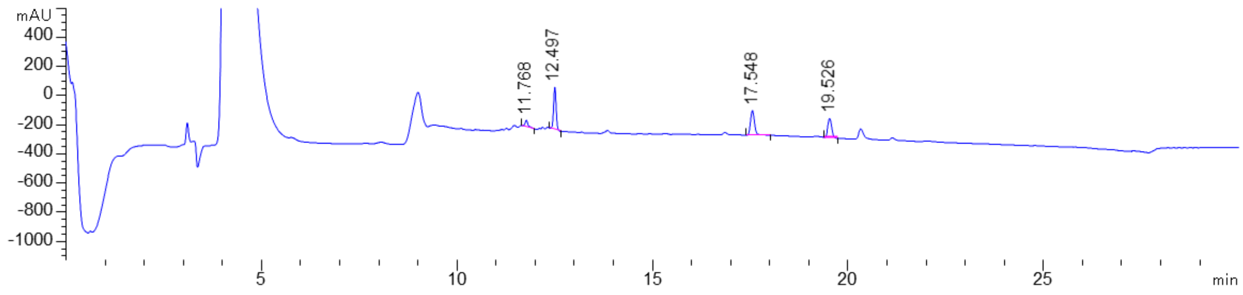

39:

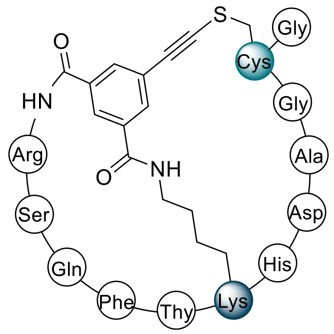

39

Following the general procedure for Cysteine-Lysine stapling on analytical scale using H-RSQFYKHDAGCG-NH_2 (**32**) (Absorbance area (210 nm): 2739 mAu of 1 mM solution) and reagent **9d** in 30 minutes product (**39**) (retention time 11.5 min) and intermediated corresponding to stapling between Cys and Lys or N-terminus were observed (retention times 11.3 and 11.7 min). After 24 hours product (**39**) was obtained in 37% relative absorption (1145 and 871 mAu for both duplicates).

HRMS (nanochip-ESI/LTQ-Orbitrap) m/z: $[M+H_2]^{+2}$ Calcd for $C_{68}H_{88}N_{20}O_{19}S^{+2}$ 760.3122; Found 760.3152.

HPLC-UV Chromatograms (210 nm) (only one of the duplicates is shown):

30 min:

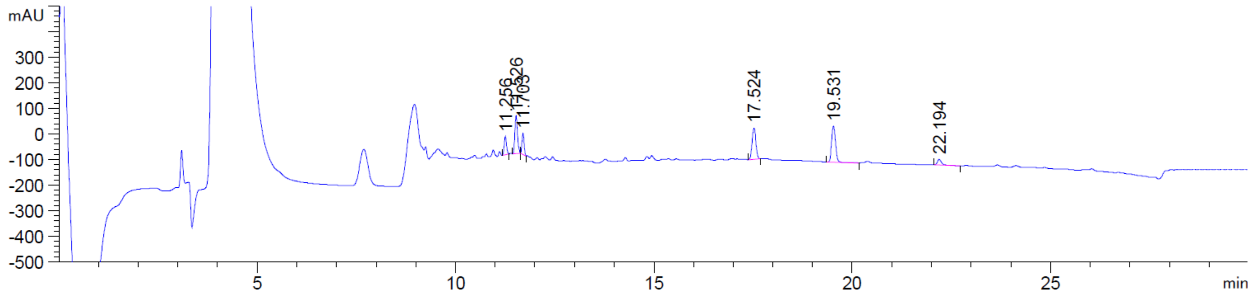

24 hours:

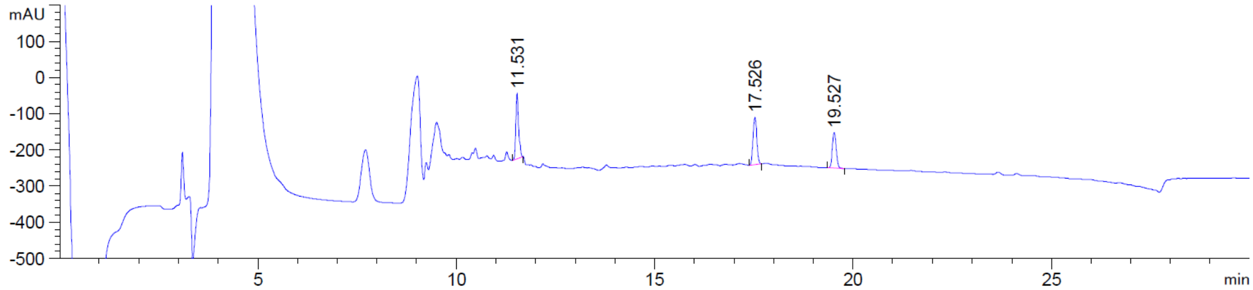

MS/MS Characterization:

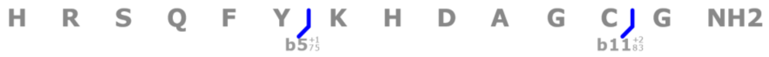

b5* (0.7), ***b11*** *(39.6)*.

14. RuAtAC procedure

a. Optimization

41a and 41a`

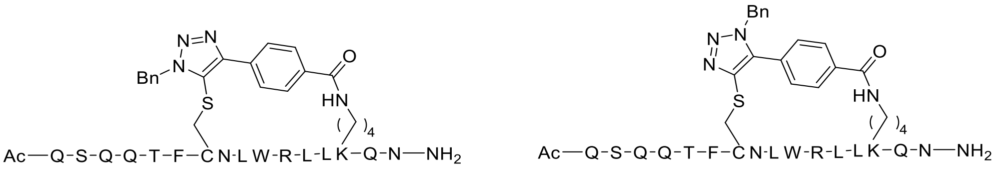

To *p*-Ac-QSQQTF(CNLWRLLK)QN-NH$_2$ (**29a**) (0.2 µmol, 1.0 equiv.) (retention time 15.6) Cp*Ru(cod)Cl (0.04 µmol, 20 mol%) and (azidomethyl)benzene (0.2 µmol, 1.0 equiv.) were added as solutions in DMF. Both solutions were prepared and added under nitrogen flow. The reaction vessel was sealed with parafilm and shaken for 24 hours under air. The conversion was followed using HPLC/MS retention times of the products were determined using LRMS obtained for each peak.

Absorbance ratio(%) = [(UV absorbance of product)/((combined UV absorbance of stapled peptide and product)] * 100. Due to the small reaction volumes it would be technically difficult to produce samples of precise concentration needed to obtain rel. abs. as in Table 2. And since formation of side products was not detected by HPLC analysis (see below), absorbance ratio was used to determine efficiency of the reaction.

Retention times of the products (**41a** and **41a`**): 16.2 and 16.5 min.
Retention time of the starting material **29a**: 15.5-15.6 min.
Retention time of the residual 2-iodobenzoic acid (**51**): 17.5-17.6 min.

HRMS of the mixture (ESI/QTOF) m/z: $[M]^+$ Calcd for $C_{106}H_{152}N_{31}O_{26}S^+$ 2307.1240; Found 2307.1330.

Table S10: Optimization of RuAtAC reaction of stapled peptides

Entry	Loading of Ru cat. (mol%)	Conc. (mM)	Equiv. of BnN_3	Time (h)	absorb. ratio (%)
1	5	2	1	24	<1
2	5	27	1	24	36
3	5	27	3	24	<1
4	20	27	1	3	96
				24	quant.
5	50	27	1	3	98
				24	quant.

Entry 1:

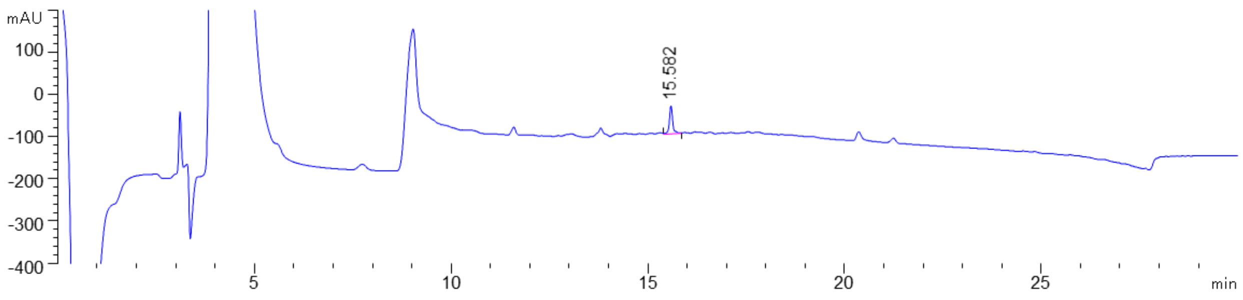

MS:

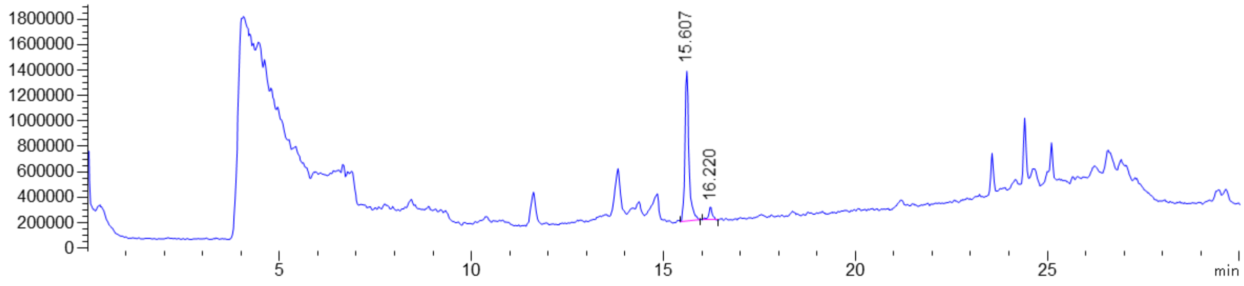

Entry 2:

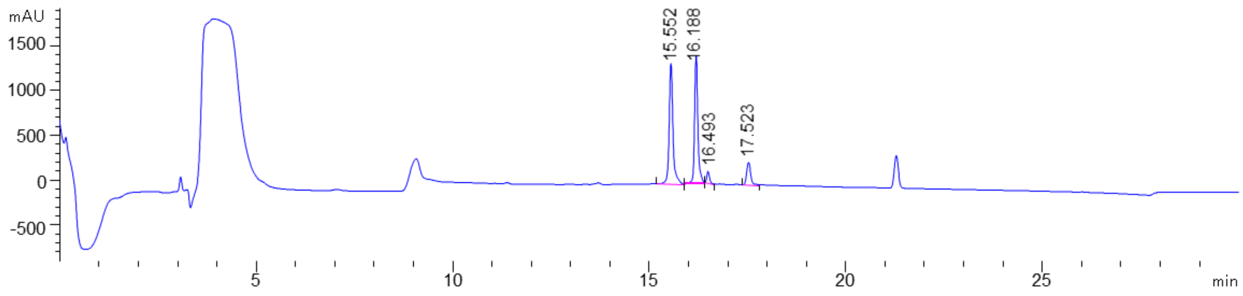

Entry 3:

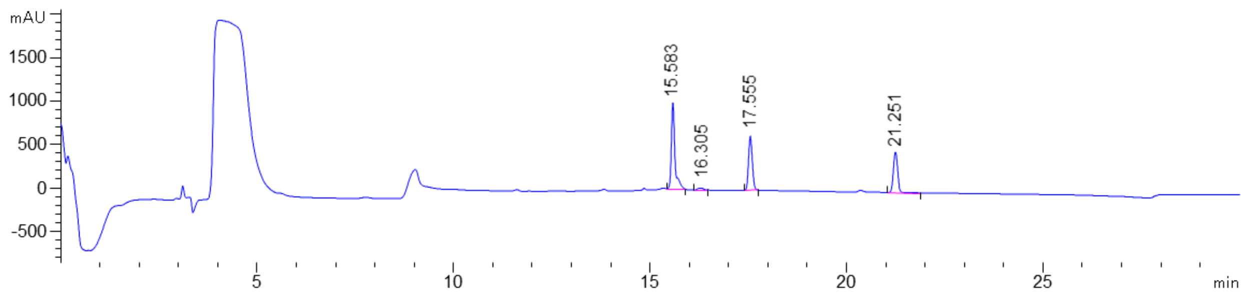

Entry 4:

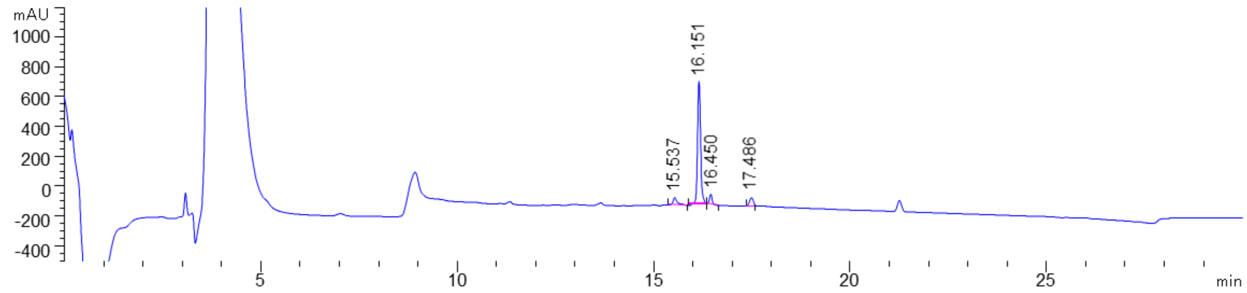

24h:

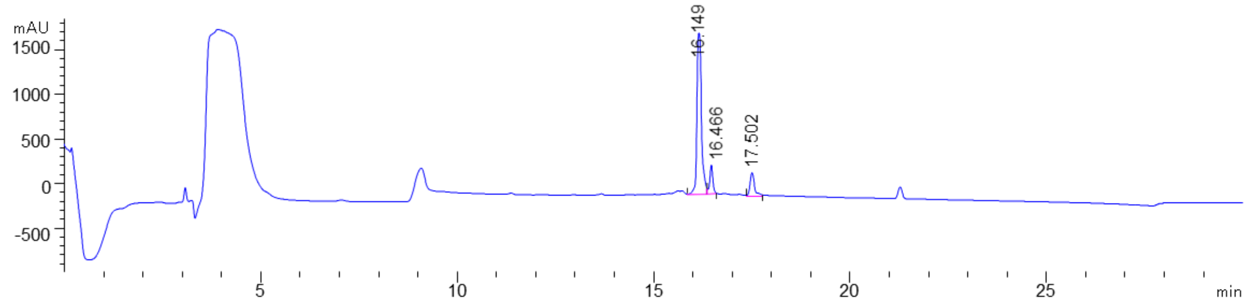

Entry 5:

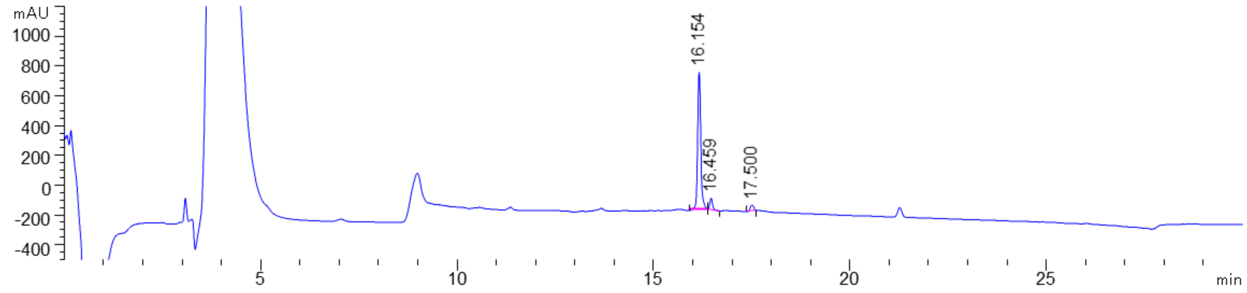

24h:

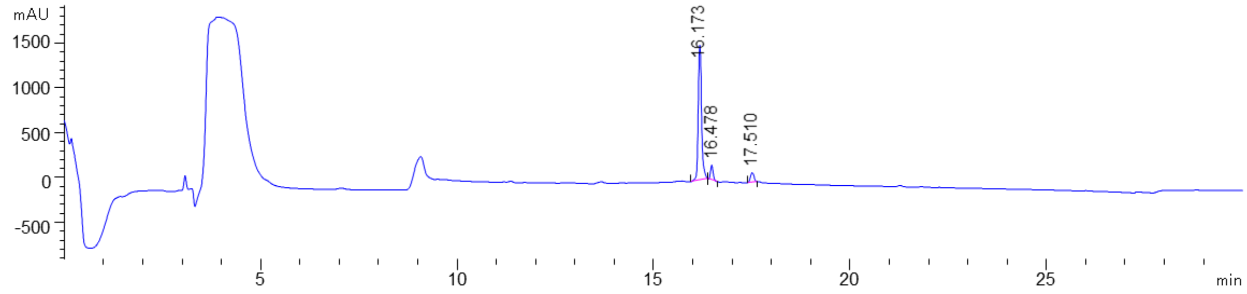

b. Azide and stapled peptide scope

General procedure for the RuAtAC reaction: to the isolated stapled peptide (0.2 µmol, 1.0 equiv.) 0.06 M solution of Cp*Ru(cod)Cl (0.04 µmol, 20 mol%) in DMF and 0.01 M solution of (azidomethyl)benzene (0.2 µmol, 1.0 equiv.) in DMF were added. Both solutions were prepared and added under nitrogen flow. The reaction vessel was sealed with parafilm and shaken for 24 h under air. The conversion was followed using HPLC/MS, retention times of the products were determined using LRMS obtained for each peak.

Absorbance ratio(%) = [(UV absorbance of product)/((combined UV absorbance of stapled peptide and product)] * 100. Due to the small reaction volumes it would be technically difficult to produce samples of precise concentration needed to obtain rel. abs. as in Table 2. And since formation of side products was not detected by HPLC analysis (see below), absorbance ratio was used to determine efficiency of the reaction.

Retention time of a residual 2-iodobenzoic acid (**51**): 17.5-17.6 min.

41a and 41a´ (Entry 1, Table4):

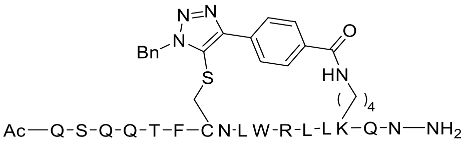

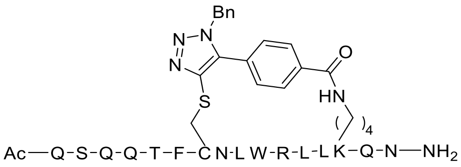

Following the general procedure for the RuAtAC reaction, *p*-Ac-QSQQTF(CNLWRLLK)QN-NH$_2$ (**29a**) together with (azidomethyl)benzene afforded the products (**41a** and **41a´**) in quant. absorb. ratio (retention times 16.2 and 16.5 min) and ratio 10:1.
HRMS of the mixture (ESI/QTOF) m/z: [M + H$_3$]$^{+3}$ Calcd for $C_{132}H_{185}N_{34}O_{33}S^{+3}$ 935.4516; Found 935.4515
HPLC-UV chromatogram at 210 nm of the crude reaction mixture after 24h:

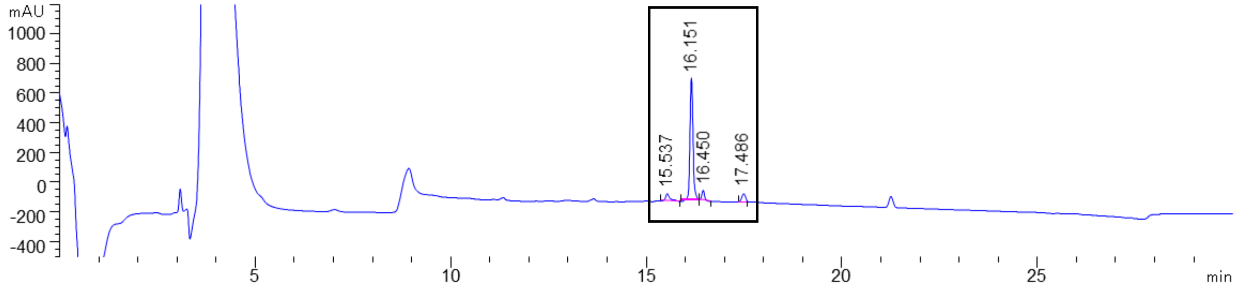

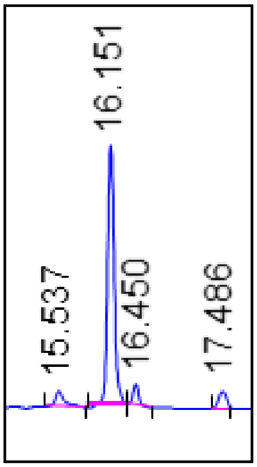

41b and 41b` (Entry 2, Table 4)

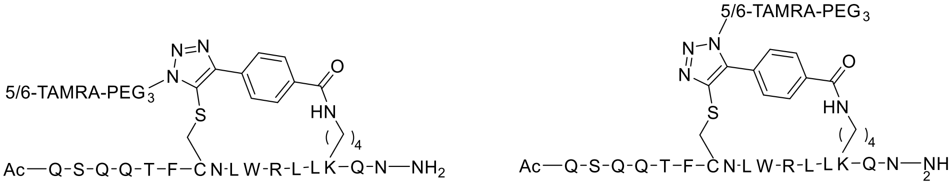

Following the general procedure for the RuAtAC reaction, *p*-Ac-QSQQTF(CNLWRLLK)QN-NH_2 (**29a**) together with 4/5-((2-(2-(2-(2-azidoethoxy)ethoxy)ethoxy)ethyl)carbamoyl)-2-(6-(dimethylamino)-3-(dimethyliminio)-3H-xanthen-9-yl)benzoate afforded the products (**41b** and **41b`**) in quant. absorb. ratio (retention times 15.3, 15.4 min).

HRMS of the mixture (ESI/QTOF) m/z: $[M + H_3]^{+3}$ Calcd for $C_{132}H_{185}N_{34}O_{33}S^{+3}$ 935.4516; Found 935.4515

HPLC-UV chromatogram at 210 nm of the crude reaction mixture after 24h:

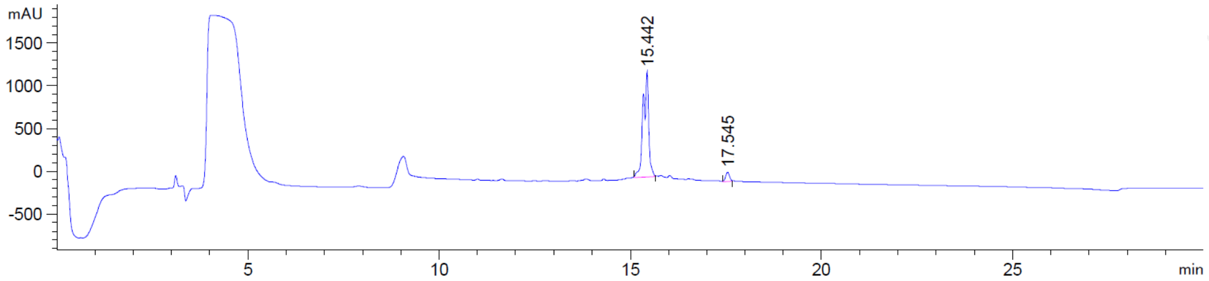

41c (Entry 3, Table 4)

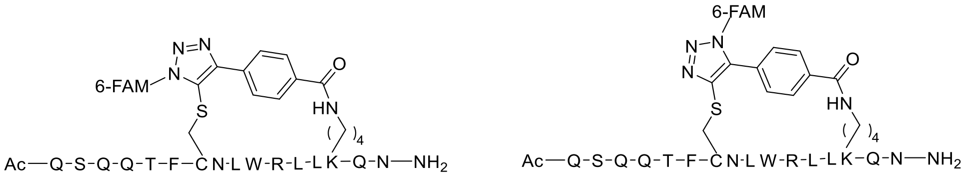

Following the general procedure for the RuAtAC reaction, *p*-Ac-QSQQTF(CNLWRLLK)QN-NH_2 (**29a**) together with 4-((3-azidopropyl)carbamoyl)-2-(3,6-dihydroxy-9H-xanthen-9-yl)benzoic acid afforded the products (**41c**) in quant. absorb. ratio (retention time 16.1 min).

HRMS of the mixture (ESI/QTOF) m/z: $[M + H_3]^{+3}$ Calcd for $C_{123}H_{165}N_{32}O_{32}S^{+3}$ 878.0657; Found 878.0647

HPLC-UV chromatogram at 210 nm of the crude reaction mixture after 24h:

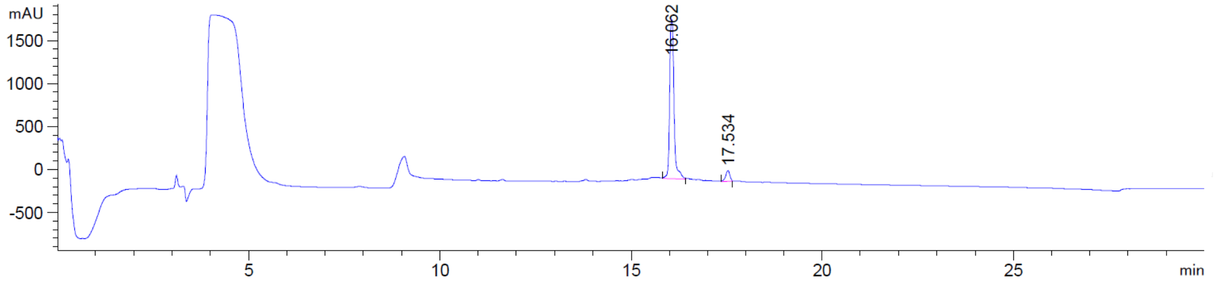

42 and 42` (Entry 4, Table 4)

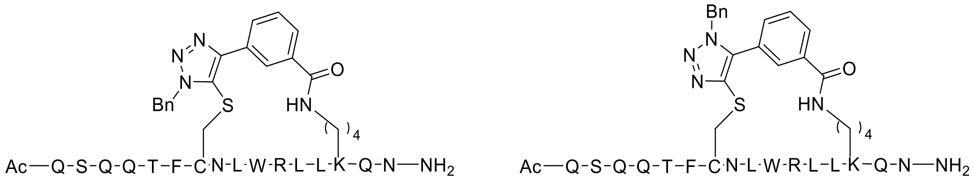

Following the general procedure for the RuAtAC reaction, *m*-Ac-QSQQTF(CNLWRLLK)QN-NH_2 (**29b**) (retention time 15.8) together with (azidomethyl)benzene afforded the products (**42** and **42`**) in 71% absorb. ratio (retention time 16.2 and 16.4 min) and ratio 36:1.

HRMS of the mixture (ESI/QTOF) m/z: $[M+H_2]^{+2}$ Calcd for $C_{106}H_{153}N_{31}O_{26}S^{+2}$ 1154.0656; Found 1154.0665.

HPLC-UV chromatogram at 210 nm of the crude reaction mixture at 24h:

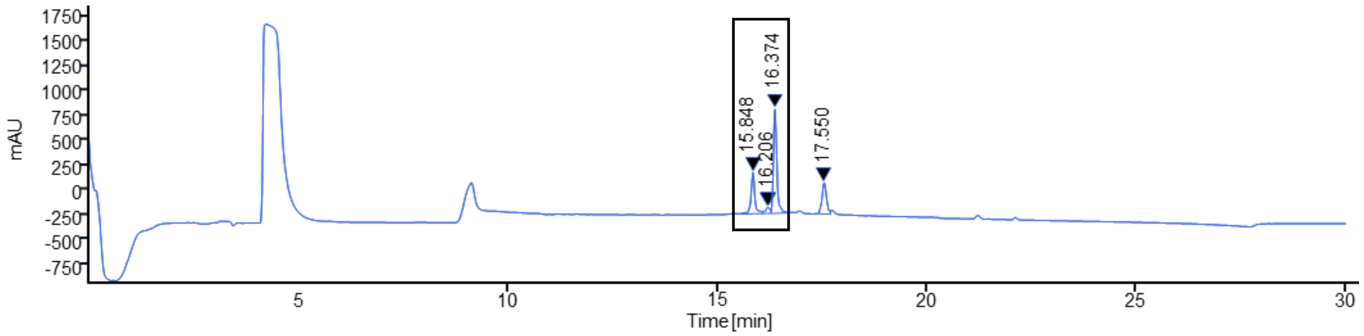

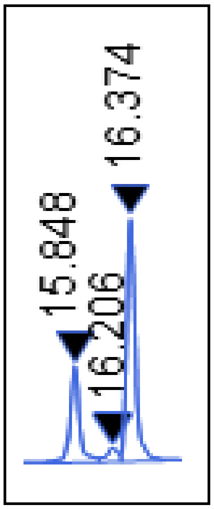

43 and 43` (Entry 5, Table 4)

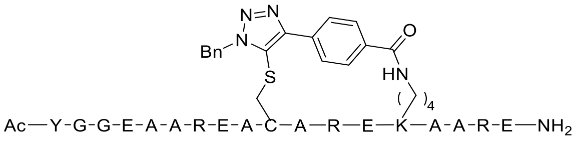

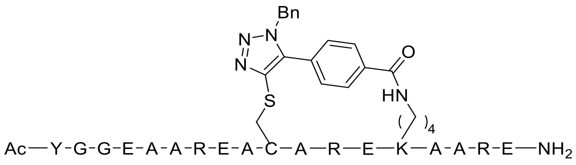

Following the general procedure for the RuAtAC reaction, *p*-Ac-YGGEAAREA(CAREK)AARE-NH_2 (**27a**) (retention time 11.6) together with (azidomethyl)benzene afforded the products (**43** and **43`**) in quant. absorb. ratio (retention times 12.5 and 12.8 min) and ratio 32:1.

HRMS of the mixture (nanochip-ESI/LTQ-Orbitrap) m/z: $[M+H_3]^{+3}$ Calcd for $C_{96}H_{143}N_{32}O_{29}S^{+3}$ 746.6801; Found 746.6818

HPLC-UV chromatogram at 210 nm of the crude reaction mixture after 24h:

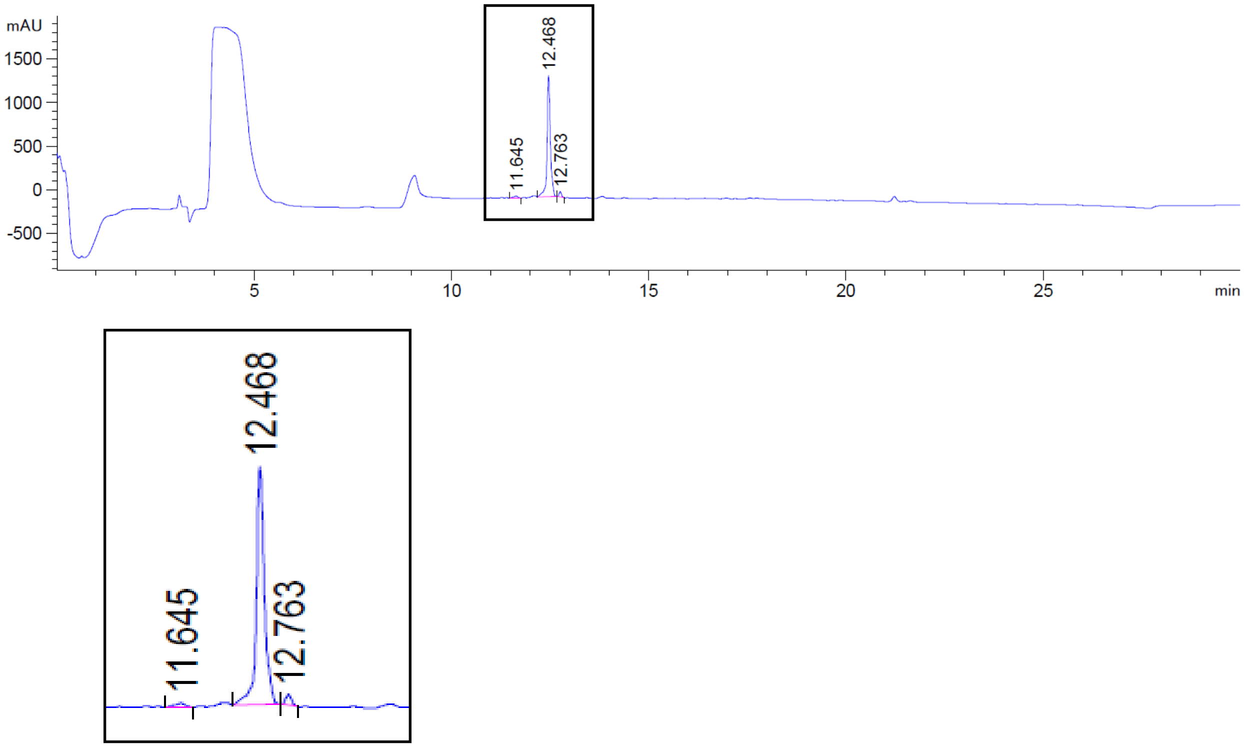

44 (Entry 6, Table 4)

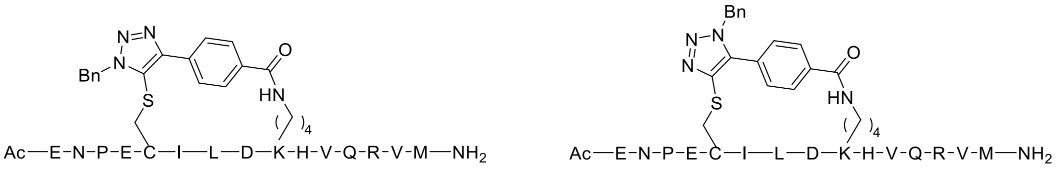

Following the general procedure for the RuAtAC reaction, *p*-Ac-ENPE(CILDK)HVQRVM-NH$_2$ (**40a**) (retention time 13.2) together with (azidomethyl)benzene afforded the product (**44**) in 59% absorb. ratio (retention time 13.9 min).

HRMS of the mixture (nanochip-ESI/LTQ-Orbitrap) m/z: $[M+H_2]^{+2}$ Calcd for $C_{94}H_{141}N_{27}O_{25}S_2^{+2}$ 1056.0011; Found 1056.0029.

HPLC-UV chromatogram at 210 nm of the crude reaction mixture after 24hours:

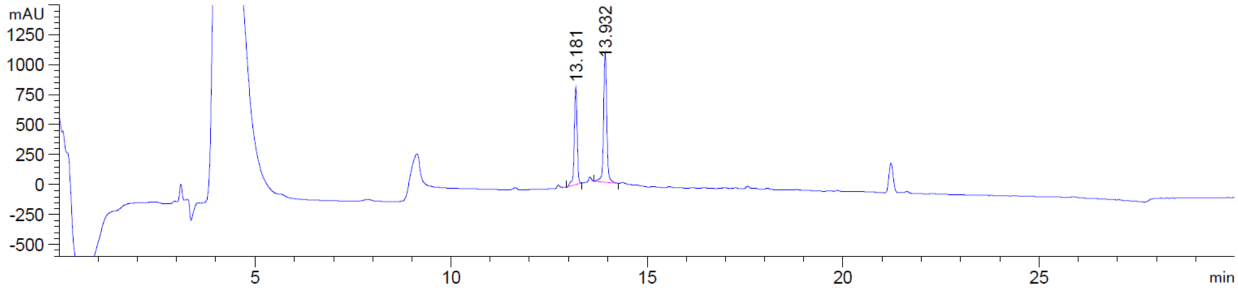

61

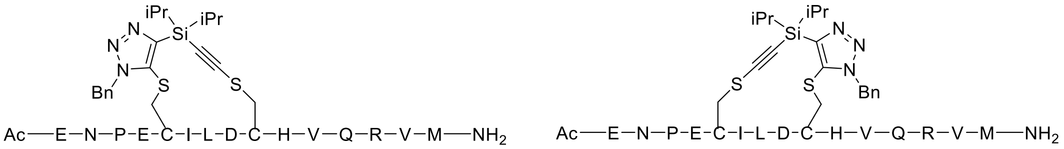

To the isolated Si(*i*Pr)$_2$-Ac-ENPE(CILDC)HVQRVM-NH$_2$ (**17a**) (0.2 µmol, 1.0 equiv.) 0.03 M solution of Cp*Ru(cod)Cl (0.1 µmol, 50 mol%) in DMF and 0.05 M solution of (azidomethyl)benzene (0.2 µmol, 1.0 equiv.) in DMF were added. Both solutions were prepared and added under nitrogen flow. The reaction vessel was sealed with parafilm and shaken for 24 h under air. The conversion was followed using HPLC. Only trace of the product (**61**) were detected by LRMS.

HPLC-UV chromatogram at 210 nm of the crude reaction mixture after 24 hours:

Retention of the starting material **17a**: 14.6 min.

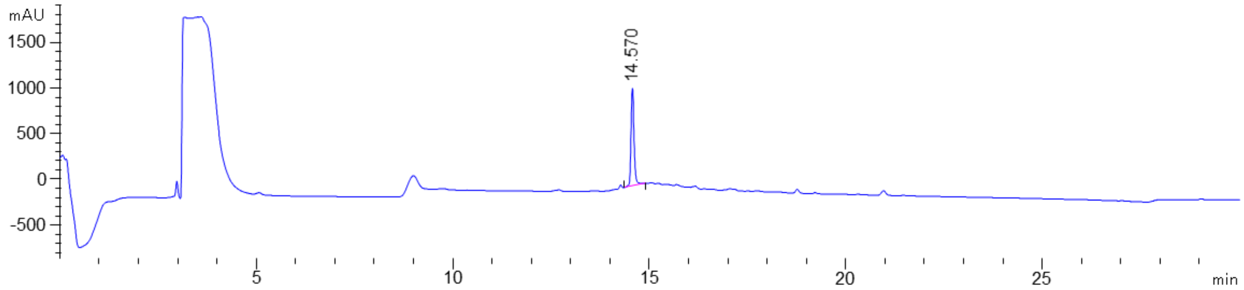

15. One-pot RuAtAC procedure

a. Optimization

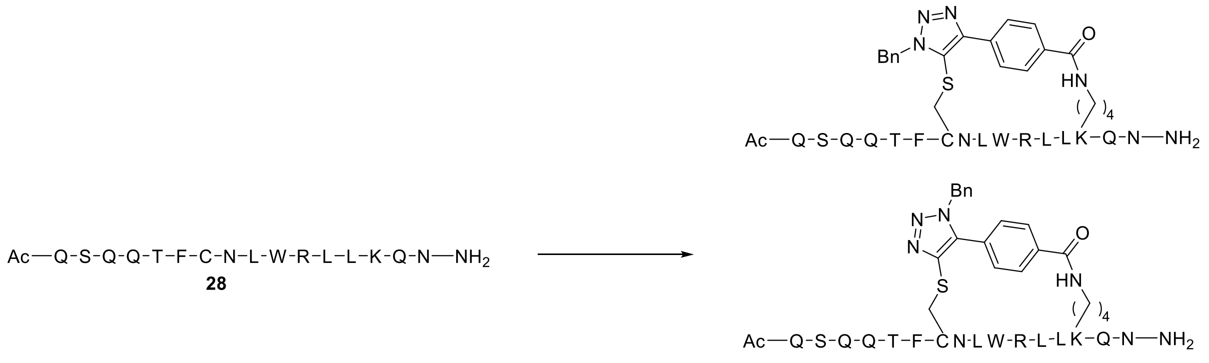

28

41a and **41a`**

Following the general procedure for the Cysteine Lysine reactions (Section 7), to Ac-QSQQTFCNLWRLLKQN-NH_2 (**28**) in DMF (84 or 94 µL) perfluorophenyl 4-((3-oxo-1λ^3-benzo[d][1,2]iodaoxol-1(3H)-yl)ethynyl)benzoate (**9a**) afforded *p*-Ac-QSQQTF(CNLWRLLK)QN-NH_2 (**29a**) in quant. HPLC yield. The crude reaction mixture of *p*-Ac-QSQQTF(CNLWRLLK)QN-NH_2 (**29a**) together with 0.03-0.04 M solution of Cp*Ru(cod)Cl (20 or 50 mol%) in DMF and 0.1 M solution of (azidomethyl)benzene in DMF afforded **41a** and **41a`** in 42% or 89% absorb. ratio accordingly.

HPLC-UV chromatogram at 210 nm of the crude reaction mixture after 24 hours using 20 mol% of Cp*Ru(cod)Cl:

Retention times of:

Linear peptide **28**	15.1 min
Product **41a**	15.7 min
2-iodobenzoic acid (**51**)	17.0 min
Diisopropylethylammonium perrfluorophenyloxide	19.2 min

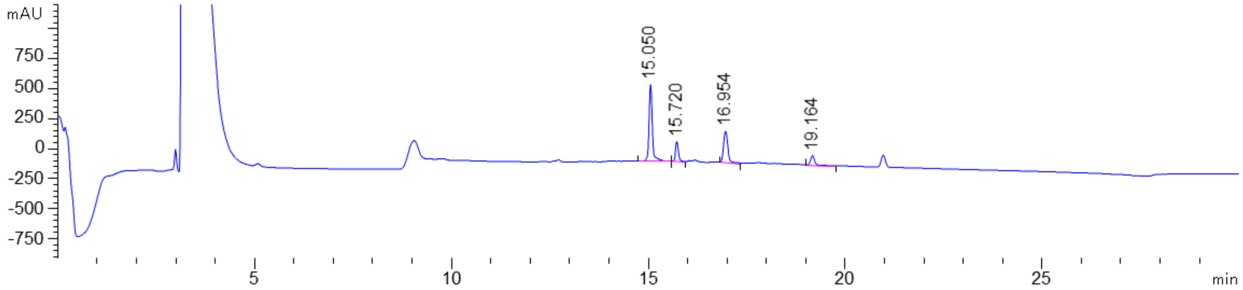

HPLC-UV chromatogram at 210 nm of the crude reaction mixture after 24 hours using 50mol% of Cp*Ru(cod)Cl:

Retention times of:

Starting material **28**	15.6 min
Products **41a** and **41a`**	16.2 and 16.5 min
2-iodobenzoic acid (**51**)	17.6 min
Diisopropylethylammonium perrfluorophenyloxide	19.6 min

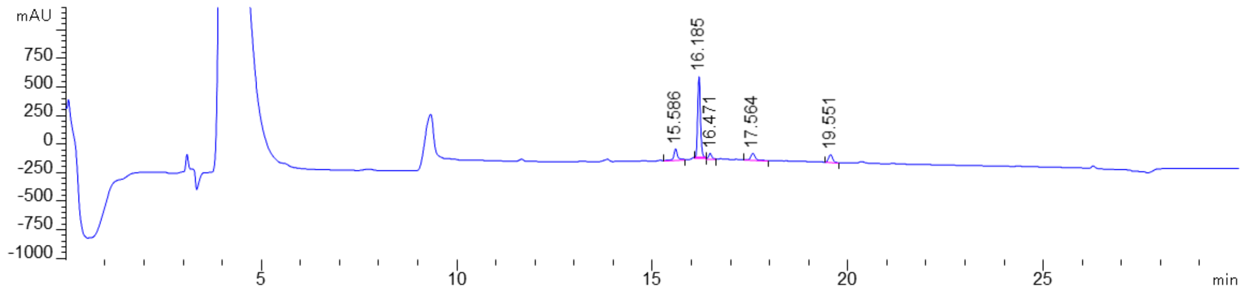

b. Isolation of the products using optimized one-pot procedure

41a and 41a`

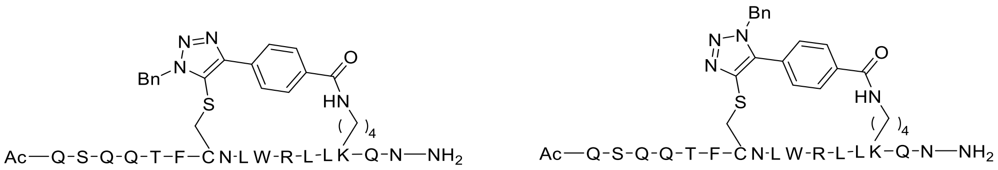

To 6.1 mM solution of Ac-QSQQTFCNLWRLLKQN-NH_2 (**28**) (6.7 mg, 2.9 µmol, 1.0 equiv.) in DMF, 0.2 M solution of N-ethyl-N-isopropylpropan-2-amine (37 µl, 7.4 µmol, 2.5 equiv.) in DMF and 0.1 M solution of **9a** (32 µl, 3.2 µmol, 1.1 equiv.) in DMF were added under nitrogen atmosphere. The solution was vortexed once more and the mixture was shaken and heated to 37 °C for 30 minutes under nitrogen atmosphere. To the crude mixture 0.1 M solution of Cp*Ru(cod)Cl (37 µl, 1.5 µmol, 50 mol%) in DMF and 0.04 M solution of (azidomethyl)benzene (30 µl, 3.0 µmol) in DMF were added under nitrogen atmosphere. All solutions were made under nitrogen flow. The reaction mixture was stirred for 3 hours. The crude mixture was purified using preparative RP-HPLC to afford the products **41a** in 52% (3.7 mg, 1.5 µmol, retention time: 16.2 min) and **41a`** 4% (0.3 mg, 0.1 µmol, retention time: 16.5 min) yield.

41a:

HRMS (nanochip-ESI/LTQ-Orbitrap) m/z: $[M + HNa]^{+2}$ Calcd for $C_{106}H_{152}N_{31}NaO_{26}S^{+2}$ 1165.0566; Found 1165.0617.

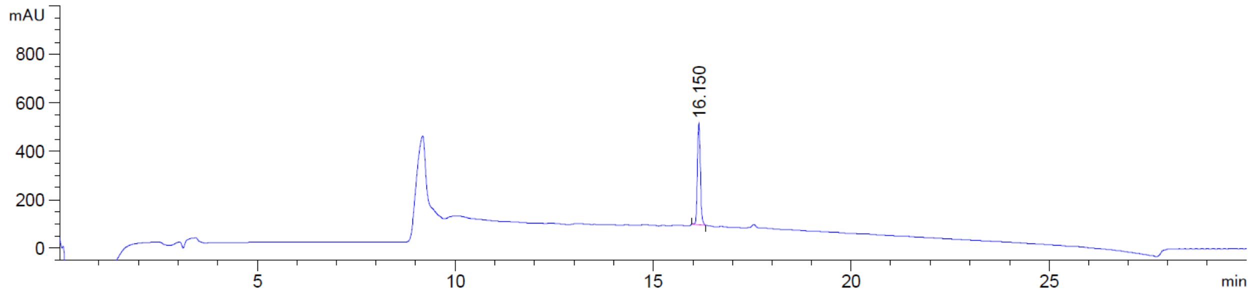

41a`:

HRMS (nanochip-ESI/LTQ-Orbitrap) m/z: [M + HNa]$^{+2}$ Calcd for $C_{106}H_{152}N_{31}NaO_{26}S^{+2}$ 1165.0566; Found 1165.0623.

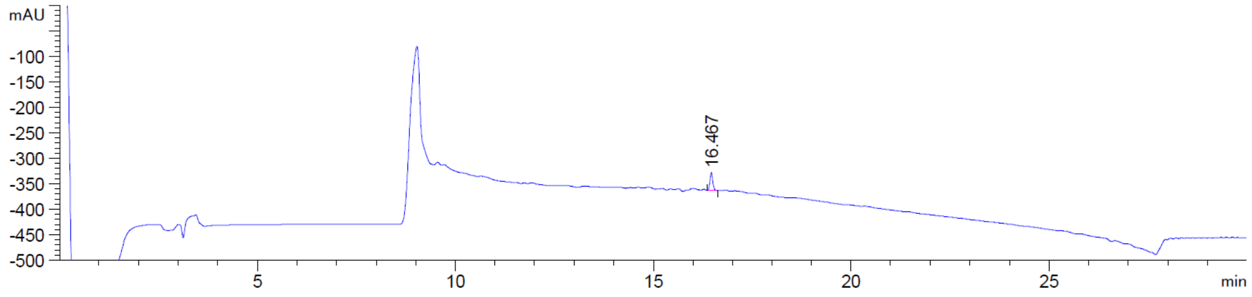

16. Circular Dichroism Measurements

General Procedure for the measurement of CD spectra: The lyophilized solid linear and staple peptides were dissolved in water (bubbled with nitrogen for 30 minutes) to afford a 1 mM solution. The 1 mM stock solution was diluted with water and TFE to afford 0.1 mM solutions with 0, 5, 10, 20 and/or 40% TFE. The ellipticity was measured from 260 nM to 180 nM. The data described is the average of at least 2 values. The mean peptide ellipticity [θ] was calculated using equation (1) and plotted against wavelength.

$$[\theta] = \frac{\theta}{10 \times C \times Np \times l} \qquad (1)$$

θ: ellipticity [mdeg], C: peptide molar concentration [M], Np: number of peptide units, l: cell path length [cm].[10]

The linear and staple peptides were compared at the same % of TFE. When possible, the comparison was made at 40% TFE. When HT went over 700 V the data was not considered accurate. For **17a** only measurements at 5% TFE provided reliable data over the whole range of wavelengths. For **27a**, no change was observed between 5% and 10 % TFA, thus for comparability **26** and **27b** are also reported at 5% TFA.

Table S11: Values of [θ] at 208 and 222 nm at 40% TFE:

	[θ], 10^{-3} deg cm^2 dmol^{-1}	
peptide	at 208 nm	at222 nm
**16**	-17.6973	-15.0094
**16**[a]	-7.72634	-5.10823
**17a**[a]	-4.41878	-3.67977
**17c**	-13.5956	-11.6452
**18**	-19.7211	-17.4597
**19a**	-9.05424	-9.67353
**19c**	-16.7328	-14.5365
**20**	-19.5064	-17.0082
**21a**	-10.1194	-10.8116
**21c**	-20.6443	-18.1432
**24**	-18.8331	-15.4929
**25a**	-9.0980	-6.45338
**25b**	-12.5676	-10.2989
**26**[a]	-12.4933	-10.9386
**27a**[a]	-4.68167	-2.83951
**27b**[a]	-11.6794	-10.5297
**28**	-14.115	-12.2692
**29a**	-19.7351	-18.6972
**29b**	-21.6834	-22.2641
**41a**	-15.1404	-18.0033

[a] 5% TFE

[10] Shepherd, N. E.; Hoang, H. N.; Abbenante, G.; Fairlie, D. P. *J. Am. Chem. Soc.* **2005**, *127*, 2974-2983.

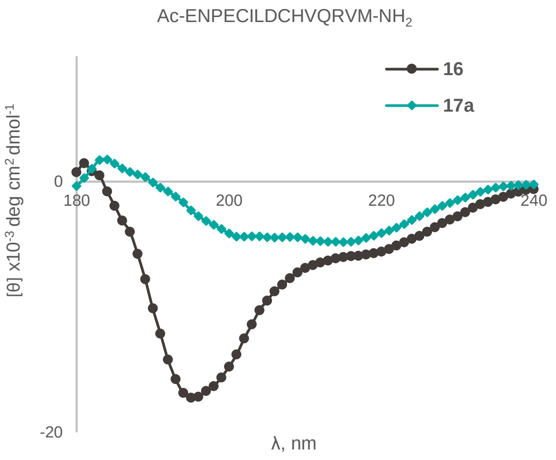

Figure S4: Circular Dichroism curve of the linear (**16**) and stapled peptide (**17a**) with a sequence of Ac-ENPECILDCHVQRVM-NH_2 at 0.1 mM in 5% TFE/Water. Interval for 180 to 240 nm.

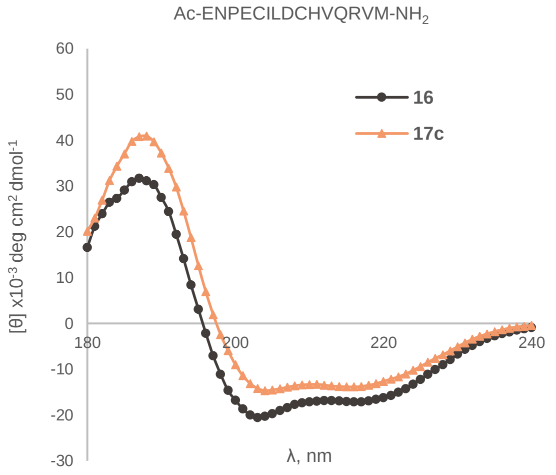

Figure S5: Circular Dichroism curve of the linear (**16**) and stapled peptide (**17c**) with a sequence of Ac-ENPECILDCHVQRVM-NH_2 at 0.1 mM in 40% TFE/Water. Interval for 180 to 240 nm.

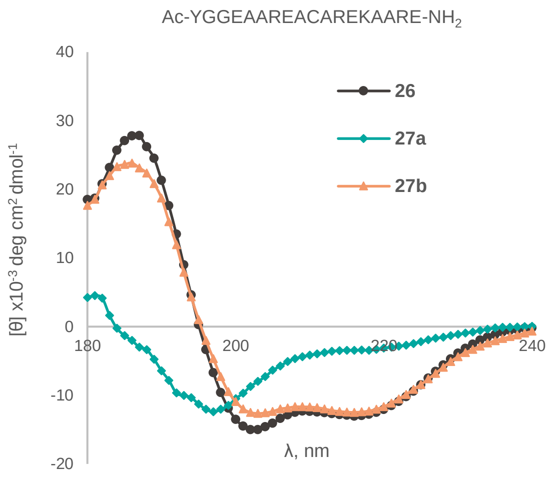

Figure S6: Circular Dichroism curve of the linear (**26**) and stapled peptides (**27a** and **27b**) with a sequence of Ac-YGGEAAREACAREKAARE-NH_2 at 0.1 mM in 5% TFE/Water. Interval for 180 to 240 nm.

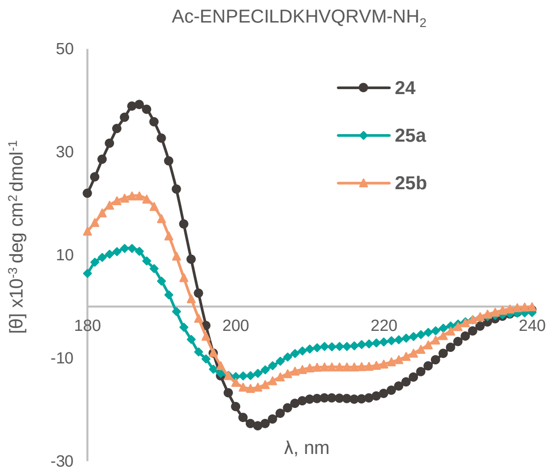

Figure S7: Circular Dichroism curve of the linear (**24**) and stapled peptides (**25a**, **25b**) with a sequence of Ac-ENPECILDKHVQRVM-NH_2 at 0.1 mM in 40% TFE/Water. Interval for 180 to 240 nm.

17. Synthesis of Fluoresceine Labelled Stapled Peptides

29a`

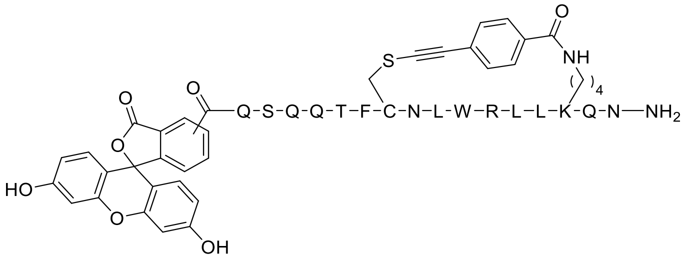

Following the general procedure for isolation of Cysteine-Lysine stapled peptides 5(6)-FAM - QSQQTFCNLWRLLKQN-NH2 (**28`**) (4.3 mg, 1.7 µmol) together with perfluorophenyl 4-((3-oxo-$1\lambda^3$-benzo[d][1,2]iodaoxol-1(3H)-yl)ethynyl)benzoate (**9a**) afforded the product (**29a`**) (3.2 mg, 1.3 µmol, 76%) (7129 mAu at 210 nm, 110% relative absorption) as a yellow amorphous solid (retention time 16.1 min).

HRMS (ESI/QTOF) m/z: [M + H]+2 Calcd for $C_{118}H_{154}N_{28}O_{31}S^{+2}$ 1245.5522; Found 1245.5557.

HPLC-UV chromatogram at 210 nm of the reaction mixture after 30 minutes:

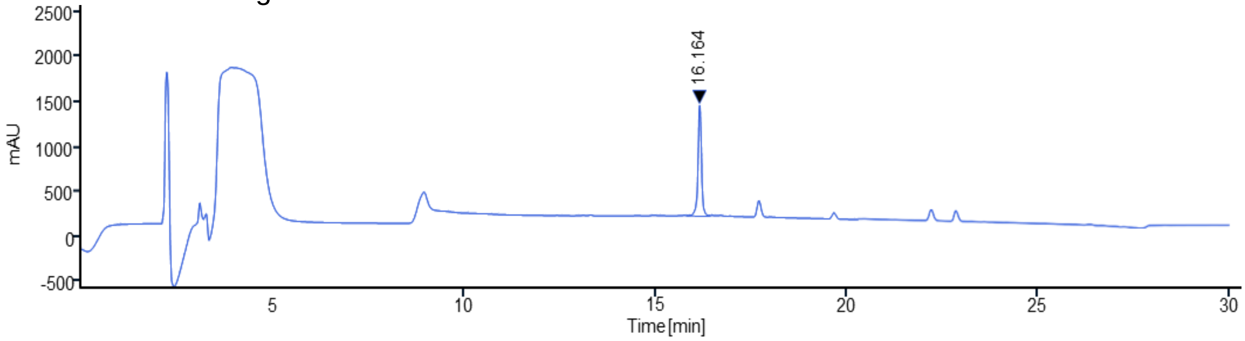

HPLC-UV chromatogram at 210 nm of the isolated product:

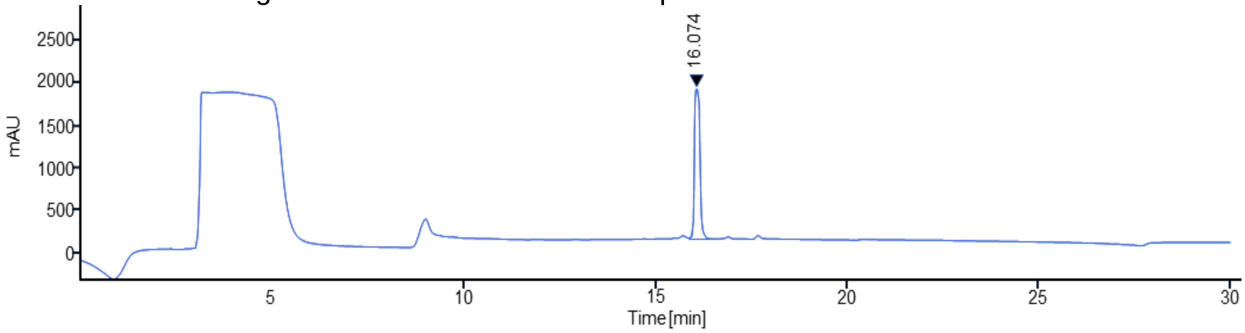

18. Binding assays

a. Competition fluorescence polarization assay

The binding of stapled peptides was measured by adding 2-fold dilutions of stapled peptide to pre-mixed fluorescent reporter peptide (5(6)-FAM-GSGSSQETFSDLWKLLPEN-NH_2) and human MDM2 (hMDM2) fused to GST (hMDM2-GST) in phosphate buffer (100 mM Na_2HPO_4, 18.5 mM NaH_2PO_4, 137 mM NaCl, 2.7 mM KCl, pH 7.4) containing 100 μM TCEP and 0.01% Tween-20. The reagents were added to wells of a 384-microwell plate (ThermoFischer NUNC™ 384 shallow well std height plates non-sterile, black) to reach a total assay volume of 15 μl. The final concentrations of stapled peptide ranged from 50 μM to 49 nM, and the final concentrations of reporter peptide and hMDM2-GST were 50 nM and 1 μM, respectively. After 30 min incubation at room temperature, the plate was read using a plate reader (Infinite 200 PRO Tecan, E_{ex}= 485 nm, E_{em} = 535 nm). Sigmoidal curves were fitted to the data using Graphpad Prism 5 software and the following dose-response equation (2):

$$y = \text{Bottom} + \frac{(\text{Top} - \text{Bottom})}{1 + 10^{(\log IC_{50} - x)p}} \qquad (2)$$

y: anisotropy, Top and Bottom: plateaus in the units of the Y axis, x: peptide concentration, p: Hill slope and IC_{50}: functional strength of the inhibitor.

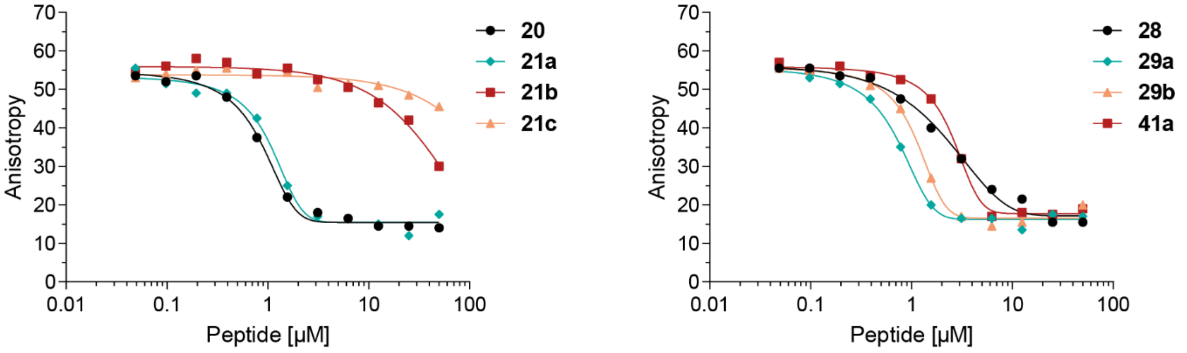

Figure S8: Binding of peptides to hMDM2 measured in a fluorescence polarization competition assays. Average values of two independent measurements are shown.

b. Direct fluorescence polarization binding assay

The binding of fluorescent peptide was measured by adding 2-fold dilutions of hMDM2-GST to a fixed concentration of fluorescein-labeled peptide in phosphate buffer (10 mM Na_2HPO_4, 1.85 mM NaH_2PO_4, 137 mM NaCl, 2.7 mM KCl, pH 7.4) containing 0.01% Tween-20. The reagents were added to wells of a 384-microwell plate (Greiner bio-one, Microplate, 384 well, PS, F-bottom, fluotrac, med binding, black) to reach a total assay volume of 50 μl. The final concentrations of fluorescein-labeled peptide was 2 nM and the one of hMDM2-GST ranged from around 10 μM to 2 nM. After 30 min incubation at room temperature, the fluorescence anisotropy was measured as described above. Sigmoidal curves were fitted to the data using Graphpad Prism 5 software and equation (3):

$$y = a + (b - a)\frac{(K_d + x + P) - \sqrt{(K_d + x + P)^2 - 4xP}}{2P} \qquad (3)$$

y: anisotropy, a: anisotropy of probe alone, b: anisotropy of probe signal in the presence of saturating concentrations of MDM2, x and P: the protein and probe concentrations respectively.

For data representation, the data was normalized wherein the anisotropy of fluorescent probe alone was set to 0% faction bound, and anisotropy of all fluorescent probe bound to 100% faction bound.

19. NMR spectra of the synthesized compounds.

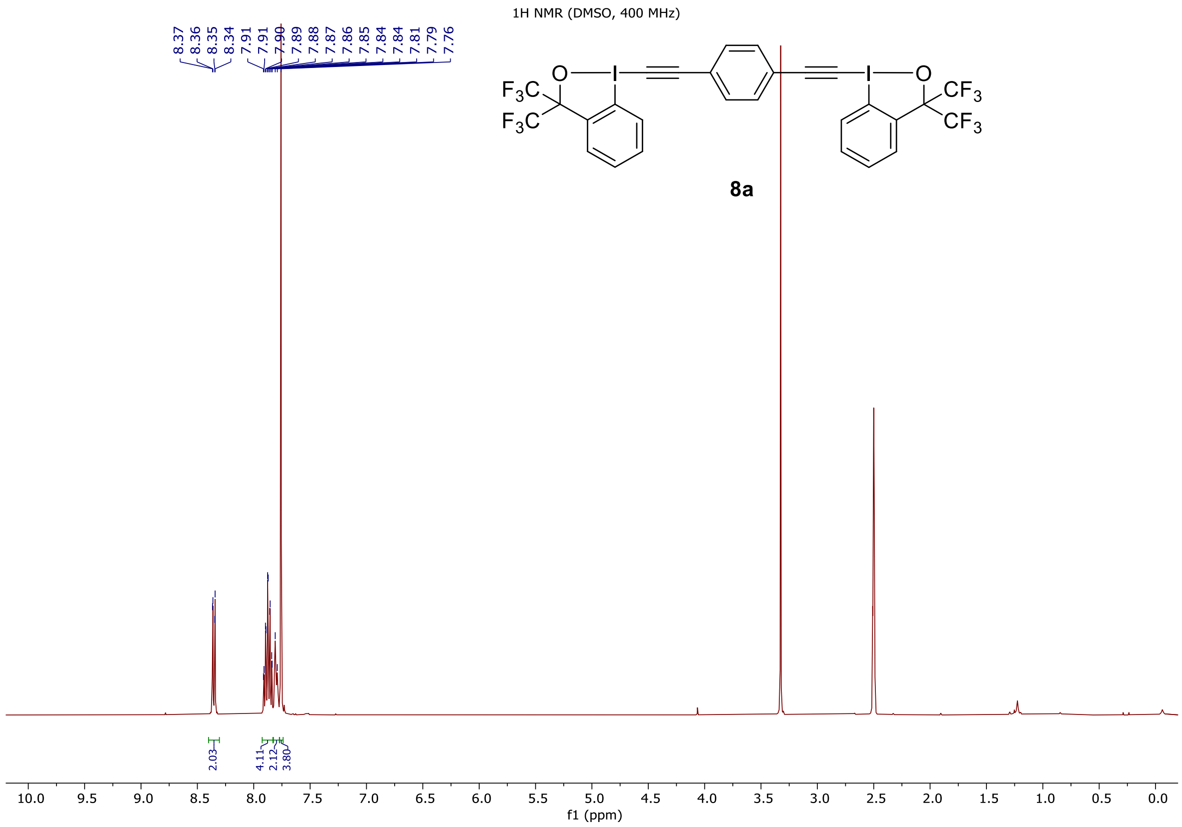

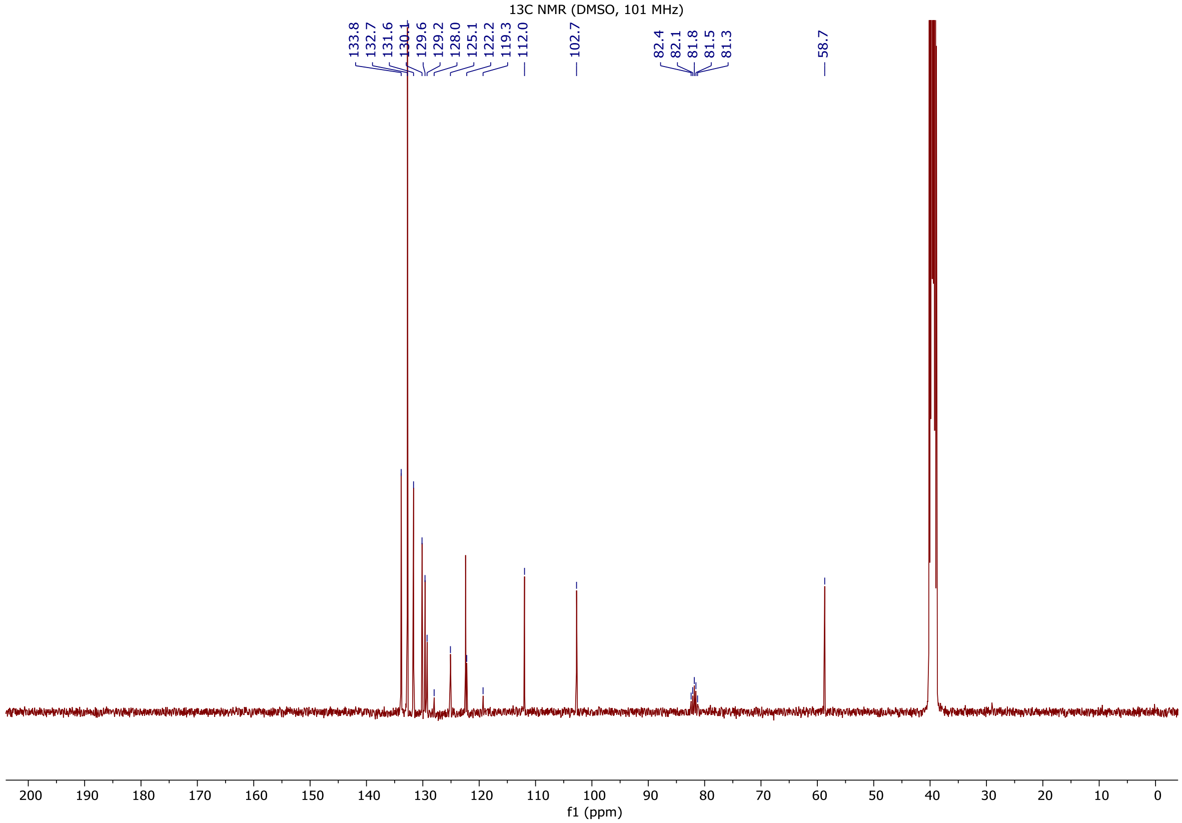

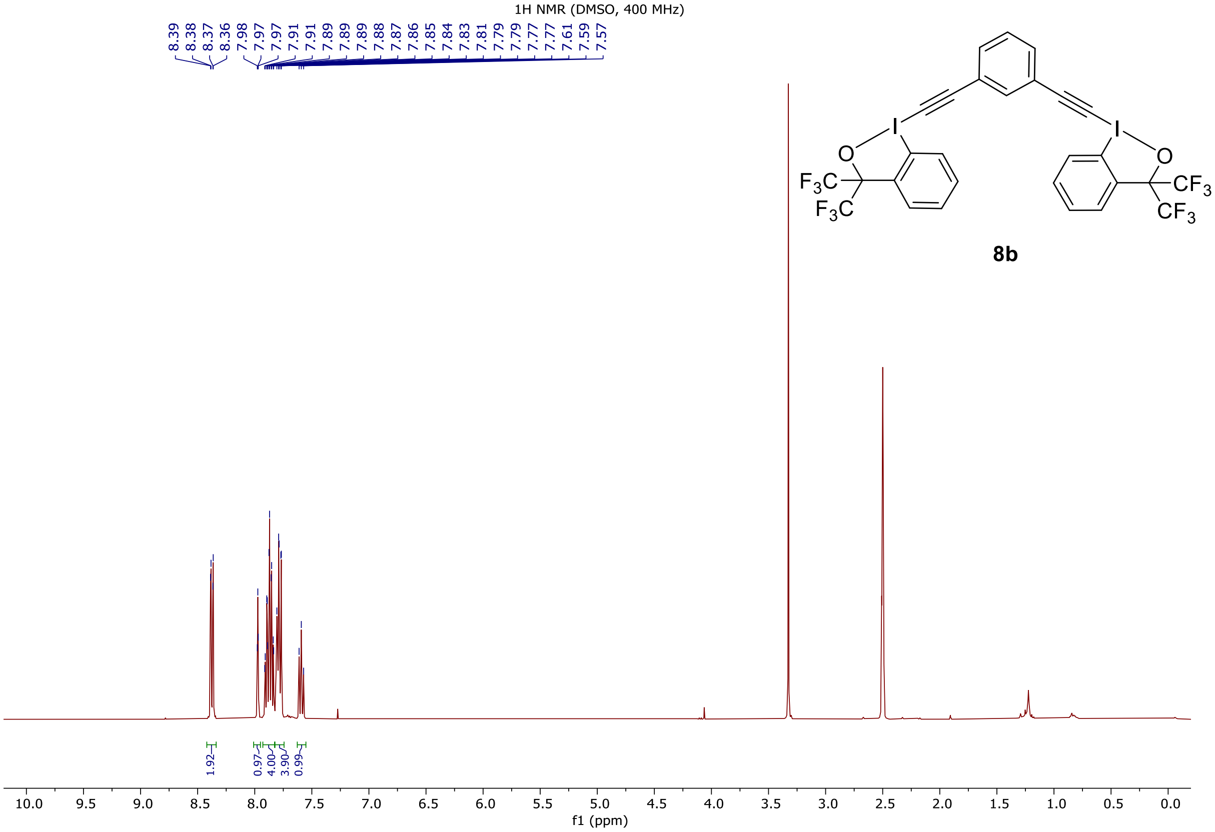
1H NMR (DMSO, 400 MHz)
8.39 8.38 8.37 8.36 7.98 7.97 7.97 7.91 7.91 7.89 7.89 7.89 7.88 7.87 7.86 7.85 7.84 7.83 7.81 7.79 7.79 7.77 7.77 7.77 7.61 7.59 7.57
1.92 0.97 4.00 3.90 0.99
10.0 9.5 9.0 8.5 8.0 7.5 7.0 6.5 6.0 5.5 5.0 4.5 4.0 3.5 3.0 2.5 2.0 1.5 1.0 0.5 0.0
f1 (ppm)
I O F_3C F_3C I O CF_3 CF_3
8b

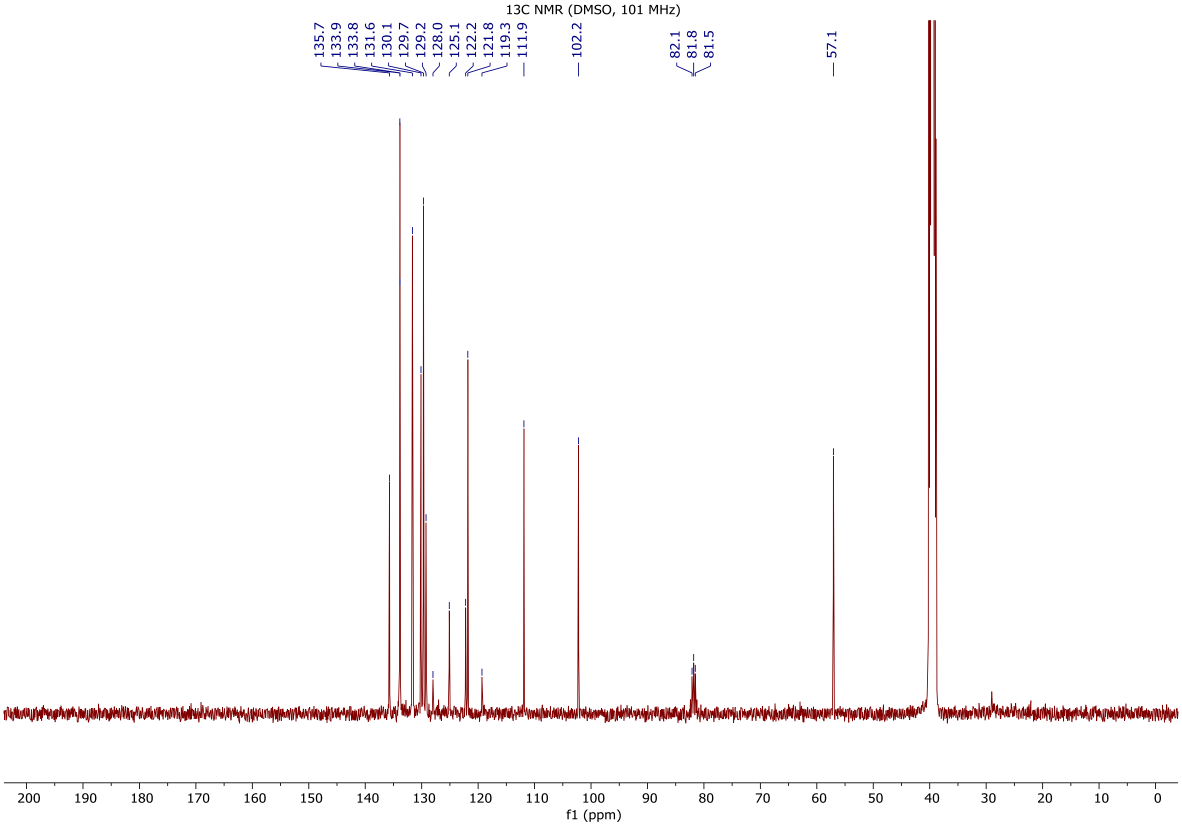
13C NMR (DMSO, 101 MHz)
135.7 133.9 133.8 131.6 130.1 129.7 129.2 128.0 125.1 122.2 121.8 119.3 111.9 102.2 82.1 81.8 81.5 57.1
200 190 180 170 160 150 140 130 120 110 100 90 80 70 60 50 40 30 20 10 0
f1 (ppm)

1H NMR (CDCl3, 400 MHz)

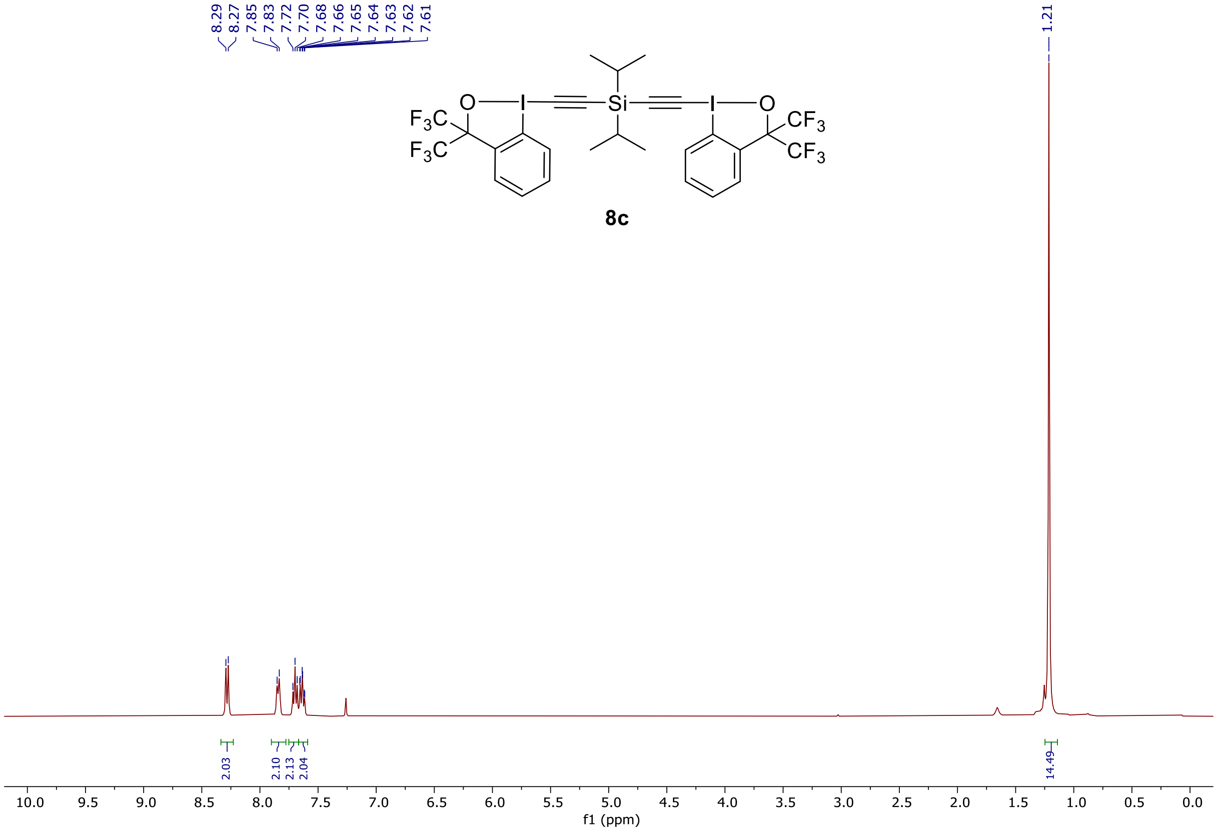

13C NMR (CDCl3, 101 MHz)

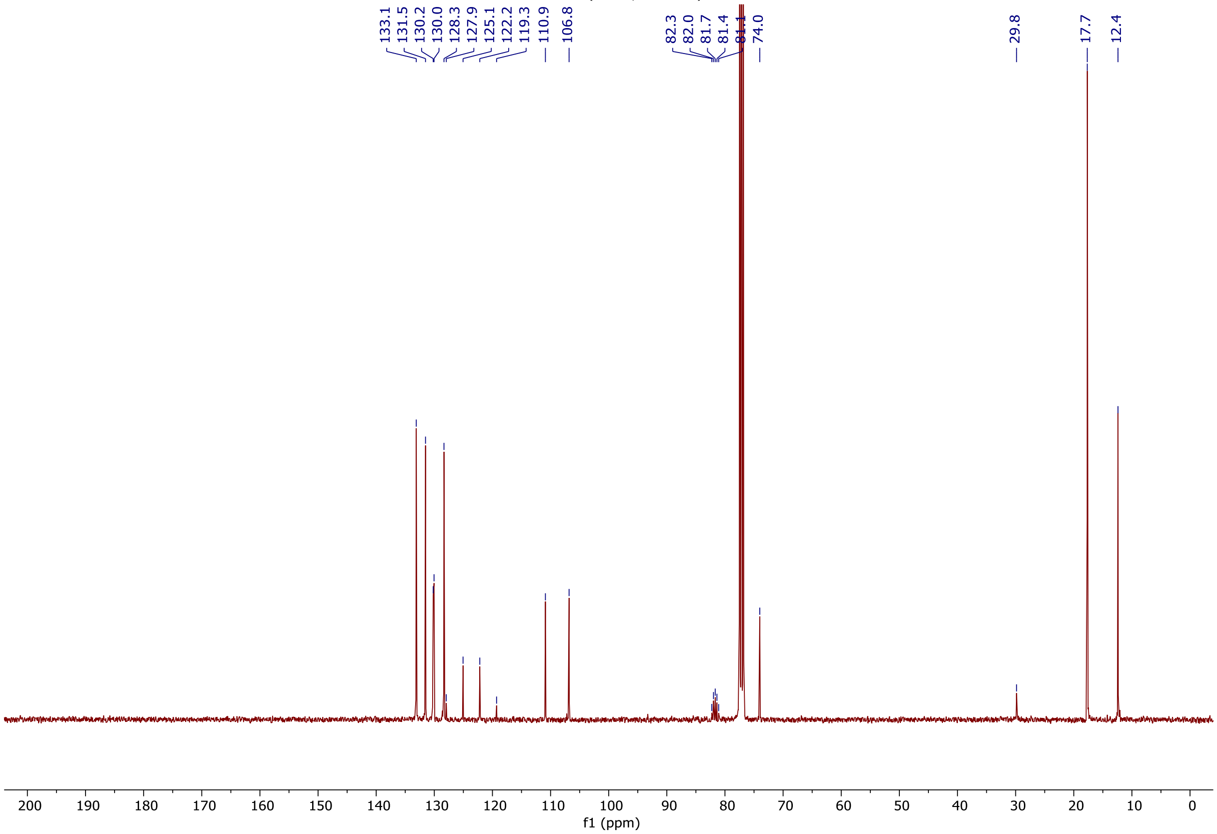

1H NMR (CDCl3, 400 MHz)

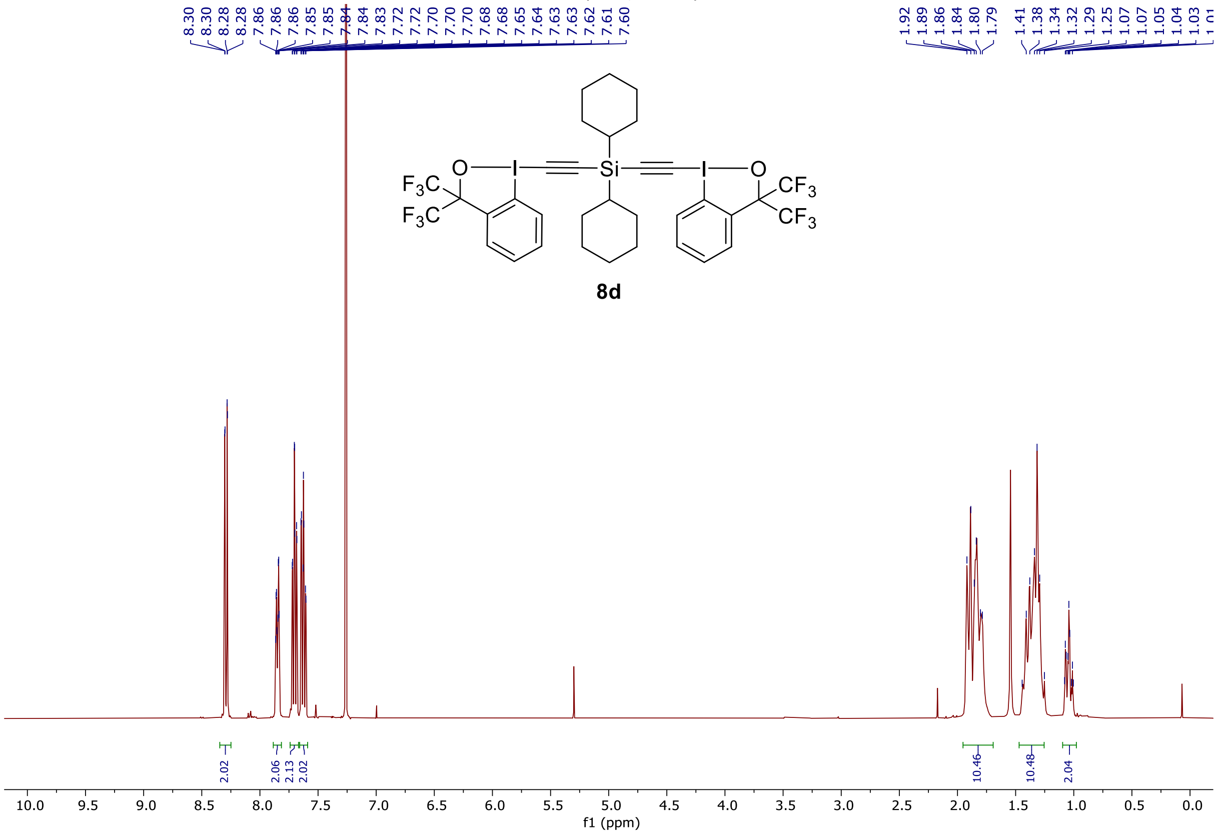

13C NMR (CDCl3, 101 MHz)

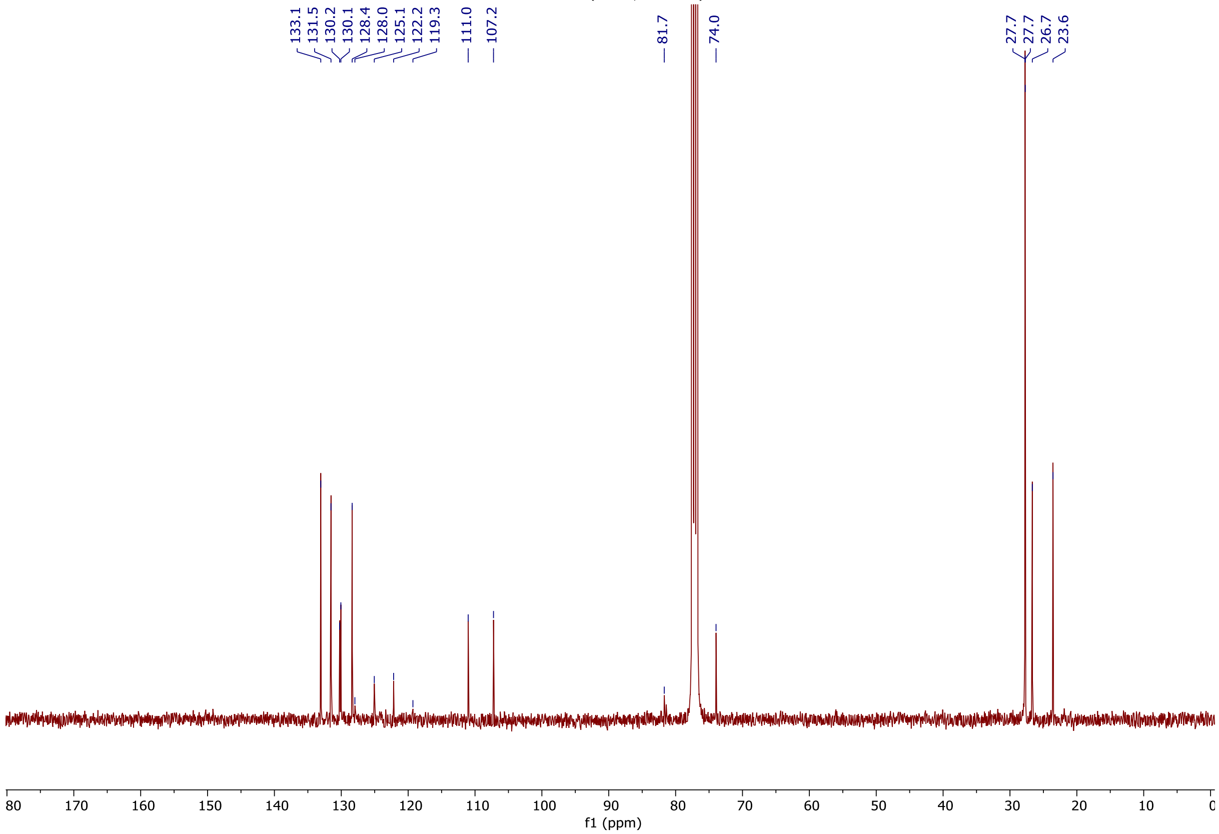

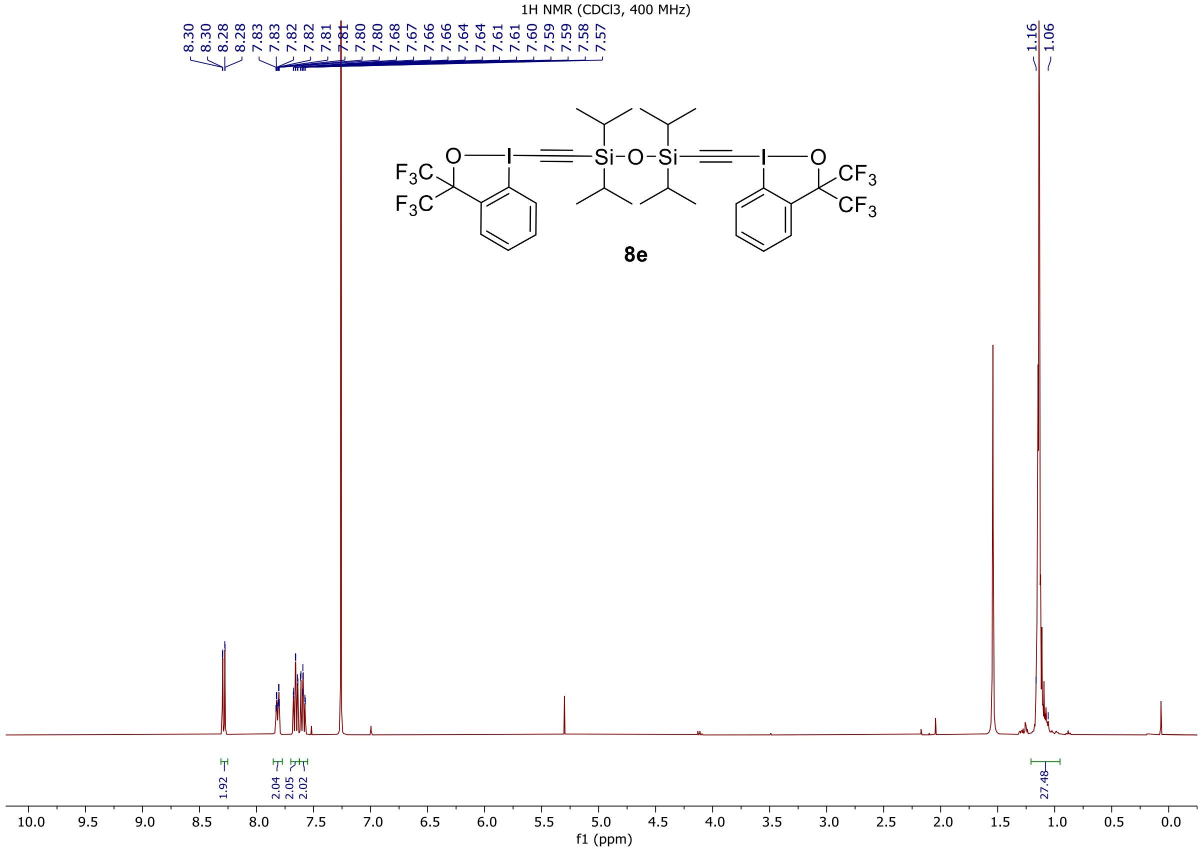
1H NMR (CDCl3, 400 MHz)
8.30 8.30 8.28 8.28 7.83 7.83 7.82 7.82 7.81 7.81 7.80 7.80 7.68 7.67 7.66 7.66 7.64 7.64 7.61 7.61 7.60 7.59 7.59 7.58 7.57
1.16 1.06
O I Si O Si I O
F3C F3C CF3 CF3
8e
1.92 2.04 2.05 2.02 27.48
10.0 9.5 9.0 8.5 8.0 7.5 7.0 6.5 6.0 5.5 5.0 4.5 4.0 3.5 3.0 2.5 2.0 1.5 1.0 0.5 0.0
f1 (ppm)

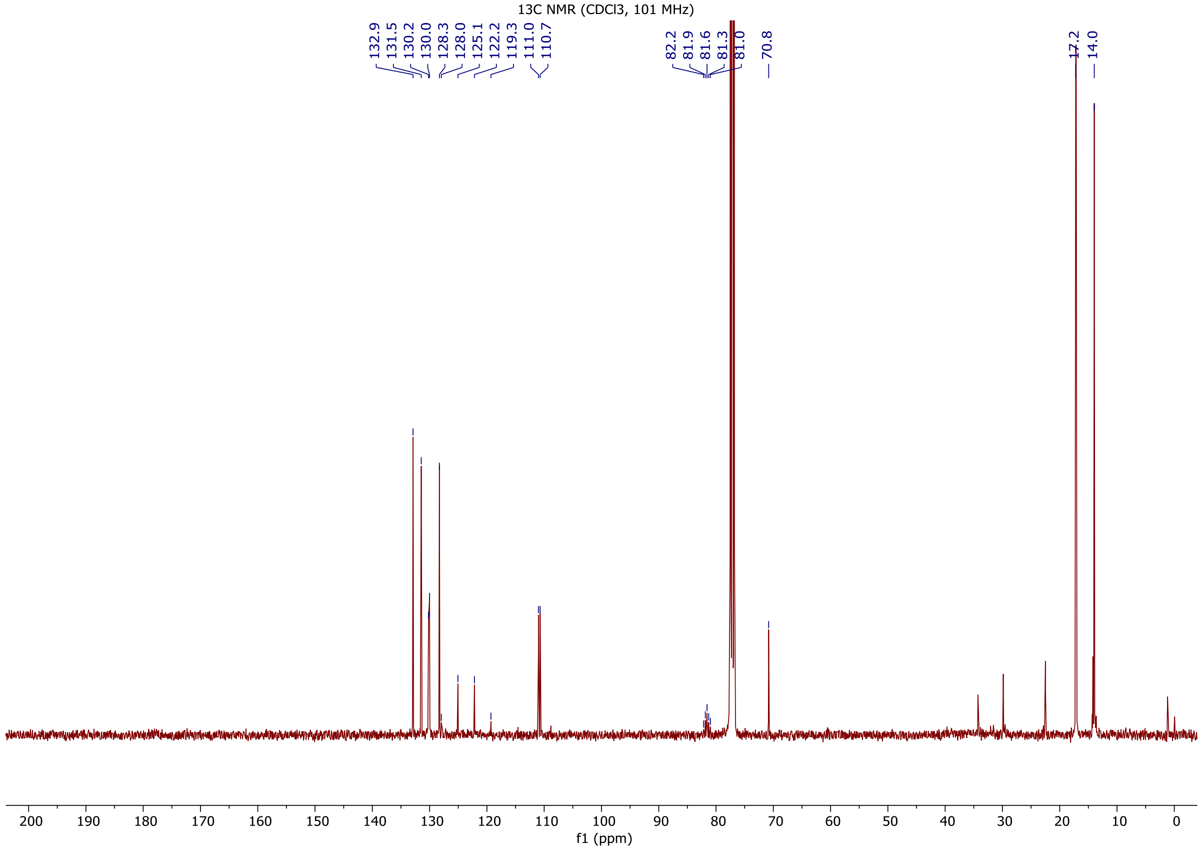
13C NMR (CDCl3, 101 MHz)
132.9 131.5 130.2 130.0 128.3 128.0 125.1 122.2 119.3 111.0 110.7
82.2 81.9 81.6 81.3 81.0 70.8
17.2 14.0
200 190 180 170 160 150 140 130 120 110 100 90 80 70 60 50 40 30 20 10 0
f1 (ppm)

1H NMR (CDCl3, 400 MHz)

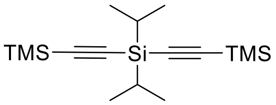

11c

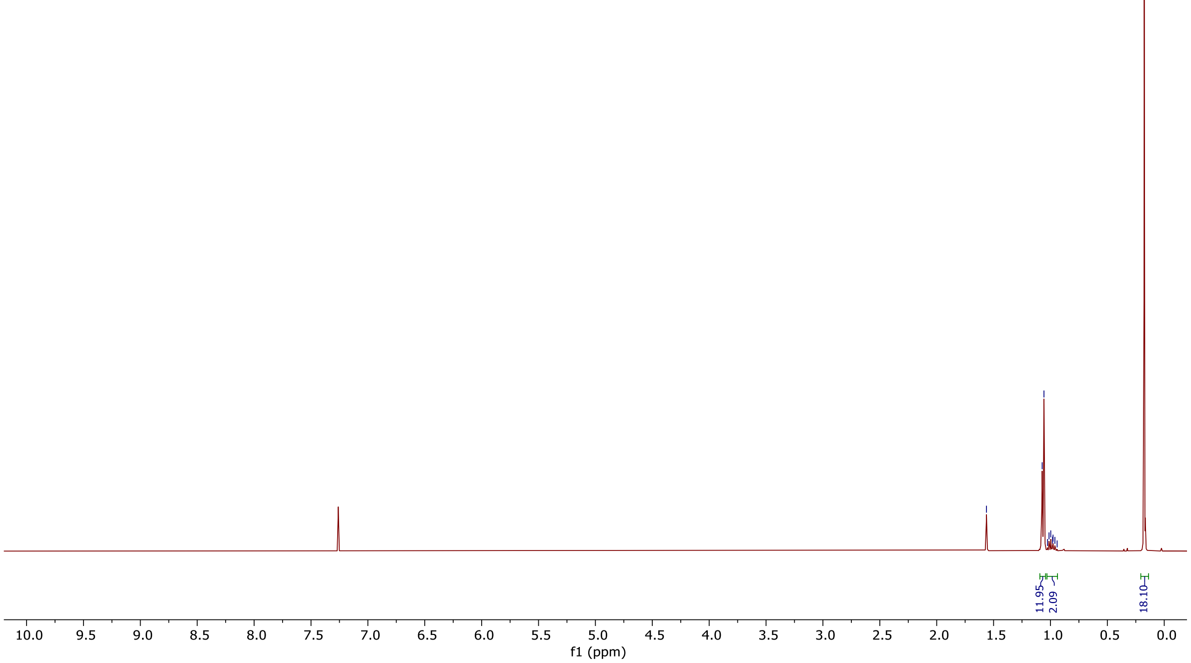

13C NMR (CDCl3, 101 MHz)

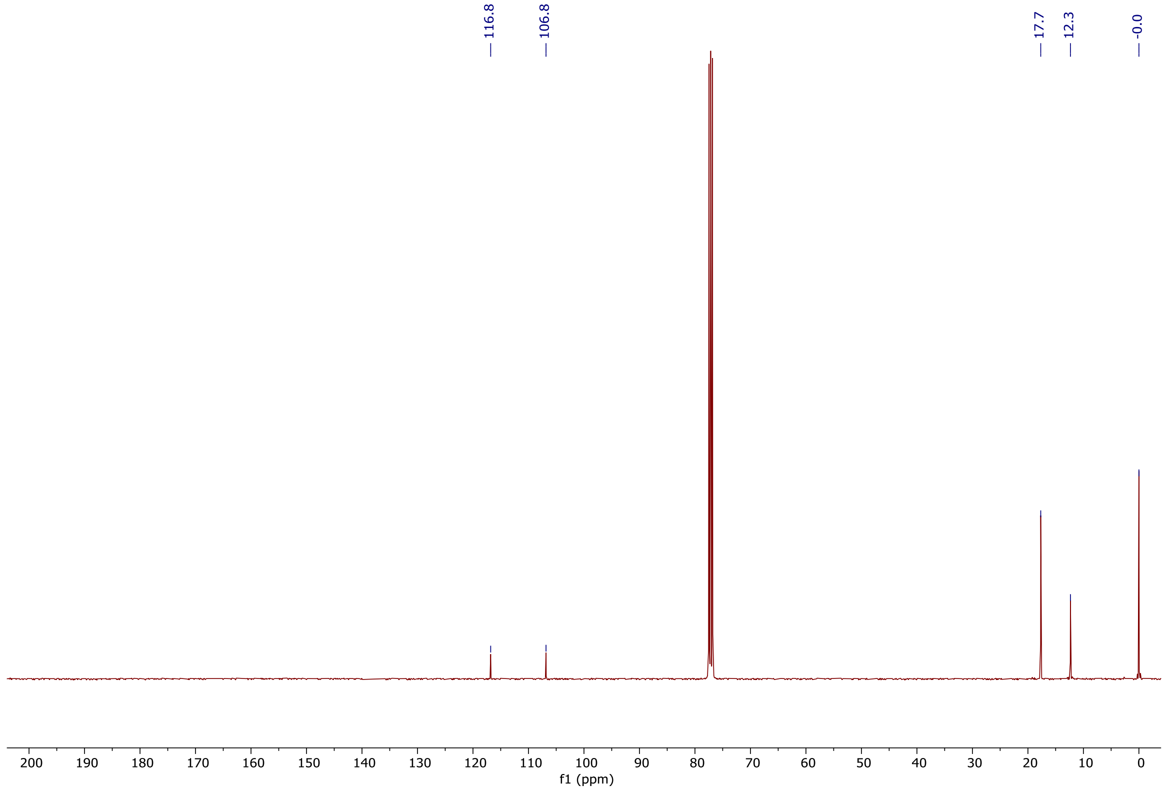

1H NMR (CDCl3, 400 MHz)

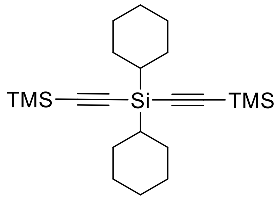

11d

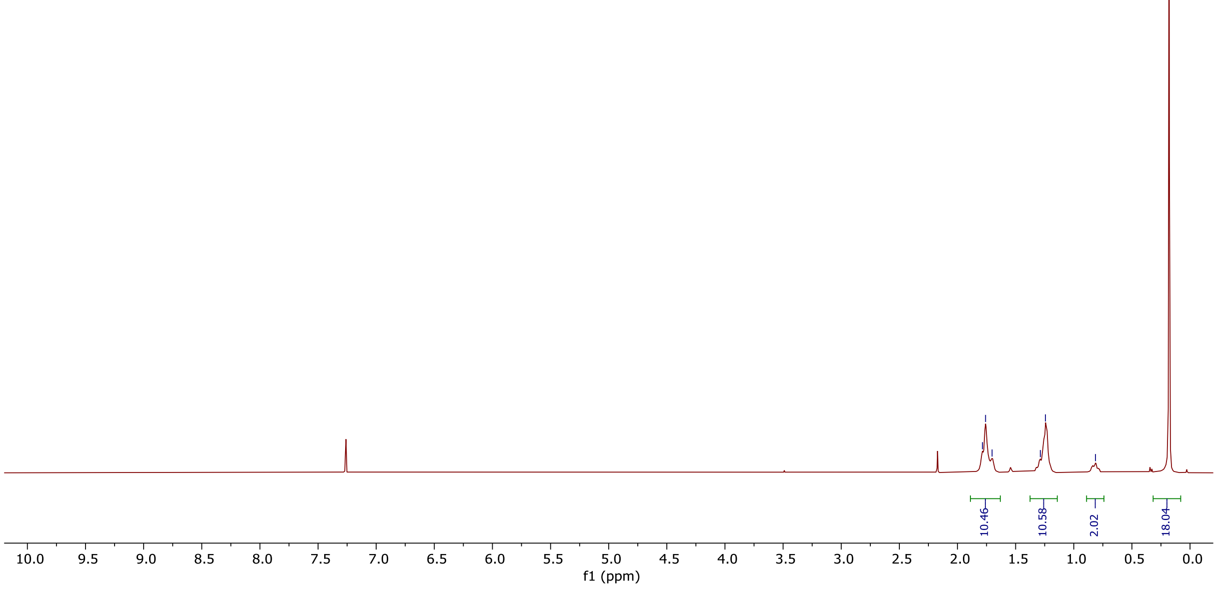

13C NMR (CDCl3, 101 MHz)

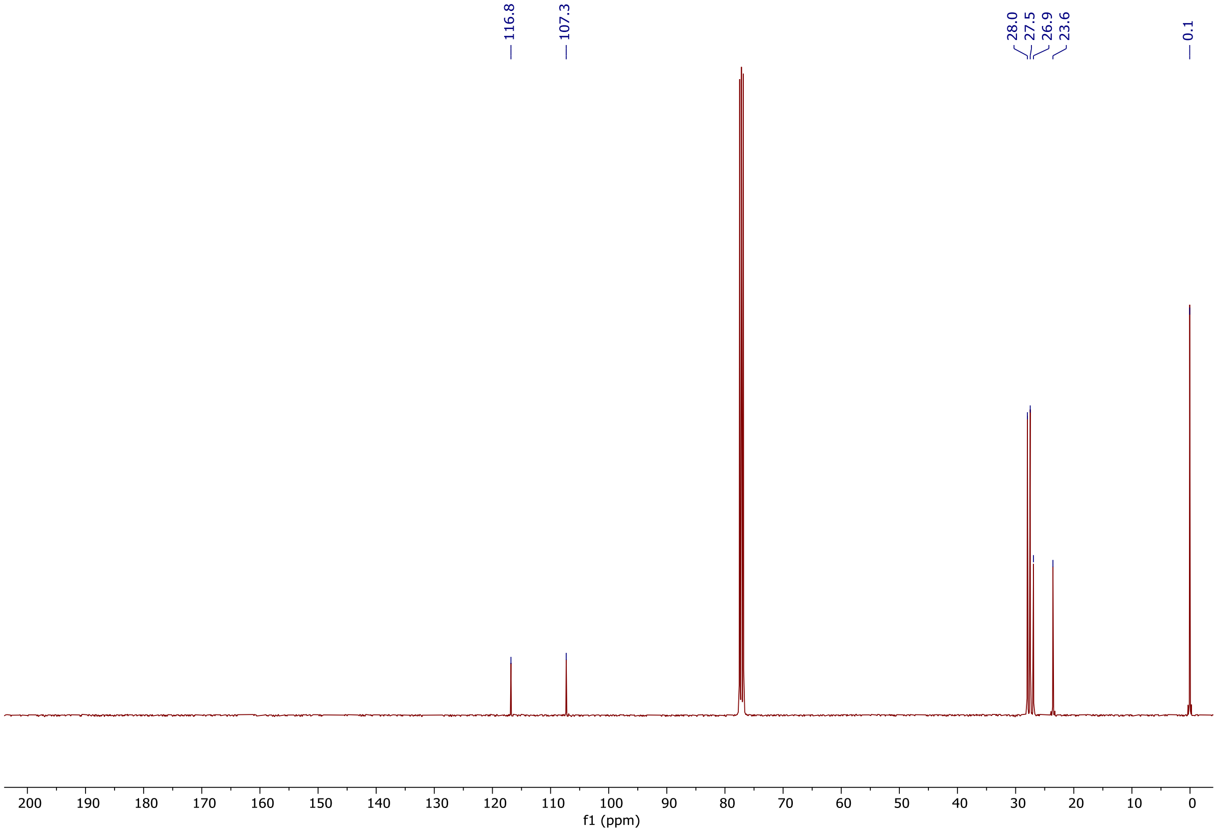

1H NMR (CDCl3, 400 MHz)

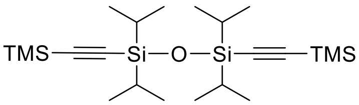

11e

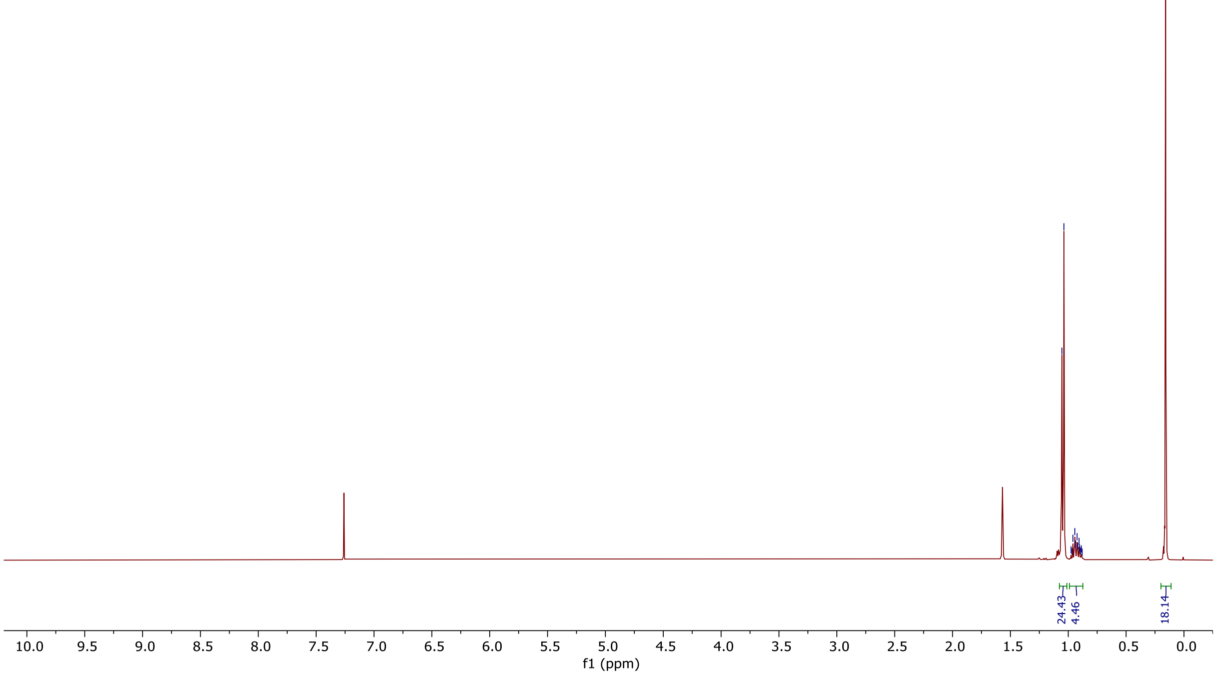

13C NMR (CDCl3, 101 MHz)

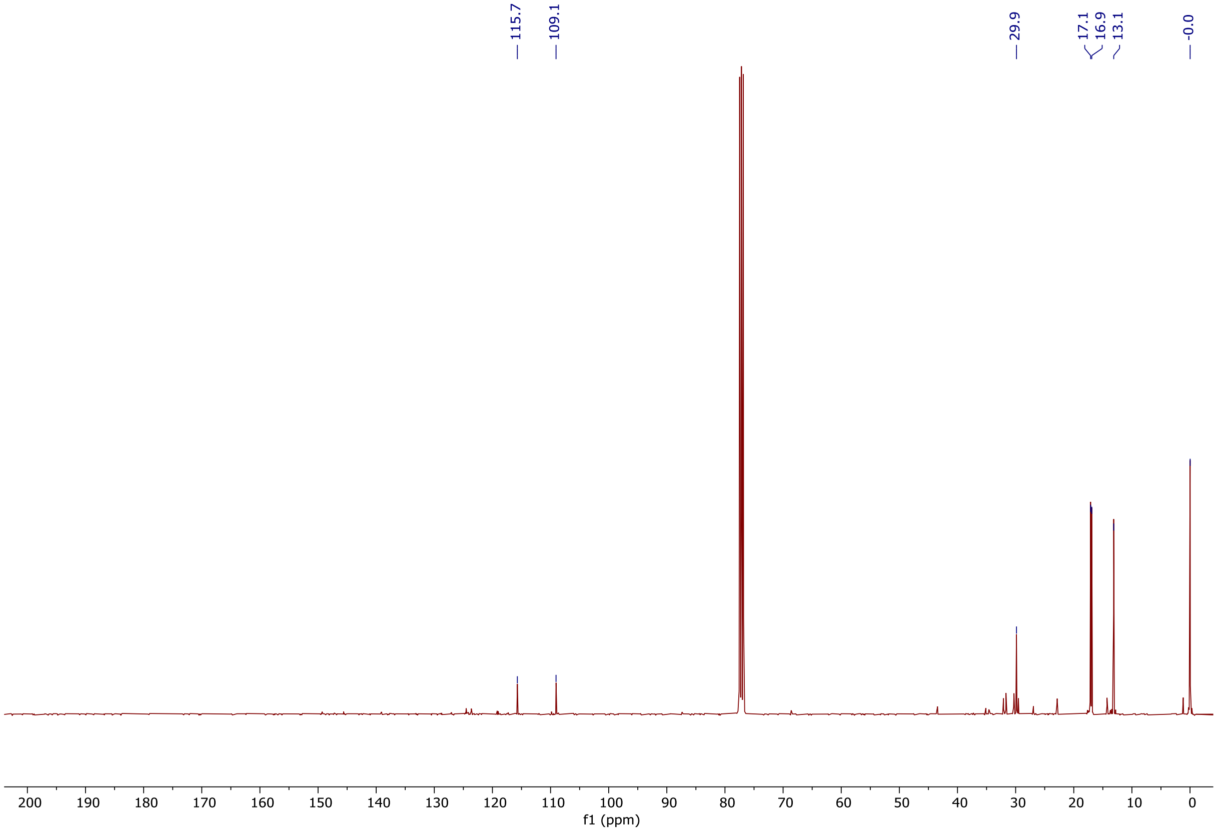

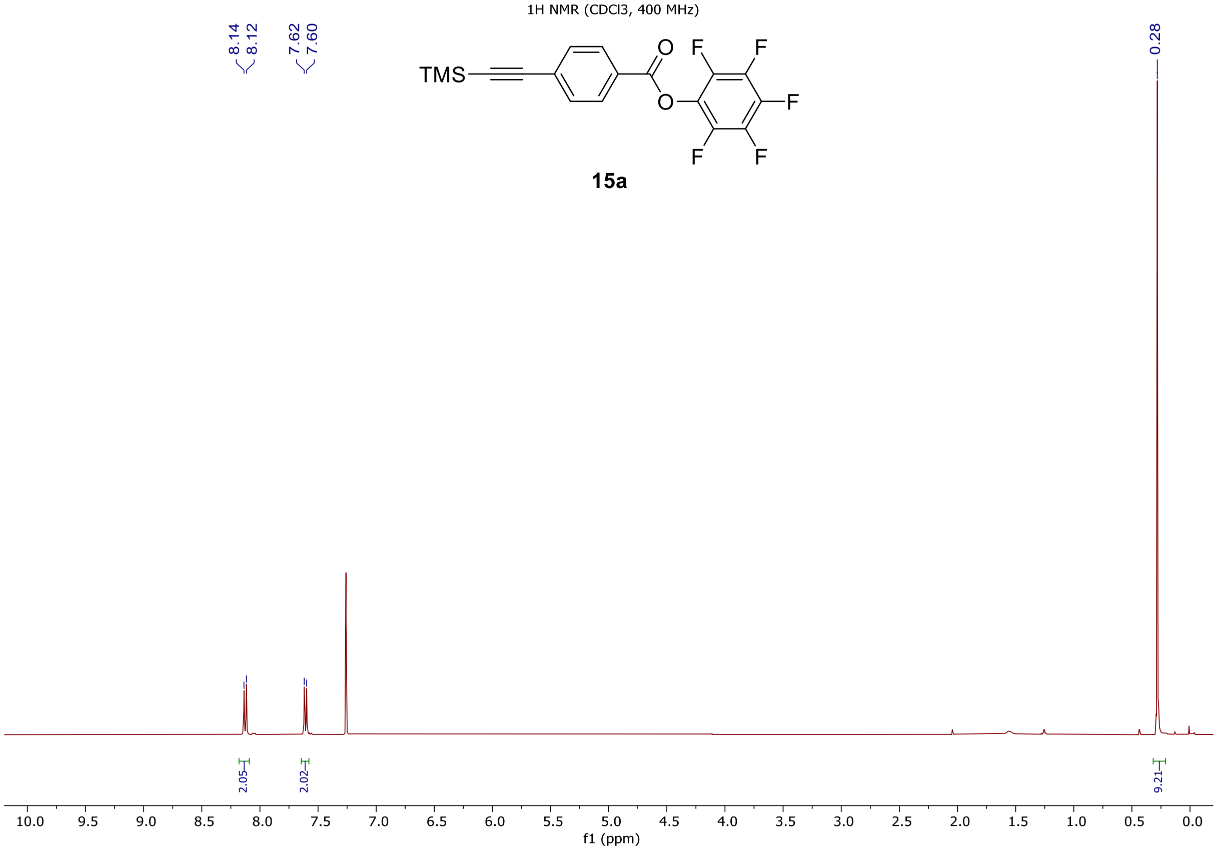
1H NMR (CDCl3, 400 MHz)
8.14
8.12
7.62
7.60
0.28
TMS
O
O
F
F
F
F
F
15a
2.05
2.02
9.21
10.0 9.5 9.0 8.5 8.0 7.5 7.0 6.5 6.0 5.5 5.0 4.5 4.0 3.5 3.0 2.5 2.0 1.5 1.0 0.5 0.0
f1 (ppm)

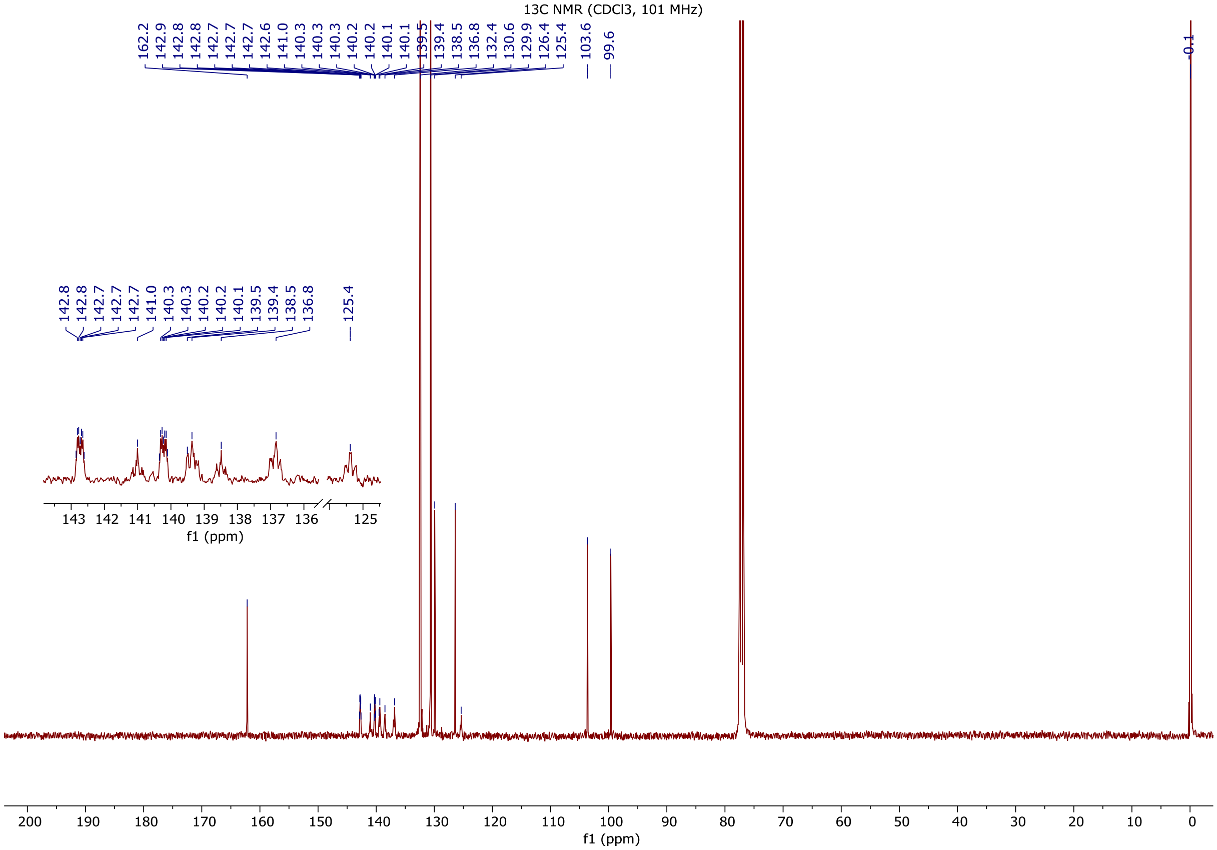
13C NMR (CDCl3, 101 MHz)
162.2 142.9 142.8 142.8 142.7 142.7 142.7 142.6 141.0 140.3 140.3 140.3 140.2 140.2 140.1 140.1 139.5 139.4 138.5 136.8 132.4 130.6 129.9 126.4 125.4
103.6
99.6
-0.1
142.8 142.8 142.7 142.7 142.7 141.0 140.3 140.3 140.2 140.2 140.1 139.5 139.4 138.5 136.8
125.4
143 142 141 140 139 138 137 136 125
f1 (ppm)
200 190 180 170 160 150 140 130 120 110 100 90 80 70 60 50 40 30 20 10 0
f1 (ppm)

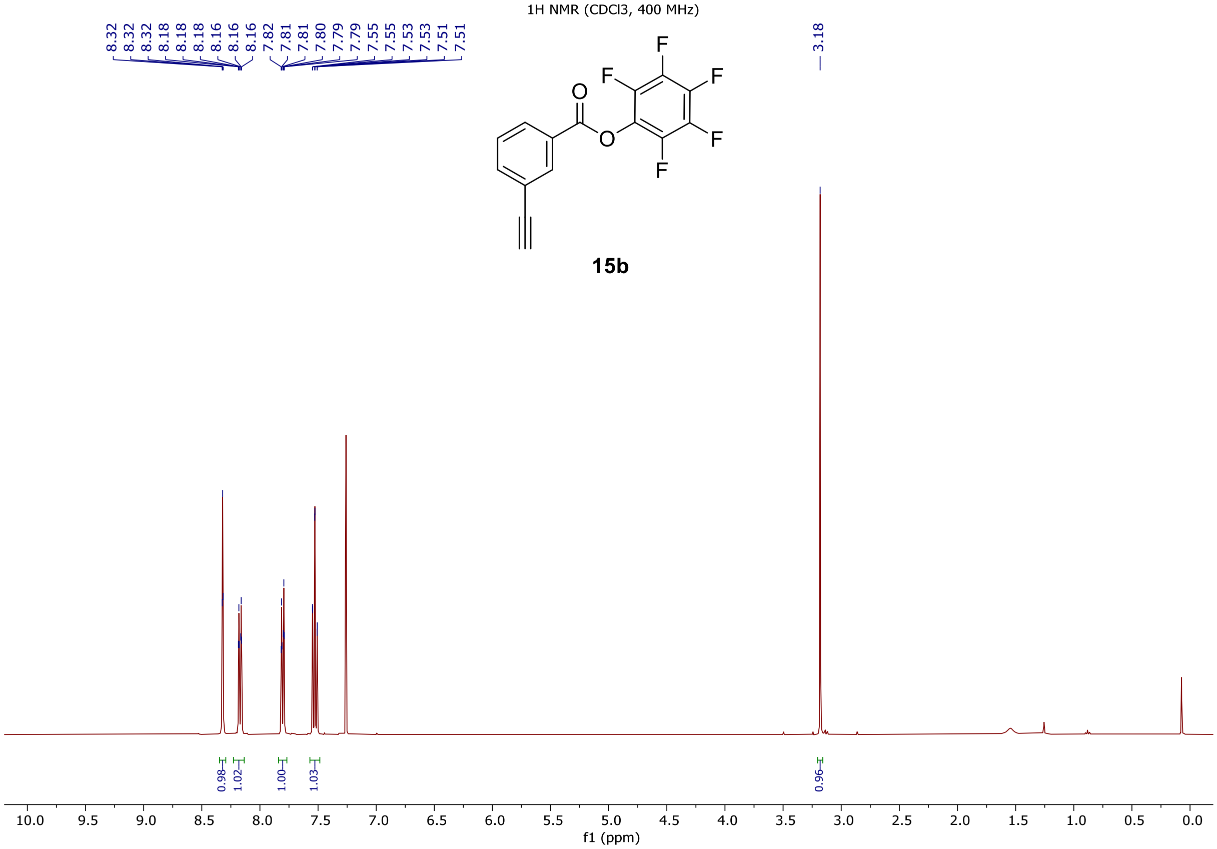

1H NMR (CDCl3, 400 MHz)
8.32 8.32 8.32 8.18 8.18 8.18 8.16 8.16 8.16 7.82 7.81 7.81 7.80 7.79 7.79 7.55 7.55 7.53 7.53 7.51 7.51
3.18
F F F F F O O
15b
0.98 1.02 1.00 1.03 0.96
10.0 9.5 9.0 8.5 8.0 7.5 7.0 6.5 6.0 5.5 5.0 4.5 4.0 3.5 3.0 2.5 2.0 1.5 1.0 0.5 0.0
f1 (ppm)

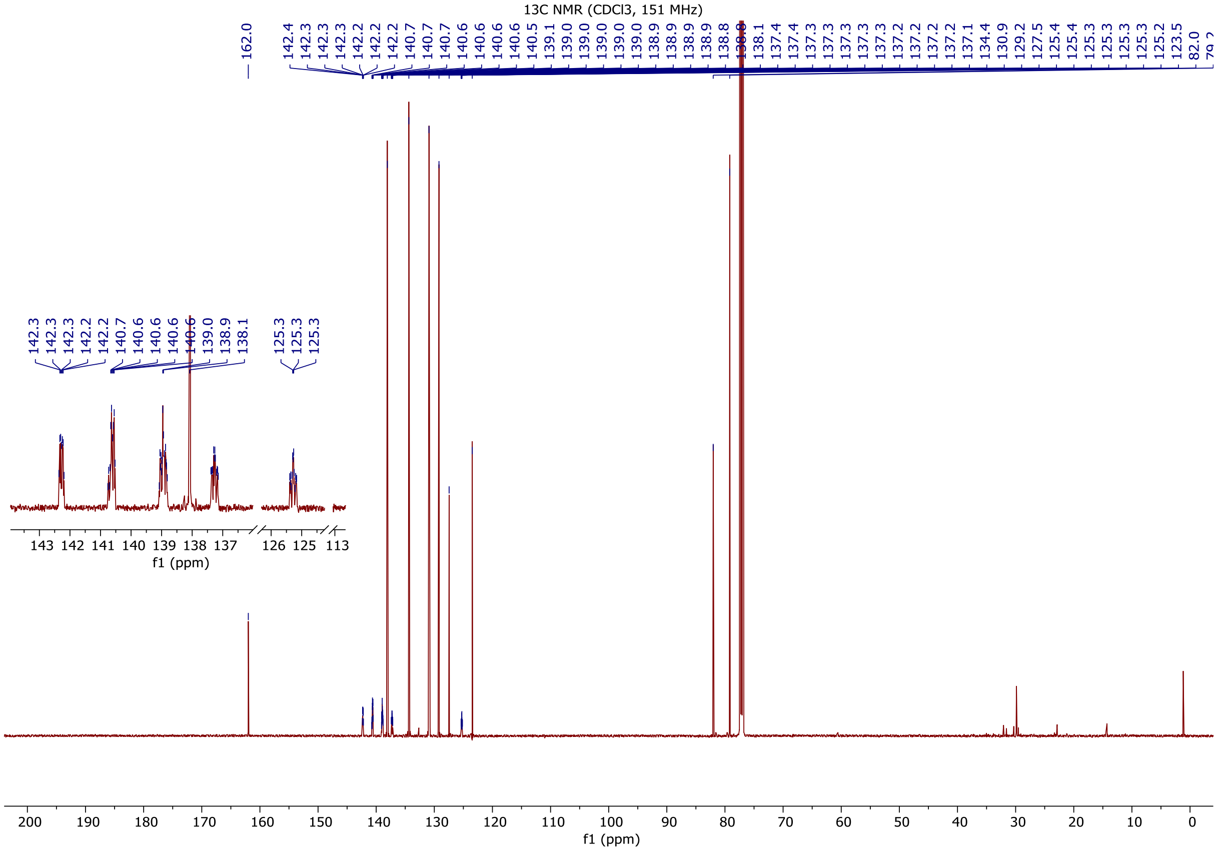

13C NMR (CDCl3, 151 MHz)
162.0
142.4 142.3 142.3 142.3 142.2 142.2 142.2 142.2 140.7 140.7 140.7 140.7 140.6 140.6 140.6 140.6 140.5 139.1 139.0 139.0 139.0 139.0 139.0 138.9 138.9 138.9 138.9 138.9 138.8 138.1 137.4 137.4 137.3 137.3 137.3 137.3 137.3 137.2 137.2 137.2 137.2 137.1 134.4 130.9 129.2 127.5 125.4 125.4 125.3 125.3 125.3 125.3 125.2 123.5 82.0
142.3 142.3 142.3 142.2 142.2 142.2 140.7 140.6 140.6 140.6 140.6 139.0 138.9 138.1
125.3 125.3 125.3
143 142 141 140 139 138 137 126 125 113
f1 (ppm)
200 190 180 170 160 150 140 130 120 110 100 90 80 70 60 50 40 30 20 10 0
f1 (ppm)

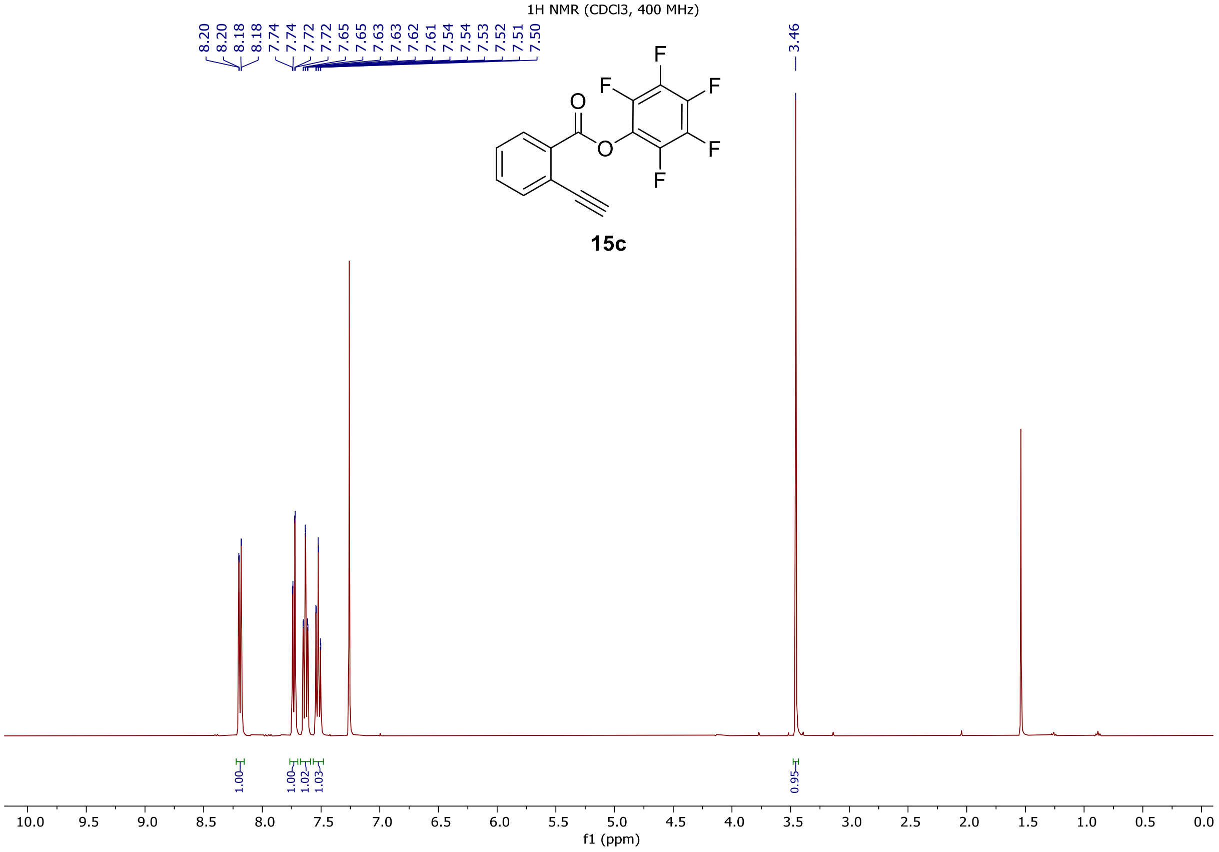
1H NMR (CDCl3, 400 MHz)
8.20
8.20
8.18
8.18
7.74
7.74
7.72
7.72
7.65
7.65
7.63
7.63
7.62
7.61
7.54
7.54
7.53
7.52
7.51
7.50
3.46
O
O
F
F
F
F
F
15c
1.00
1.00
1.02
1.03
0.95
f1 (ppm)

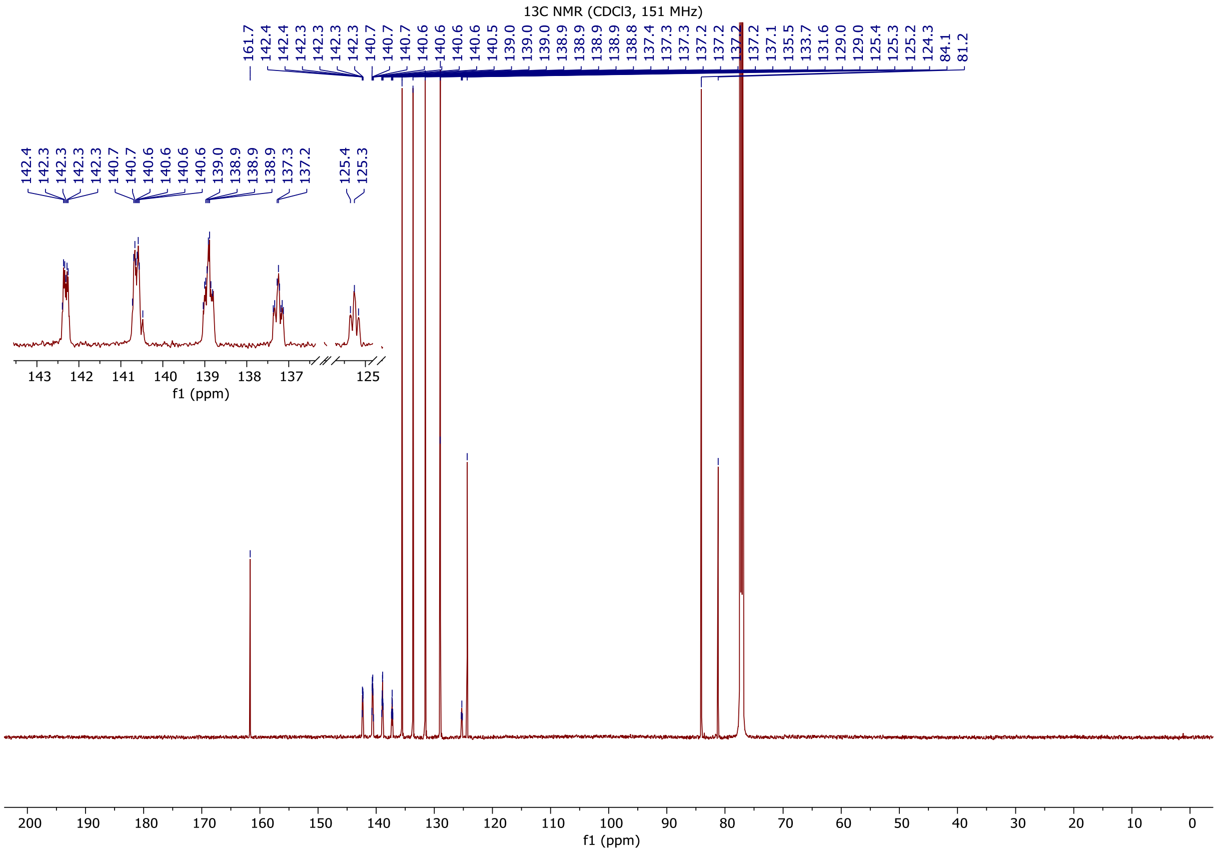
13C NMR (CDCl3, 151 MHz)
161.7
142.4
142.4
142.3
142.3
142.3
142.3
140.7
140.7
140.7
140.6
140.6
140.6
140.5
140.5
139.0
139.0
138.9
138.9
138.9
138.8
137.4
137.3
137.3
137.2
137.2
137.2
137.2
137.1
135.5
133.7
131.6
129.0
129.0
125.4
125.3
125.2
124.3
84.1
81.2
f1 (ppm)

1H NMR (CDCl3, 400 MHz)

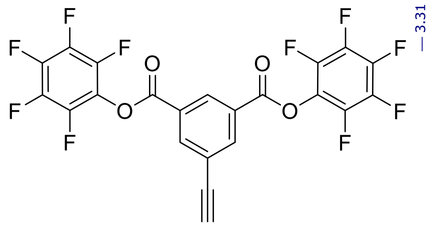

15d

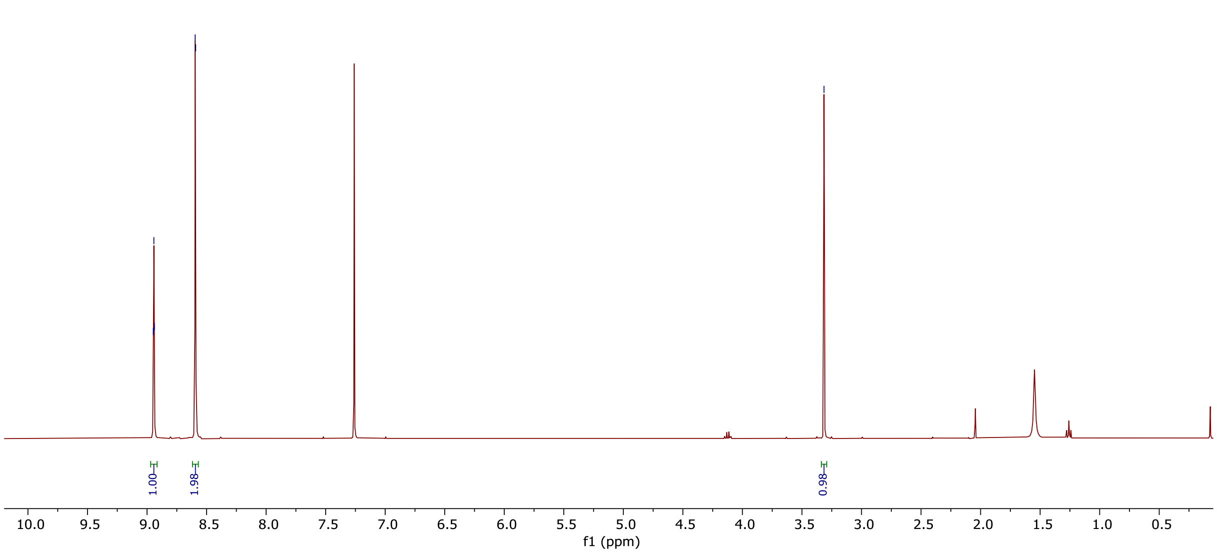

13C NMR (CDCl3, 101 MHz)

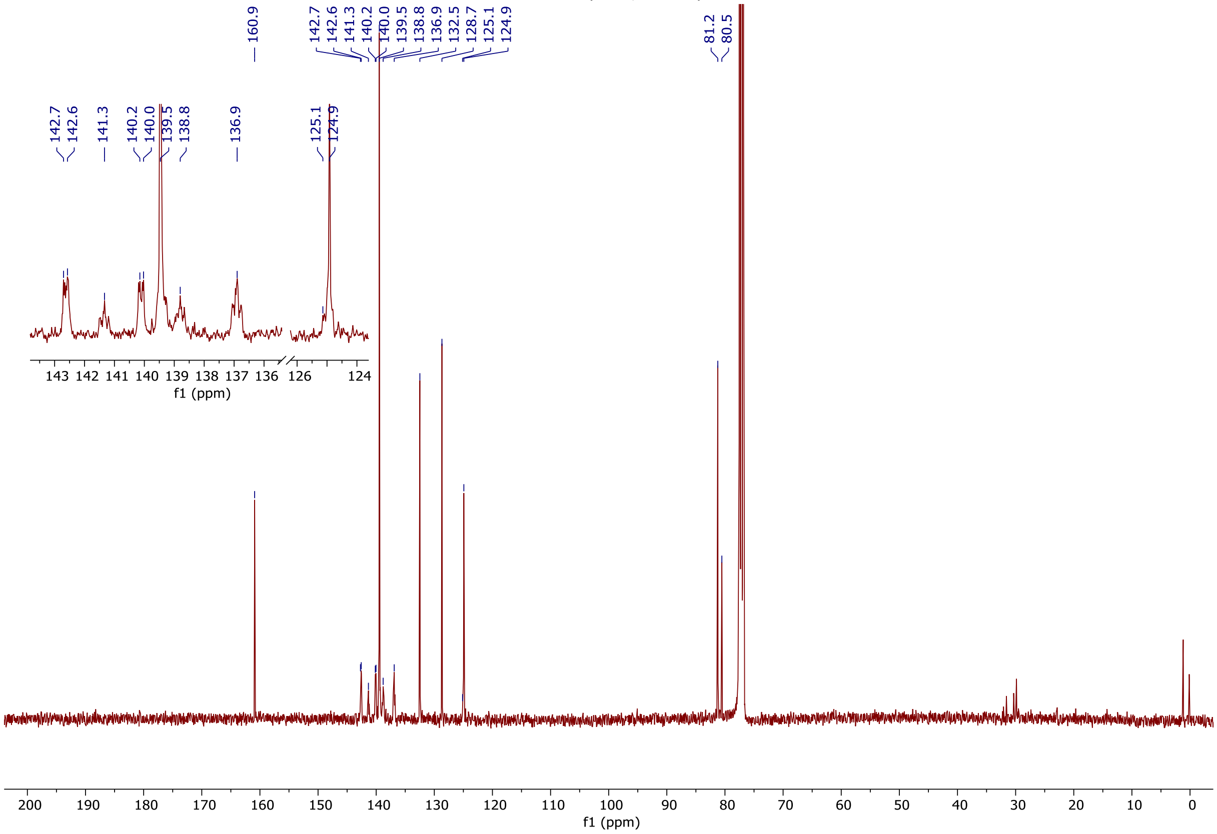

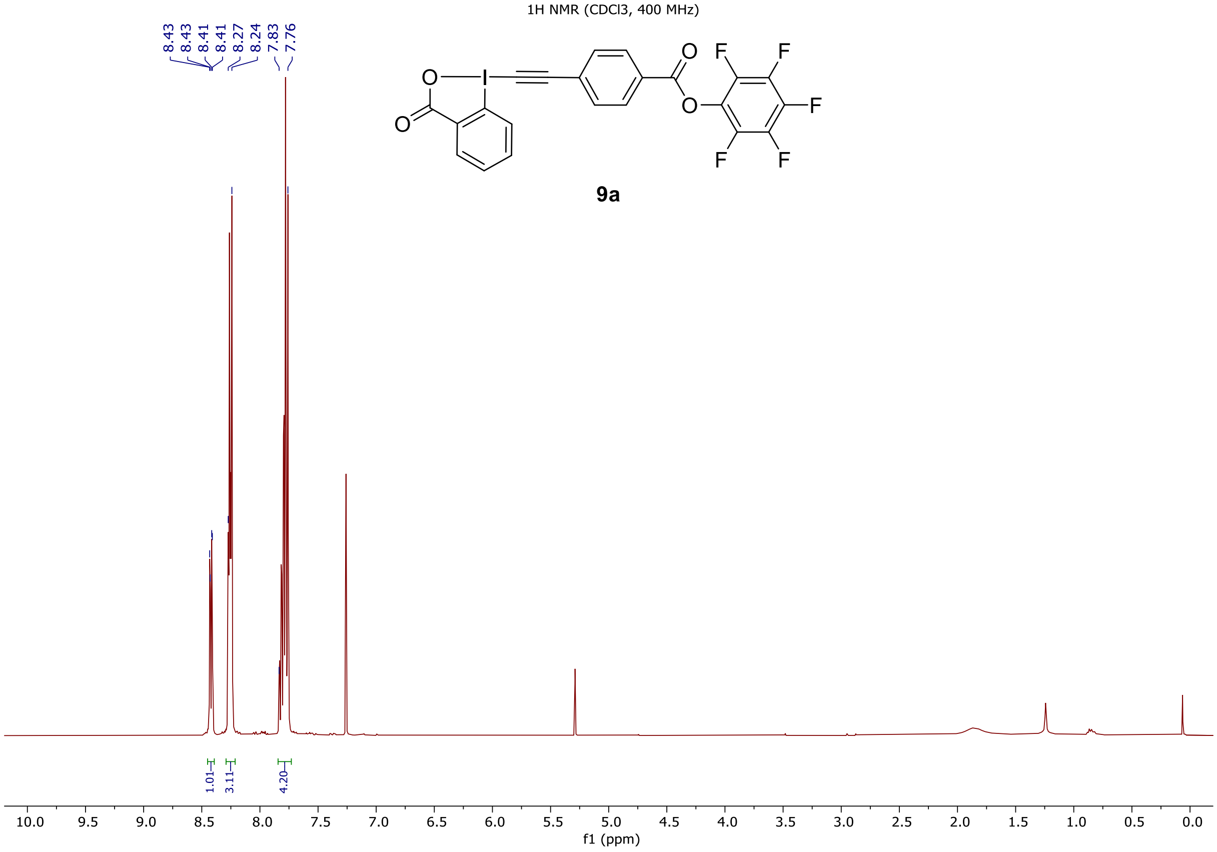
1H NMR (CDCl3, 400 MHz)
8.43
8.43
8.41
8.41
8.27
8.24
7.83
7.76
9a
1.01
3.11
4.20
f1 (ppm)

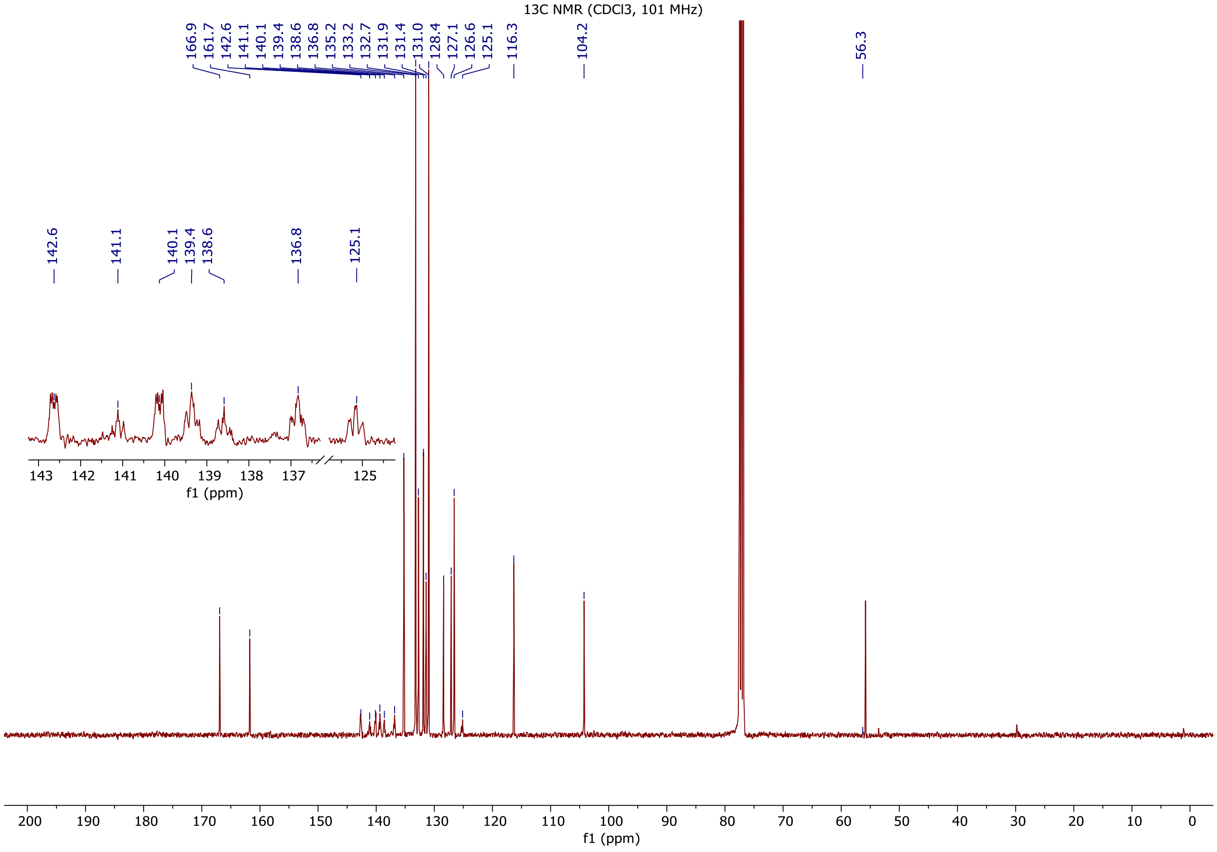
13C NMR (CDCl3, 101 MHz)
166.9
161.7
142.6
141.1
140.1
139.4
138.6
136.8
135.2
133.2
132.7
131.9
131.4
131.0
128.4
127.1
126.6
125.1
116.3
104.2
56.3
f1 (ppm)

1H NMR (CDCl3, 400 MHz)

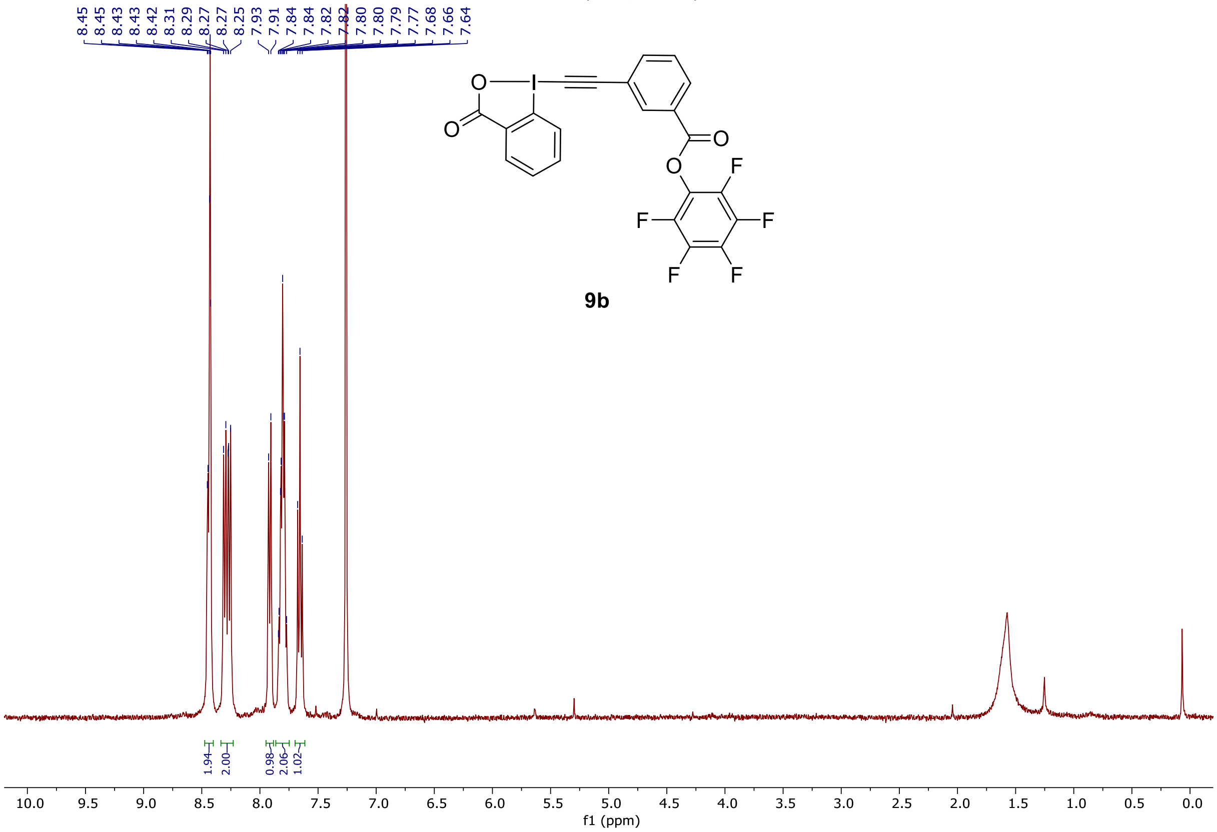

13C NMR (CDCl3, 151 MHz)

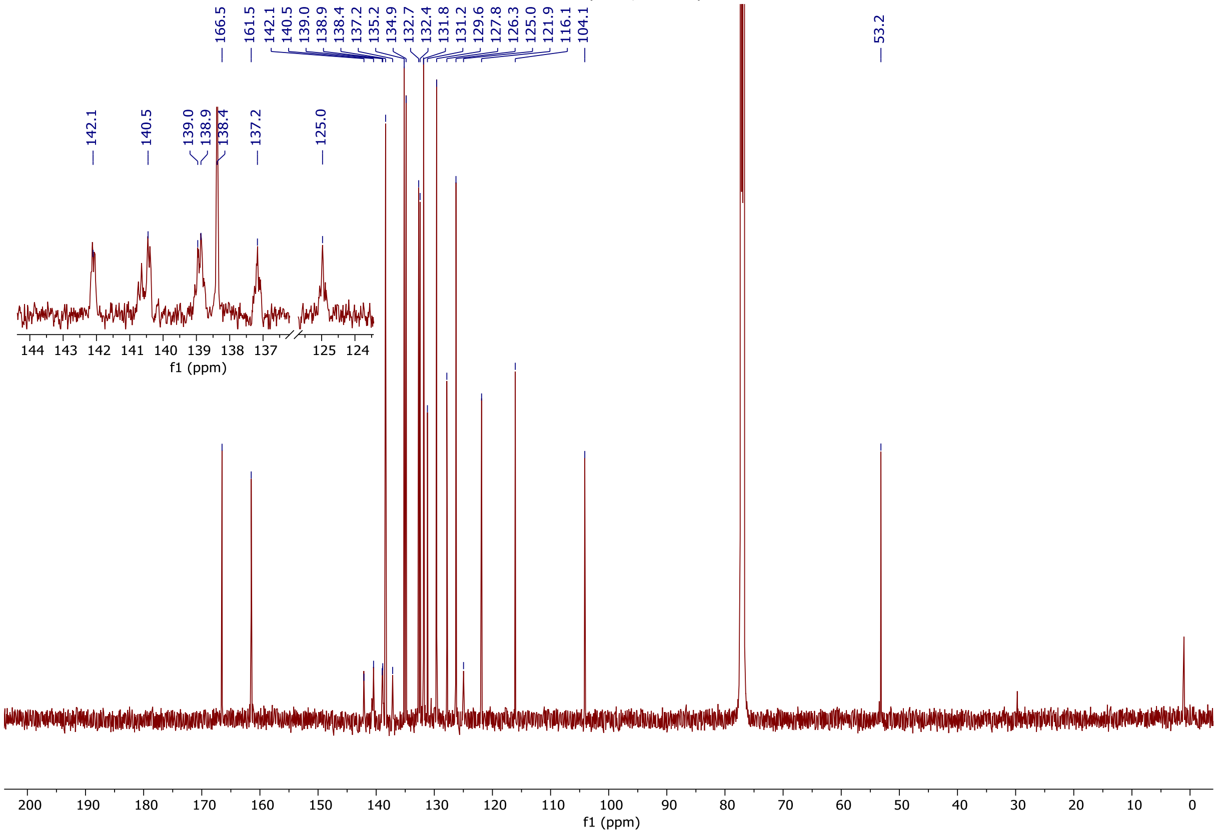

1H NMR (CDCl3, 400 MHz)

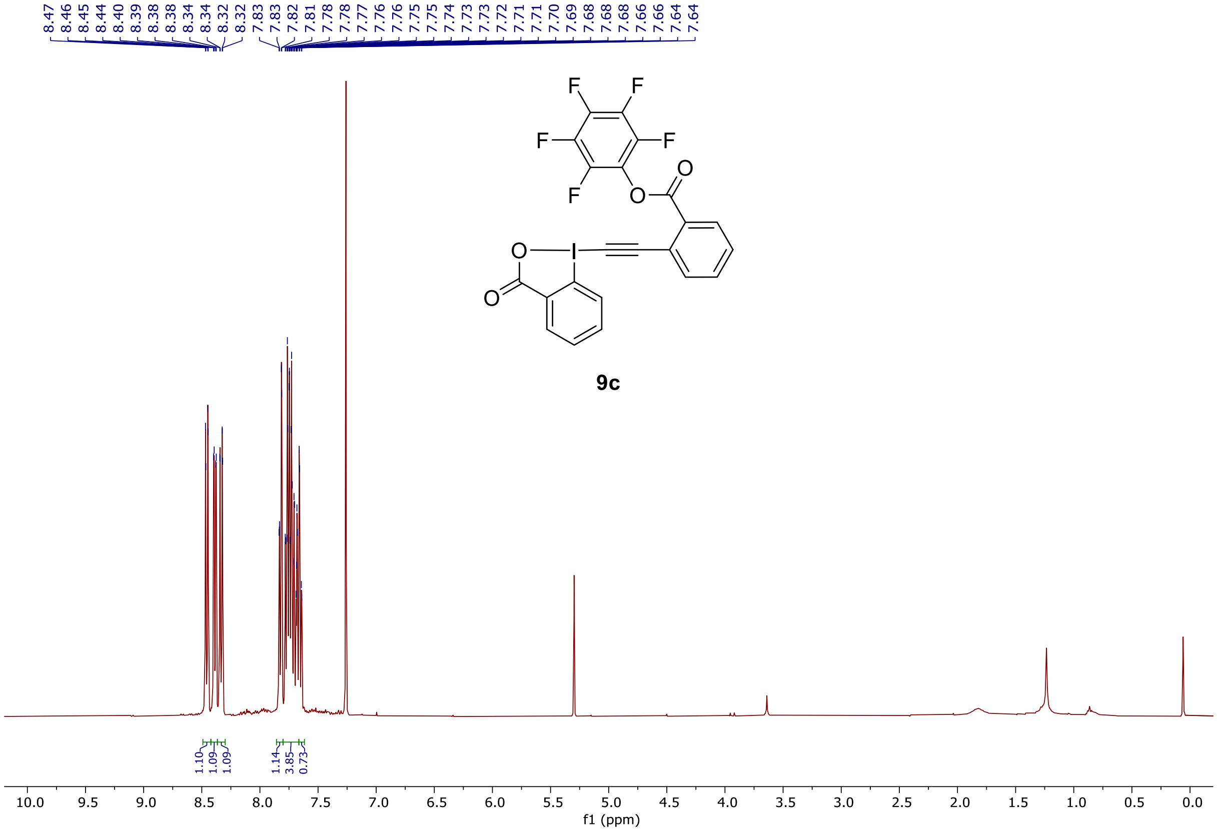

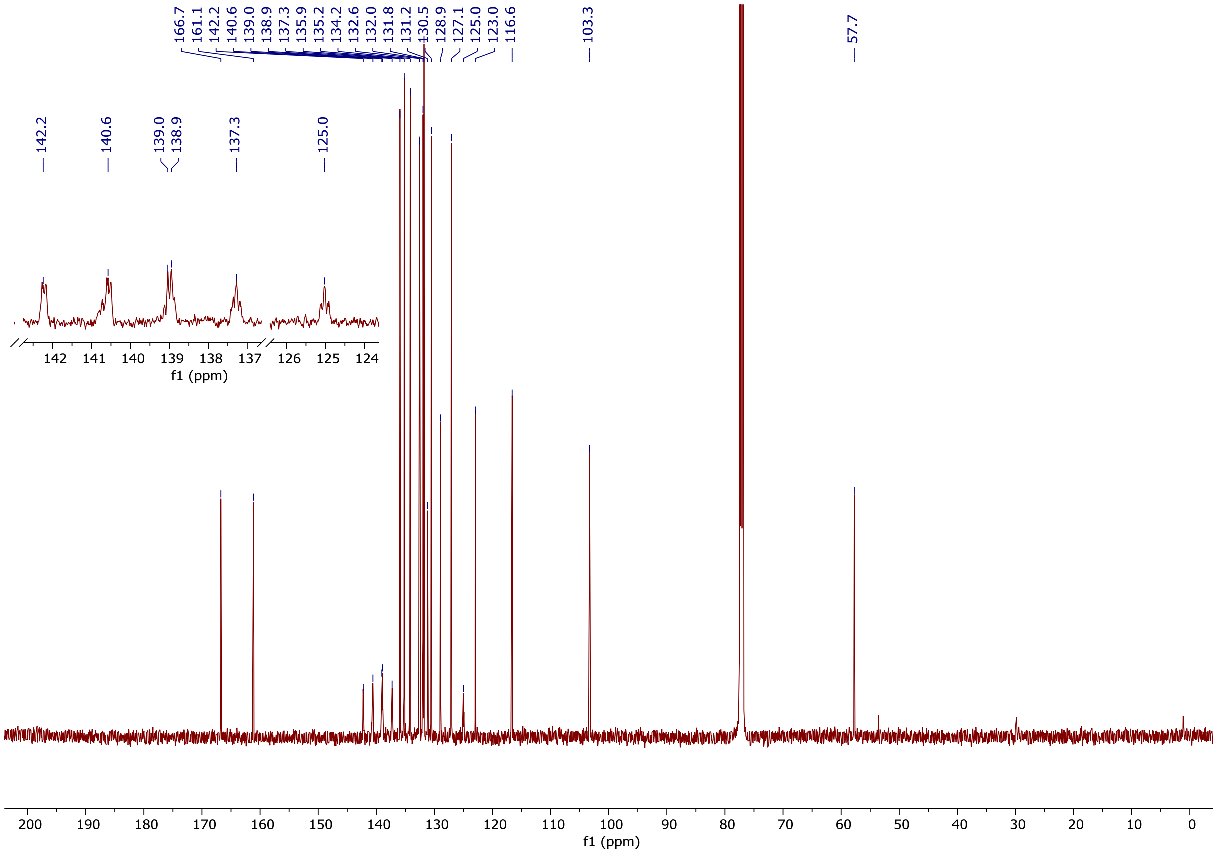

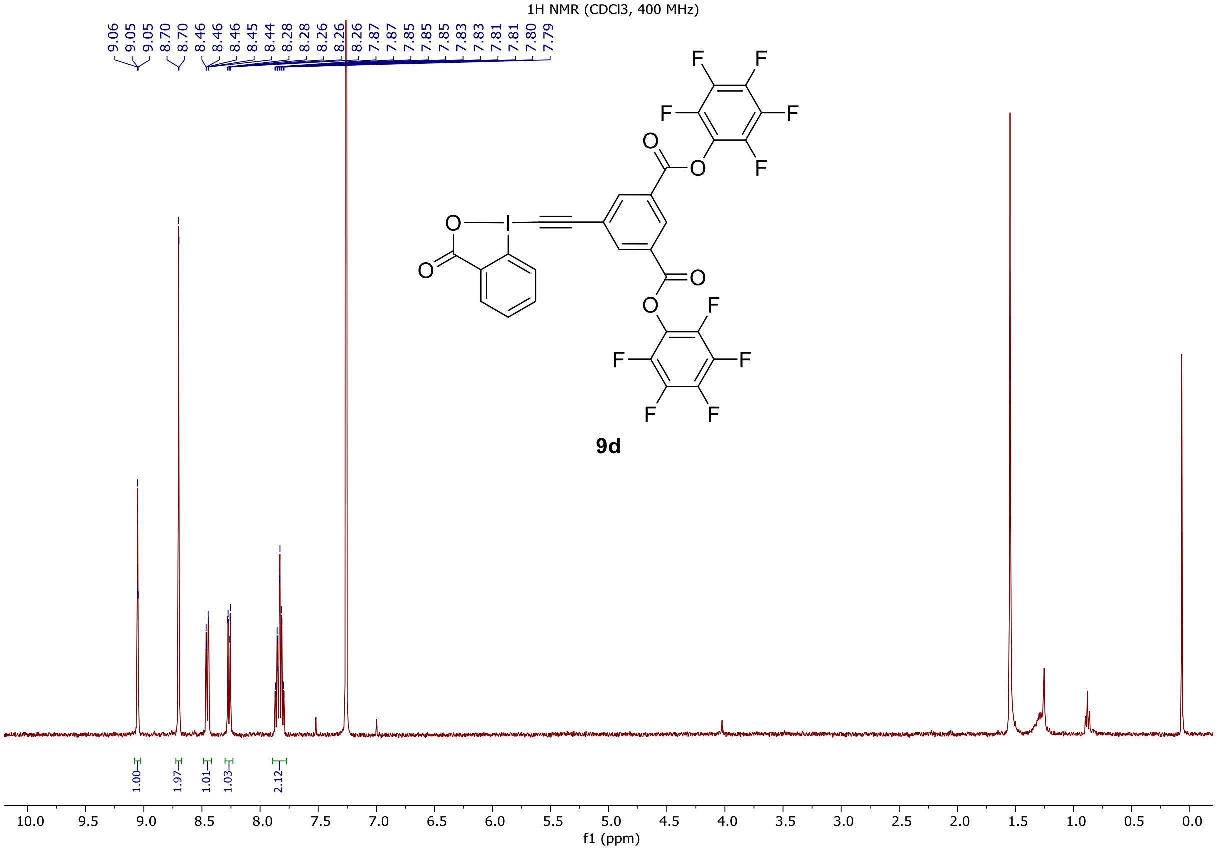
1H NMR (CDCl3, 400 MHz)
9.06 9.05 9.05 8.70 8.70 8.46 8.46 8.45 8.44 8.28 8.28 8.26 8.26 7.87 7.87 7.85 7.85 7.83 7.83 7.81 7.80 7.79
9d
1.00 1.97 1.01 1.03 2.12
10.0 9.5 9.0 8.5 8.0 7.5 7.0 6.5 6.0 5.5 5.0 4.5 4.0 3.5 3.0 2.5 2.0 1.5 1.0 0.5 0.0
f1 (ppm)

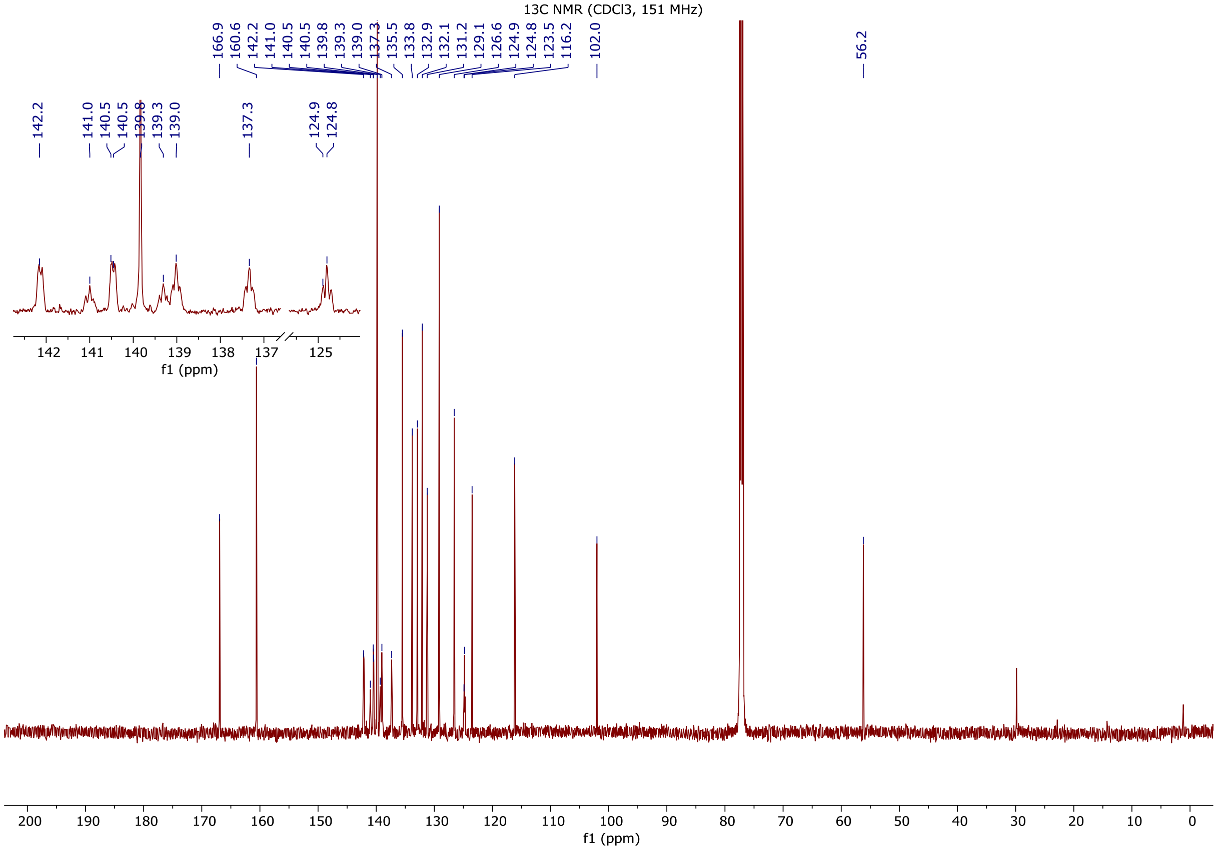
13C NMR (CDCl3, 151 MHz)
166.9 160.6 142.2 141.0 140.5 140.5 139.8 139.3 139.0 137.3 135.5 133.8 132.9 132.1 131.2 129.1 126.6 124.9 124.8 123.5 116.2 102.0 56.2
142.2 141.0 140.5 140.5 139.8 139.3 139.0 137.3 124.9 124.8
142 141 140 139 138 137 125
f1 (ppm)
200 190 180 170 160 150 140 130 120 110 100 90 80 70 60 50 40 30 20 10 0
f1 (ppm)

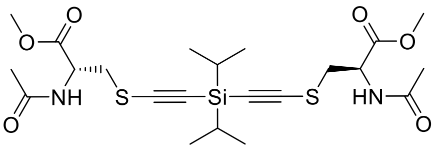

13

1H NMR (CDCl3, 400 MHz)

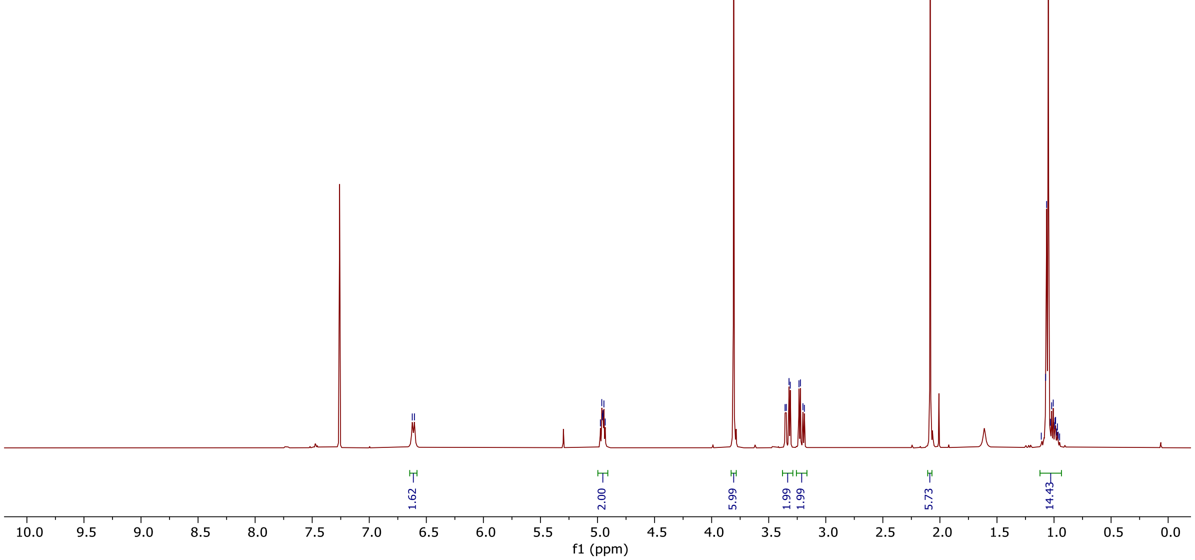

13C NMR (CDCl3, 101 MHz)

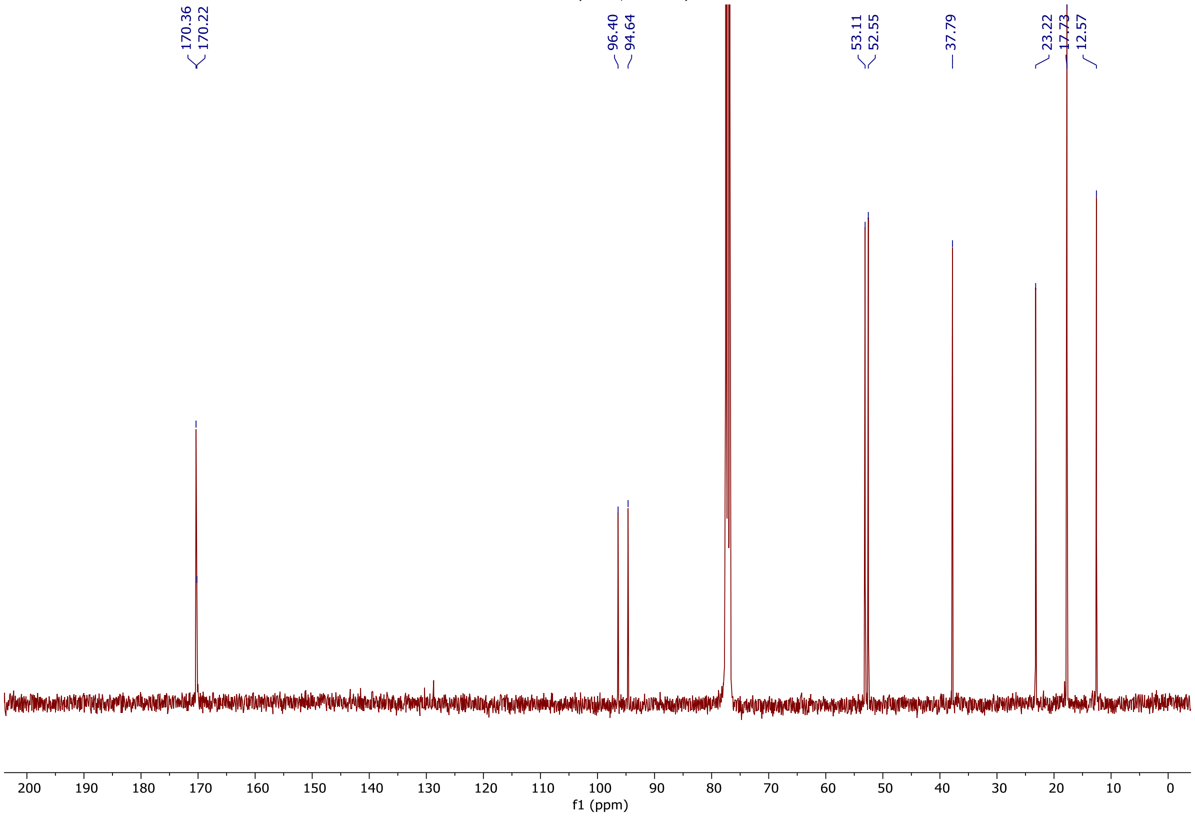

Supplement: Supplementary file 1 — Supplementary [file ANIE-60-9022-s001.pdf]
